# The Hexameric Resorcinarene Capsule as a Brønsted Acid Catalyst for the Synthesis of Bis(heteroaryl)methanes in a Nanoconfined Space

**DOI:** 10.3389/fchem.2019.00687

**Published:** 2019-10-22

**Authors:** Stefania Gambaro, Pellegrino La Manna, Margherita De Rosa, Annunziata Soriente, Carmen Talotta, Carmine Gaeta, Placido Neri

**Affiliations:** Laboratory of Supramolecular Chemistry, Dipartimento di Chimica e Biologia “A. Zambelli”, Università degli Studi di Salerno, Salerno, Italy

**Keywords:** supramolecular organocatalysis, resorcinarene hexameric capsule, bis(heteroaryl)methanes, self-assembly, H-bond catalyst, Brønsted acid catalyst

## Abstract

Herein, we show that the hexameric resorcinarene capsule **C** is able to catalyze the formation of bis(heteroaryl)methanes by reaction between pyrroles or indoles and carbonyl compounds (α-ketoesters or aldehydes) in excellent yields and selectivity. Our results suggest that the capsule can play a double catalytic role as a H-bond catalyst, for the initial activation of the carbonyl substrate, and as a Brønsted acid catalyst, for the dehydration of the intermediate alcohol.

## Introduction

Supramolecular organocatalysis is an emerging area in supramolecular chemistry whose principal aim is the design of novel systems able to perform catalytic functions mimicking the chemo-, regio-, and stereoselectivity of the natural enzymes (Conn and Rebek, 1997). At this regard, much attention has been focused on designing self-assembled molecular capsules (MCs) able to catalyze organic reaction by confinement of the reactants in their internal cavity (Borsato and Scarso, 2016; Catti et al., 2016; Gaeta et al., 2019). MCs are self-assembled structures sealed by weak non-covalent interactions between the single complementary units. Resembling to an enzyme pocket, the nanoconfined space inside a self-assembled molecular capsule allows the formation of a microenvironment with different physical and chemical features with respect to the external medium. In fact, the nanoconfinement of the reactants inside a MC slows down their molecular mobility determining a different stereo- and regiochemical outcome of the reaction with respect to the bulk conditions. Analogously to the natural systems, when the reactants are hosted inside a MC, the proximity effect between them and the stabilization of the intermediates and transition states induces a reaction acceleration.

**Figure 1:**
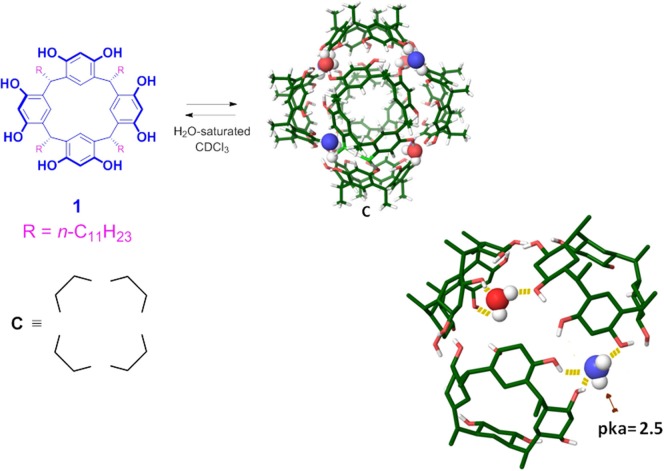
Chemical drawing of the *C*-undecyl-resorcin[4]arene **1**. Tube model of the hexameric capsule **C**, the undecyl chains have been omitted for clarity. Chemical drawing of the model representing the hydrogen bond belt between the eight bridged water molecules and the six resorcinarene molecules, in blue the bridging water molecule with one H-bond donating free valence.

Interestingly, Atwood and MacGillivray reported an interesting example of self-assembled capsule **C** (**1**)_6_·(H_2_O)_8_ (Figure 1; MacGillivray and Atwood, 1997), which is constituted by six resorcin[4]arene units **1** sealed by eight water molecules, and shows an hydrophobic cavity with an internal volume of 1,375 Å^3^. The six resorcinarene units and the eight water molecules are located, respectively, on the sides and on the corners of a cube, and the aggregate is sealed by 60 (O-H^….^O) hydrogen bonding interactions. The 8 bridged-water molecules establish H-bonds with the adjacent resorcinol OH groups and, in particular, four of them act as double H-bonds donor (Figure 1, H_2_O drawing in red) and single H-bond acceptor, saturating in this way their H-bonding valence. The other four bridged-water molecules act as single H-bond acceptor and single H-bond donor (Figure 1, blu), remaining with one H-bond donating free valence. Cohen et al. (Avram and Cohen, 2002b) demonstrated by NMR diffusion experiments, that the capsule **C** is self–assembled also in solution when water-saturated chloroform or benzene is used as a solvent.

The capsule **C** is able to accommodate eight benzene (or chloroform) molecules inside its cavity (Avram and Cohen, 2002a,b, 2004; Shivanyuk and Rebek, 2003). Numerous studies showed that **C** is also able to host in its π-electron rich cavity, complementary guests by H-bonding and/or cation–π interactions (Shivanyuk and Rebek, 2001; Avram and Cohen, 2002a; Yamanaka et al., 2004; Evan-Salem et al., 2006). Tiefenbacher et al. demostrated that **C** behaves as a Brønsted acid (Zhang and Tiefenbacher, 2013; Köster and Tiefenbacher, 2018). In particular, their studies revealed that the hexameric aggregate has an estimated pK_a_ value of about 5.5–6.0, a value certainly not comparable with that of the single resorcinarene unit. The acidic behavior of **C** is explained by the stabilization of its conjugate-base due to the delocalization of its negative charge over the phenolic groups and water molecules of the assembly. QM calculations, recently reported by our group (La Manna et al., 2018b) estimated a local p*K*_a_ of ≈2.5 for the bridged-water molecules with one H-bond donating free valence (in blue in Figure 1), while the mean p*K*_a_ value of all OH groups of **C** is 6.1, in agreement with the experimental datum.

Several reports clearly show that the mild Brønsted acidity of **C** and its ability to stabilize cationic transition states, are crucial factors for the catalytic activity of the capsule (Borsato and Scarso, 2016; Catti et al., 2016; Gaeta et al., 2019). Thus, amazing results have been reported in the last decade regarding the catalysis of chemical reactions into the nanoconfined space of the self-assembled capsule **C**, including the cyclization of terpenes (Zhang and Tiefenbacher, 2015, 2019; Zhang et al., 2017, 2018, 2019; Pahima et al., 2019), the hydration of the alkynes (La Sorella et al., 2016a), the carbonyl-olefin metathesis (Catti and Tiefenbacher, 2018), the sulfoxidation of thioethers (La Sorella et al., 2016b), the synthesis of substituted 1-H-tetrazoles (Giust et al., 2015), the activation of C-F bonds (Köster et al., 2019), and the iminium catalysis (Bräuer et al., 2017; La Manna et al., 2018a). Recently, we showed that the capsule **C** acts as a nanoreactor for a Friedel-Crafts alkylation of arenes and heteroarenes with benzyl chloride (La Manna et al., 2018b) under mild metal-free conditions. We showed that the bridged-water molecules with one H-bond donating free valence exert a crucial role in the activation of the C-Cl bond of benzyl chloride by H-bonding interaction. Analogously, the H-bond donor abilities of the water molecules of **C** have been exploited in the activation of β-nitrostyrenes toward the Michael reaction using pyrroles and indoles as nucleophiles (Gambaro et al., 2019).

As a part of our research program focused on the extension of the catalytic opportunities offered by the hexameric capsule **C**, we turned our attention to the synthesis of bis(heteroaryl)methanes (BHM) (Palmieri et al., 2010; Shiri et al., 2010; Shiri, 2012). BHM are fundamental building blocks in the synthesis of natural and unnatural porphyrin derivatives (Cho and Lee, 1998; Burrell et al., 2001; Laha et al., 2003). Moreover, they find applications in several fields, ranging from medicine (Sivaprasad et al., 2006; Awuah and You, 2012; Josefsen and Boyle, 2012) to environment and industry (Kursunlu et al., 2012). In particular, bis(indol)methanes (BIM) and bis(pyrrole)methanes, containing two simple or two substituted heteroaryl moieties are molecules with interesting biological properties (Sakemi and Sun, 1991; Gunasekera et al., 1994; Fürstner, 2003; Bao et al., 2005). This class of products is generally obtained by means of strategies relying upon the use of Brønsted (Palmieri et al., 2010; Shiri et al., 2010; Shiri, 2012) and Lewis acids (Ji et al., 2004; Guo et al., 2009; Ling et al., 2019; Qiang et al., 2019; Wu et al., 2019), strong Brønsted acids (Biaggi et al., 2006; Singh et al., 2011; Lucarini et al., 2013; Norouzi et al., 2018; Tran et al., 2018), and electrochemical methods (Du and Huang, 2018).

## Results and Discussion

Prompted by these considerations and considering our interest in the development of novel organocatalytic strategies, we attempted the synthesis of BHMs derivatives by reaction between aromatic heterocycles and aldehydes and pyruvates in the presence of capsule **C** as a Brønsted acid catalyst. At this regard, as a model reaction for investigating the catalytic performance of **C**, we chose the reaction between pyrrole **2a** and ethyl pyruvate **3a** in Table 1.

**Table 1:** Optimization of reaction conditions for the synthesis of BHMs catalyzed by **C**.

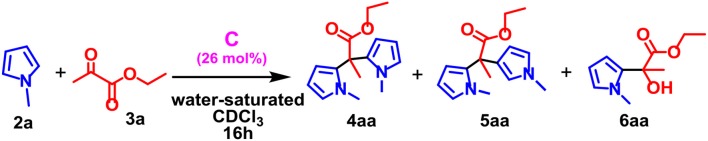							
**Entry^a^**	**Capsule**	**T (°C)**	**2a/3a**	**Yield (%)^b^**	**4aa (%)^c^**	**5aa (%)^c^**	**6aa (%)^c^**
**1**	No	30	1/1	—	—	—	—
	Yes			35	23	4	8
**2**	No	50	1/1	—	—	—	—
	Yes			43	30	4	9
**3**	No	10	1/1	—	—	—	—
	Yes			20	10	5	5
**4**	No	30	2/1	—	—	—	—
	Yes			60	40	5	15
**5**	No	30	4/1	—	—	—	—
	Yes			98	60	10	28

a *Reactions were performed on a 0.16 mmol scale using **2a** (from 1 to 4 equiv.), **3a** (1 equiv.), and capsule **C** (0.26 equiv.) in water saturated CDCl_3_ (1.1 mL) under stirring for 16 h*.

b *Overall yield of all the isolated products*.

c *Yields of the isolated products by chromatography on column*.

We started performing the reaction in Table 1 in the presence of capsule **C** in water-saturated CDCl_3_ at 30°C and with a 1/1 ratio of **2a**/**3a**. It was found that the reaction proceeded smoothly to afford preferentially *meso*-α,α-substituted dipyrromethane **4aa** in 23% yield, accompanied by a negligible amount of α,β-linked dipyrromethane **5aa** and monoalkylated adduct **6aa** (entry 1, Table 1). No evidence was detected of higher oligomers and other side products. In contrast, when the reaction in Table 1 was carried out under the same reaction conditions but in the absence of capsule **C**, no products could be evidenced (entry 1, Table 1). This result encouraged us to carry out a study for the optimisation of the reaction parameters in order to improve the reaction efficiency.

Initially, the influence of the reaction temperature was investigated (Table 1, entries 1–3). When the temperature was decreased to 10°C, both reaction efficiency and selectivity dropped (entry 3, Table 1), while an increase in the temperature had a little positive effect on the reaction outcome (entry 4, Table 1). Next, we moved to examine the molar ratio of **2a**/**3a** on the yield of the reaction in Table 1. When an excess of **2a** was used, an increase of the reaction efficiency in terms of yield was observed while keeping the selectivity for the adducts substantially unchanged, with the preferential formation of **4aa** (entries 4–5, Table 1). These preliminary results indicated that capsule **C** was capable to promote the reaction in selective and efficient way and suggested that the reaction took place inside the cavity of **C**.

In order to confirm this conclusion, and in accord to a protocol previously reported by us and other groups (Bräuer et al., 2017; La Manna et al., 2018a), we performed a series of control experiments. In details, when the reaction between **2a** and **3a** was conducted under the conditions reported in Table 1 in the presence of **C** and of tetraethylammonium tetrafluoroborate, which is a known competitive guest, no hint of products were detected after 16 h at 50°C. Under these conditions, the ammonium guest occupying the cavity of capsule **C** acts as an inhibitor. In addition, the ^1^H NMR spectrum of the reaction mixture in the presence of tetraethylammonium tetrafluoroborate in Figure S3 featured shielded signals at negative chemical shifts values attributable to the cation inside the cavity of **C**. Finally, no hint of products was observed when the reaction reported in Table 1 was performed in the presence of DMSO (Figure S4), a hydrogen-bonding competitor solvent able to disaggregate the capsule **C**.

With these results in hand, we next studied the generality of the reaction with regard to both reactants (Table 2). Initially, we evaluated the influence of the α-ketoester structure on the reaction outcome. When α-ketoester **3c**, bearing an isopropyl group, was reacted with **2a** in the presence of **C** (26 mol%), the formation of the mono-alkylated adduct **6ac** was observed with a yield of 55% (entry 3, Table 2), while no hint of other products was detected. Interestingly, under analogous conditions the α-ketoester **3b** (R = Me) reacted with **2a** giving the *meso*-dipyrromethane product **4ab** (entry 2, Table 2) in 90% yield. Probably, by increasing the steric encumbrance of the R group of **3** from methyl (**3b**) to isopropyl (**3c**) the formation of the di-pyrromethane was hindered. When **3d** (entry 4, Table 2), bearing a benzyloxy group, was used as substrate alongside **2a**, then the formation of the double alkylated adducts α,α and α,β **4ad** and **5ad** was observed in a 1/1 ratio and with a complete loss of selectivity. Differently, using **3b** (entry 2, Table 2) only the α,α adduct **4ab** was obtained. Interestingly, when **3f** bearing an electron-withdrawing trifluoromethyl group was used, the reaction in Table 2 was almost quantitative displaying a complete selectivity for the mono-alkylated adduct **6af** and no evidence of bis-adduct or other side products (entry 6, Table 2). Finally, with α-ketoacid **3e** no reaction took place and a decarboxylate product was recovered.

**Table 2:** Scope of the reaction between different pyrroles **2a–d** and α-ketoesters **3a–f**.

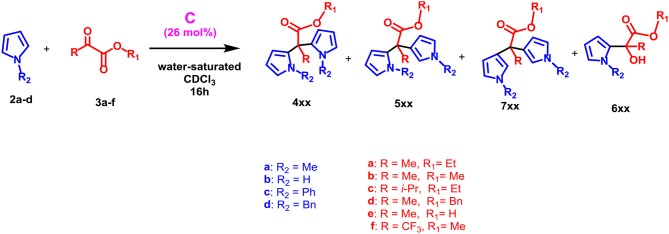								
**Entry^a^**	**Capsule**	**2**	**3**	**Yield (%)^b^**	**% (4xx)^c^**	**% (5xx)^c^**	**% (6xx)^c^**	**% (7xx)^c^**
	No			—	—	—	—	—
**1**		**2a**	**3a**					
	Yes			98	60 (**4aa**)	10 (**5aa**)	28 (**6aa**)	—
	No			—	—	—	—	—
**2^d^**		**2a**	**3b**					
	Yes			99	90 (**4ab**)	—	—	—
	No			—	—	—	—	—
**3**		**2a**	**3c**					
	Yes			55	—	—	55 (**6ac**)	—
	No			—	—	—	—	—
**4**		**2a**	**3d**					
	Yes			76	38 (**4ad**)	38 (**5ad**)	—	—
	No			—	—	—	—	—
**5^e^**		**2a**	**3e**					
	Yes			64	—	—	—	—
	No			35	—	—	35 (**6af**)	—
**6**		**2a**	**3f**					
	Yes			99	—	—	99 (**6af**)	—
	No			—	—	—	—	—
**7**		**2b**	**3a**					
	Yes			99	99 (**4ba**)	—	—	—
	No			38	—	—	38 (**6bf**)	—
**8**		**2b**	**3f**					
	Yes			98	—	—	98 (**6bf**)	—
	No			—	—	—	—	—
**9**		**2c**	**3a**					
	Yes			50	—	—	—	50 (**7ca**)
	No			—	—	—	—	—
**10**		**2d**	**3a**					
	Yes			65	—	—	65 (**6da**)	—

a *Reactions were performed on a 0.16 mmol scale using **2a–d** (4 equiv.), **3a–e** (1 equiv.), and capsule **C** (0.26 equiv.) in water saturated CDCl_3_ (1.1 mL) under stirring for 16 h at 30°C*.

b *Overall yield of all the isolated products*.

c *Yields of the isolated products by chromatography on column*.

d *9% of adduct of pyrrole with two molecules of pyruvate is present; see Supporting Information*.

e *Decarboxylated product is present, see Supporting Information*.

At this point, we examined effect of the substitution at the pyrrole nitrogen atom on the reaction outcome. The reaction between pyrrole **2b** and **3a** selectively delivered the *meso* bis-adduct **4ba** in high yield (entry 7, Table 2). Even with pyrrole **2b**, the reaction with **3f** afforded to mono-adduct **6bf** as the only reaction product (entry 8, Table 2), indicating that the choice of the ketoester influenced the outcome of the reaction.

When a more sterically demanding group was introduced on the nitrogen atom of pyrrole, the yield of the reaction in Table 2 decreased and the selectivity of the products was influenced. In fact, when pyrrole **2c**, bearing a *N*-benzyl group, was used with **3a** under the conditions reported in Table 2, then the mono-adduct **6da** was obtained selectively and in good yield (entry 10, Table 2), whereas with *N*-phenyl pyrrole **2d** we observed for the first time the selective formation of a β, β-di-adduct (**7ca**) (entry 9, Table 2). When the reaction was performed using indole derivatives (Table 3), only the formation of di-pyrromethane β, β-**9** was observed in high yield independently of the substituents present on the benzene and pyrrole rings.

**Table 3:** Scope of the reaction with different indoles.

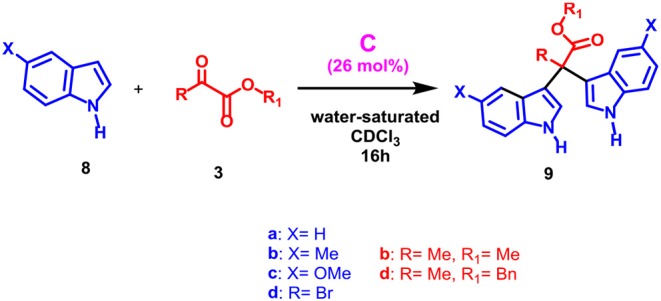				
**Entry^a^**	**Capsule**	**8**	**3**	**Yield (%)^b^**
**1**	No	**8a**	**3b**	—
	Yes			86
**2**	No	**8b**	**3b**	—
	Yes			90
**3**	No	**8c**	**3b**	—
	Yes			88
**4**	No	**8d**	**3b**	—
	Yes			85
**5**	No	**8a**	**3d**	—
	Yes			80

a *Reactions were performed on a 0.16 mmol scale using **8** (4 equiv.), **3** (1 equiv.), and capsule **C** (0.26 equiv.) in water saturated CDCl_3_ (1.1 mL) under stirring for 16 h at 30°C*.

b *Isolated yield*.

**Scheme 1:**
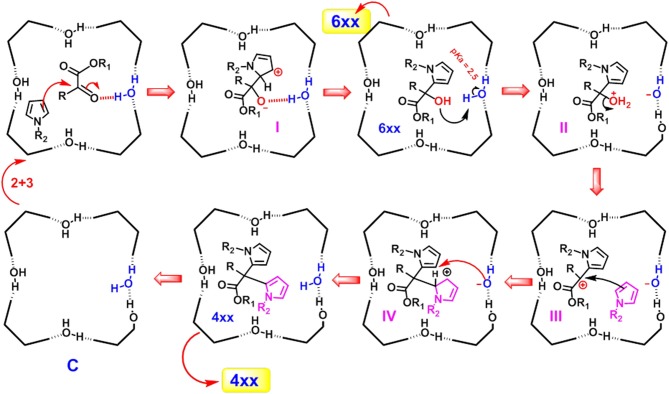
Mechanism proposed for the formation of the products **4xx** and **6xx** in the nano-confined space inside the cavity of **C**.

The mechanism proposed for the formation of α,α-substituted dipyrromethane **4xx** and monoalkylated adduct **6xx** in the nanoconfined space inside the capsule **C**, is outlined in Scheme 1. In detail, α-ketoester **3** is probably stabilized inside the capsule **C** through the formation of a H-bonding interaction with a bridged water molecule (Scheme 1).

Previously, we have already shown that pyrrole derivatives are hosted inside the cavity of **C** (La Manna et al., 2018b). At this point, an α-attack of pyrrole to the activated ketone group of **3** occurs inside the capsule, leading to intermediate **I** (Scheme 1) stabilized through H-bonding and cation···π interactions, which is re-aromatizated to **6xx**. On the basis of the local acidity (p*K*_a_ of ≈ 2.5) of the bridged water molecules with H-bond donating free valence, the product **6xx** can be protonated inside the capsule **C** (**II** in Scheme 1) and converted to carbocation **III** by losing a water molecule. **III** undergoes an α-attack of a new pyrrole molecule to give the carbocation **IV** which is stabilized by cation···π interactions. This latter is rearomatizated to **4xx**, by losing the β-proton and recovering the electroneutrality of the capsule **C**. The mechanism proposed in Scheme 1 is corroborated by the finding that α-ketoester **3f**, bearing an electron-withdrawing trifluoromethyl moiety in α-position to ketone group, in the presence of **C** and **2a** or **2b** gives the mono-alkylated adduct **6af** and **6bf** in almost quantitative yields, while no evidence of di-adduct was detected. Probably, under these conditions, the presence of the electron-withdrawing trifluoromethyl group disfavours the formation of carbocation **IV**, which would have a positive charge on the carbon atom directly bonded to the electron-withdrawing trifluoromethyl group.

On the basis of these results and in order to extend the scope of the reaction between **2** and carbonyl compounds in the presence of **C**, we studied the procedure with a different carbonyl compound such as benzaldehyde **10a** (Table 4). When the substrates **2a** and **10a** were mixed in 1/1 ratio in the presence of **C** in water-saturated CDCl_3_ then α,α-dipyrromethane **11a** was obtained in 34% yield with a regioselectivity ratio of 8.5/1 (entry 1, Table 4) with respect to the α,β-isomer **12a**. Interestingly, when the **2a/10a** molar ratio was progressively increased to 2/1 and to 4/1 then the efficiency of the reaction was improved with a 54 and 87% yield of **11a**, respectively (entries 2 and 3, Table 4). Interestingly, no hint of product **11a** and **12a** were detected in the reaction mixture in the absence of capsule **C**. The lowering of the reaction temperature from 50 to 25°C (entry 4 in Table 4) gives rise to a drop in the yield of **11a**. Once the reaction conditions were optimized (Table 4), the substrate scope was then evaluated in order to determine the generality of the reaction.

**Table 4:** Optimization of reaction conditions for the reaction between **2a** and **10a**.

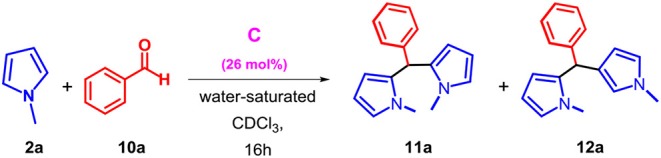						
**Entry^a^**	**Capsule**	**T (°C)**	**2a/10a**	**Yield (%)^b^**	**11a (%)^c^**	**12a (%)^c^**
1	No	50°C	1/1	—	—	—
	Yes			38	34	4^d^
2	No	50°C	2/1	—	—	—
	Yes			60	54	6
3	No	50°C	4/1	—	—	—
	Yes			97	87	10
4	No	25°C	4/1	—	—	—
	Yes			20	18	2^d^

a *Reactions were performed on a 0.16 mmol scale using **2a** (from 1 to 4 equiv.), **3a** (1 equiv.), and capsule **C** (0.26 equiv.) in water saturated CDCl_3_ (1.1 mL) under stirring for 16 h*.

b *Overall yield of all the isolated products*.

c *Yields of the isolated products by chromatography on column*.

d *The column gave an inseparable mixture with regioisomer and the yield was calculated by integration of the respective ^1^H-NMR signals of the regioisomers in the isolated fraction*.

As regards the effect of the substitution at the pyrrole nitrogen atom, we found that the introduction of a more hindering group, such as a phenyl or benzyl group, caused a complete loss of reactivity (entries 2–3, Table 5). Instead, the reaction with unsubstituted pyrrole **2b** proceeded with a small decrease in yield but preserving the selectivity for adduct **11a** (entry 1, Table 5). Interestingly, under the conditions reported in Table 5 no hint of mono-adduct heteroaryl methane was observed. Successively, we investigated the generality of the reaction between **2a** and several aromatic aldehydes bearing electron-donating or -withdrawing groups (Table 5).

**Table 5:** Scope of the reaction with different pyrroles **2a–d** and aldehydes **10a–j**.

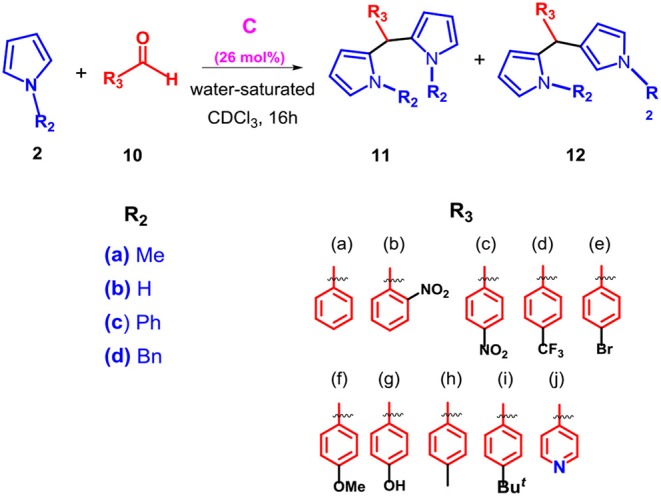						
**Entry^a^**	**Capsule**	**2**	**10**	**Yield(%)^b^**	**% (11)^c^**	**% (12)^c^**
**1**	No	**2b**	**10a**	—	—	—
	Yes			70	70 (**11ba**)^e^	—
**2**	No	**2c**	**10a**	—	—	—
	Yes			—	—	—
**3**	No	**2d**	**10a**	—	—	—
	Yes			**—**	—	—
**4**	No	**2a**	**10b**	—	—	—
	Yes			99	90 (**11ab**)	9 (**12ab**)
**5**	No	**2a**	**10c**	—	—	—
	Yes			98	96 (**11ac**)	2 (**12ac**)^d^
**6**	No	**2a**	**10d**	—	—	—
	Yes			98	88 (**11ad**)	10 (**12ad**)
**7**	No	**2a**	**10e**	—	—	—
	Yes			95	93 (**11ae**)	2 (**11ae**)^d^
**8^f^**	No	**2a**	**10f**	—	—	—
	Yes			98	96 (**11af**)	2 (**12af**)^d^
**9**^f^	No	**2a**	**10g**	—	—	—
	Yes			98	96 (**11ag**)	2 (**12ag**)^d^
**10**	No	**2a**	**10h**	—	—	—
	Yes			97	95 (**11ah**)	2 (**12ah**)^d^
**11**	No	**2a**	**10i**	—	—	6 (**12ai**)
	Yes			97	91 (**11ai**)	
**12**	No	**2a**	**10j**	—	—	—
	Yes			85	76 (**11aj**)^d^	9 (**12aj**)^d^

a *Reactions were performed on a 0.16 mmol scale using **2a–d** (4 equiv.), **10a–j** (1 equiv.), and capsule **C** (0.26 equiv.) in water saturated CDCl_3_ (1.1 mL) under stirring for 16 h at 50°C*.

b *Overall yield of all the isolated products*.

c *Yields of the isolated products by chromatography on column*.

d *The column gave an inseparable mixture with the regioisomer and the yield was calculated by integration of the respective ^1^H-NMR signals of the regioisomers in the isolated fraction*.

e *^1^H NMR spectrum on crude reaction mixture showed presence of other species obtained after chromathography purification as a complex and inseparable fraction not characterized*.

f *These reactions were performed under stirring for 48 h at 50°C*.

The protocol was found to be tolerant to a variety of aromatic aldehydes **10a–j**, independently by the electronic nature and position of the substituents on the aryl group, affording α,α-adducts **11** in high yields and excellent regioselectivities. In fact, the double attack took place in a completely regioselective way to give **11** as almost the only product with a negligible amount of the corresponding isomer **12**. No evidence of monoalkylated adduct was observed. Additionally, when the protocol was extended to the *N*-methyl indole **8e**, the reaction proceeded smoothly and the adduct **13** was obtained as the only product in high yield (Table 6).

**Table 6:** Scope of the reaction between indole **8e** and various aldehydes **10a, b, d, e, j**.

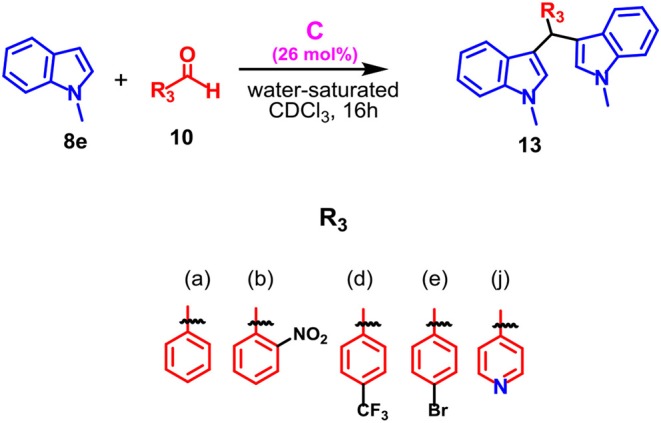			
**Entry^a^**	**Capsule**		**Yield (%)^b^**
**1**	No	**10a**	—
	Yes		97 **(11ea)**
**2**	No	**10b**	—
	Yes		99 (**11eb**)
**3**	No	**10d**	—
	Yes		98 (**11ed**)
**4**	No	**10e**	—
	Yes		98 (**11ee**)
**5**	No	(**10j**)	—
	Yes		98 (**11ej)**

a *Reactions were performed on a 0.16 mmol scale using **8e** (4 equiv.), **10** (1 equiv.), and capsule **C** (0.26 equiv.) in water saturated CDCl_3_ (1.1 mL) under stirring for 16 h at 50°C*.

b *Isolated yield*.

## Conclusions

The resorcinarene hexameric capsule **C** is able to catalyze the reaction between pyrroles or indoles and α-ketoesters or aldehydes for the formation of bis(heteroaryl)methanes. The reactions take place in the nanoconfined space inside the capsule **C**. The observed results suggested its double catalytic function: **C** can act as H-bond catalyst for the initial activation of the carbonyl functions and as a Brønsted acid catalyst for the dehydration of the intermediate alcohol. Generally, in the presence of **C** the formation of the α,α-bis(heteroaryl)methanes occurs with excellent yields and regioselectivity with respect to the α,β- or β,β-regioisomers.

## Data Availability Statement

All experimental data are reported in the Supplementary Material.

## Author Contributions

SG and PL performed the experiments. CT performed NMR studies. CG, AS, and PN participated in manuscript preparation. CT and MD prepared the manuscript.

### Conflict of Interest

The authors declare that the research was conducted in the absence of any commercial or financial relationships that could be construed as a potential conflict of interest.
