# Supplementary material for: The Hexameric Resorcinarene Capsule as a Brønsted Acid Catalyst for the Synthesis of Bis(heteroaryl)methanes in a Nanoconfined Space
Source: Front Chem. 2019 Oct 22;7:687. doi: 10.3389/fchem.2019.00687 (PMC6817573; doi:10.3389/fchem.2019.00687)
Supplement: Supplementary file 1 [file Data_Sheet_1.pdf]

Supporting Information

The Hexameric Resorcinarene Capsule as a Brønsted Acid Catalyst for the Synthesis of Bis(heteroaryl)methanes in a Nanoconfined Space

Stefania Gambaro, Pellegrino La Manna, Margherita De Rosa*, Annunziata Soriente, Carmen Talotta*, Carmine Gaeta and Placido Neri

Laboratory of Supramolecular Chemistry, Dipartimento di Chimica e Biologia "A. Zambelli", Università degli Studi di Salerno, via Giovanni Paolo II 132, I-84084 Fisciano, Salerno, Italy

maderosa@unisa.it; ctalotta@unisa.it

Table of Contents

1. General Remarks

2. Procedures

2.1 Reaction Monitoring

2.2 General Procedures for Alkylation of Heteroarenes by Carbonyl Compounds Inside the Resorcinarene Self-Assembled Hexameric Capsule

3.Inhibition Experiments

3.1 Inhibition Experiments with Tetraethylammonium Tetrafluoroborate

3.2 Inhibition Experiments with DMSO

4. Encapsulation Experiments

4.1 Encapsulation Experiments of Ethyl Pyruvate inside the hexameric capsule

4.2 Encapsulation Experiments of Ethyl Pyruvate inside the hexameric capsule

5.Spectral data of new compounds

6.NMR and Mass spectra of new compounds

1. General Remarks

All chemicals were reagent grade and were used without further purification. Solvents were purchased from Aldrich. Reaction temperatures were measured externally; reactions were monitored by ^{1}H NMR. Flash chromatography was performed on Merck silica gel (60, 40-63 μm). NMR spectra were recorded on Reaction temperatures were measured externally; reactions were monitored by ^{1}H NMR and by TLC on Merck silica gel plates (0.25 mm) and visualized by UV light. Flash chromatography was performed on Merck silica gel (60, 40-63 μm). NMR spectra were recorded on Bruker Avance-600 spectrometer [600.13 MHz (^{1}H) and 150.03 MHz (^{13}C)], Bruker Avance-400 spectrometer [400 (^{1}H) and 100.57 MHz (^{13}C)], Bruker Avance-300 spectrometer [300 (^{1}H) and 75.48 MHz (^{13}C)]; chemical shifts are reported relative to the residual solvent peak ($CHCl_3$: δ 7.26, $CDCl_3$: δ 77.23). DOSY experiments were performed on a Bruker Avance-600 spectrometer equipped with 5 mm PABBO BB|19F-1H\D Z-GRD Z114607/0109. The standard Bruker pulse program, ledbpgp2s, employing a double stimulated echo sequence and LED, bipolar gradient pulses for diffusion, and two spoil gradients were utilized. Diffusion times were 150 ms, eddy current delay was 5 ms, gradient recovery delays was 0.2 ms and gradient pulse was 1400 ms. Individual rows of the quasi-2D diffusion databases were phased and baseline corrected. NOESY experiments were performed on Bruker Avance 400 and Bruker Avance 600 spectrometers with a D8 value range from 0.05 to 0.75 s. High-resolution mass spectra (HRMS) were acquired using a Bruker Solaris XR Fourier transform ion cyclotron resonance mass spectrometer equipped with a 7 T refrigerated actively-shielded superconducting magnet. The samples were ionized in positive ion mode using the ESI ion source (Bruker Daltonik GmbH, Bremen, Germany). The mass spectra were calibrated externally using a NaTFA solution in positive ion mode. Low resolution mass spectral analyses were carried out using an electrospray spectrometer Waters 4 micro quadrupole. A linear calibration was applied. All final compounds purity was determined by elemental analysis on a Flash EA 1112 Series with Thermal Conductivity Detector, for C, H, N, and S. The final compounds purity was found to be >95% when analyzed. Water saturated deuterated chloroform was prepared as reported in the literature[1]. Resorcinarene[2] (**1**), *N*-benzylpirrole[3] (), *N*-phenylpyrrole[4] () and Benzyl-2-oxo-propanoate[5] () were synthesized according to literature procedures.

2. Procedures

[1] La Sorella, G., Sperni, L., Strukul, G., and Scarso, A. (2016). Supramolecular Activation of Hydrogen Peroxide in the Selective Sulfoxidation of Thioethers by a Self-Assembled Hexameric Capsule. *Adv. Synth. Catal*. 358, 3443-3449. doi: 10.1002/adsc.201600430.

[2] Tunstad, L. M., Tucker, J. A., Dalcanale, E., Weiser, J., Bryant, J. A., Sherman, J. C., Helgeson, R. C., Knobler, C. B., and Cram, D. J. (1989). Host-Guest Complexation. 48. Octol Building Blocks for Cavitands and Carcerands. *J. Org. Chem*. 54, 1305-1312. doi: 10.1021/jo00267a015.

[3] Scaggs, W. R., Scaggs, T. D., and Snaddon, T. N. (2019). An enantioselective synthesis of α-alkylated pyrroles via cooperative isothiourea/palladium catalysis. *Org. Biomol. Chem*. 17, 1787-1790. doi: 10.1039/C8OB02600A.

[4] Hong, X., Tan, Q., Liu, B., and Xu, B. (2017). Isocyanide-Induced Activation of Copper Sulfate: Direct Access to Functionalized Heteroarene Sulfonic Esters. *Angew. Chem. Int. Ed*. 56, 3961-3965. doi: 10.1002/anie.201612565.

[5] Kijima, M., Miyamori, K., and Sato, T. (1988). Highly selective photoinduced dimerization of alkyl pyruvates catalyzed by cobaloxime *J. Org. Chem*. 53, 1719-1722. doi: 10.1021/jo00243a022.

2.1 Reaction Monitoring

40 µL of the reaction solution in the vial was taken, diluted with 360 µL of $CDCl_3$ and the reaction progress was monitored by ^{1}H NMR.

2.2 General Procedures for Alkylation of Heteroarenes by Carbonyl Compounds Inside the Resorcinarene Self-Assembled Hexameric Capsule

Typical procedure in presence of ***(1)₆• 8H₂O*** **(C).**

Resorcinarene **1** (281.6 mg, 254.7 µmol, 1.56 eq.) was weighed in a 4 ml vial. Then, 1.1 mL of water saturated deuterated chloroform was added and the mixture was warmed at 50°C until clarification (ca 5 min). The aromatic heterocycle (650.4 µmol, 4 eq.) was added and the solution was stirred at 50°C for 5 minutes. Then, the carbonyl compound (162.6 µmol, 1.0 eq) was added and the reaction mixture was vigorously stirred (1400 rpm) for the appropriate time. Then, the reaction mixture was poured into a 50 mL Eppendorf conical tube and diluted with 35 mL of a solution of DMSO in hexane (0.13% (*v*/*v*)). The mixture was cooled to -20°C until massive precipitation of resorcinarene **1** was observed. Next, the precipitated resorcinarene was removed by centrifugation (1750 rpm for 10 min). The diluted reaction mixture was subjected three times to this procedure. Finally, the clear solution was removed and concentrated under reduced pressure. The oily residue thus obtained was purified by flash chromatography on silica gel (Hexane/Diethyl Ether, from 99/1 to 80/20) to afford the desired title compounds.

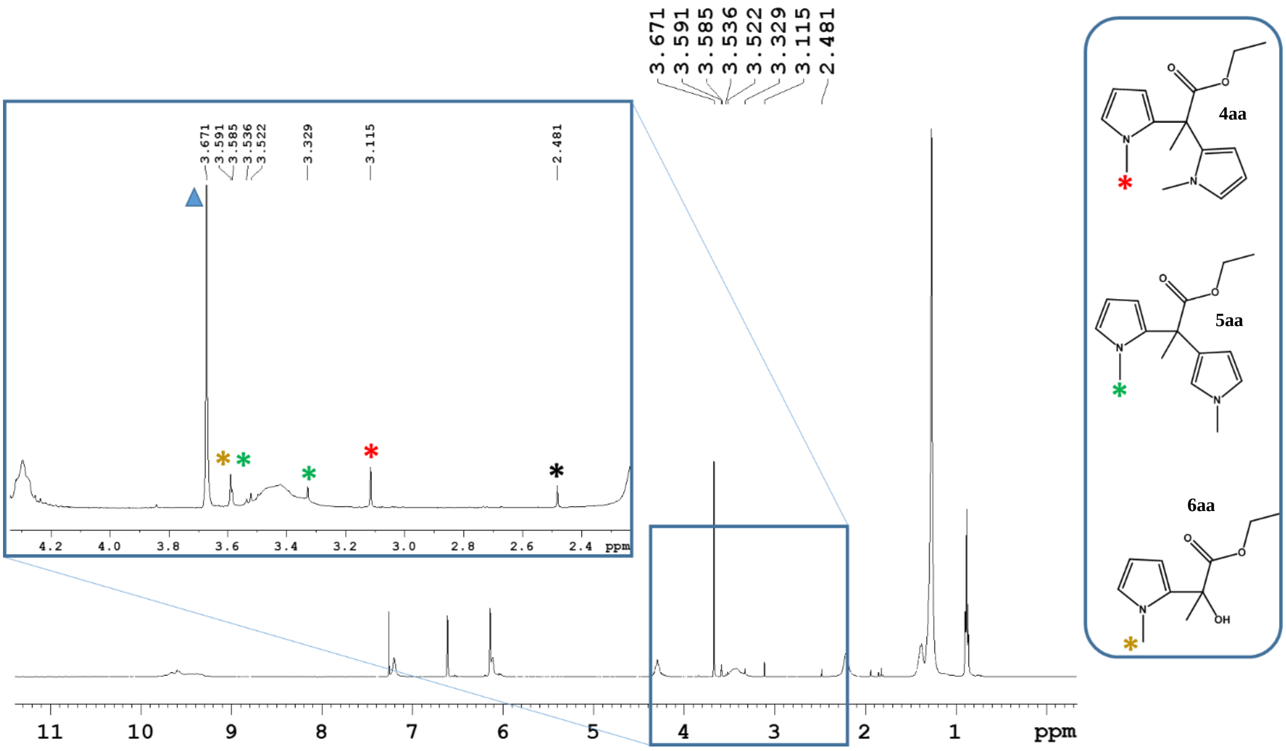

Figure S1. ^{1}H NMR spectrum (400 MHz, 298 K, $CDCl_3$) of the reaction mixture between *N*-methylpyrrole (**2a**) and ethylpyruvate (**3a**) in presence of the capsule **1_6• $8H_2O$** after 8h at 30°C. Black asterisk is indicative of the presence of ethylpyruvate (**3a**).

Typical procedure in absence of ***(1)₆• 8H₂O (C)***.

The aromatic heterocycle (650.4 μmol, 4 eq) and the appropriate carbonyl compound (162.6 μmol, 1 eq) were mixed in a 4 ml vial, then 1.1 mL of water saturated deuterated chloroform was added. The reaction mixture was vigorously stirred (1400 rpm) at 30°C for 48h, then dried in vacuum and subjected to flash chromatography on silica gel.

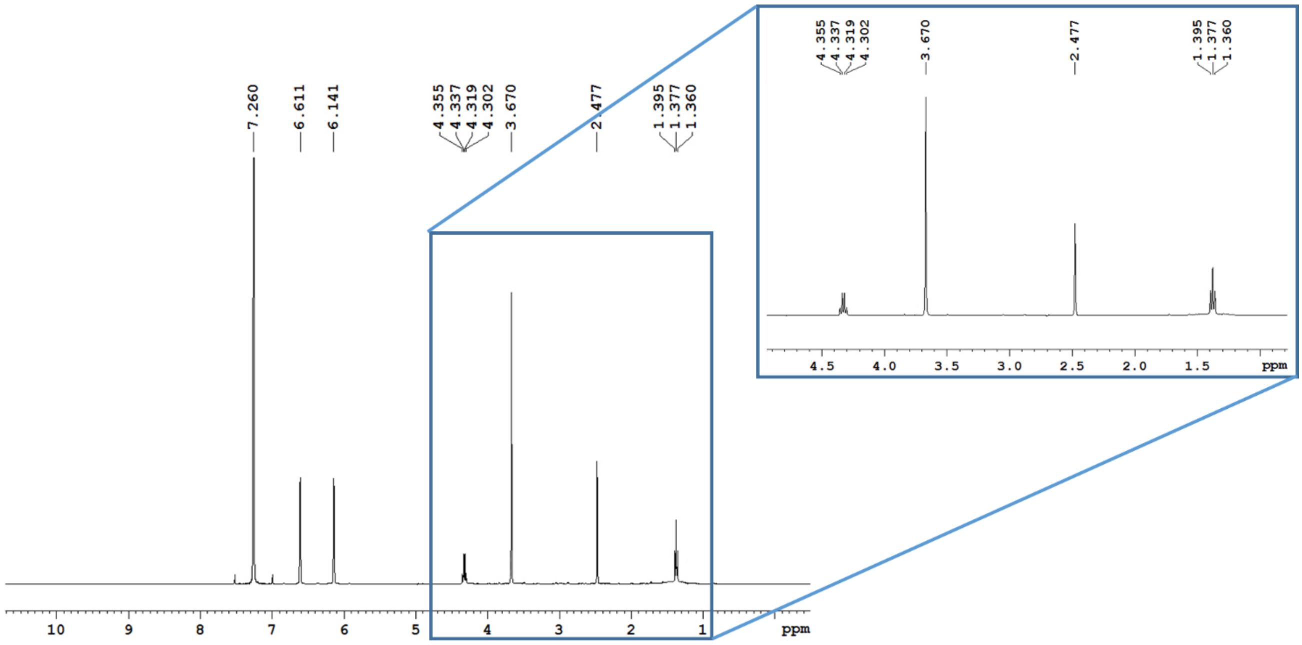

Figure S2. ^{1}H NMR spectrum (400 MHz, 298 K, $CDCl_3$) of the reaction mixture between *N*-methylpyrrole (**2a**) and ethylpyruvate (**3a**) without capsule **1_6• $8H_2O$** after 48h at 30°C. As can be seen in the inset in the Figure, no trace of product is present.

3.Inhibition Experiments

3.1 Inhibition Experiments with Tetraethylammonium Tetrafluoroborate

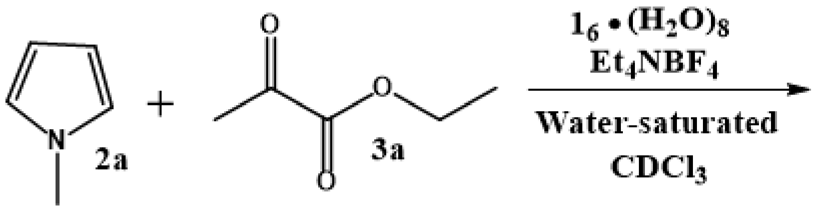

The inhibition experiment was conducted mixing **2a** (592 mM, 4 eq) and **3a** (148 mM, 1 eq) under the conditions described at page S4 in presence of tetraethylammonium tetrafluoroborate as inhibitor[6]. The ^{1}H NMR spectrum of the crude reaction mixture in Figure S3 shows NMR signals at negative values of chemical shifts, indicative of the presence of tetraethylammonium guest inside the cavity of **1_6• $(H_2O)_8$**. As can be seen in Figure S3 (see also Figure S1) no signal attributable to

[6] La Sorella, G., Sperni, L., Strukul, G., Scarso, A. (2015). Supramolecular Encapsulation of Neutral Diazoacetate Esters and Catalyzed 1,3-Dipolar Cycloaddition Reaction by a Self-Assembled Hexameric Capsule. *ChemCatChem*, 7, 291-296. doi: 10.1002/cctc.201402631.

the reaction products are detected. Also, signals of encapsulation of ammonium salt are evidenced.

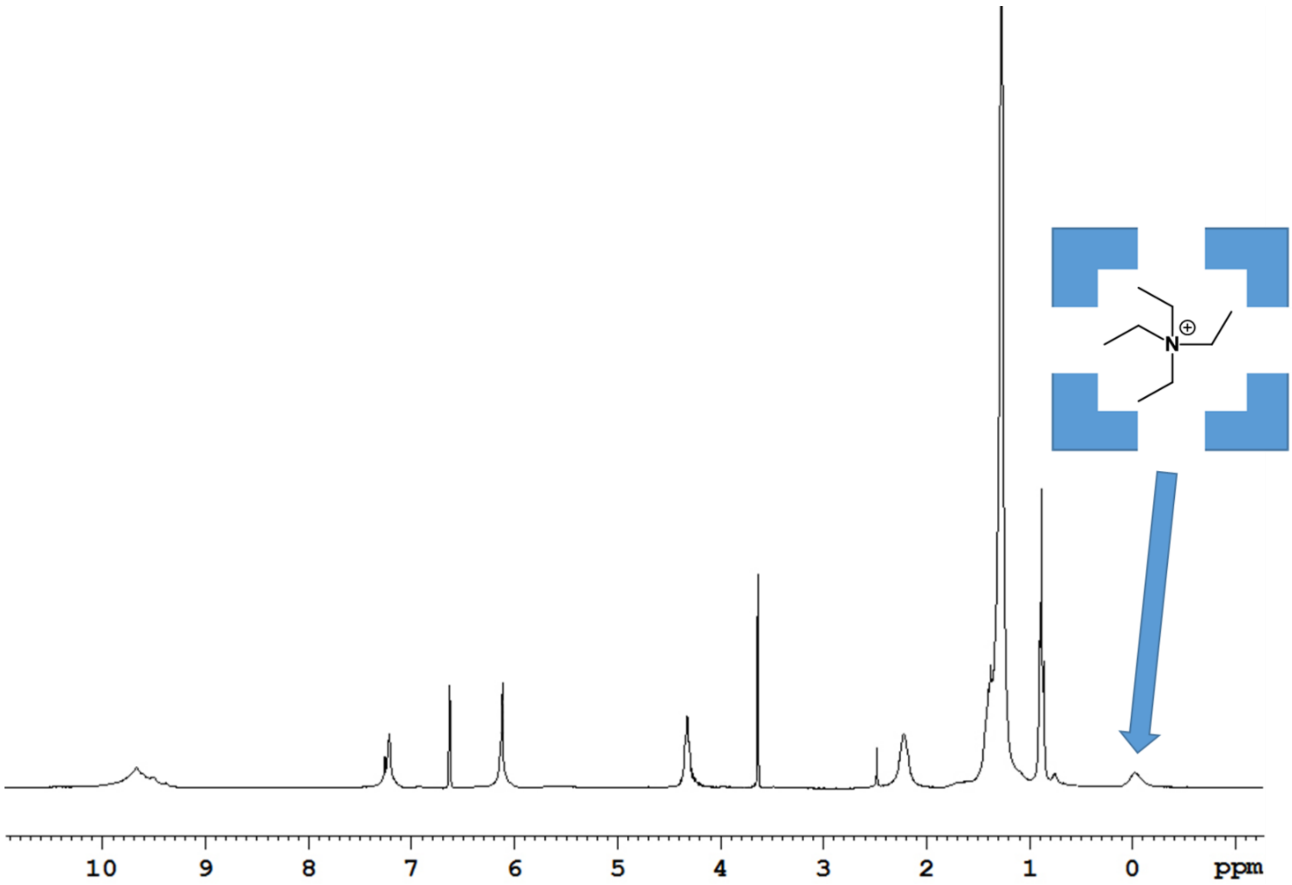

Figure S3. Reaction between **2a** (592 mM) and **3a** (148 mM) in the presence of **1_6• $(H_2O)_8$** (38.6 mM) and NEt_4BF_4 (386 mM, 10 eq) as inhibitor. ^{1}H NMR spectrum (400 MHz, $CDCl_3$, 298 K) of the crude reaction mixture after 16h at 50°C.

3.2 Inhibition Experiments with DMSO

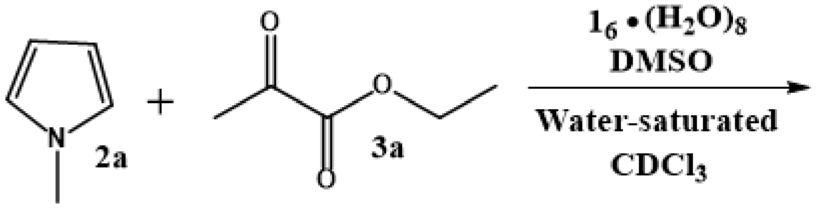

According to the ability of DMSO to make hydrogen bonding and so disrupt the hexameric structure of the capsule[7], inhibition experiments in presence of DMSO (60 μL, 770 mM) and capsule **1_6 • $(H_2O)_8$** (38.6 mM) were performed. If catalysis of the reaction occurs inside the nanocontainer, the reaction with the addition of DMSO should not proceed. In fact, with adding DMSO no signals attributable to product are visible (see Figure S4).

[7] Avram, L., Cohen, Y. (2002). Spontaneous Formation of Hexameric Resorcinarene Capsule in Chloroform Solution as Detected by Diffusion NMR. *J. Am. Chem. Soc*. 124, 15148-15149. doi: 10.1021/ja0272686.

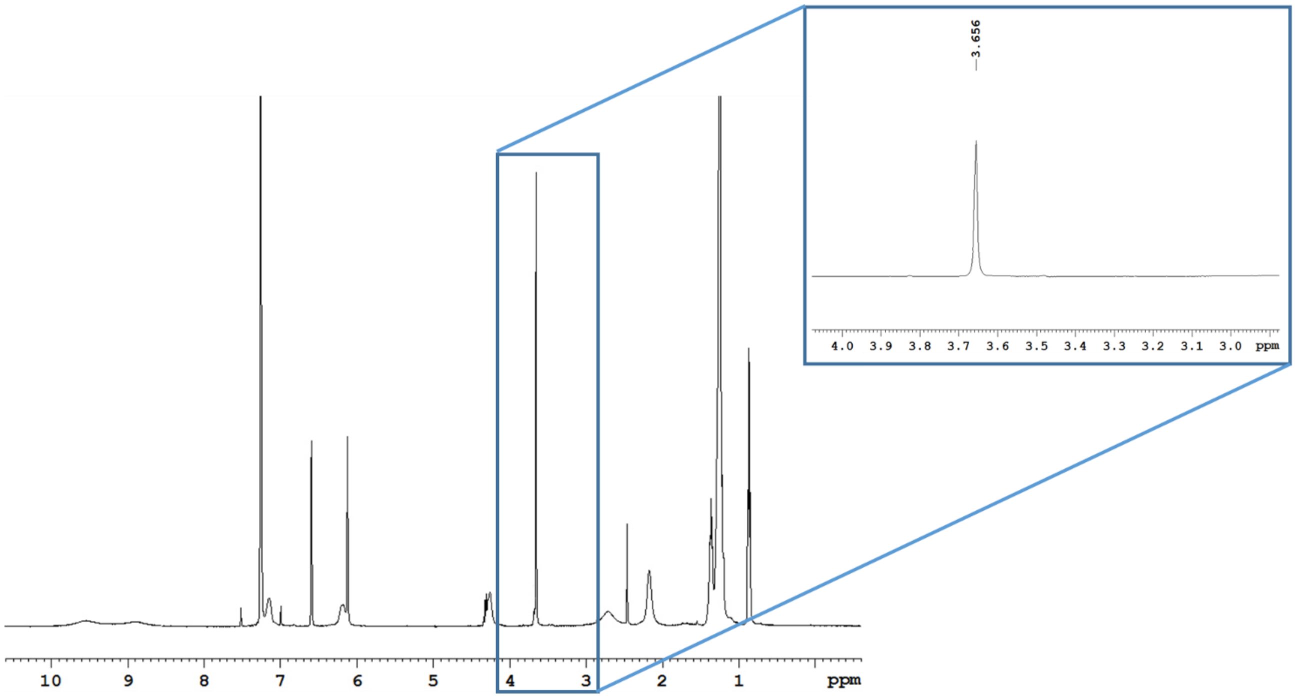

Figure S4. Reaction between **2a** (592 mM) and **3a** (148 mM) in the presence of $\mathbf{1_6\bullet (H_2O)_8}$ (38.6 mM) and DMSO (770 mM, 20 eq) as inhibitor. ^{1}H NMR spectrum (400 MHz, $CDCl_3$, 298 K) of the crude reaction mixture after 16h at 50°C. As evidenced, no signals attributable to the reaction products are detected.

4. Encapsulation Experiments

4.1 *Encapsulation Experiments of Ethyl Pyruvate* ***3a*** *inside the hexameric capsule* $\boldsymbol{1_6 \bullet (H_2O)_8}$

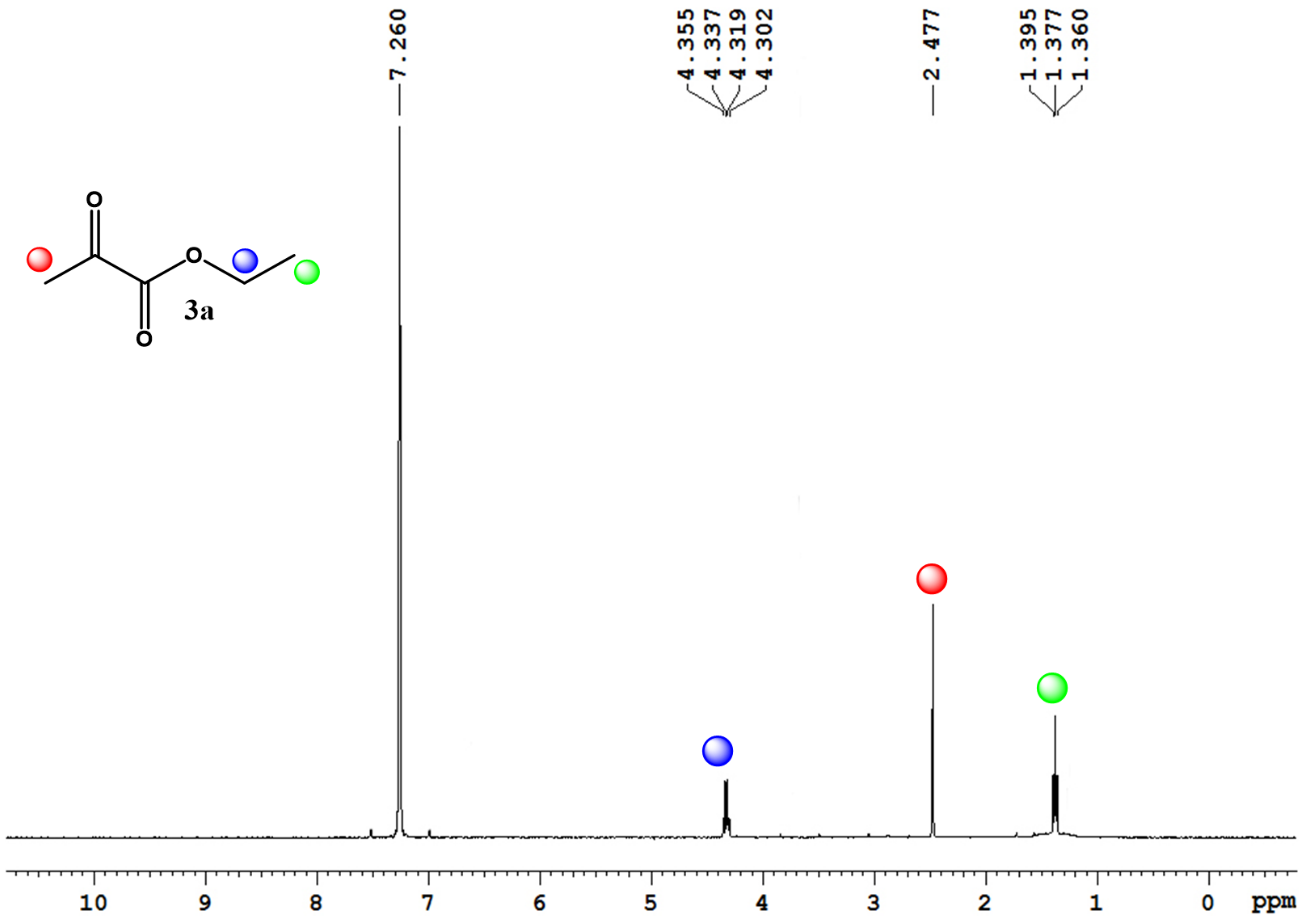

Figure S5. ^{1}H NMR spectrum (400 MHz, $CDCl_3$, 298 K) of **3a**.

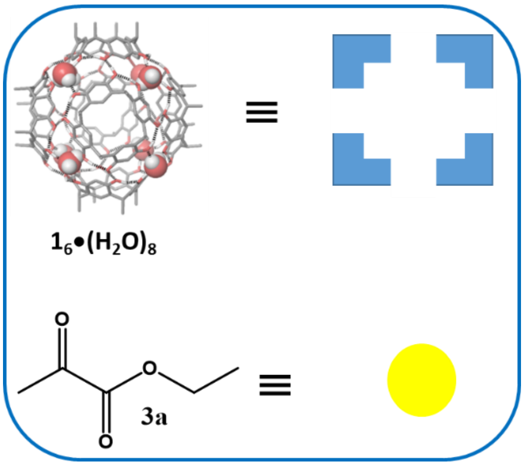

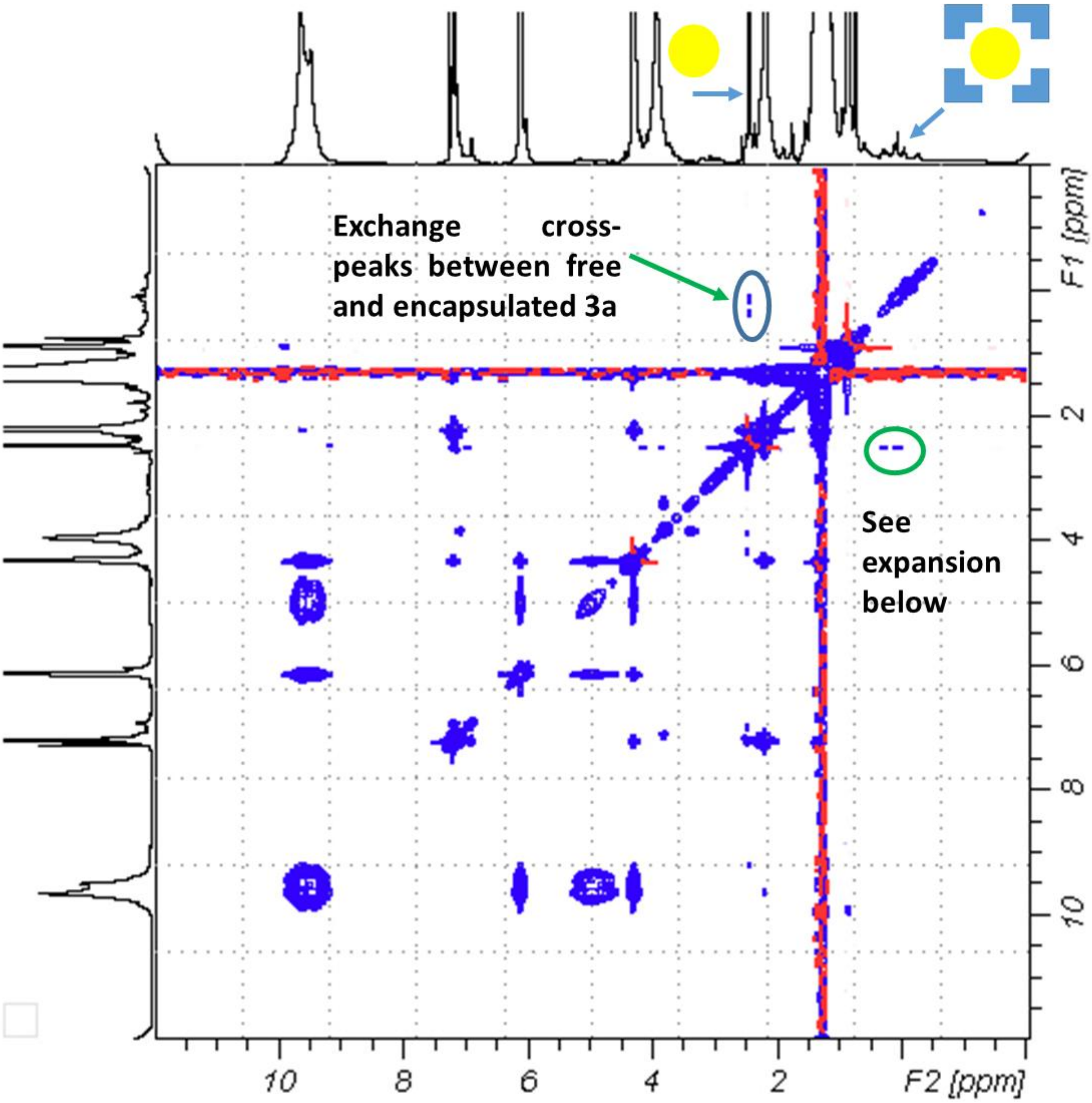

Figure S6. 2D EXSY experiment (400 MHz, $CDCl_3$, mixing time 400 ms) of the mixture of **3a** and **1_6•$(H_2O)_8$**.

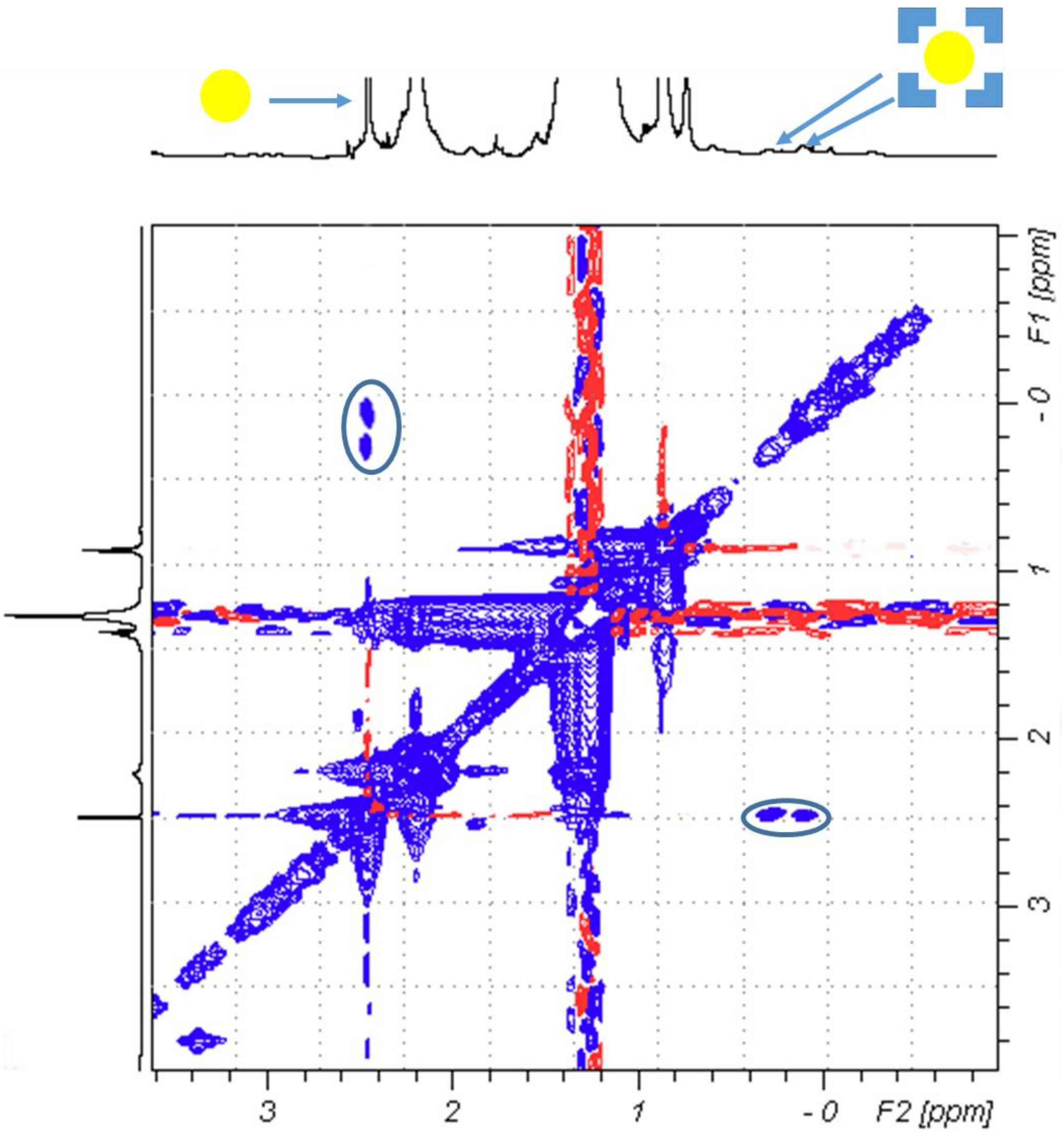

Figure S7. Relevant region of the 2D EXSY experiment in Figure S6. Exchange cross-peaks between free and encapsulated **3a** are evidenced.

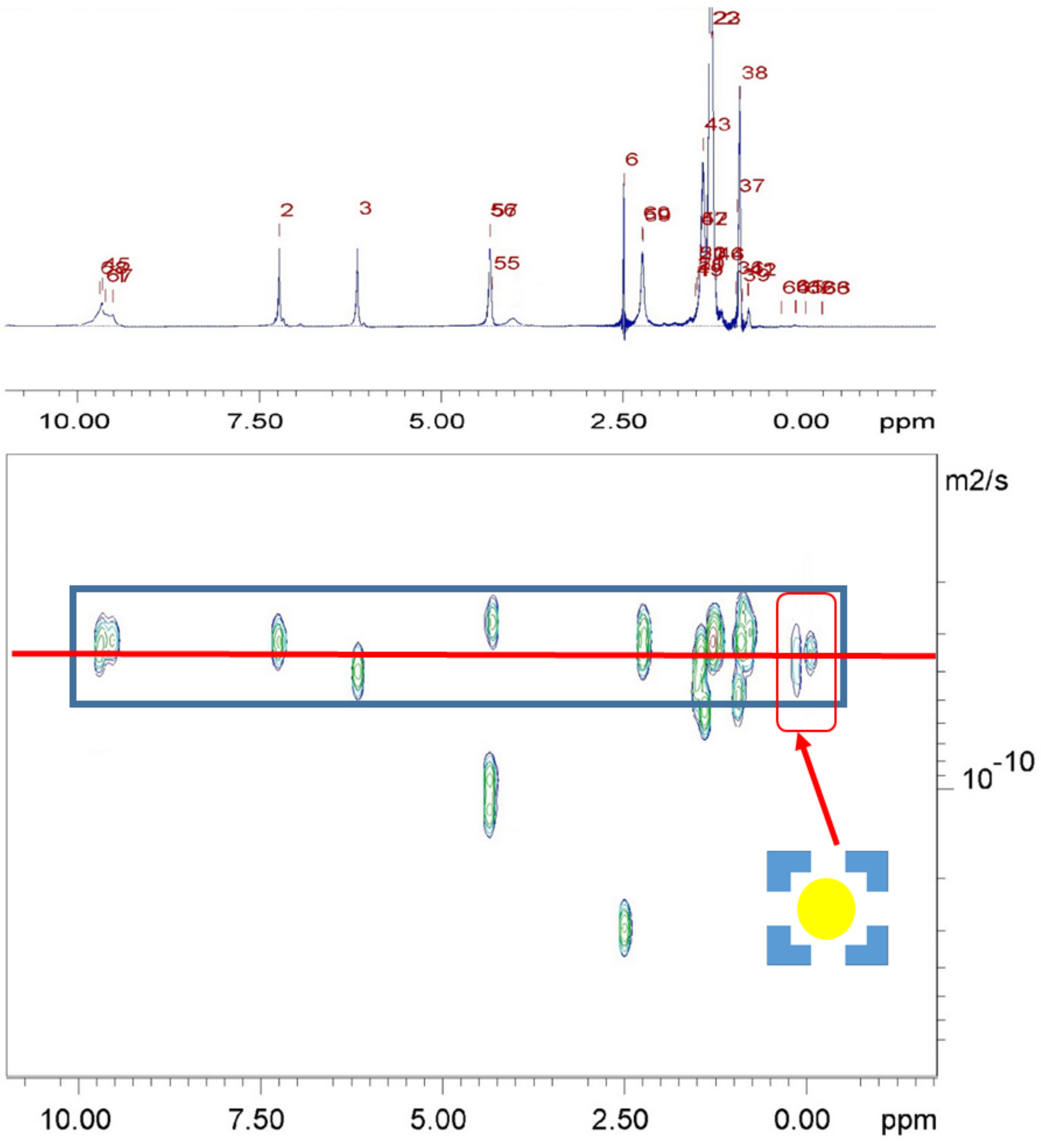

Figure S8. The pattern associated to the pyruvate **3a** inside the hexameric capsule $\mathbf{1_6\bullet(H_2O)_8}$ is aligned with capsule diffusion coefficient.

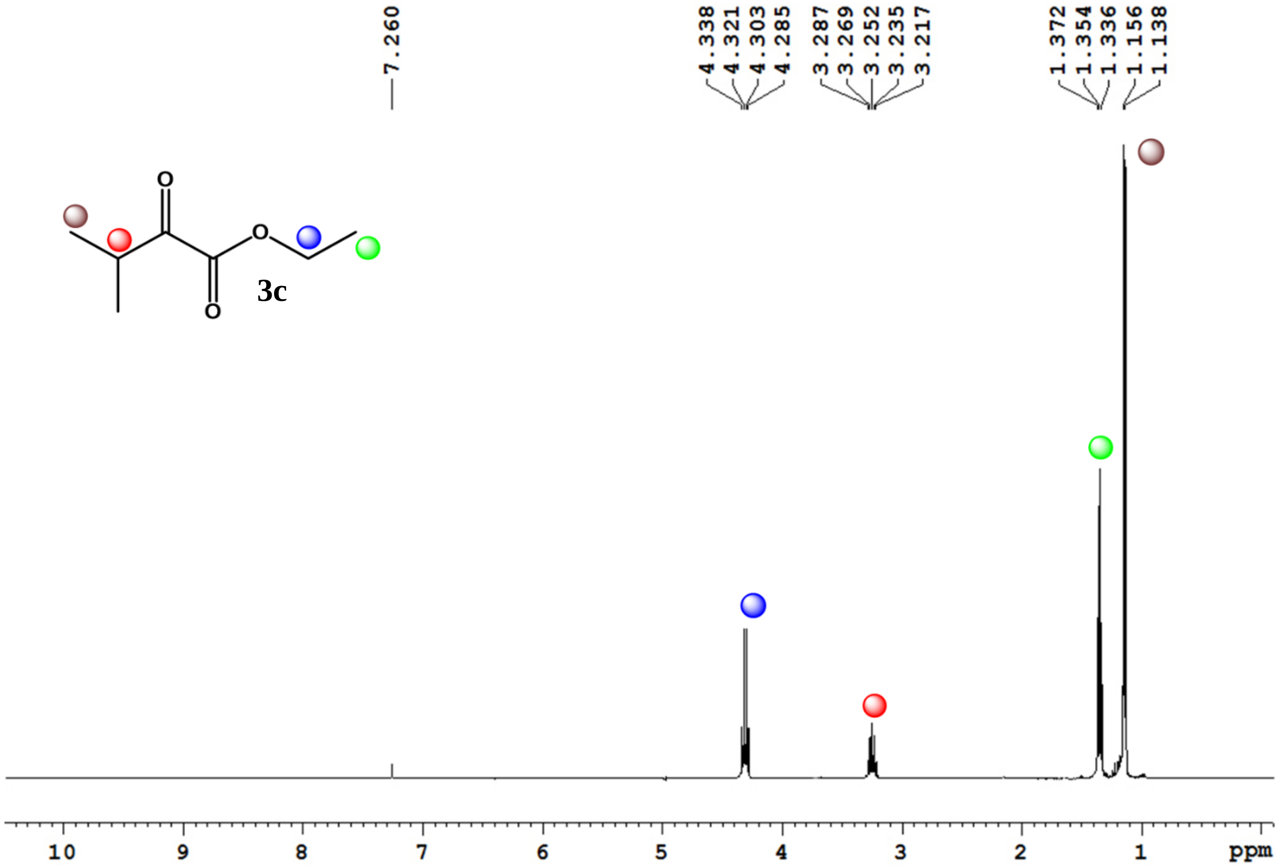

Figure S9. ^{1}H NMR spectrum (400 MHz, $CDCl_3$, 298 K) of **3c**.

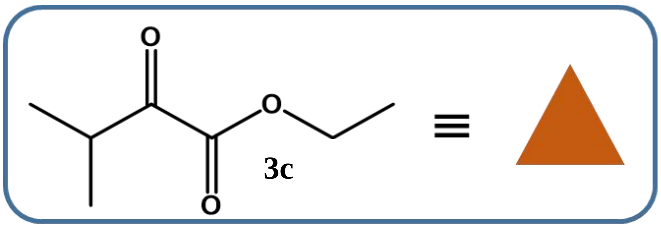

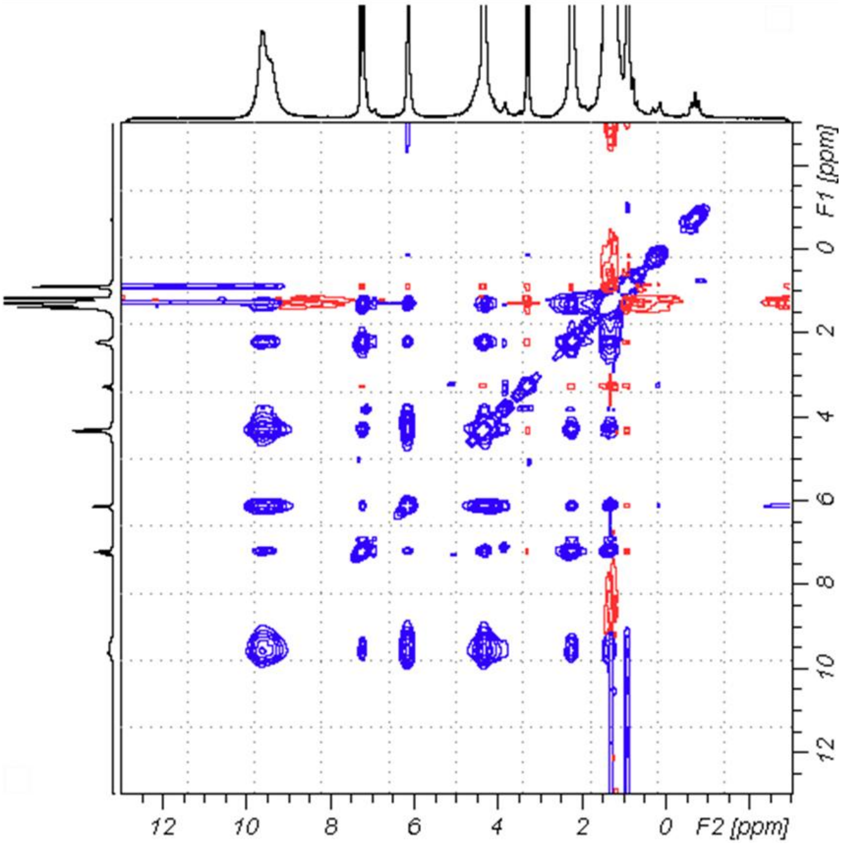

Figure S10. 2D EXSY experiment (400 MHz, $CDCl_3$, mixing time 600 ms) of the mixture of **3c** and **1_6•$(H_2O)_8$**.

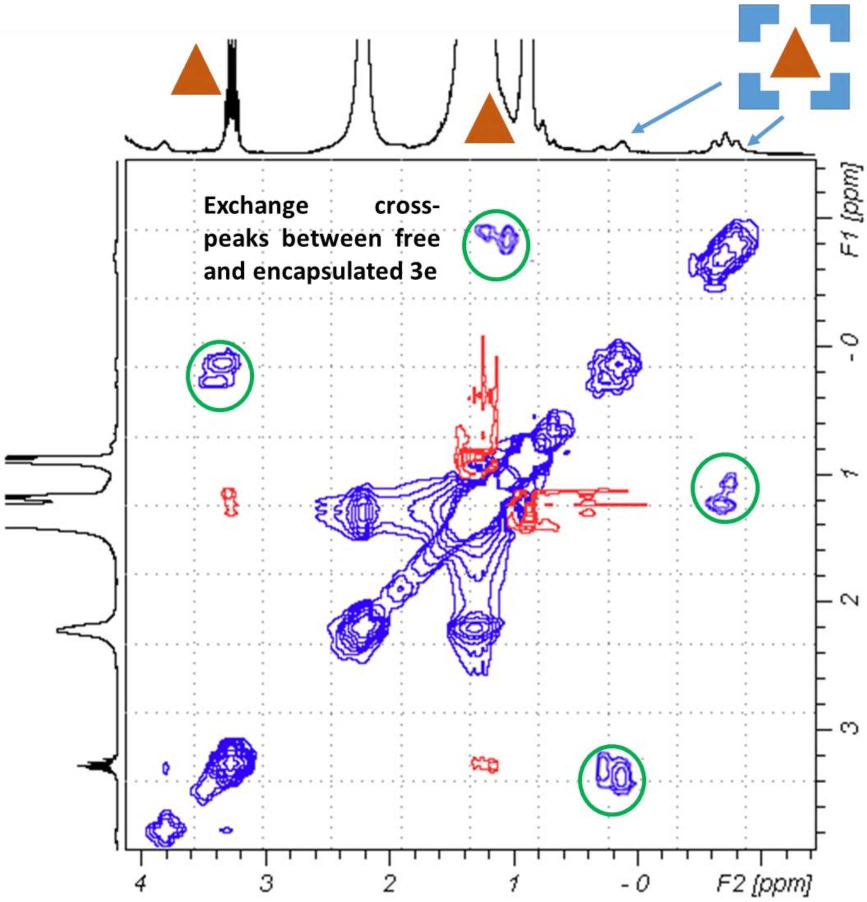

Figure S11 Relevant region of the 2D EXSY experiment in Figure S10. Exchange cross-peaks between free and encapsulated **3c** are evidenced.

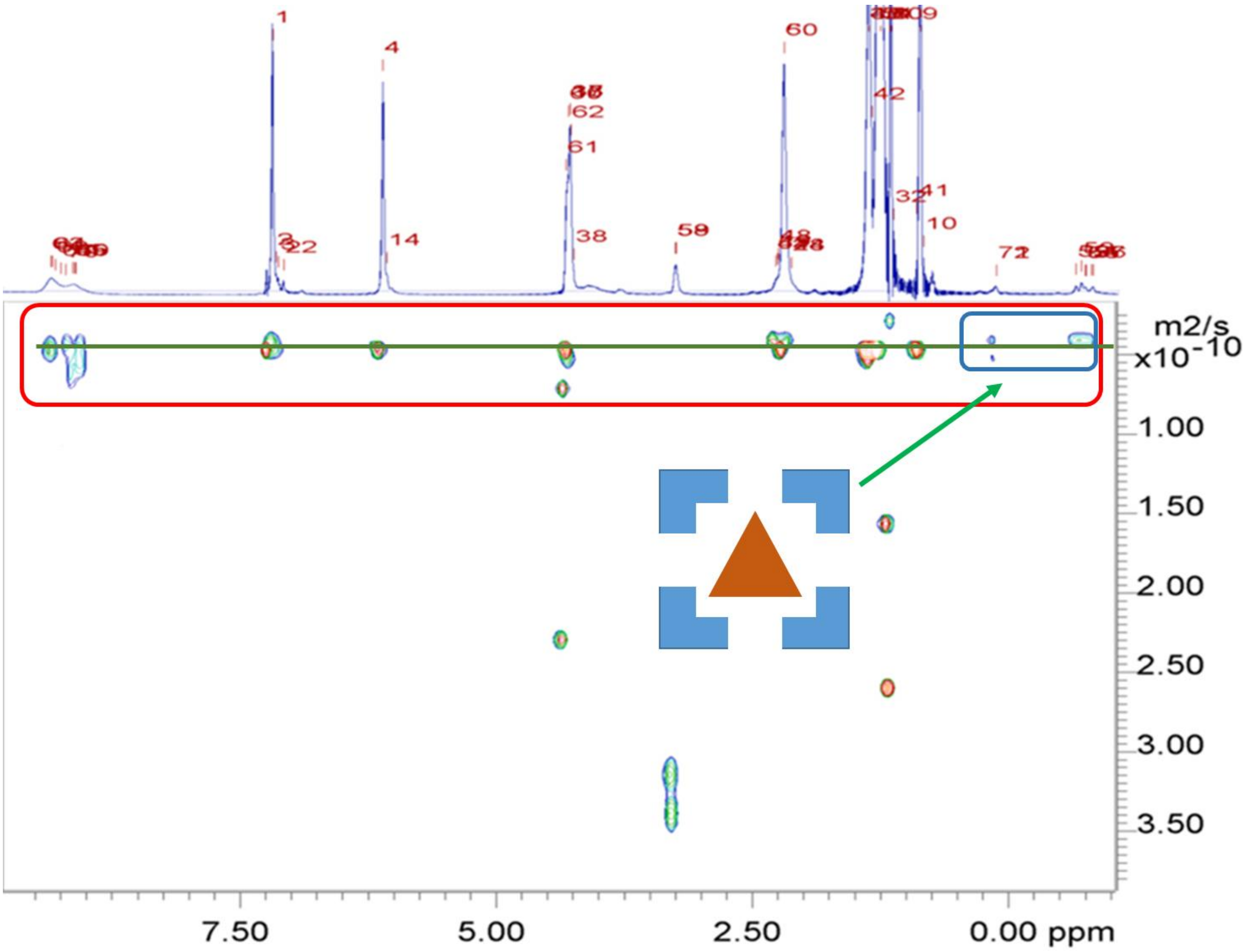

Figure S12. The pattern associated to the pyruvate **3c** inside the hexameric capsule $\mathbf{1_6{\bullet}(H_2O)_8}$ is aligned with capsule diffusion coefficient.

5. Spectral data of new compounds

ethyl 2-(1-methyl-1H-pyrrol-2-yl)-2-(1-methyl-1H-pyrrol-3-yl)propanoate (5aa)

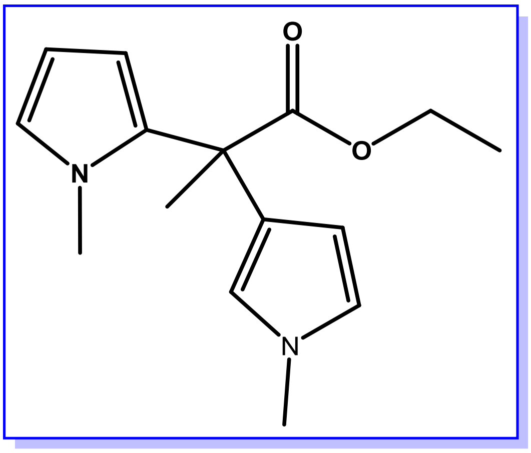

Obtained as a clear yellow oil, following general procedure described above (pag S4) using *N*-methylpyrrole (**2a**) as nucleophile and ethylpyruvate (**3a**) as electrophile. Purification of the residue was carried out by column chromatography on silica flash (Hexane/Et_2O, 8/2) to give **5aa**. ^{1}H NMR (600 MHz, $CDCl_3$, 298 K): δ 1.26 (t, *J* = 7.2 Hz, 3H, -OCH_2*CH_3*), 1.86 (s, 3H, CH_3), 3.33 (s, 3H, NCH_3), 3.59 (s, 3H, NCH_3), 4.19-4.25 (m, 2H, -O*CH_2*CH_3), 5.98-6.07 (m, 3H, ArH), 6.42-6.56 (m, 3H, ArH). ^{13}C NMR (150 MHz, $CDCl_3$, 298 K): δ 14.4, 28.5, 35.3, 36.4, 46.9, 61.3, 106.1, 107.4, 108.4, 120.0, 121.4, 123.3, 126.8, 136.0, 175.4. MALDI (m/z): calcd for $C_{15}H_{20}N_2O_2$ $[M+H]^+$: 261.17540, found: 261.17527.

ethyl 2-hydroxy-2-(1-methyl-1H-pyrrol-2-yl)propanoate (6aa)

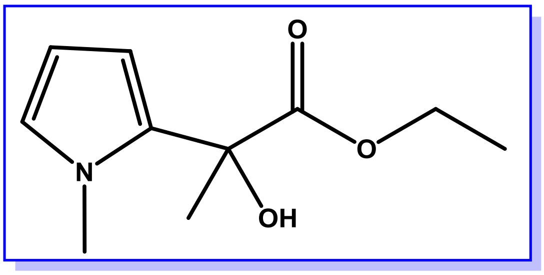

Obtained as a clear oil, following general procedure described above (pag. S4) using *N*-methylpyrrole (**2a**) as nucleophile and ethylpyruvate (**3a**) as electrophile. Purification of the residue was carried out by column chromatography on silica flash (Hexane/Et_2O, 8/2) to give **6aa**. ^{1}H NMR (600 MHz, $CDCl_3$, 298 K): δ 1.26 (t, *J* = 7.2 Hz, 3H, -OCH_2*CH_3*), 1.82 (s, 3H, -*CH_3*), 3.52 (s, 1H, -*OH*), 3.59 (s, 3H, -N*CH_3*), 4.20-4.33 (m, 2H,-O*CH_2*CH_3), 6.03-6.04 (m, 2H, ArH), 6.19-6.20 (m, 2H, ArH), 6.56-6.57 (m, 2H, ArH). ^{13}C NMR (150 MHz, $CDCl_3$, 298 K): δ 14.3, 26.9, 35.2, 62.7, 72.2, 106.5, 108.6, 124.7, 131.9, 176.3. MALDI (m/z): calcd for $C_{10}H_{15}NO_3$ $[M+H]^+$: 198.11247, found: 198.11242 $[M+H]^+$.

ethyl 2,2-di(1H-pyrrol-2-yl)propanoate (4ba)

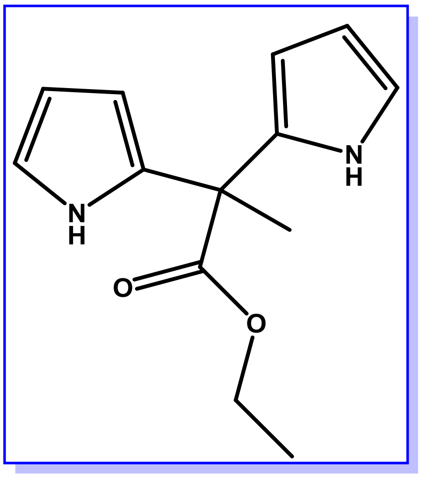

Obtained as a clear oil, following general procedure described above (pag. S4) using pyrrole (**2b**) as nucleophile and ethylpyruvate (**3a**) as electrophile. Purification of the residue was carried out by column chromatography on silica flash (Hexane/Et_2O, 8/2) to give **4ba**. ^{1}H NMR (600 MHz, $CDCl_3$, 298 K): δ 1.29 (t, J = 7.2 Hz, 3H, -OCH_2*CH_3*), 1.91 (s, 3H, CH_3), 4.24 (q, *J* = 7.2 Hz, 2H, -OCH_2CH_3), 6.07-6.08 (m, 2H, ArH), 6.14-6.16 (m, 2H, ArH), 6.70-6.71 (m, 2H, ArH), 8.46 (s, 2H, NH). ^{13}C NMR (150 MHz, $CDCl_3$, 298 K): δ 14.3, 25.1, 47.2, 62.1, 106.1, 108.4, 117.8, 132.8, 173.9. MALDI (m/z): calcd for $C_{13}H_{16}N_2O_2$ $[M+H]^+$: 233.12845, found: 233.12867.

1,1-bis(1-methyl-1H-pyrrol-2-yl)ethan-1-ol (4ae)

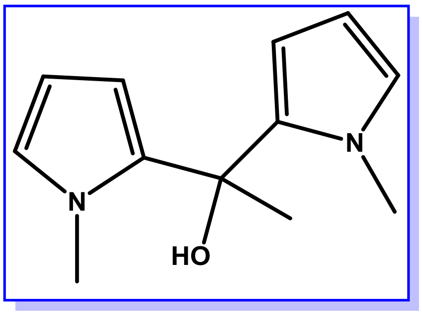

Obtained as a light brown oil, following general procedure described above (pag. S4) using *N*-methylpyrrole (**2a**) as nucleophile and pyruvic acid (**3e**) as electrophile. Purification of the residue was carried out by column chromatography on silica flash (Hexane/Et_2O, 8/2) to give **4ae**. ^{1}H NMR (600 MHz, $CDCl_3$, 298 K): δ 1.59 (d, *J* = 7.2 Hz, 3H, CH_3), 3.40 (s, 6H, NCH_3), 4.08 (q, *J* = 7.2 Hz, 1H, OH), 5.84-5.85 (m, 2H, ArH), 6.03-6.04 (m, 2H, ArH), 6.51 (s, 2H, ArH). ^{13}C NMR (150 MHz, $CDCl_3$, 298 K): δ 20.8, 30.2, 33.9, 106.3, 106.6, 121.9, 135.6. MALDI (m/z): calcd for $C_{12}H_{16}N_2O$ $[M+H]^+$: 205.13354, found: 205.13337.

ethyl 2-hydroxy-3-methyl-2-(1-methyl-1H-pyrrol-2-yl)butanoate (6ac)

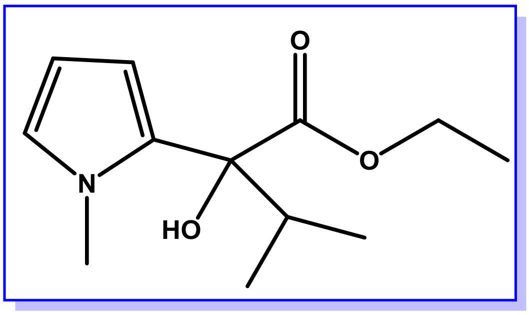

Obtained as a yellow oil, following general procedure described above (pag S4) using *N*-methylpyrrole (**2a**) as nucleophile and 3-methyl-2-oxobutyrrate (**3c**) as electrophile. Purification of the residue was carried out by column chromatography on silica flash (Hexane/Et_2O, 8/2) to give **6ac**. ^1H NMR (600 MHz, $CDCl_3$, 298 K): δ 0.89 (d, *J* = 5.9 Hz, 3H, CH(CH_3)$_2$), 1.06 (d, *J* = 6.7 Hz, 3H, CH(CH_3)$_2$), 1.26 (t, *J* = 7.2 Hz, 3H, -OCH$_2$$CH_3$), 2.62 (sept, *J* = 6.8 Hz, 1H, *CH*(CH_3)$_2$), 3.59 (s, 1H, OH), 3,66 (s, 3H, NCH_3), 4.15-4.32 (m, 2H, -O$CH_2$$CH_3$), 6.04 (dd, *J* = 2.7 Hz, 3.7 Hz, 1H, ArH), 6.18 (dd, J = 1.8 Hz, 3.7 Hz, 1H ArH), 6.50-6.51 (m, 1H, ArH). ^{13}C NMR (150 MHz, $CDCl_3$, 298 K): δ 14.1, 17.0, 17.1, 34.4, 36.1, 62.4, 78.4, 106.3, 109.4, 124.4, 130.4, 175.3. MALDI (m/z): calcd for $C_{12}H_{19}NO_3$ $[M+H]^+$: 226.14377, found: 226.14400.

ethyl 2-(1-benzyl-1H-pyrrol-2-yl)-2-hydroxypropanoate (6da)

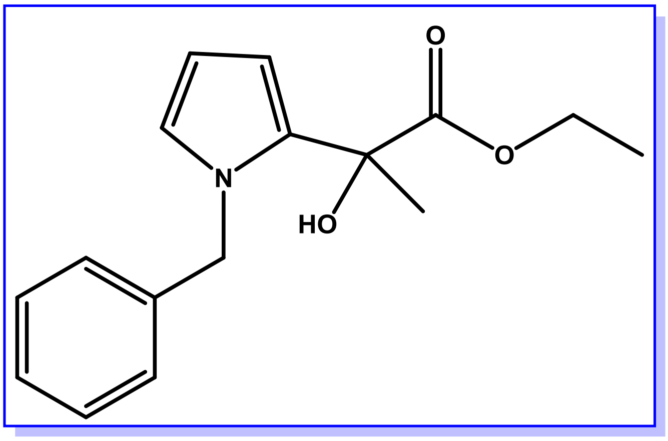

Obtained as a colourless oil, following general procedure described above (pag. S4) using *N*-benzylpyrrole (**2d**) as nucleophile and ethylpyruvate (**3a**) as electrophile. Purification of the residue was carried out by column chromatography on silica flash (Hexane/Et_2O, 8/2) to give **6da**. ^1H NMR (600 MHz, $CDCl_3$, 298 K): δ 1.11 (t, *J* = 7.1 Hz, 3H, -OCH$_2$$CH_3$), 1.81 (s, 3H, CH_3), 3.54 (s, 1H, OH), 3.58 (dq, *J* = 7.1 Hz, 10.7 Hz, 1H, -O$CH_2$$CH_3$), 3.97 (dq, *J* = 7.1 Hz, 10.6 Hz, 1H, -O$CH_2$$CH_3$), 5.20 (ABq, *J* = 16.3 Hz, 2H, NCH_2-Ar), 6.14 (dd, *J* = 2.9 Hz, 3.6 Hz, 1H, Ar$H_{pyrrole}$), 6.28 (dd, *J* = 1.8 Hz, 3.7 Hz, 1H, Ar$H_{pyrrole}$), 6.62 (dd, *J* = 2.6 Hz, 2.7 Hz, 1H, Ar$H_{pyrrole}$), 6.99 (d, *J* = 7.3 Hz, 2H, ArH), 7.21-7.23

(m, 2H, ArH), 7.27-7.29 (m, 1H, ArH). ^{13}C NMR (150 MHz, $CDCl_3$, 298 K): δ 14.1, 27.5, 51.1, 62.5, 72.2, 107.3, 108.9, 124.5, 126.8, 127.4, 128.6, 132.1, 138.7, 176.1. MALDI (m/z): calcd for $C_{16}H_{19}NO_3$ $[M+Na]^+$: 296.12571, found: 296. 12571.

ethyl 2,2-bis(1-phenyl-1H-pyrrol-3-yl)propanoate (**7ca**)

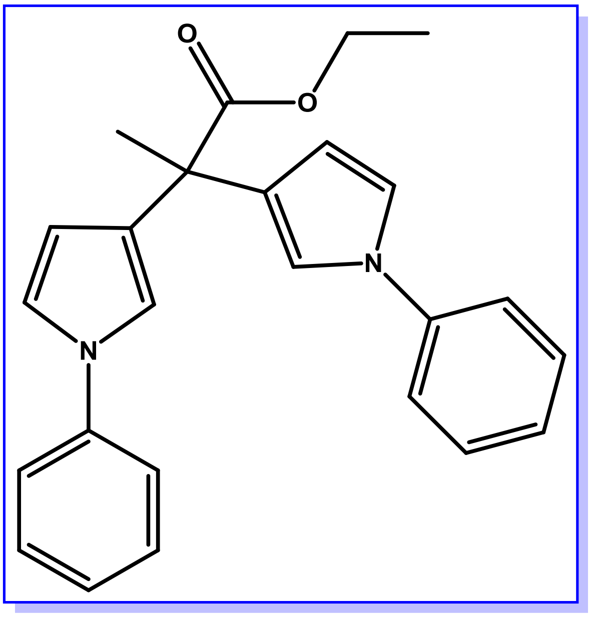

Obtained as a colourless oil, following general procedure described (pag. S4) using *N*-phenylpyrrole (**2c**) as nucleophile and ethylpyruvate (**3a**) as electrophile. Purification of the residue was carried out by column chromatography on silica flash (Hexane/Et_2O, 8/2) to give **7ca**. ^1H NMR (600 MHz, $CDCl_3$, 298 K): δ 1.29 (t, J = 7.2 Hz, 3H, $-OCH_2CH_3$), 1.93 (s, 3H, CH_3), 4.23 (q, J = 7.2 Hz, 2H, $-OCH_2CH_3$), 6.37 (dd, J = 1.8 Hz, 2.9 Hz, 2H, $ArH_{pyrrole}$), 7.00-7.01 (m, 2H, $ArH_{pyrrole}$), 7.04-7.05 (m, 2H, $ArH_{pyrrole}$), 7.19-7.22 (m, 2H, ArH), 7.36-7.40 (m, 8H, ArH). ^{13}C NMR (150 MHz, $CDCl_3$, 298 K): δ 14.4, 26.9, 46.3, 61.2, 110.7, 117.3, 119.0, 120.3, 125.5, 129.7, 130.7, 140.9, 175.8. MALDI (m/z): calcd for $C_{25}H_{24}N_2O_2$ $[M+Na]^+$: 407.17300, found: 407.17279.

dimethyl 2,2'-(1-methyl-1H-pyrrole-2,4-diyl)bis(2-hydroxypropanoate)

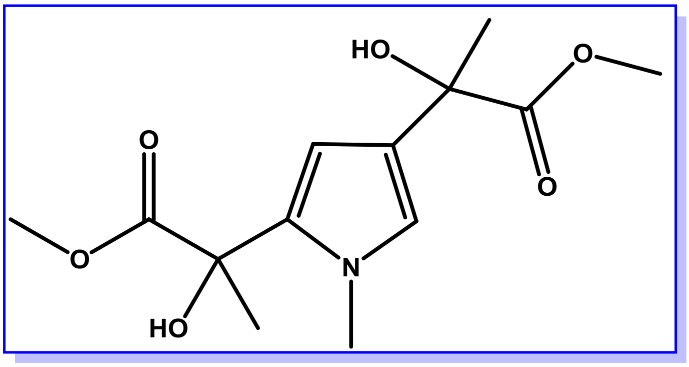

Obtained as a colourless oil from the reaction between *N*-methylpyrrole (**2a**) as nucleophile and methylpyruvate (**3b**) as electrophile under the conditions reported at pag. S4. Purification of the residue was carried out by column chromatography on silica flash (Hexane/Et_2O, 8/2) to give the title compound. ^1H NMR (600 MHz, $CDCl_3$, 298 K): δ 1.72 (s, 3H, CH_3), 1.74 (s, 3H, CH_3), 3.77-3.81 (overlapped, 11H, NCH_3, OCH_3, OH), 6.11 (s, 1H, ArH), 6.16 (s, 1H, ArH). ^{13}C NMR (150 MHz, $CDCl_3$, 298 K): δ 23.3, 23.4, 53.5, 58.5, 58.6, 82.4, 82.7, 116.7, 116.8, 147.8, 166.4, 169.7, 172.4. MALDI (m/z): calcd for $C_{13}H_{19}NO_6$ $[M]^+$: 285.12069, found: 285.12072.

benzyl 2,2-di(1H-indol-3-yl)propanoate **(9ad)**

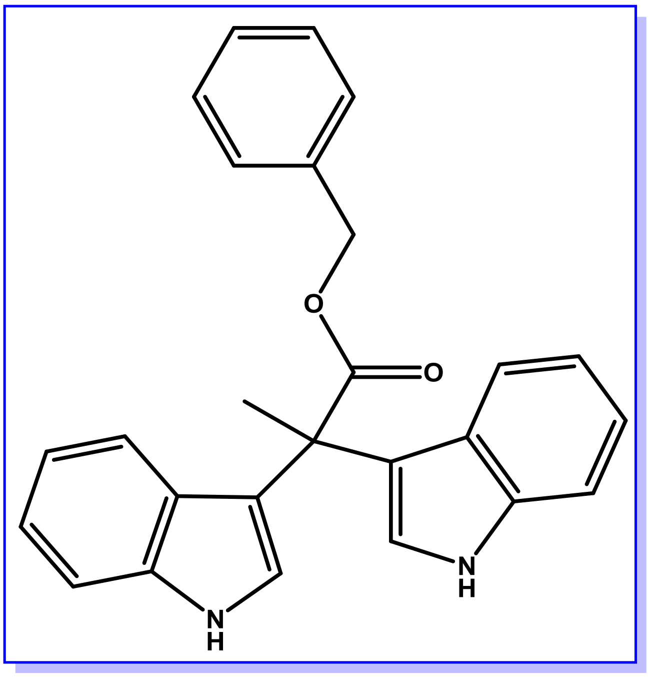

Obtained as a yellow solid (m.p: 153.2-154.1 °C)., following general procedure described above (pag. S4) for the reaction between indole (**8a**) and benzylpyruvate (**3d**). Purification of the residue was carried out by column chromatography on silica flash (Hexane/Et_2O, 8/2) to give **9ad**. ^1H NMR (600 MHz, $CDCl_3$, 298 K): δ 2.15 (s, 3H, CH_3), 5.15 (s, 2H, CH_2), 6.91-6.95 (overlapped, 4H, ArH_{indole}), 7.10 (d, J = 6.6 Hz, 2H, ArH), 7.14 (t, J = 7.8 Hz, 2H, ArH_{indole}), 7.18-7.23 (overlapped, 3H, ArH), 7.33 (d, J = 8.1 Hz, 2H, ArH_{indole}), 7.44, (d, J = 8.1 Hz, 2H, ArH_{indole}), 8.00 (s, 2H, NH). ^{13}C NMR (150 MHz, $CDCl_3$, 298 K): δ 26.0, 46.7, 67.0, 111.4, 119.2, 119.4, 121.6, 121.9, 123.1, 126.2, 128.0, 128.3, 128.4, 136.1, 136.9, 175.3. MALDI (m/z): calcd for $C_{26}H_{22}N_2O_2$ $[M + Na]^+$: 417.15735, found: 417.15705.

***benzyl 2,2-bis(1-methyl-1H-pyrrol-2-yl)propanoate* (4ad)**

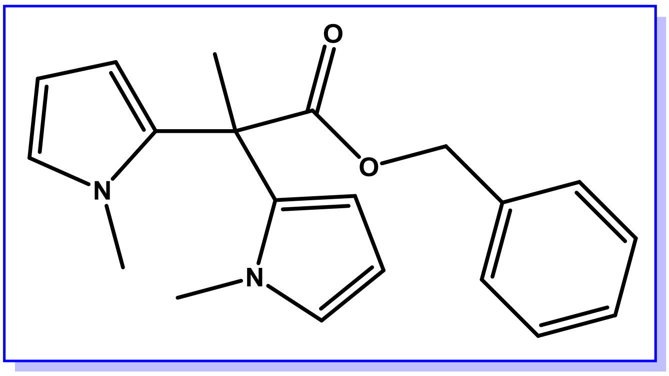

Obtained as a colourless oil, following general procedure described above (pag S4) using *N*-methylpyrrole (**2a**) as nucleophile and benzylpyruvate (**3d**) as electrophile. Purification of the residue was carried out by column chromatography on silica flash (Hexane/Et_2O, 8/2) to give **4ad**. ^{1}H NMR (600 MHz, $CDCl_3$, 298 K): δ 1.89 (s, 3H, CH_3), 3.00 (s, 6H, $N(CH_3)$)), 5.14 (s, 2H, -CH_2), 5.92 (dd, J_1 = 1.9 Hz, J_2 = 3.7 Hz, 2H, ArH_{pyrrole}), 5.95 (dd, J_1 = 2.8 Hz, J_2 = 3.7 Hz, 2H, Ar$H_{pyrrole}$), 6.44 (t, J = 2.2 Hz, 2H, Ar$H_{pyrrole}$), 7.18-7.25 (overlapped, 5H, ArH). ^{13}C NMR (150 MHz, $CDCl_3$, 298 K): δ 25.8, 34.8, 48.3, 67.3, 106.6, 108.2, 124.2, 128.2, 128.3, 128.7, 132.5, 135.9, 172.8. MALDI (m/z): calcd for $C_{20}H_{22}N_2O_2$ $[M + H]^+$: 323.16890, found: 323.16889.

***2,2'-((4-(tert-butyl)phenyl)methylene)bis(1-methyl-1H-pyrrole)* (11ai)**

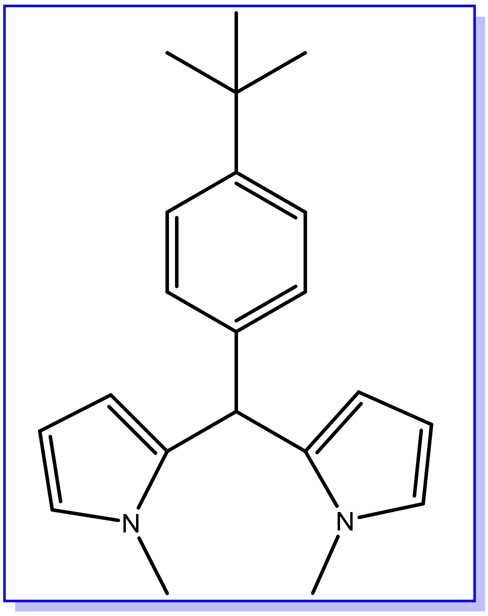

Obtained as brown solid (mp 95-97 °C), following general procedure described above (pag S4) using *N*-methylpyrrole (**2a**) as nucleophile and *p*-butylbenzaldehyde(**10i**) as electrophile.

Purification of the residue was carried out by column chromatography on silica flash (Hexane/EtOAc, 8/2) to give **11ai.** ^{1}H NMR (600 MHz, $CDCl_3$, 298 K): δ 1.32 (s, 9H, CH_3), 3.40 (s, 6H, $N(CH_3)$), 5.25 (bs, 1H, CH), 5.53-5.23 (m, 2H, ArH_{Pyr}), 6.03 (t, J = 3.2 Hz, 2H, ArH_{Pyr}), 6.58 (t, J = 2.2 Hz, 2H, ArH_{Pyr}), 7.07 (d, J = 8.2 Hz, 2H, ArH), 7.31 (d, J = 8.2 Hz, 2H, ArH). ^{13}C NMR (150 MHz, $CDCl_3$, 298 K): δ 31.6, 34.1, 34.6, 41.7, 106.6, 109.0, 122.0, 125.4, 128.5, 134.0, 138.3, 149.6. ESI m/z calcd for $C_{21}H_{26}N_2$ $[M]^+$: 306.20905, found: 306.20450.

2,2'-((4-(trifluoromethyl)phenyl)methylene)bis(1-methyl-1H-pyrrole) (11ad)

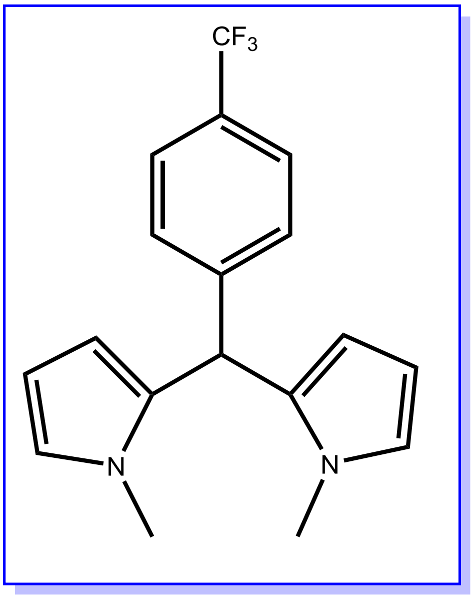

Obtained as brown solid (mp 103.4-105.5°C), following general procedure described above (pag. S4) using *N*-methylpyrrole (**2a**) as nucleophile and *p-(*trifluoromethyl)benzaldehyde (**10d**) as electrophile. Purification of the residue was carried out by column chromatography on silica flash (Hexane/EtOAc, 8/2) to give **11ad.** ^{1}H NMR (400 MHz, $CDCl_3$, 298 K): δ 3.42 (s, 6H, $N(CH_3)$), 5.36 (s, 1H, CH), 5.51 (bs, 2H, ArH_{Pyr}), 6.06 (t, J = 3.1 Hz, 2H, ArH_{Pyr}), 6.63 (bs, 2H, ArH_{Pyr}), 7.29 (d, J = 7.6 Hz, 2H, ArH), 7.69 (d, J = 7.6Hz, 2H, ArH). ^{13}C NMR (100 MHz, $CDCl_3$, 298 K): δ 34.0, 41.9, 106.7, 109.3, 122.4, 124.4 (q, $^1J_{CF}$ = 272.2 Hz), 125.5 (q, $^3J_{CF}$ = 3.8 Hz), 129.3 (q, $^2J_{CF}$ = 32.1 Hz), 129.2, 132.6, 154.1. MALDI (m/z) calcd for $C_{18}H_{17}F_3N_2$ $[M+H]^+$: 319.14166, found: 319.14491.

4-(bis(1-methyl-1H-pyrrol-2-yl)methyl)pyridine (11aj)

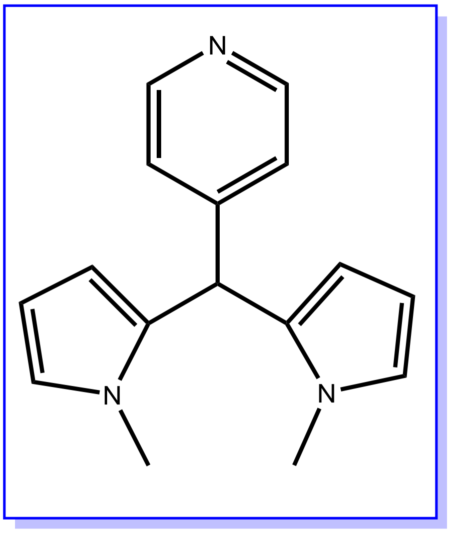

Obtained as inseparable mixture with the isomer **12aj** as a brown solid following general procedure described above (pag. S4) using *N*-methylpyrrole (**2a**) as nucleophile and *p*-pyridincarboxaldehyde (**10j**) as electrophile. Purification of the residue was carried out by column chromatography on silica flash (Hexane/EtOAc, 5/5) to give **11aj** in mixture with other regioisomer. ^{1}H NMR (400 MHz, $CDCl_3$, 298 K) : δ 3.38 (s, 6H, $N(CH_3)$), 5.26 (s, 1H, CH), 5.50 (bs, 2H, ArH_{Pyr}), 6.03 (bs, 2H, ArH_{Pyr}), 6.61 (bs, 2H, ArH_{Pyr}), 7.08 (d, J = 5.3 Hz, 2H, ArH), 8.54 (d, J = 5.3, 2H, ArH). ^{13}C NMR (100 MHz, $CDCl_3$, 298 K): δ 34.1, 41.6, 106.9, 109.6, 122.7, 124.2, 131.8, 150.2, 150.6. MALDI (m/z) calcd for $C_{16}H_{17}N_3$ $[M+H]^+$: 252.14952, found: 252.14968

3,3'-((4-(trifluoromethyl)phenyl)methylene)bis(1-methyl-1H-indole) (13ed)

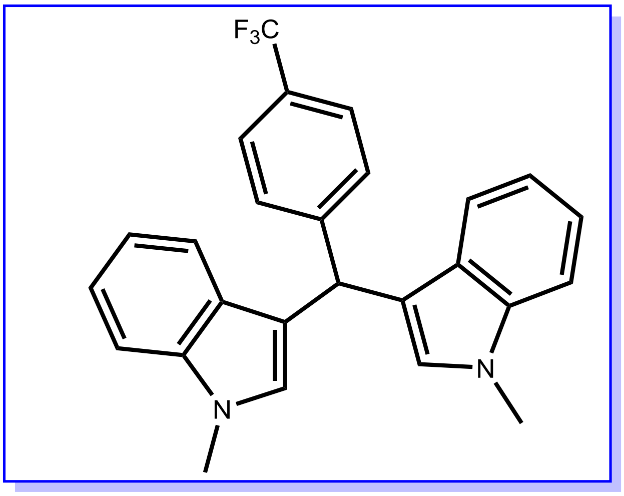

Obtained as pink solid (mp 157.1-158.3), following general procedure described (pag. S4) using *N*-methylindole (**8e**) as nucleophile and *p-(*trifluoromethyl)benzaldehyde (**10d**) as electrophile. Purification of the residue was carried out by column chromatography on silica flash (Hexane/EtOAc, 8/2) to give 11ed**.** ^{1}H NMR (400 MHz, $CDCl_3$, 298 K) : δ 3.74 (s, 6H, $N(CH_3)$), 6.00 (s, 1H, CH), 6.58 (s, 2H, ArH_{Ind}), 7.07 (t, J = 7.8, 2H, ArH_{Ind}), 7.27 (t, J = 7.8, 2H, ArH_{Ind}),7.36 (d, J = 7.8 Hz, 2H, ArH_{Ind}), 7.40 (d, J =7.8, 2H, ArH_{Ind}), 7.50 (d, J = 8.1, 2H, ArH), 7.58 (d, J = 8.1, 2H, ArH). ^{13}C

NMR (100 MHz, $CDCl_3$, 298 K): δ 32.9, 40.2, 109.4, 119.1, 120.0, 121.9, 124.6 (q, $^1J_{CF}$ = 271.9 Hz), 125.4 (q, $^3J_{CF}$ = 3.5 Hz), 125.4, 127,4, 128.5 (q, $^2J_{CF}$ = 32.1 Hz), 128.5, 129.2, 137.6, 148.8. ESI (m/z) calcd for $C_{26}H_{21}N_2$ $[M]^+$: 418.16513, found: 418.16052

3,3'-((2-nitrophenyl)methylene)bis(1-methyl-1H-indole) (13eb)

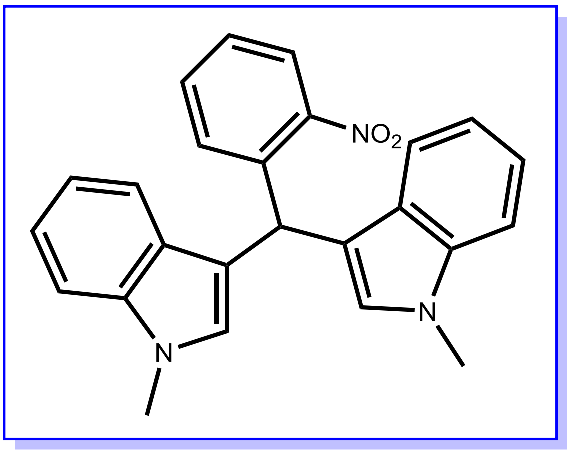

Obtained as orange solid (mp 242°C decomp.), following general procedure described above (pag. S4) using *N*-methylindole (**8e**) as nucleophile and *o*-nitrobenzaldehyde (**10b**) as electrophile. Purification of the residue was carried out by column chromatography on silica flash (Hexane/EtOAc, 8/2) to give **13eb.** ^{1}H NMR (600 MHz, $CDCl_3$, 298 K) : δ 3.68 (s, 6H, $N(CH_3)$), 6.54 (s, 2H, ArH_{Ind}), 6.67 (s, 1H, CH), 7.02 (t, J = 7.8, 2H, ArH_{Ind}), 7.21 (t, J = 7.8, 2H, ArH_{Ind}), 7.30 (d, J = 7.8 Hz, 2H, ArH_{Ind}), 7.34 (t, J= 7.9, 1H, ArH), 7.38 (d, J = 7.9, 2H, ArH_{Ind}), 7.41 (t, J=7.9, 1H, ArH), 7.46 (d, J =7.9, 1H, ArH), 7.85 (d, J = 7.9, 1H, ArH). ^{13}C NMR (150 MHz, $CDCl_3$, 298 K): δ 33.0, 40.2, 109.4, 116.5, 119.2, 120.1, 122.0, 124.6, 127.3, 127.4, 128.8, 131.4, 132,5, 137.7, 138,7, 150.0. ESI (m/z) calcd for $C_{25}H_{21}N_3O_2$ $[M]^+$: 395.16283, found: 395.15819

3,3'-(pyridin-4-ylmethylene)bis(1-methyl-1H-indole) (13ej)

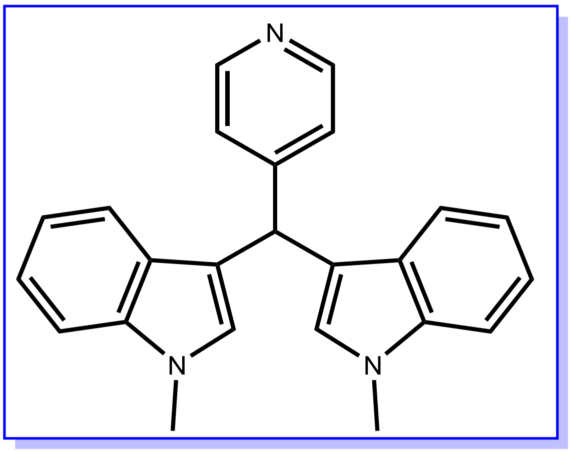

Obtained as pink solid (mp 164.6-165.4) following general procedure described above (pag. S4) using *N*-methylindole (**8e**) as nucleophile and *p*-pyridincarboxaldehyde (**10j**) as electrophile. Purification of the residue was carried out by column chromatography on silica flash (Hexane/EtOAc, 5/5) to give **11ej.** ^{1}H NMR (600 MHz, $CDCl_3$, 298 K) : δ 3.72 (s, 6H, $N(CH_3)$)), 5.98 (s, 1H, CH), 6.57 (s, 2H, ArH_{Ind}), 7.04 (t, J = 7.3, 2H, ArH_{Ind}), 7.25 (t, J = 7.3, 2H, ArH_{Ind}), 7.29 (d, J = 6.5 Hz, 2H, ArH), 7.34 (d, J=7.3, 1H, ArH_{Ind}), 7.37 (d, J =7.3 , 2H, ArH_{Ind}), 8.5 (d, J = 6.5, 2H, ArH). ^{13}C NMR (150 MHz, $CDCl_3$, 298 K): δ 32.9, 39.7, 109.4, 116.5, 119.1, 119.8, 121.9, 124.2, 127.3, 128.4, 137.6, 149.8, 153.7. ESI (m/z) calcd for $C_{24}H_{21}N_3$ $[M+H]^+$: 352.18082, found: 352.18439.

3,3'-((4-bromophenyl)methylene)bis(1-methyl-1H-indole) (13ee)

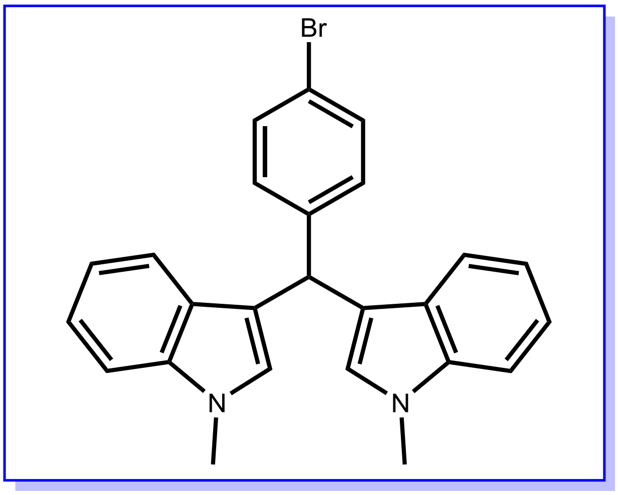

Obtained as red solid (mp 202.3-203.5.), following general procedure described above (pag. S4) using *N*-methylindole (**8e**) as nucleophile and *p*-bromobenzaldehyde (**10e**) as electrophile. Purification of the residue was carried out by column chromatography on silica flash (Hexane/EtOAc, 8/2) to give **13ee.** ^{1}H NMR (400 MHz, $CDCl_3$, 298 K) : δ 3.70 (s, 6H, $N(CH_3)$)), 5.86 (s, 1H, CH), 6.53 (s, 2H, ArH_{Ind}), 7.03 (t, J = 7.7, 2H, ArH_{Ind}), 7.21-7.24 (m, 4H, overlapped ArH_{ind}), 7.32 (d, J = 7.7Hz, 2H, ArH_{Ind}), 7.38 (d, J=7.7, 1H, ArH), 7.41 (d, J =7.9 , 2H, ArH). ^{13}C NMR (75 MHz, $CDCl_3$, 298 K): δ 32.8, 39.7, 109.3, 117.8, 118.9, 119.9, 120.1, 121.7, 127.4, 128.4, 130.6, 131.4, 137.6, 143.7. ESI (m/z) calcd for $C_{25}H_{21}BrN_2$ $[M]^+$: 428.08826, found: 428.08363.

6. NMR and Mass Spectra of New Compounds

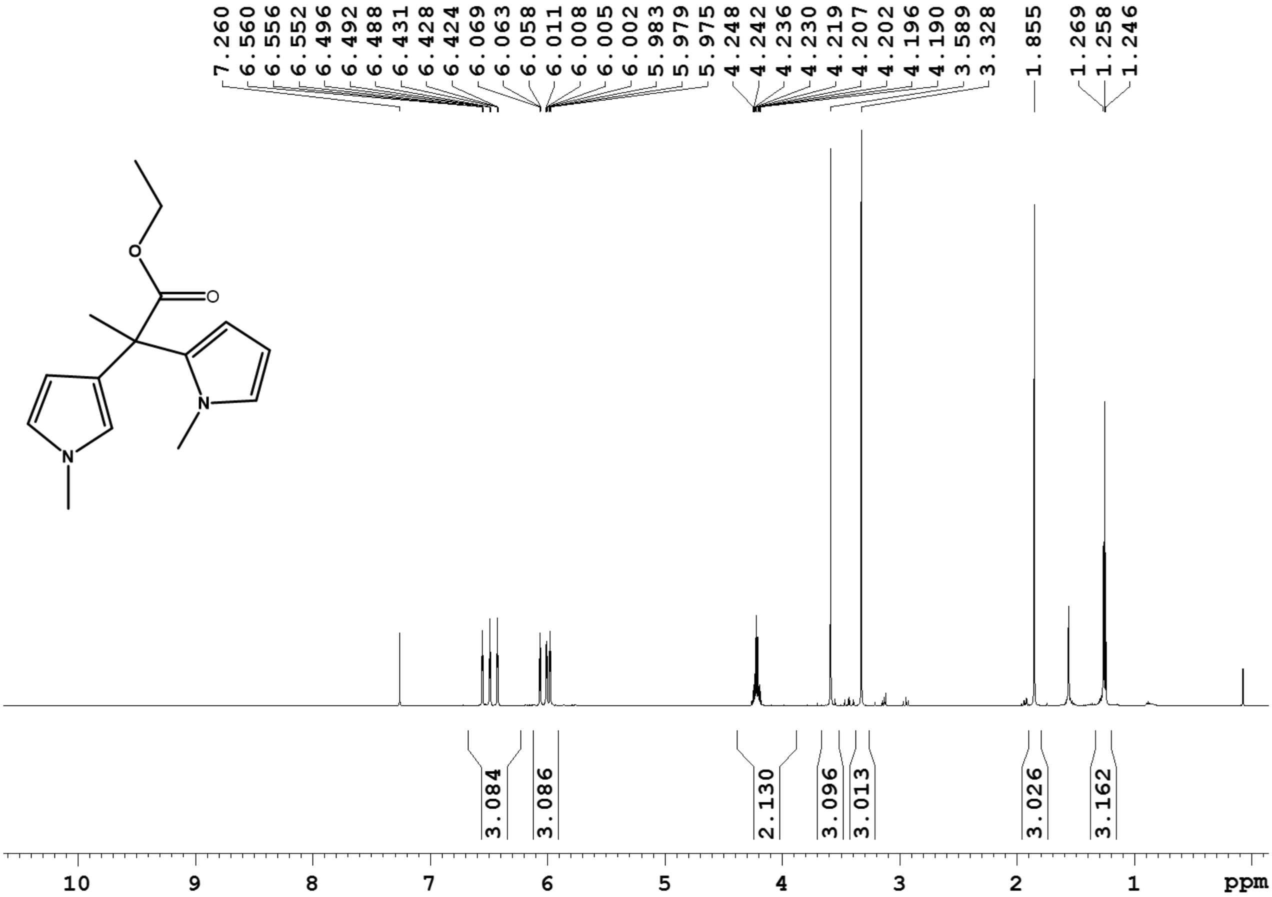

Figure S13. ^{1}H NMR (600 MHz, $CDCl_3$, 298 K) of the derivative **5aa**.

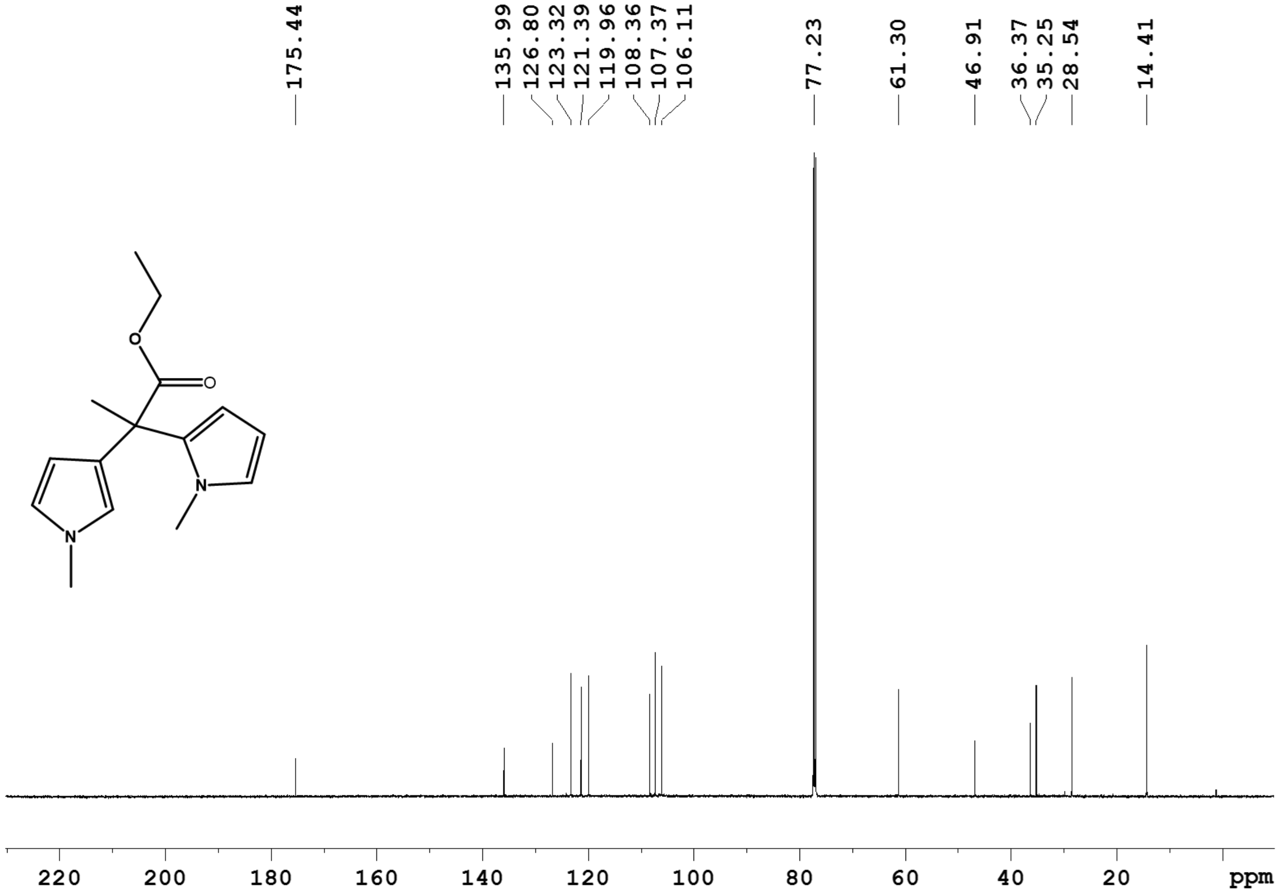

Figure S14. ^{13}C NMR (150 MHz, $CDCl_3$, 298 K) of the derivative **5aa**.

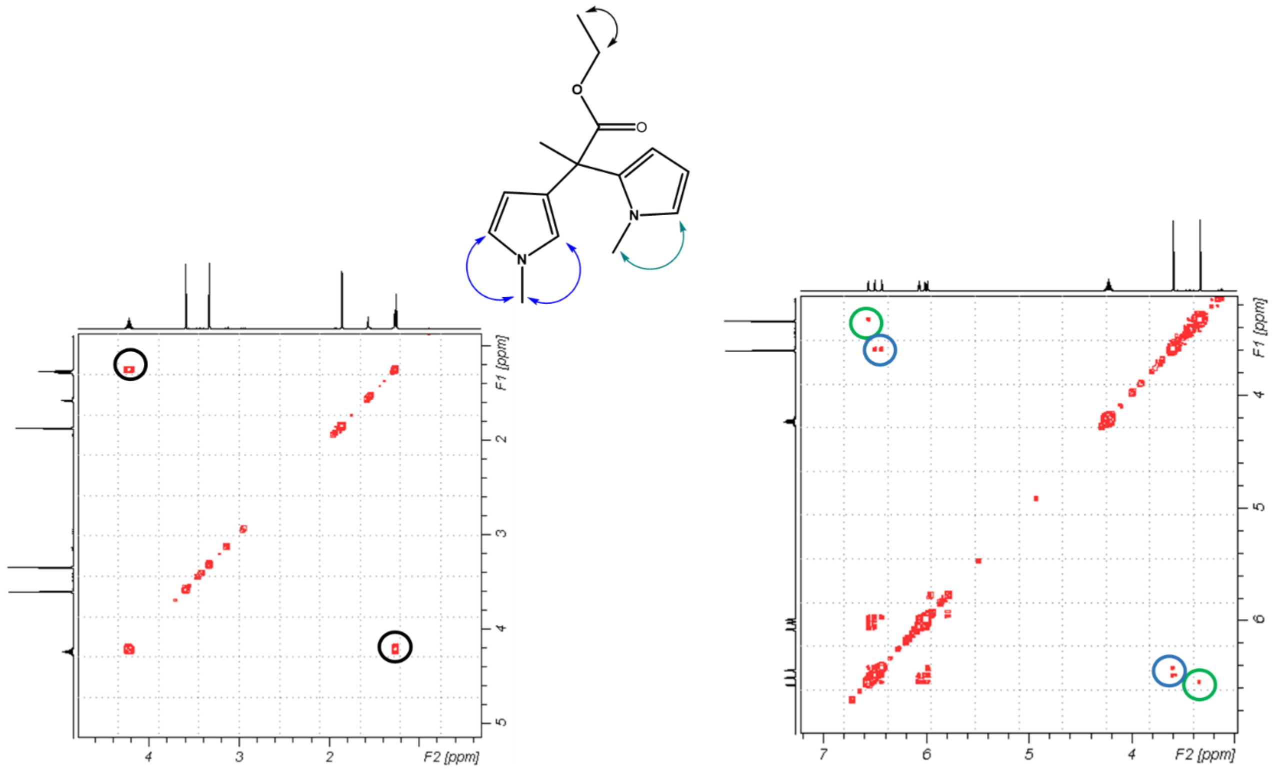

Figure S15. COSY NMR spectrum (600 MHz, $CDCl_3$, 298 K) of the derivative **5aa**.

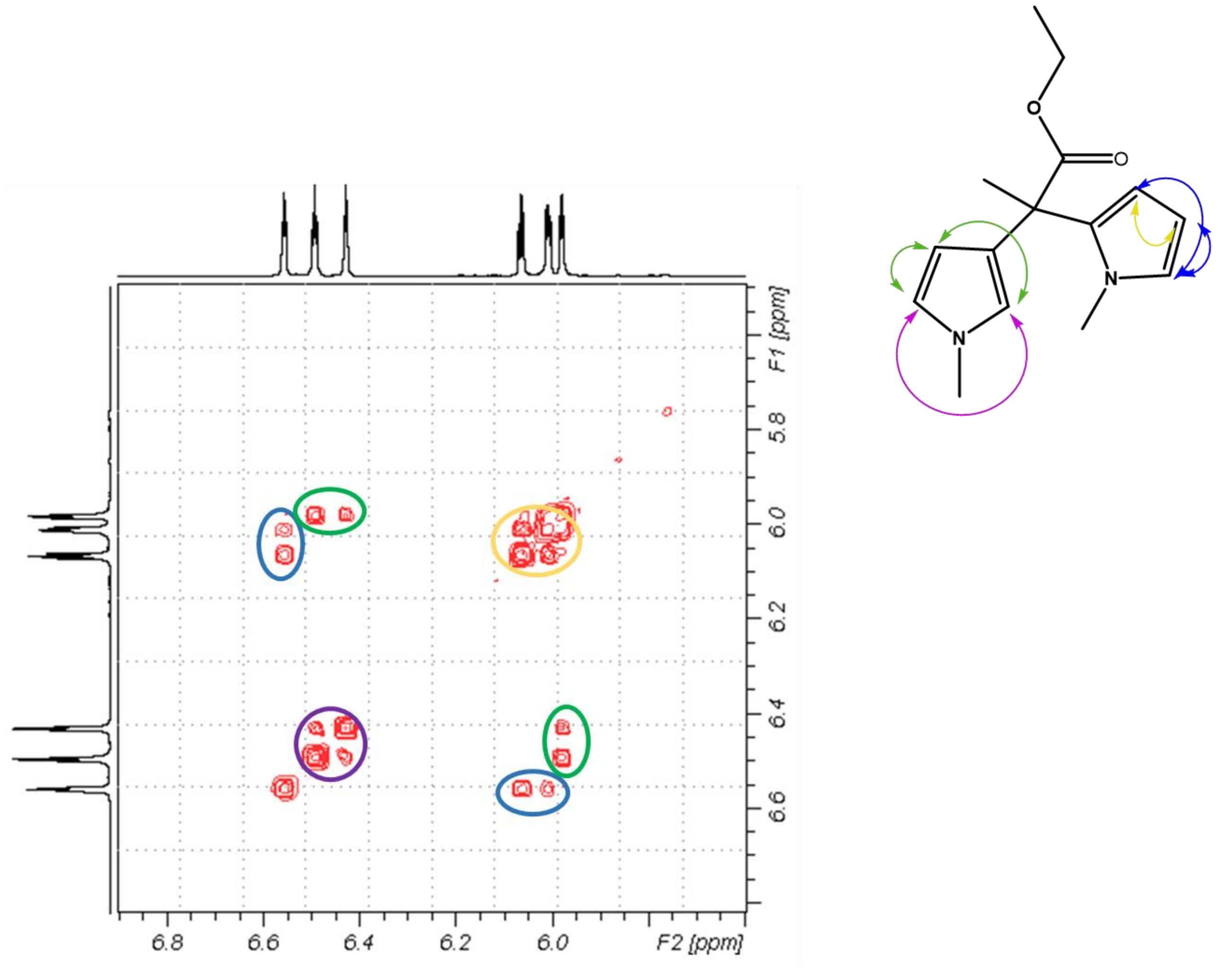

Figure S16. Expansion of COSY NMR spectrum (600 MHz, $CDCl_3$, 298 K) of the derivative **5aa.**

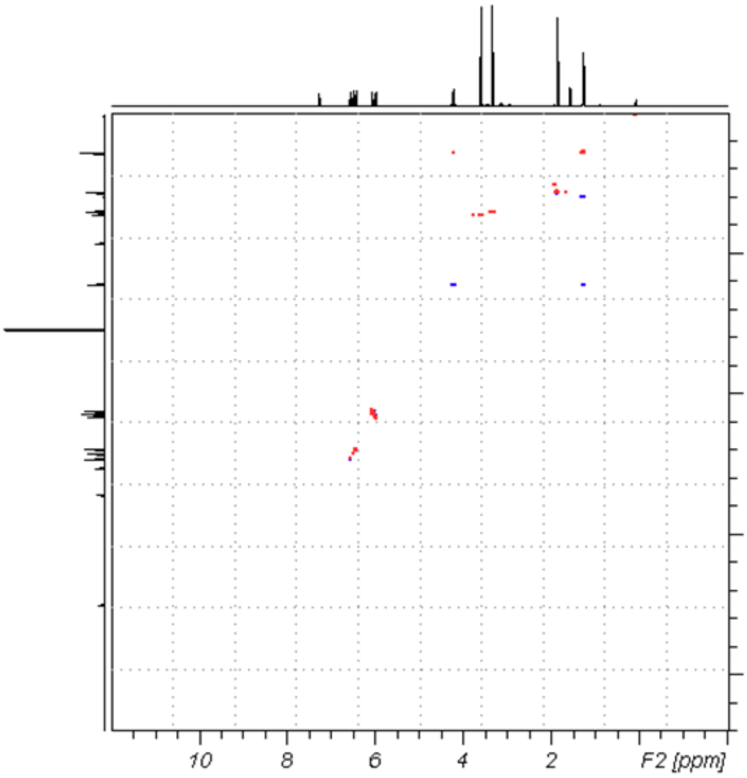

Figure S17. HSQC NMR spectrum (600 MHz, $CDCl_3$, 298 K) of the derivative **5aa**.

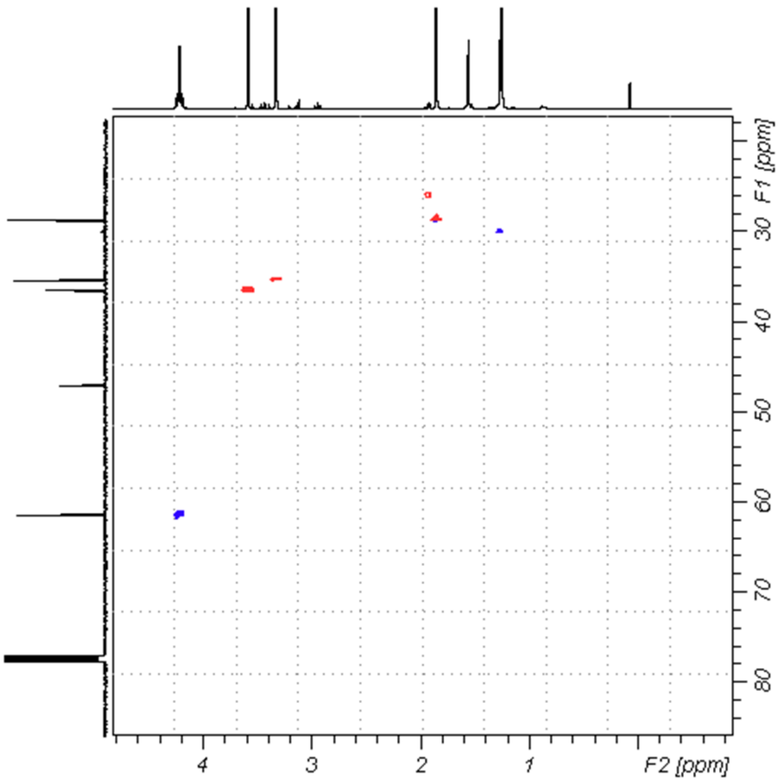

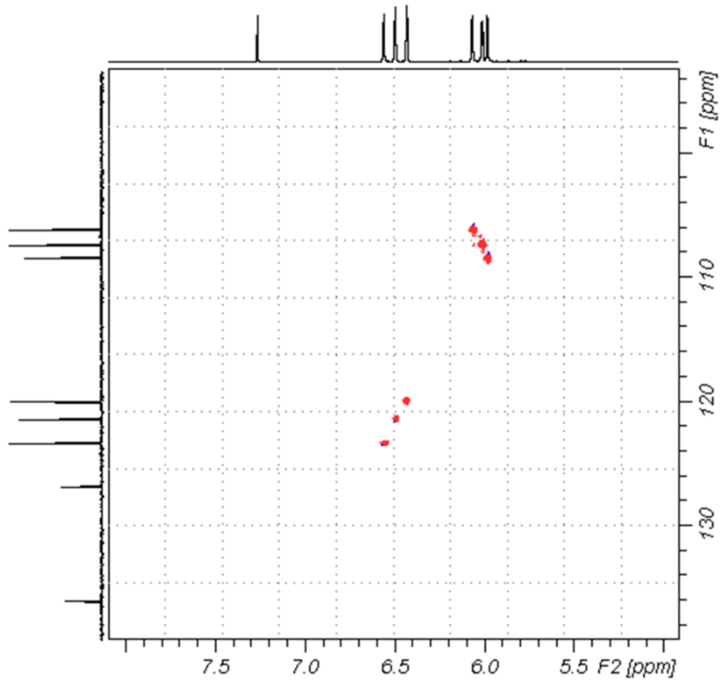

Figure S18. Expansions of HSQC NMR spectrum (600 MHz, $CDCl_3$, 298 K) of the derivative **5aa**.

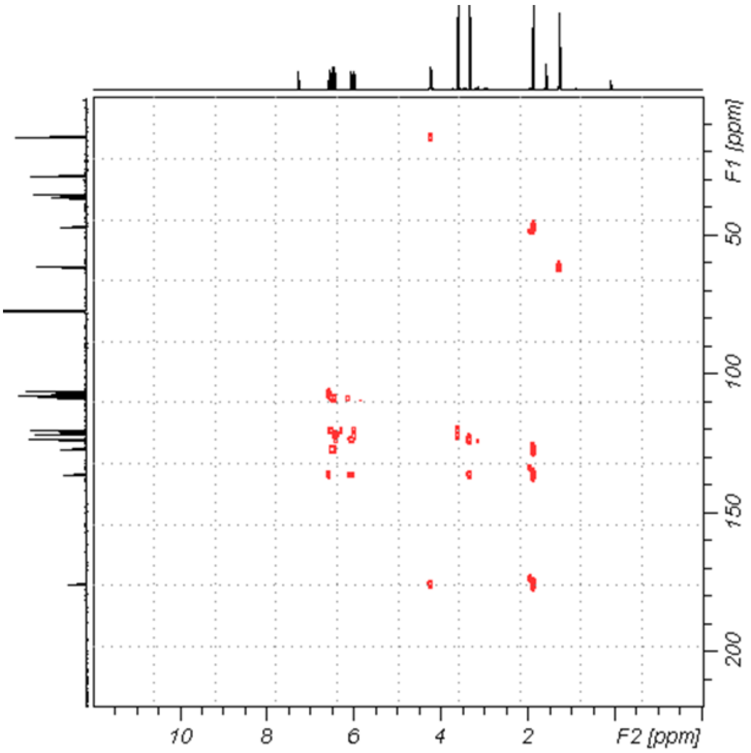

Figure S19. HMBC NMR spectrum (600 MHz, $CDCl_3$, 298 K) of the derivative **5aa**.

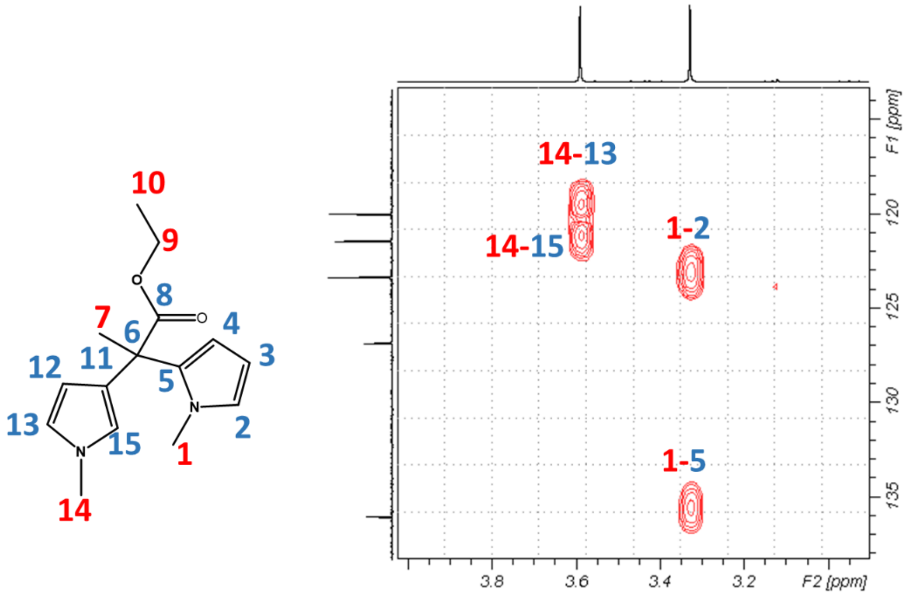

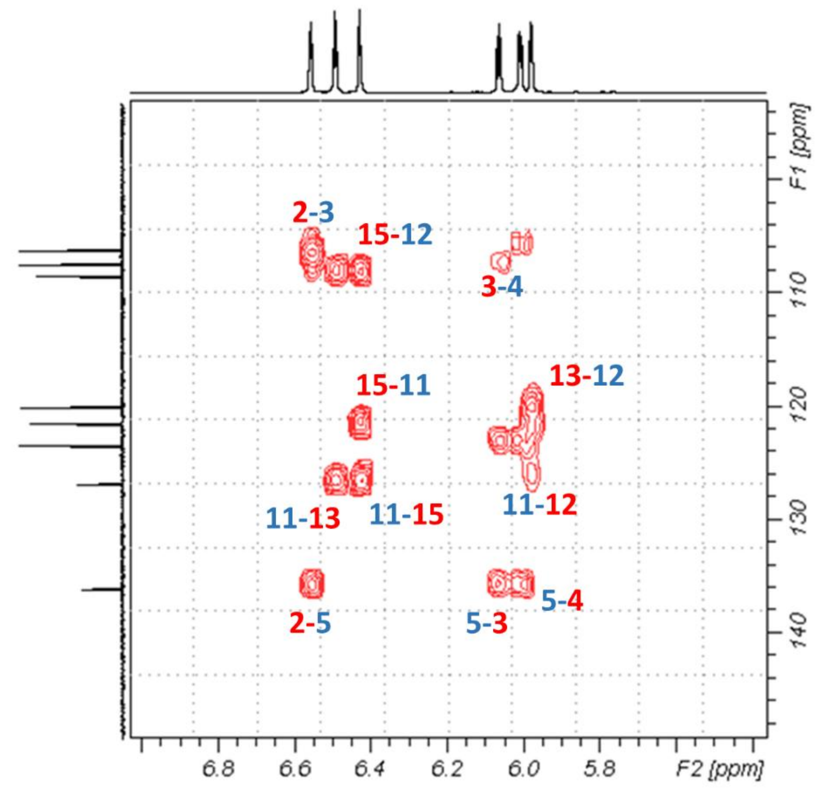
2-3
15-12
3-4
15-11
13-12
11-13
11-15
11-12
2-5
5-3
5-4
F1 [ppm]
110
120
130
140
6.8
6.6
6.4
6.2
6.0
5.8
F2 [ppm]

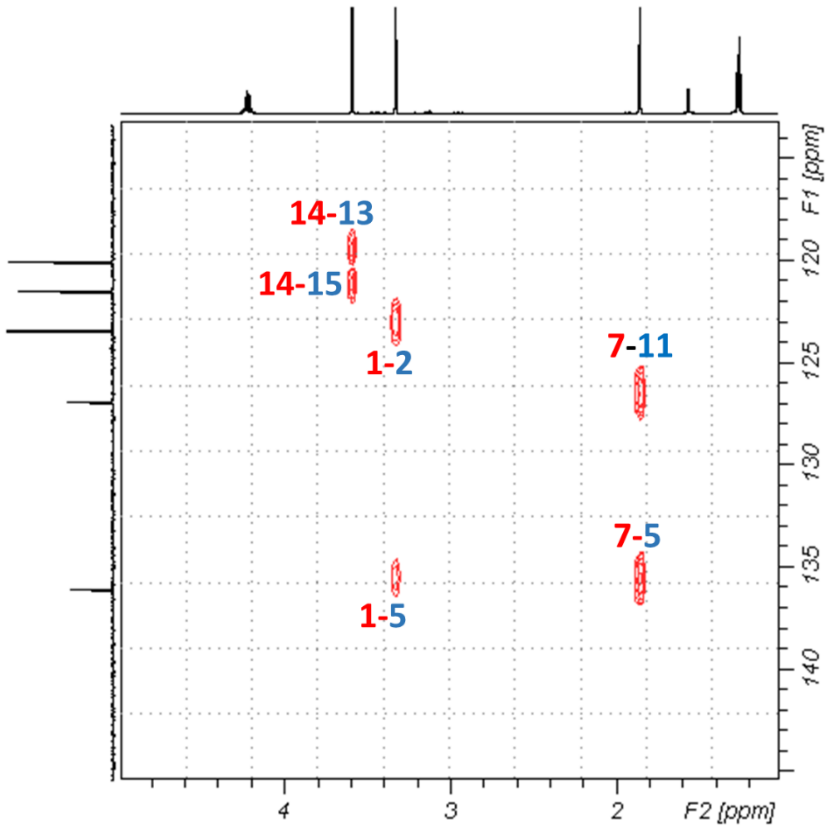
14-13
14-15
1-2
7-11
7-5
1-5
F1 [ppm]
120
125
130
135
140
4
3
2
F2 [ppm]

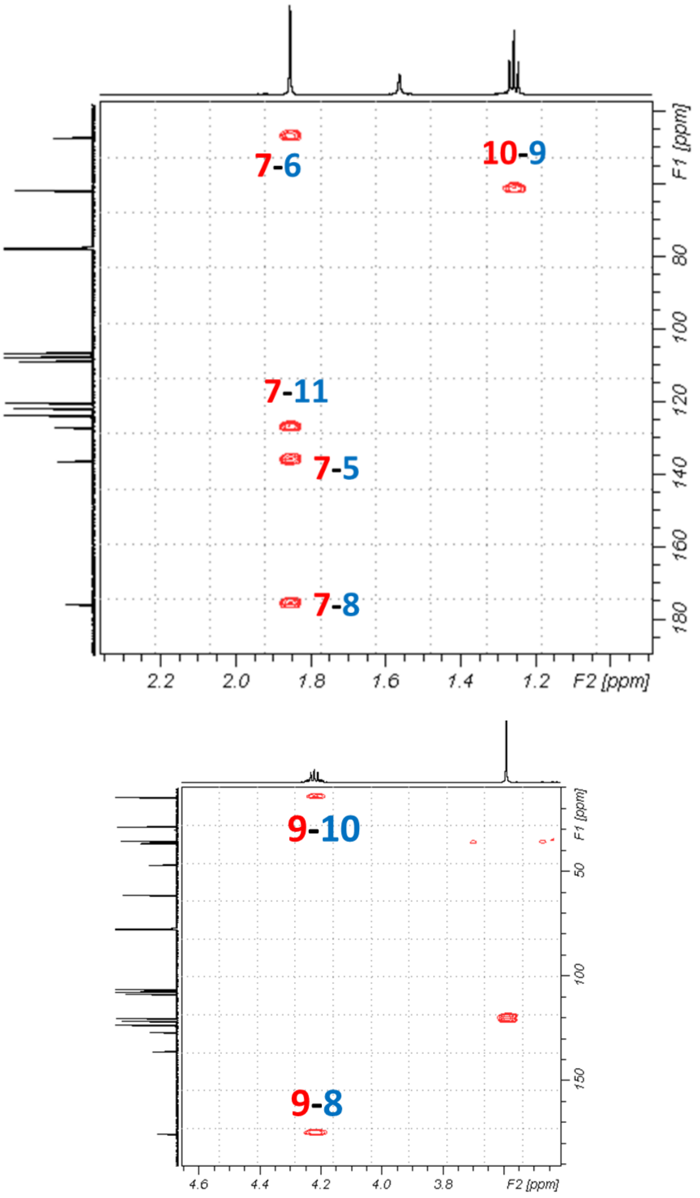

Figure S20. Expansions of HMBC NMR spectrum (600 MHz, $CDCl_3$, 298 K) of the derivative **5aa**.

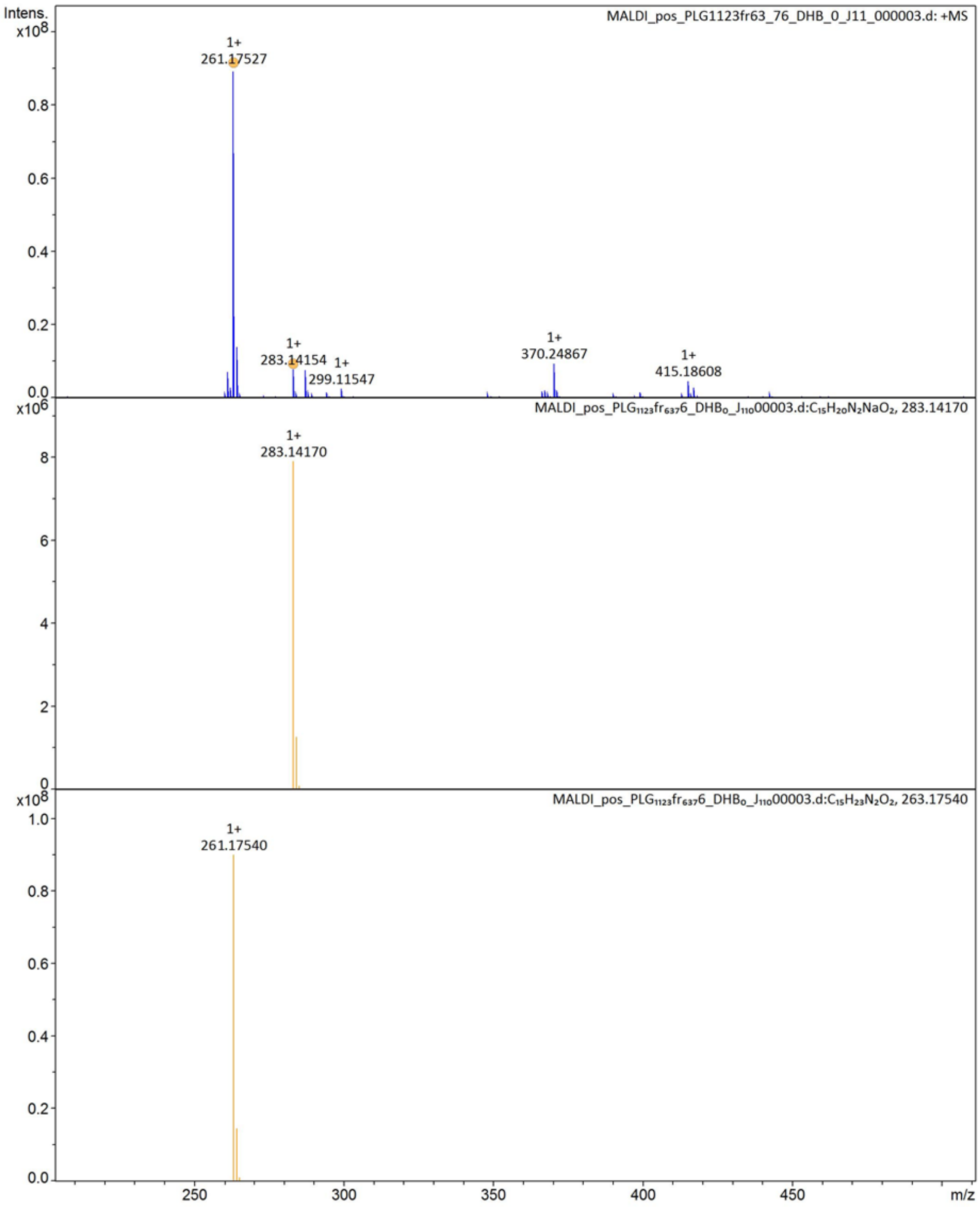

Figure S21. MALDI Mass spectrum of the derivative **5aa**.

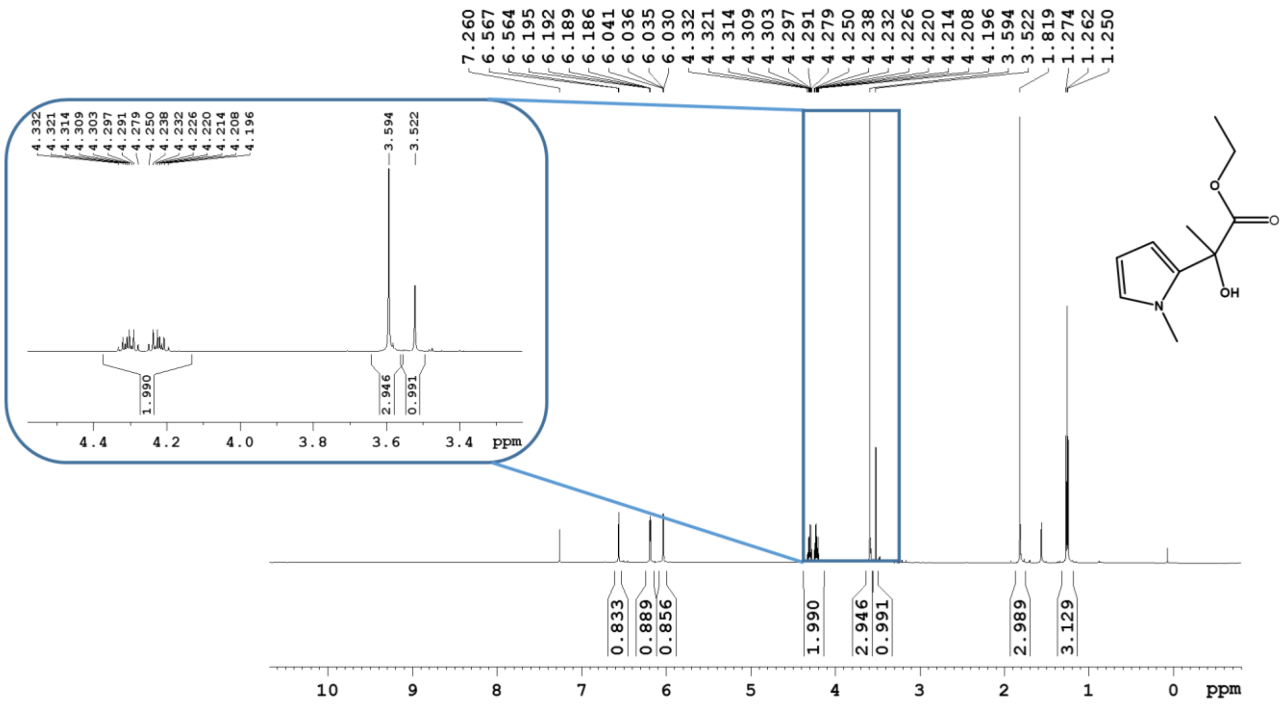

Figure S22. ^{1}H NMR (600 MHz, $CDCl_3$, 298 K) of the derivative **6aa** with expansion of relevant region between 4.5 and 3.3 ppm.

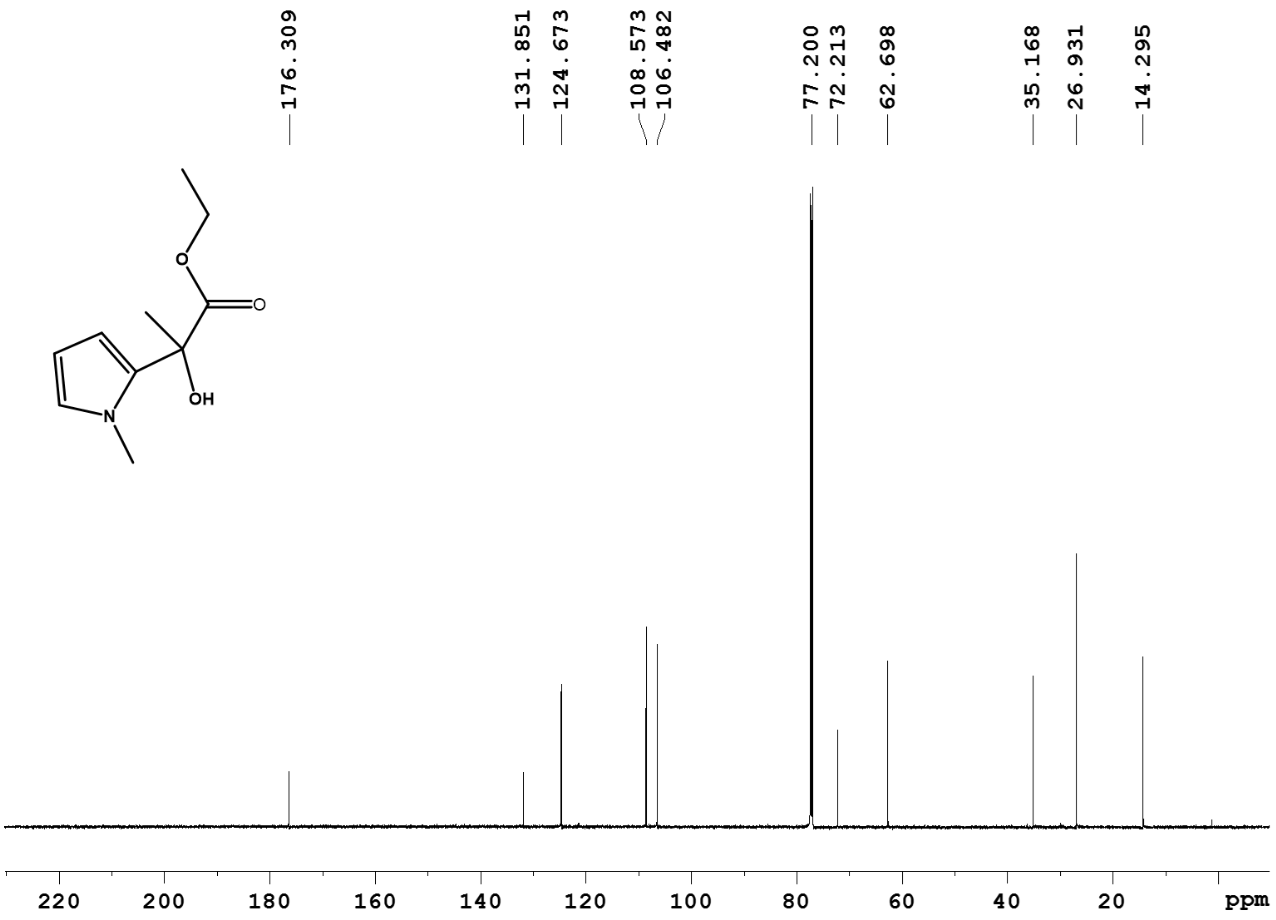

Figure S23. ^{13}C NMR (150 MHz, $CDCl_3$, 298 K) of the derivative **6aa**.

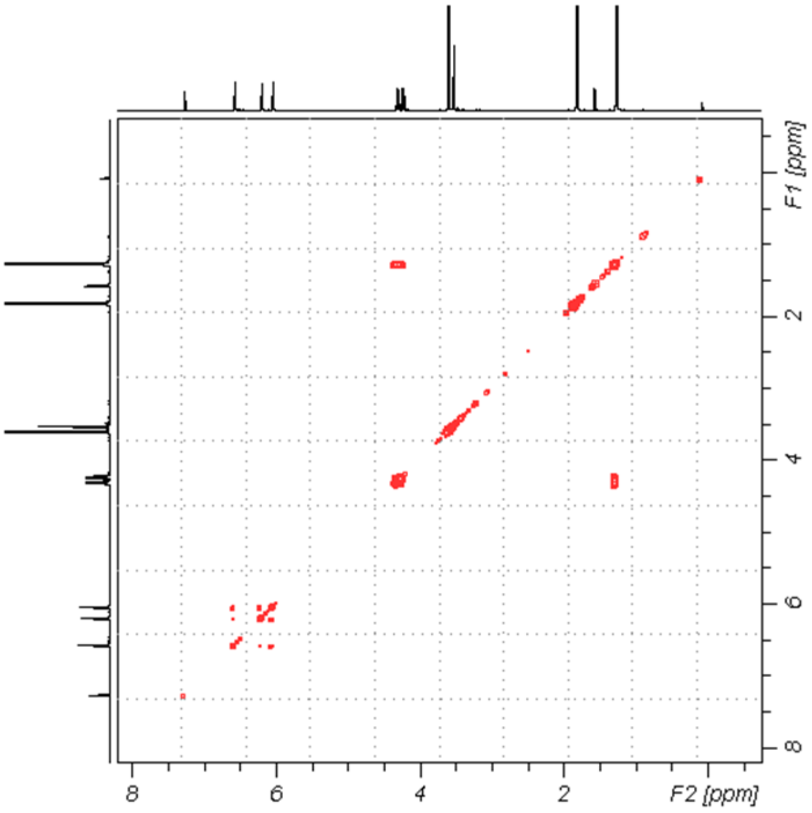

Figure S24. COSY NMR spectrum (600 MHz, $CDCl_3$, 298 K) of the derivative **6aa**.

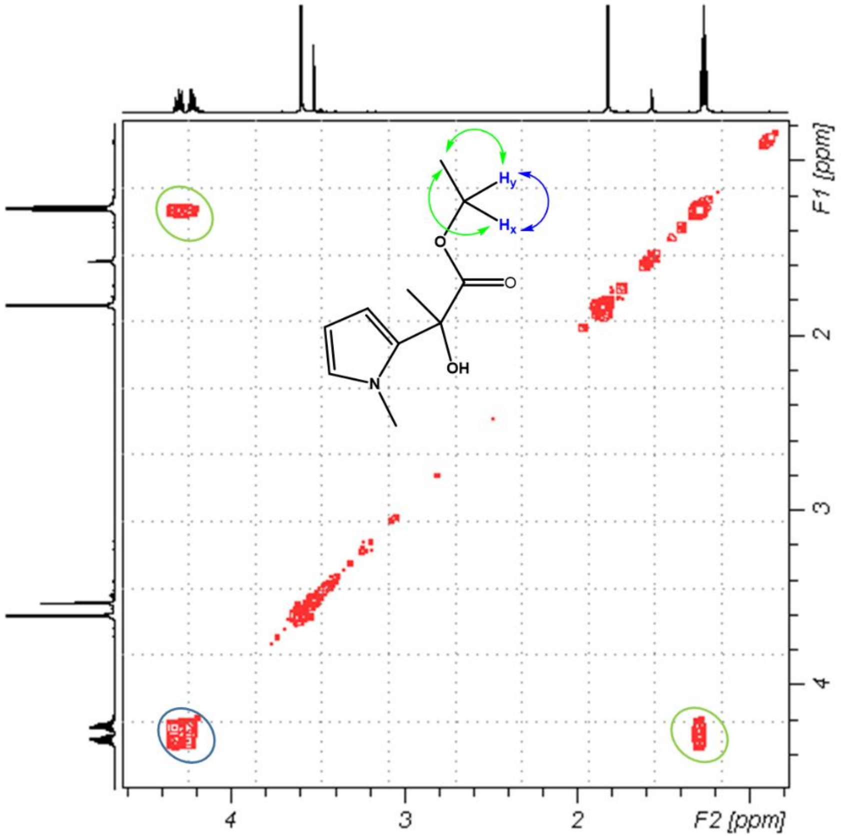

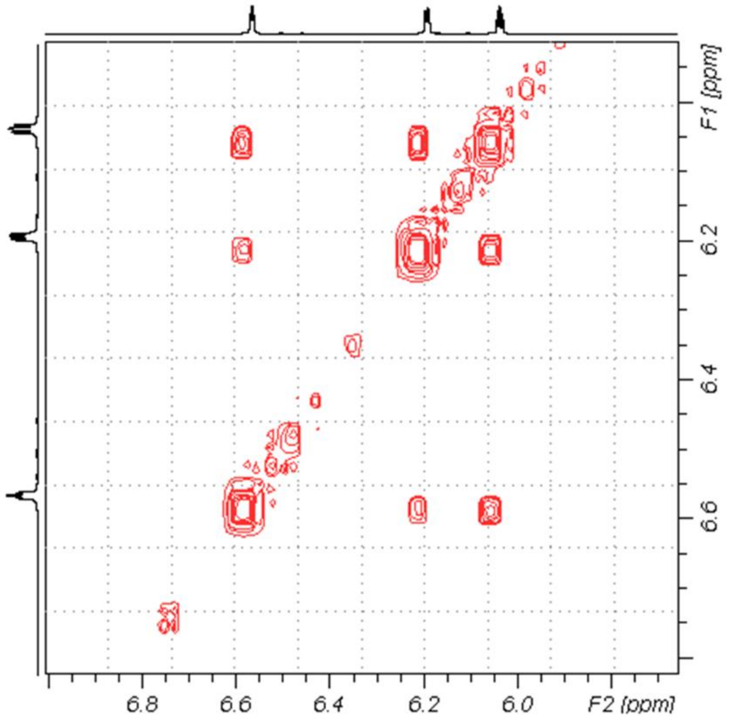

Figure S25. Expansions of COSY NMR spectrum (600 MHz, $CDCl_3$, 298 K) of the derivative **6aa**.

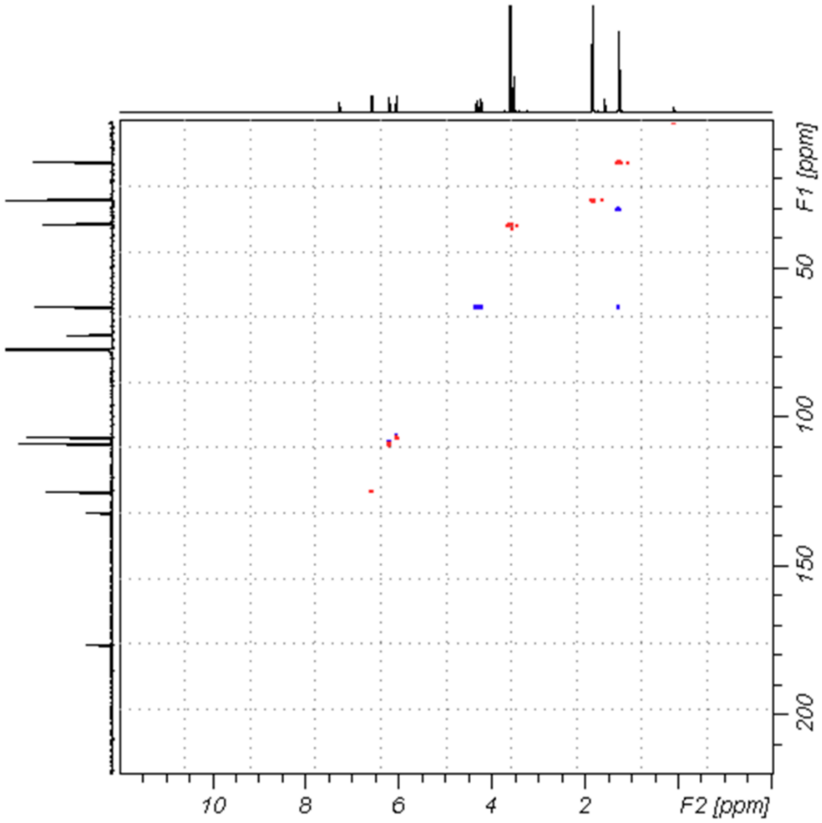

Figure S26. HSQC NMR spectrum (600 MHz, $CDCl_3$, 298 K) of the derivative **6aa**.

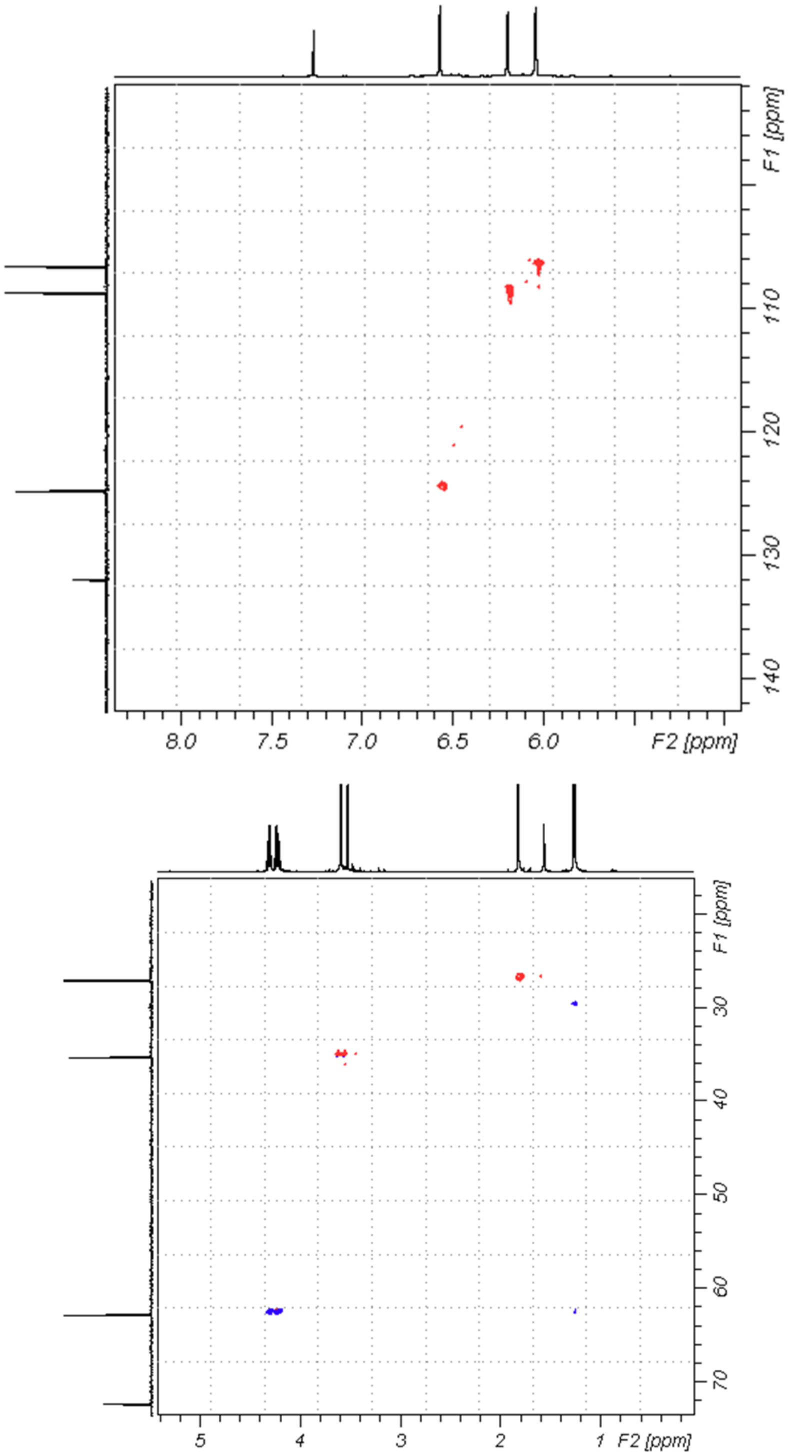

Figure S27. Expansions of HSQC NMR spectrum (600 MHz, $CDCl_3$, 298 K) of the derivative **6aa**.

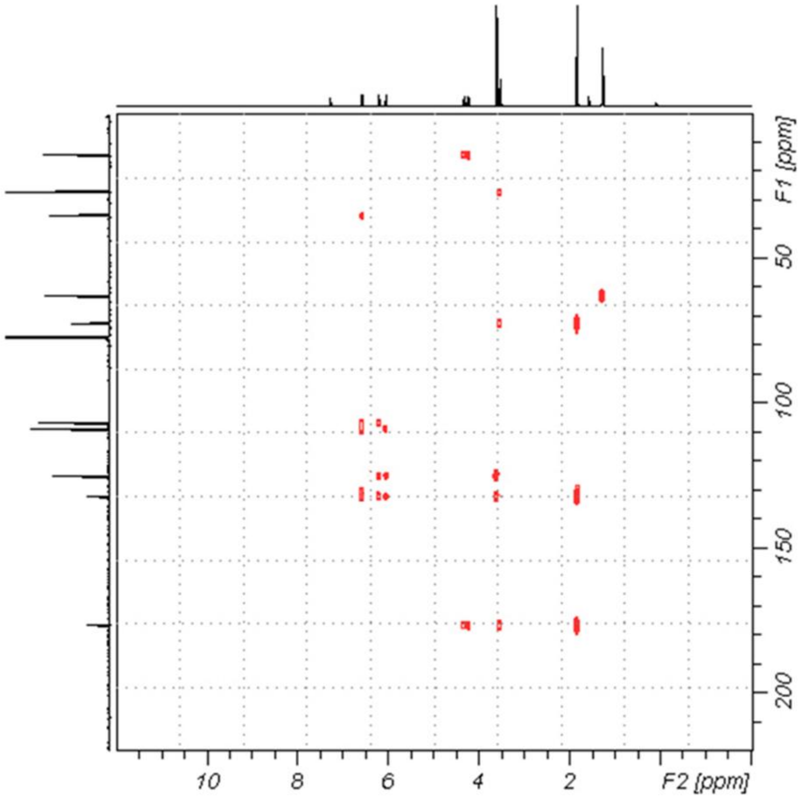

Figure S28. HMBC NMR spectrum (600 MHz, $CDCl_3$, 298 K) of the derivative **6aa**.

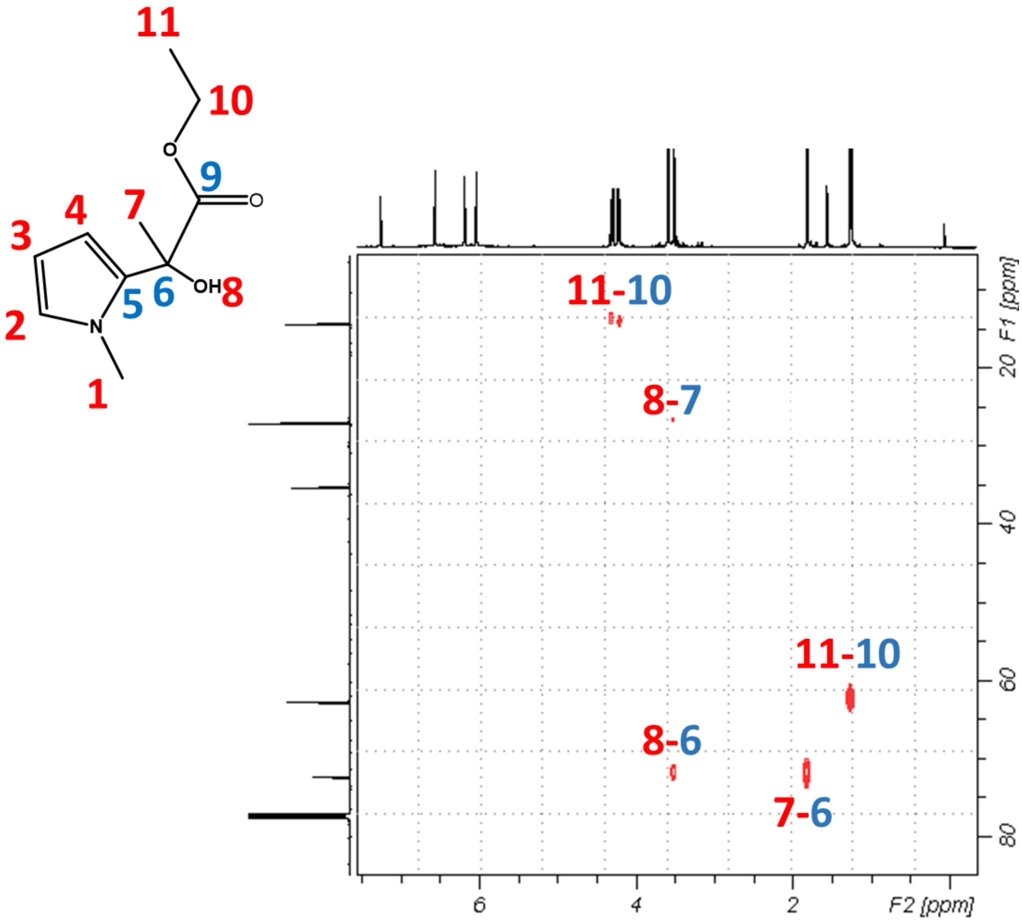

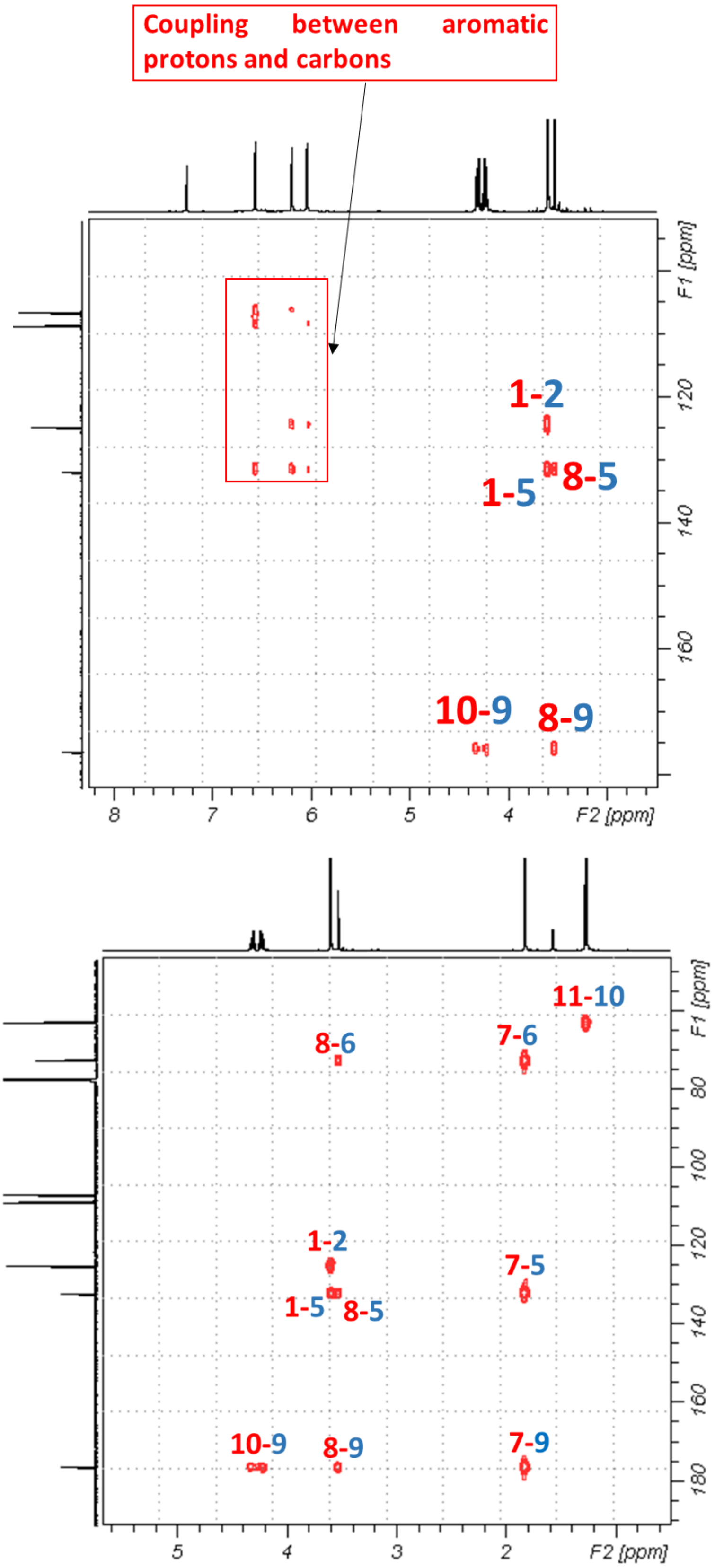

Figure S29. Expansions of HMBC NMR spectrum (600 MHz, $CDCl_3$, 298 K) of the derivative **6aa**.

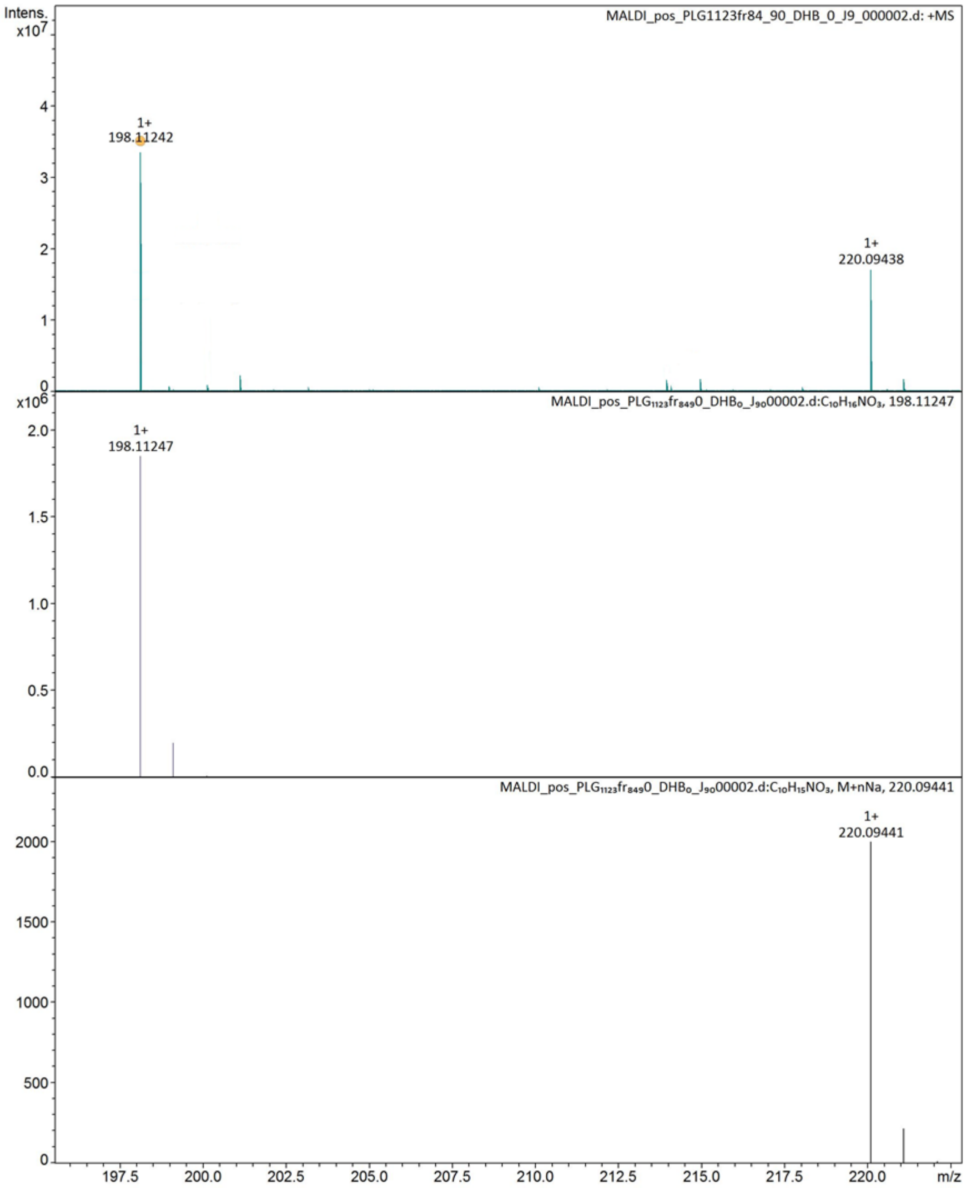

Figure S30. MALDI Mass spectrum of the derivative **6aa**.

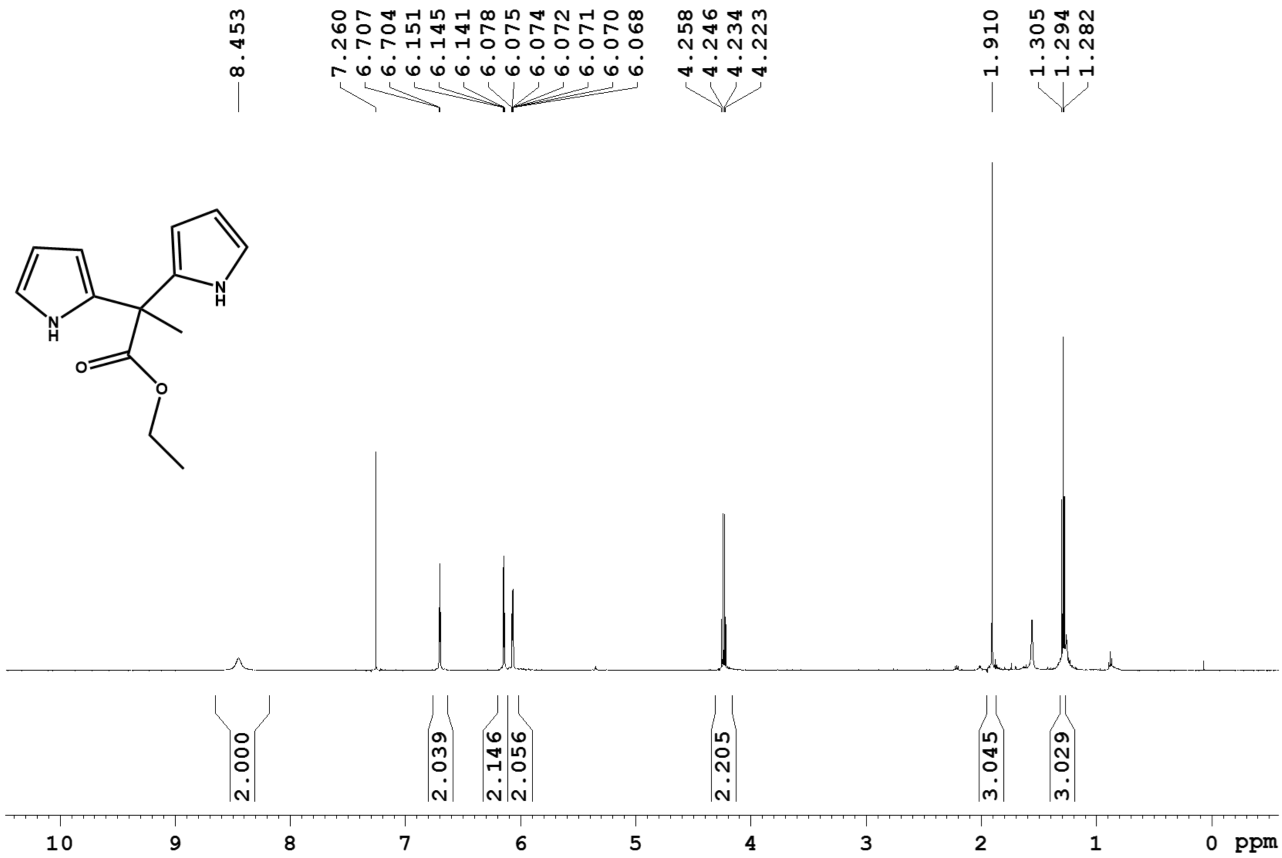

Figure S31. ^{1}H NMR (600 MHz, $CDCl_3$, 298 K) of the derivative **4ba.**

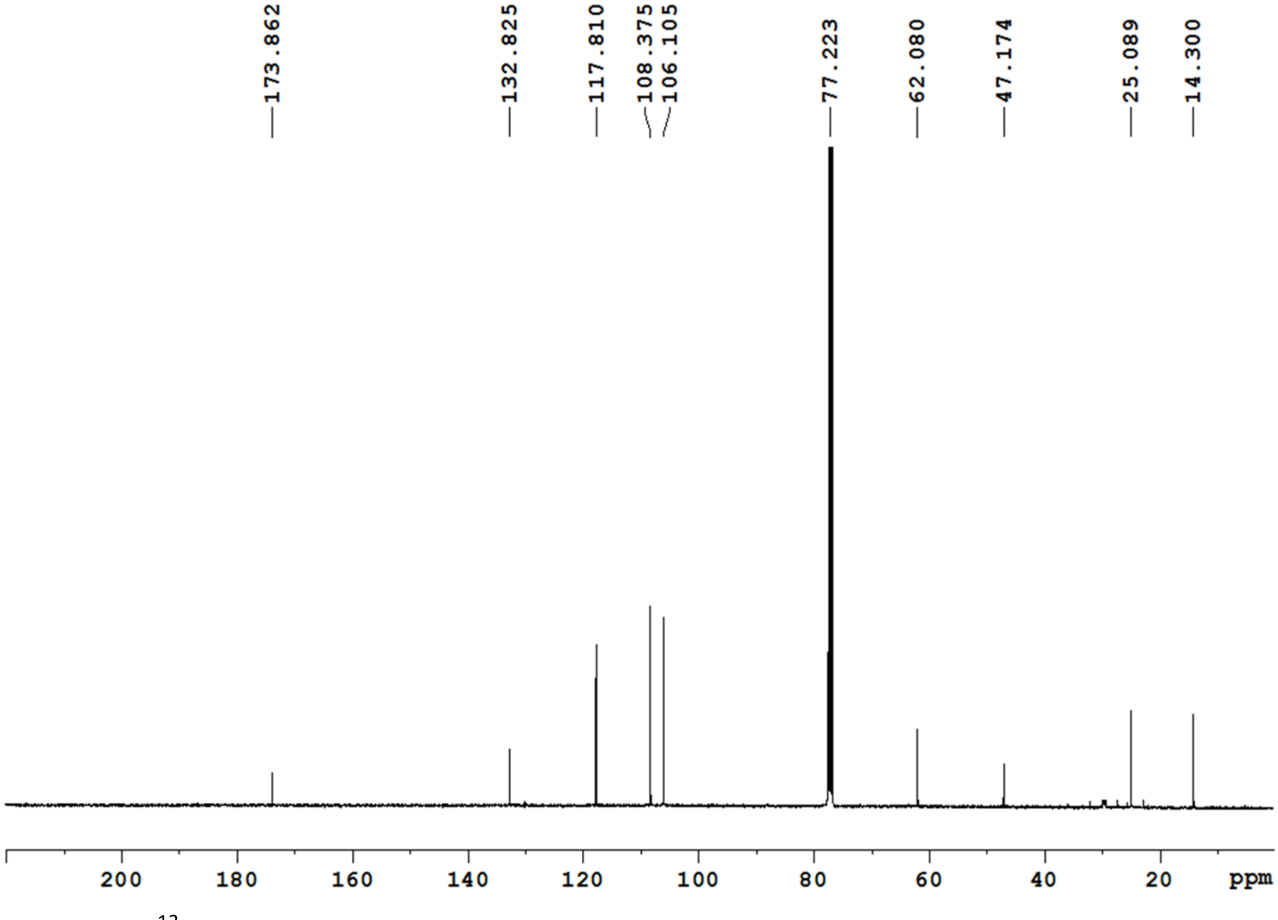

Figure S32. ^{13}C NMR (150 MHz, $CDCl_3$, 298 K) of the derivative **4ba**.

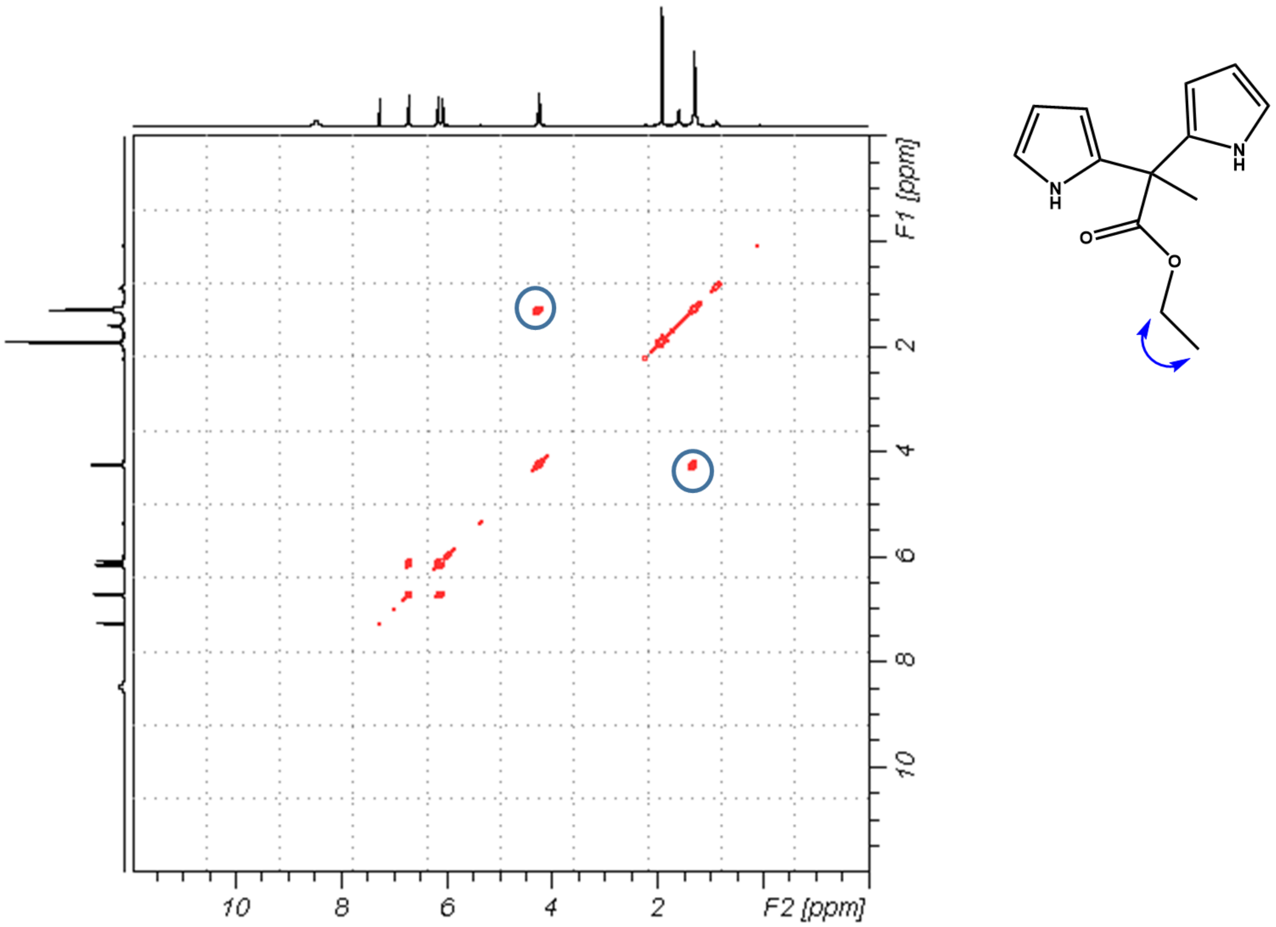

Figure S33. COSY NMR spectrum (600 MHz, $CDCl_3$, 298 K) of the derivative **4ba**.

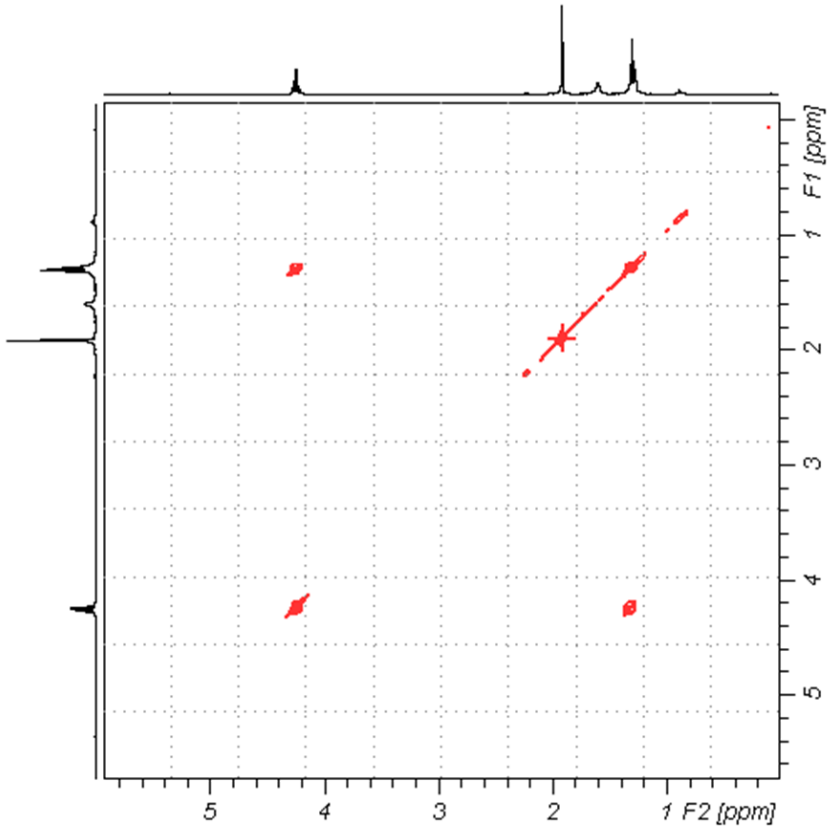

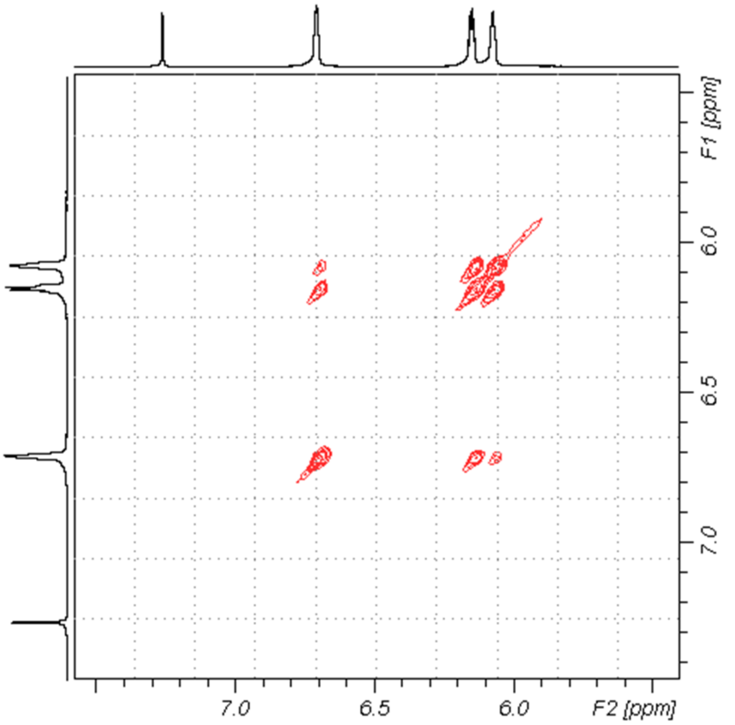

Figure S34. Expansions of COSY NMR spectrum (600 MHz, $CDCl_3$, 298 K) of the derivative **4ba**.

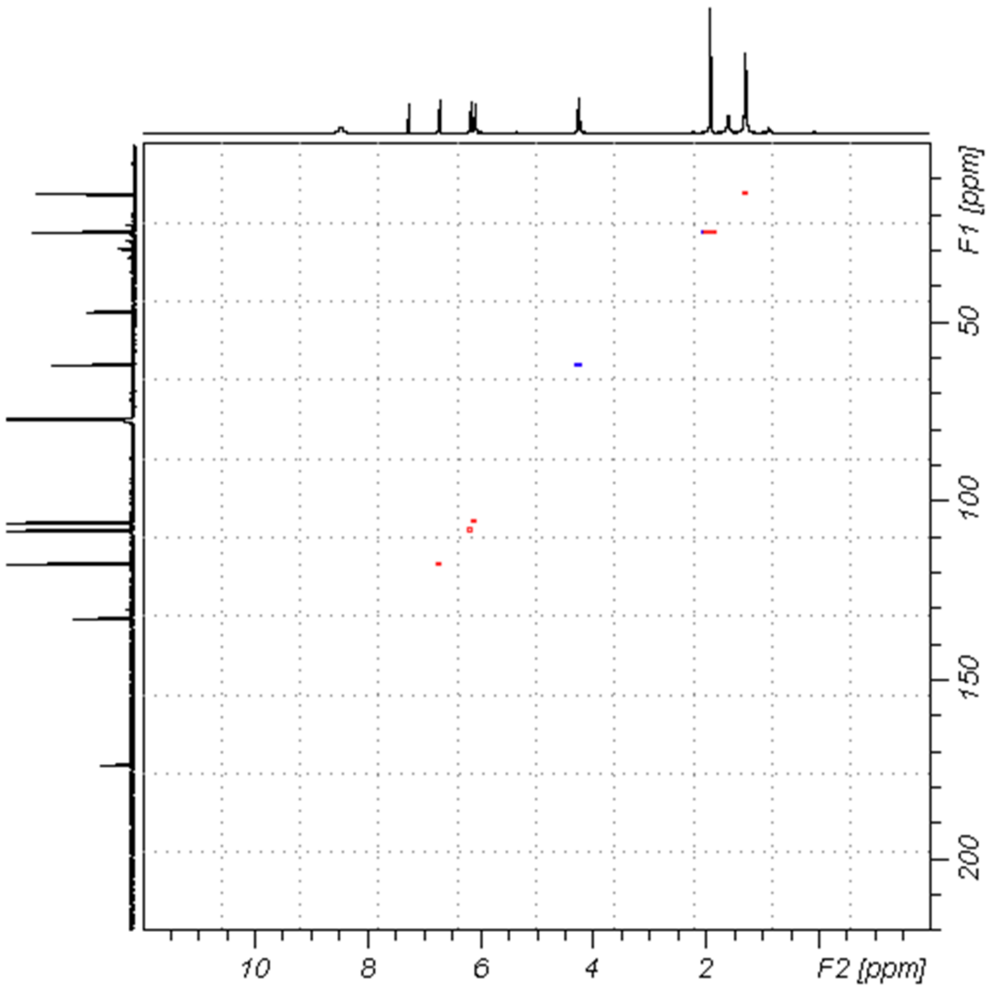

Figure S35. HSQC NMR spectrum (600 MHz, $CDCl_3$, 298 K) of the derivative **4ba.**

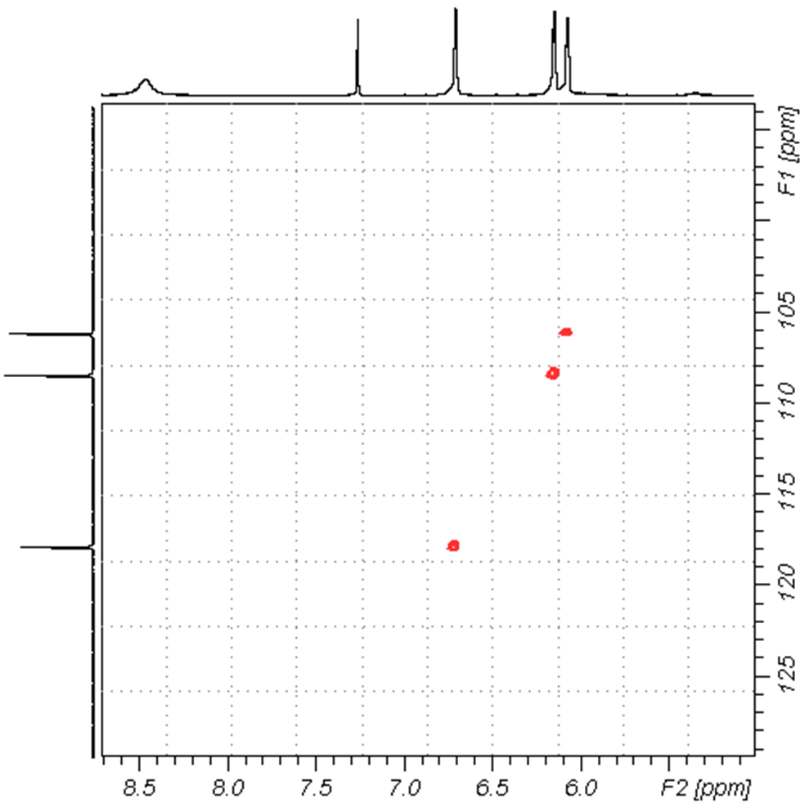

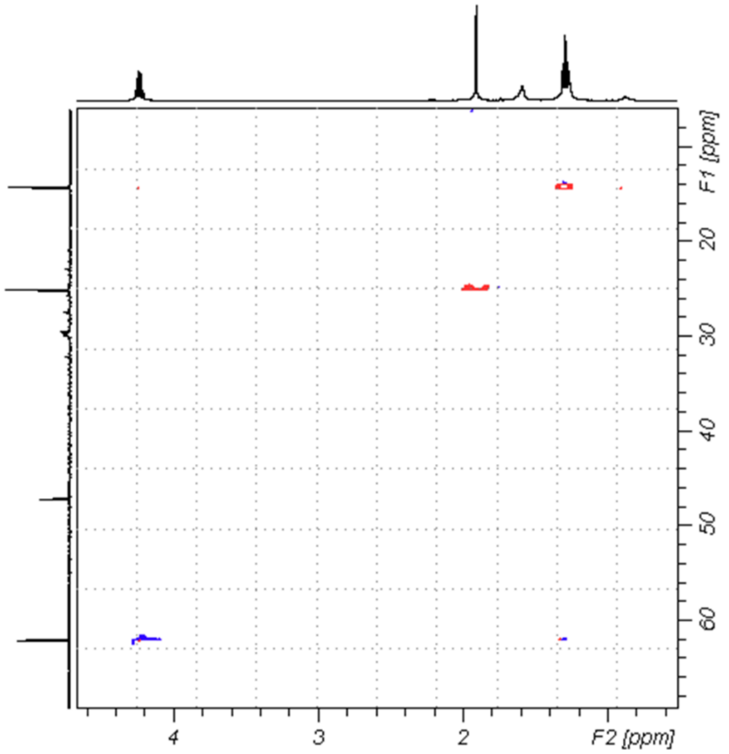

Figure S36. Expansions of HSQC NMR spectrum (600 MHz, $CDCl_3$, 298 K) of the derivative **4ba**.

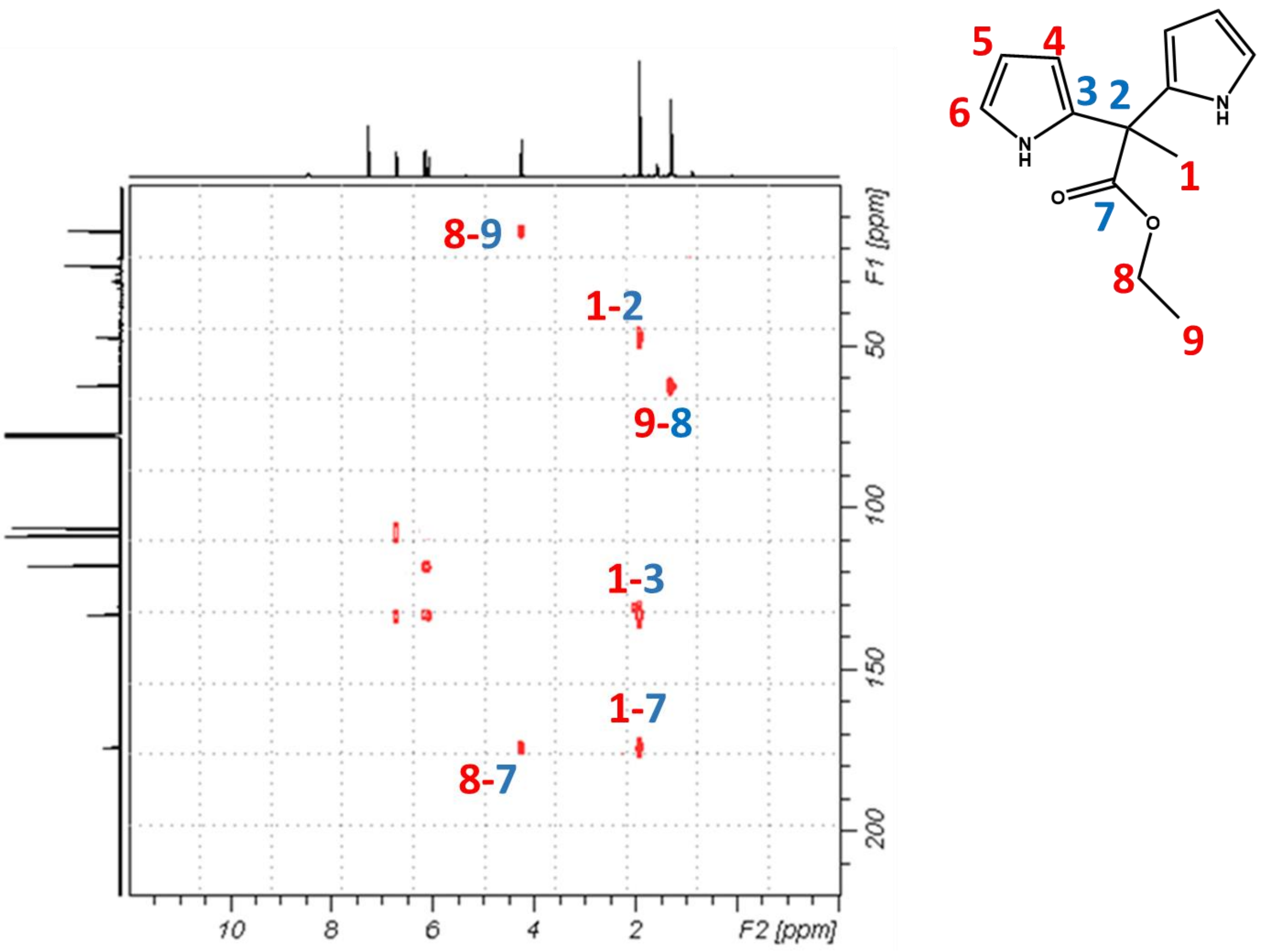

Figure S37. HMBC NMR spectrum (600 MHz, $CDCl_3$, 298 K) of the derivative **4ba.**

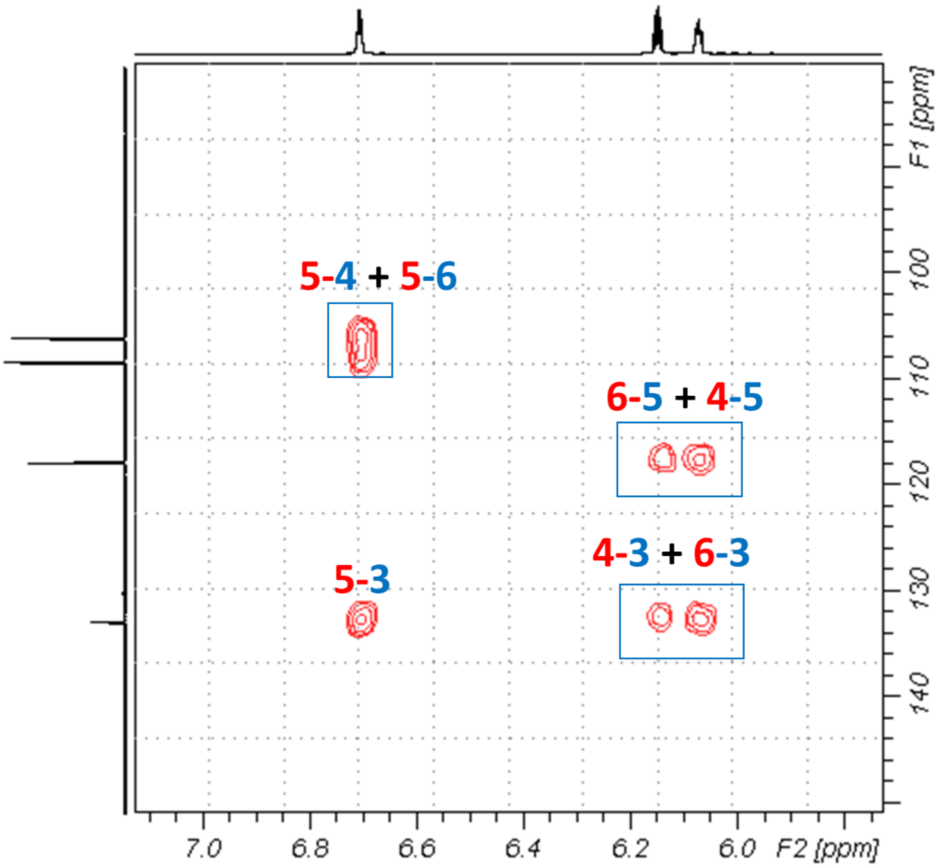

Figure S38. Expansion of HMBC NMR spectrum (600 MHz, $CDCl_3$, 298 K) of the derivative **4ba**.

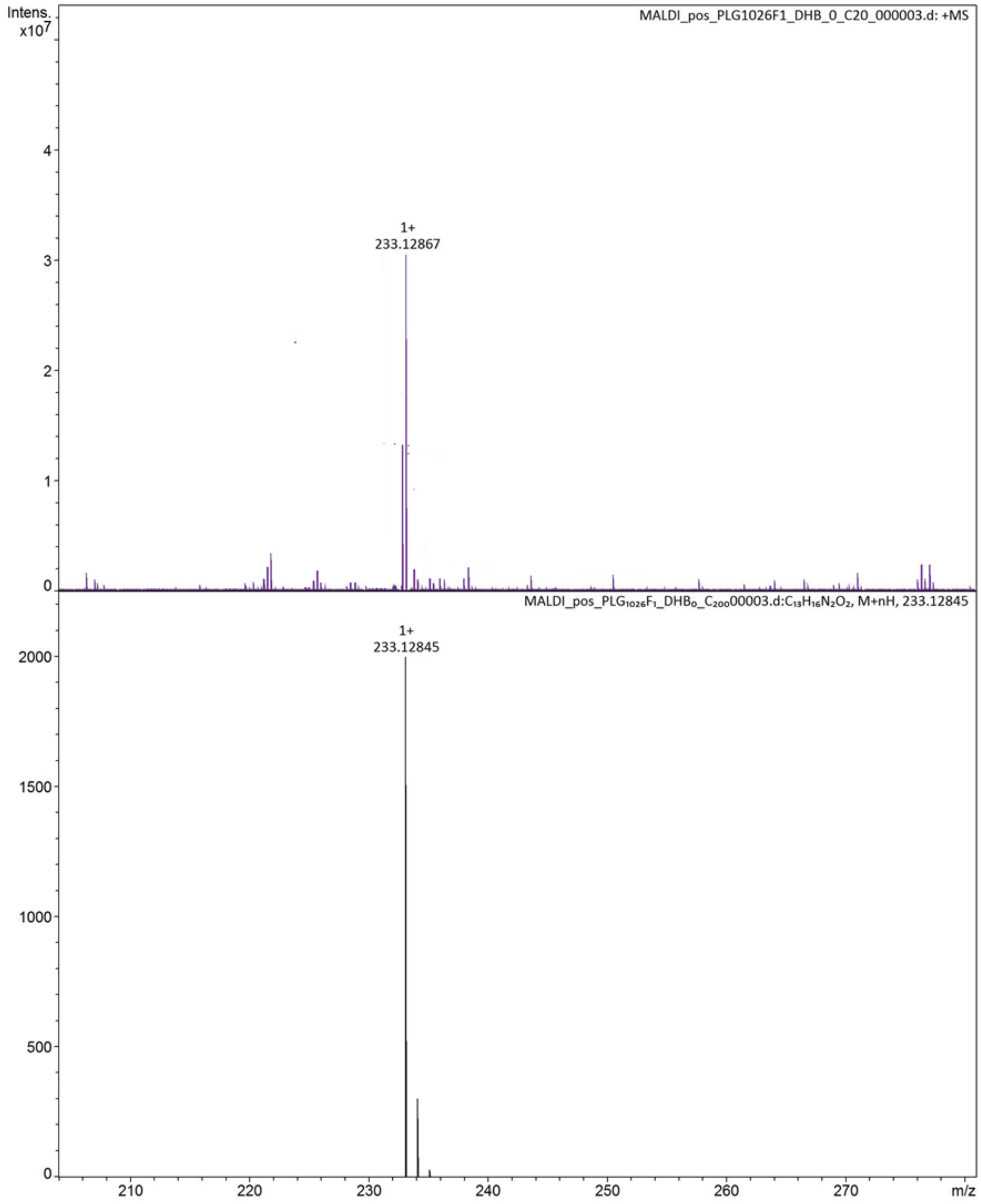

Figure S39. MALDI Mass Spectrum of the derivative **4ba.**

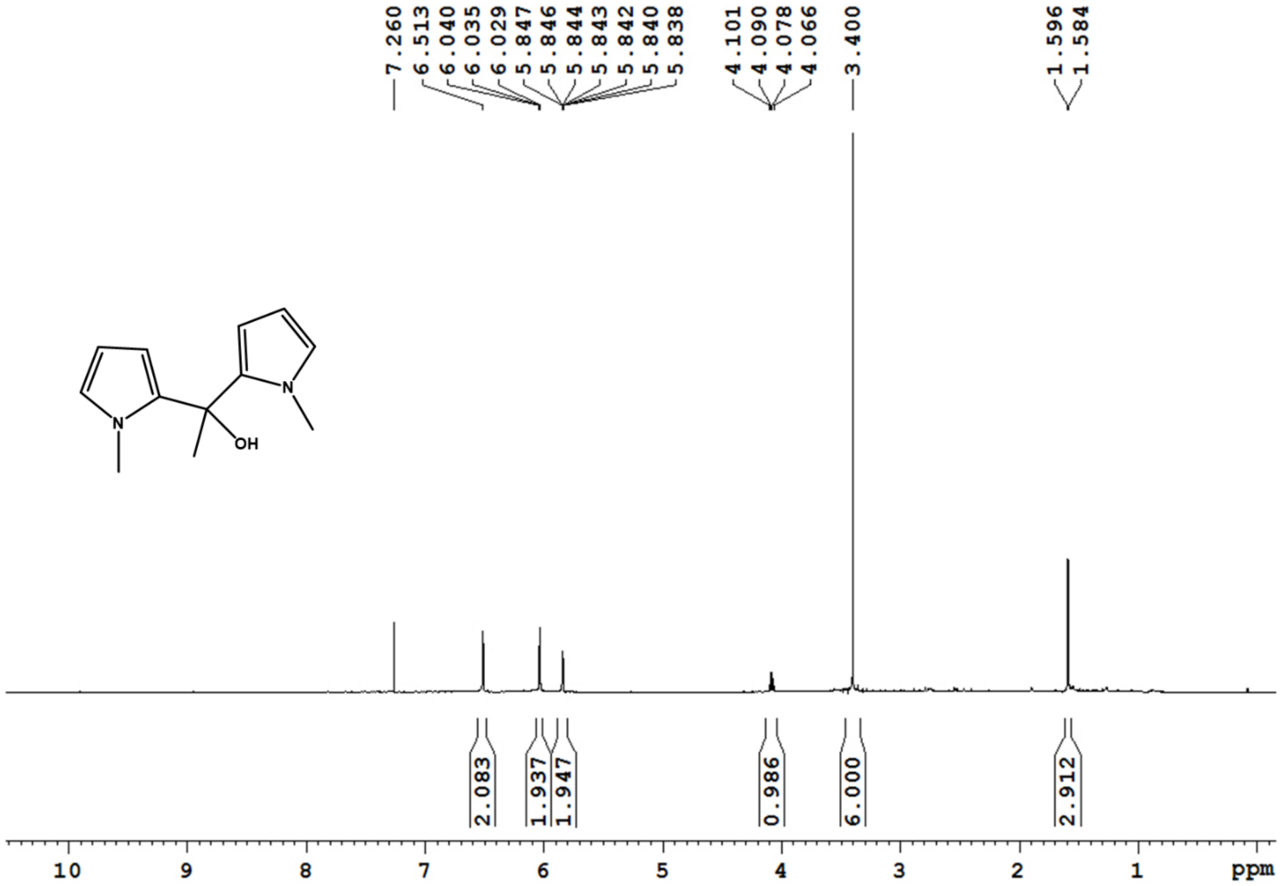

Figure S40. ^{1}H NMR (600 MHz, $CDCl_3$, 298 K) of the decarboxylate derivative.

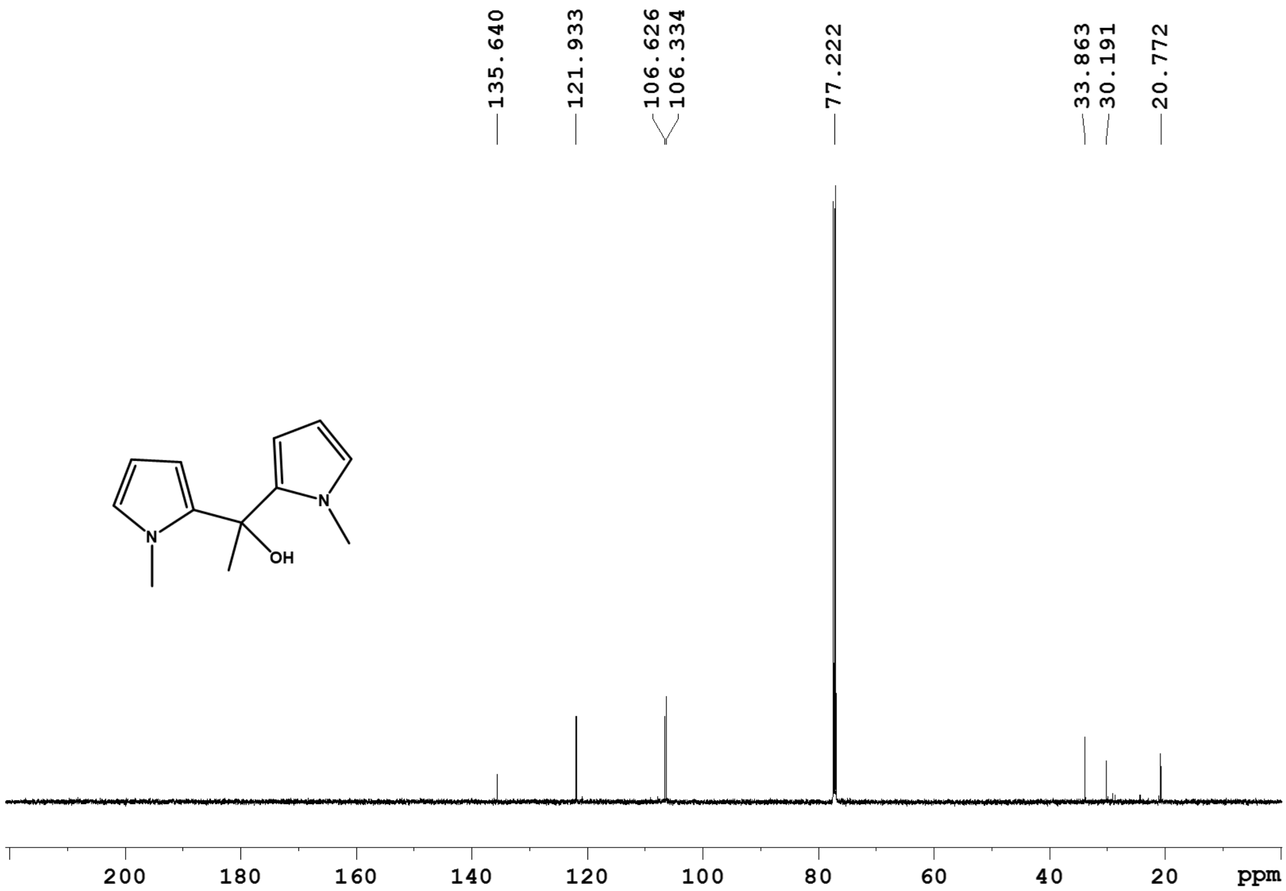

Figure S41. ^{13}C NMR (600 MHz, $CDCl_3$, 298 K) of the decarboxylate derivative.

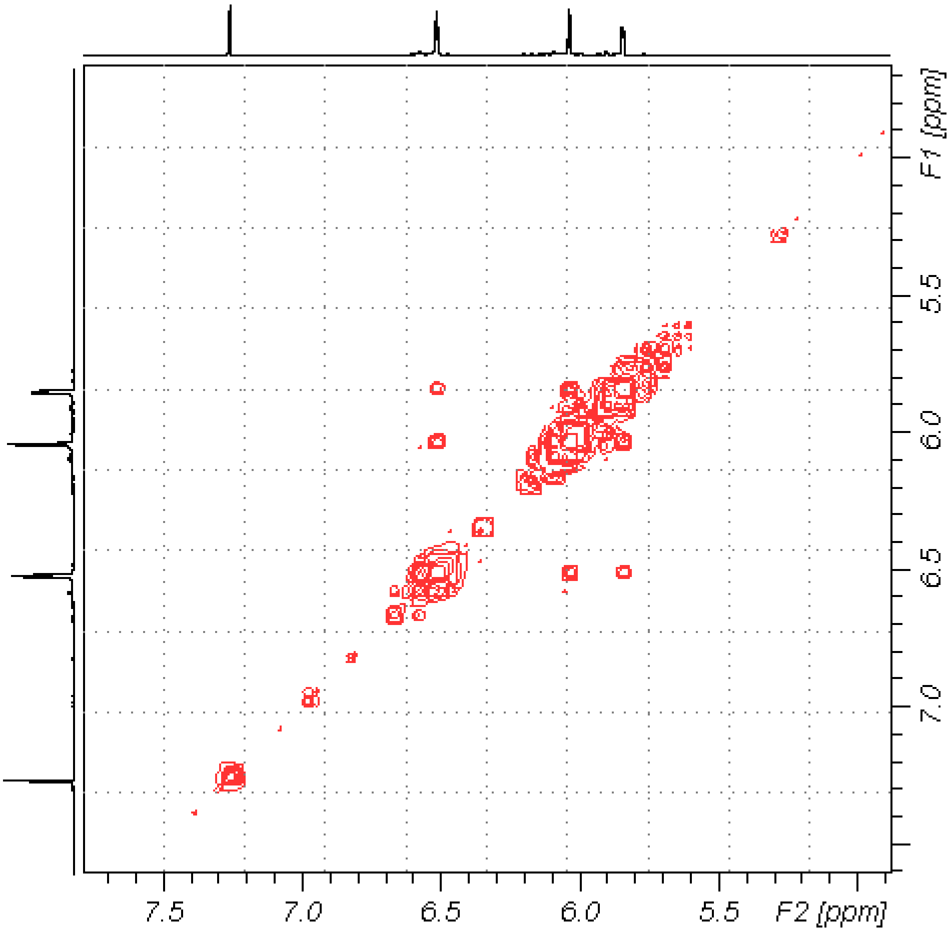

Figure S42. COSY NMR Spectrum (600 MHz, $CDCl_3$, 298 K) of the decarboxylate derivative.

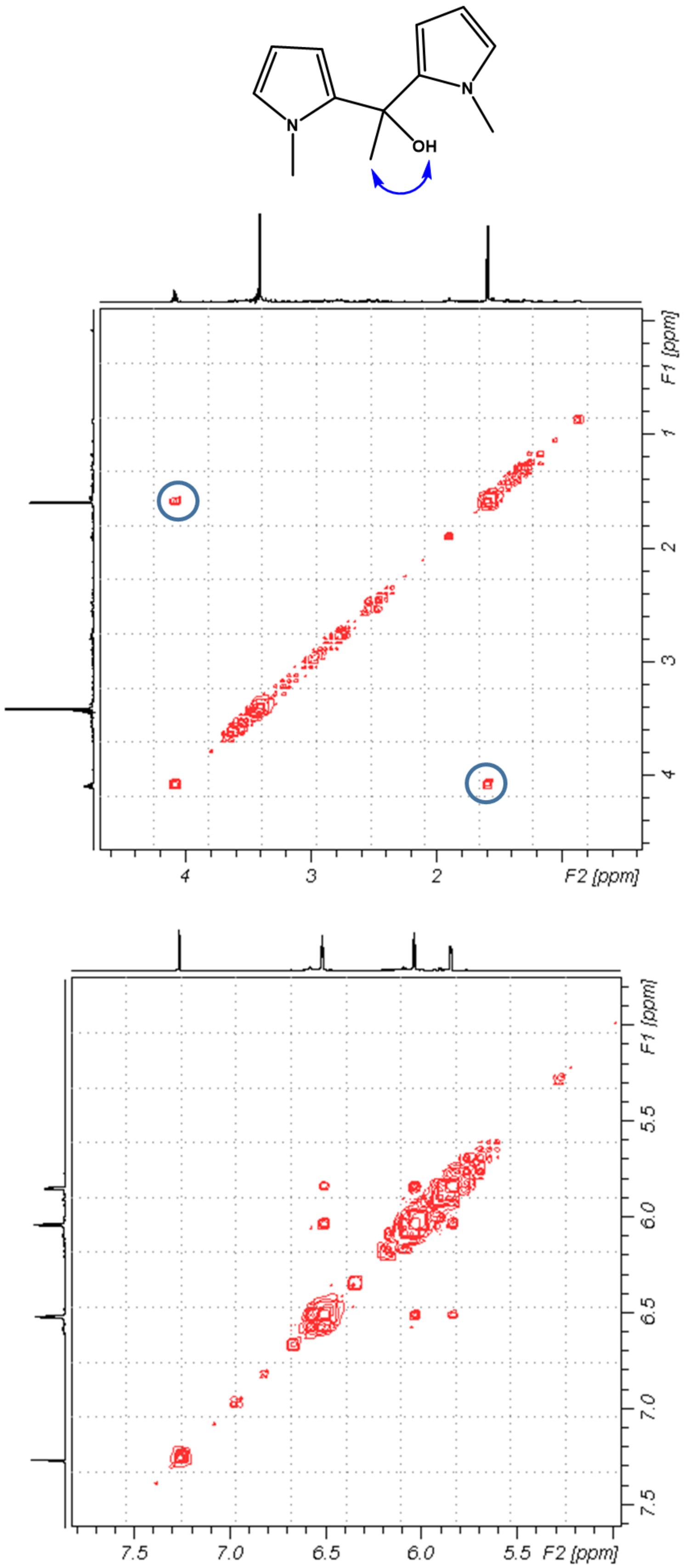

Figure S43. Expansions of COSY NMR Spectrum (600 MHz, $CDCl_3$, 298 K) of the decarboxylate derivative.

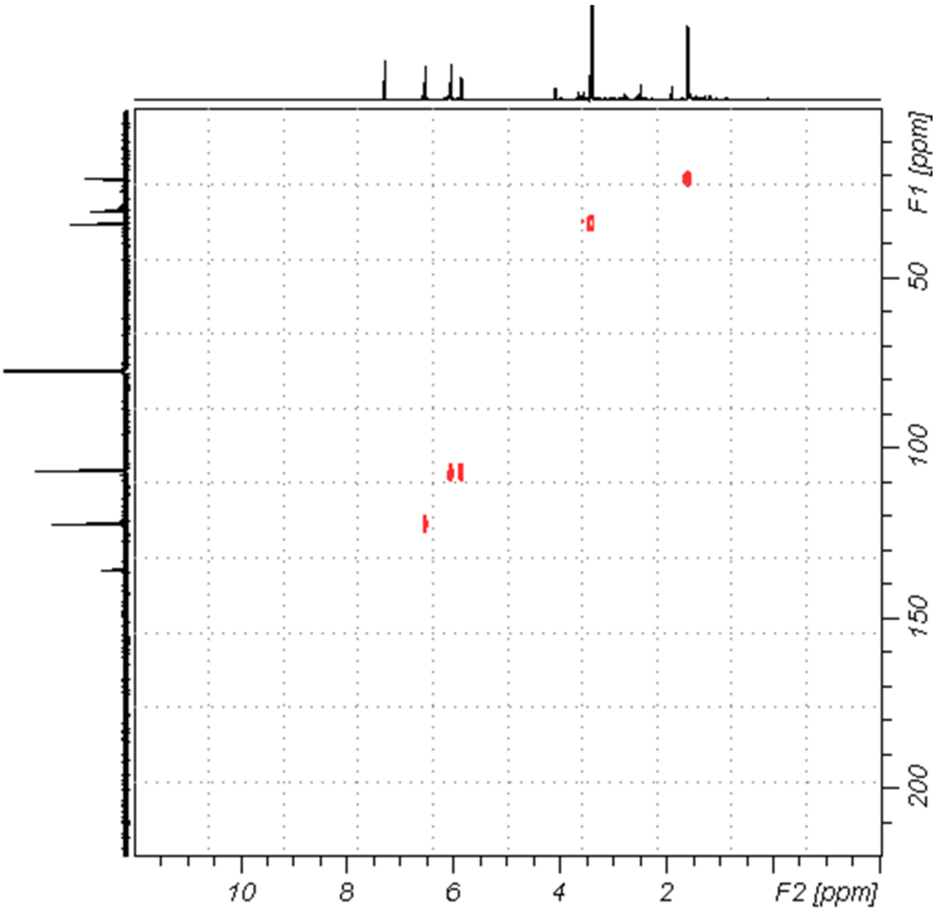

Figure S44. HSQC NMR Spectrum (600 MHz, $CDCl_3$, 298 K) of the decarboxylate derivative.

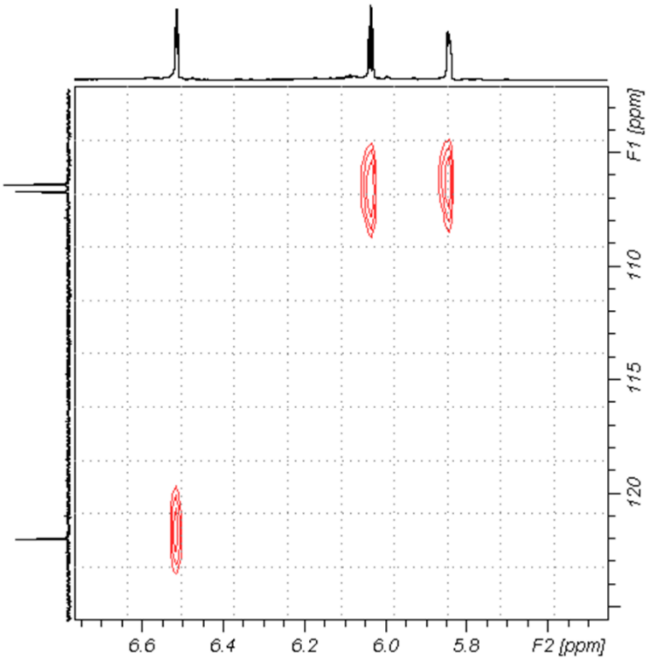

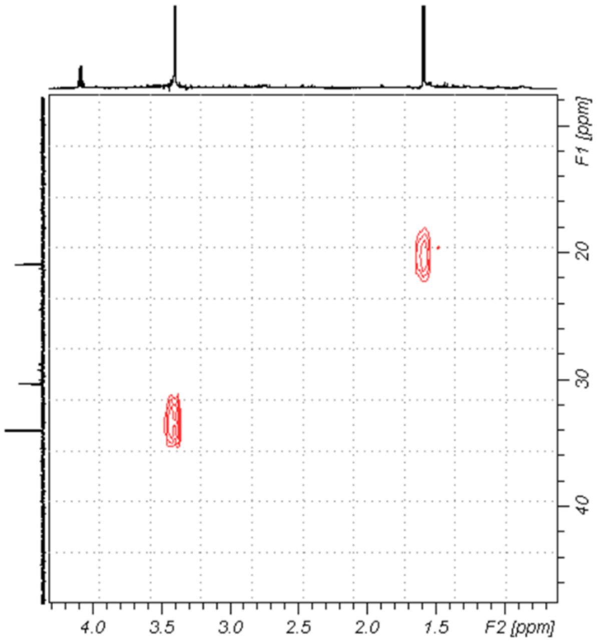

Figure S45. Expansions of HSQC NMR Spectrum (600 MHz, $CDCl_3$, 298 K) of the decarboxylate derivative.

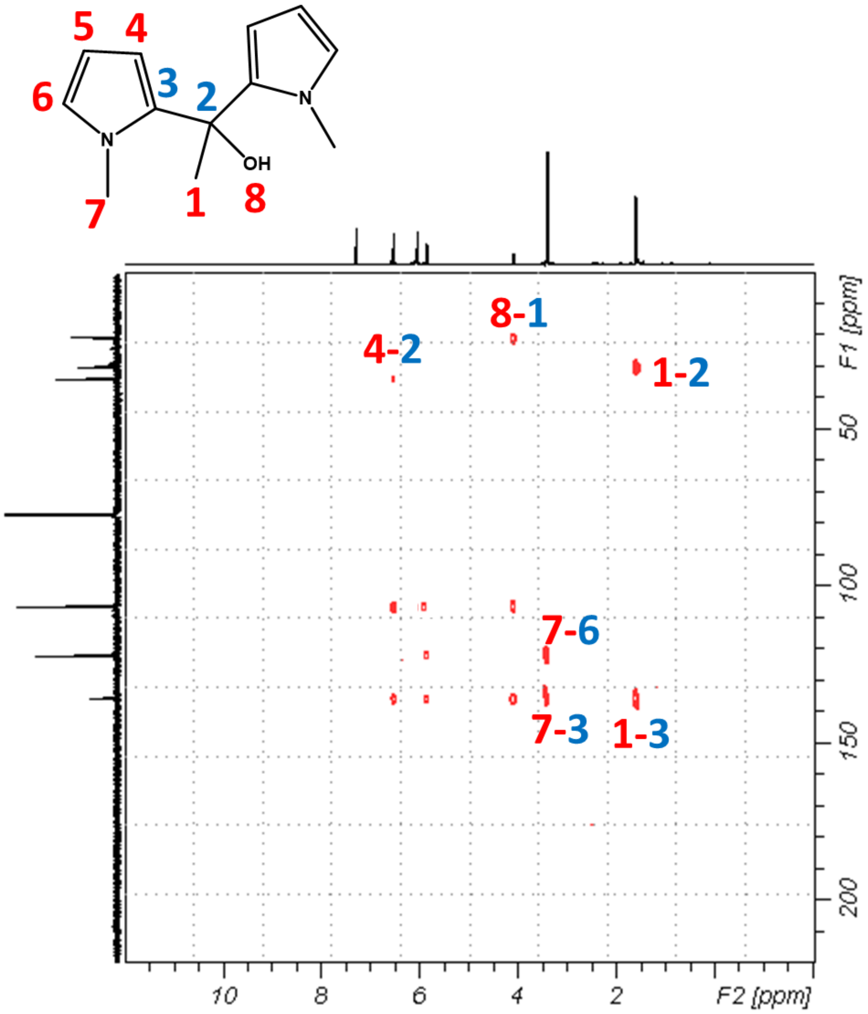

Figure S46. HMBC NMR Spectrum (600 MHz, $CDCl_3$, 298 K) of the decarboxylate derivative.

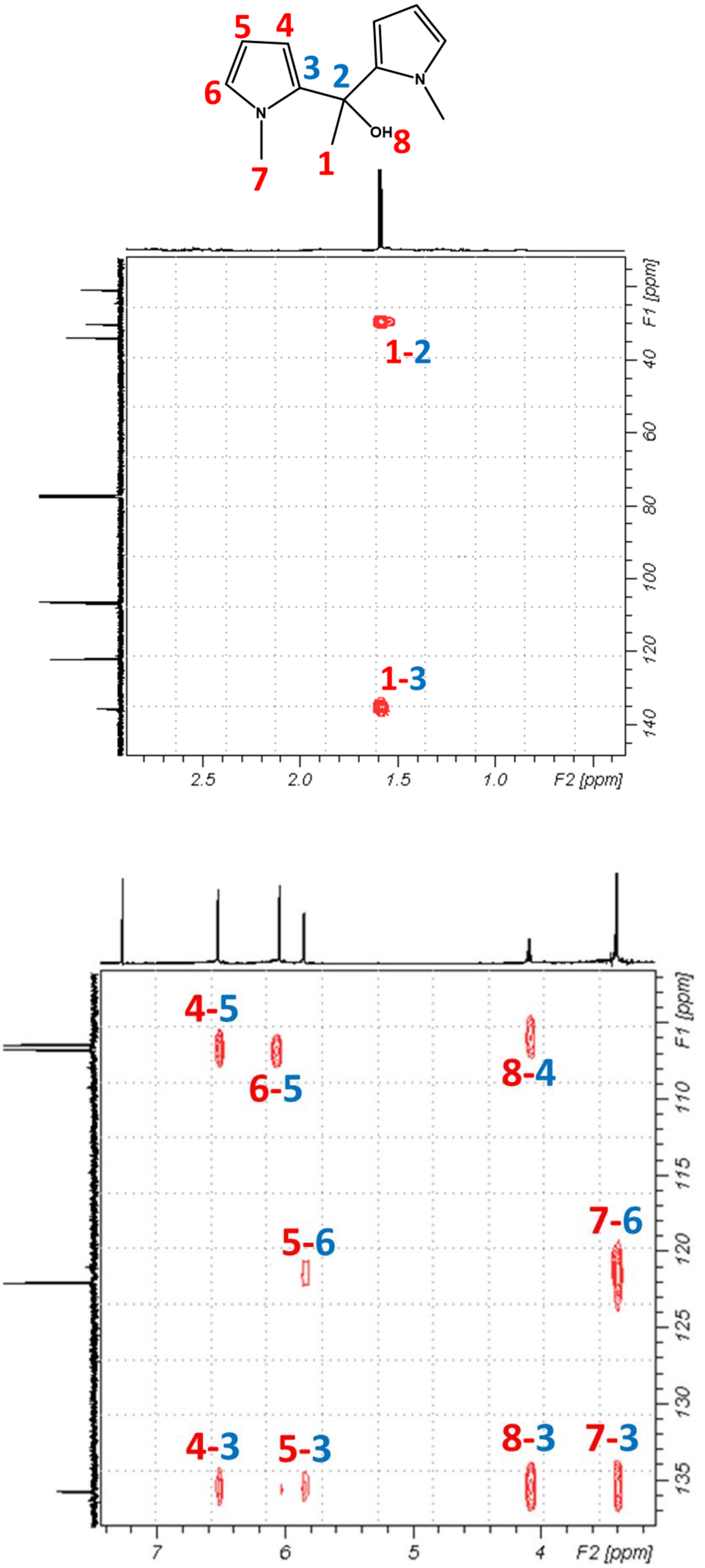

Figure S47. Expansions of HMBC NMR Spectrum (600 MHz, $CDCl_3$, 298 K) of the decarboxylate derivative.

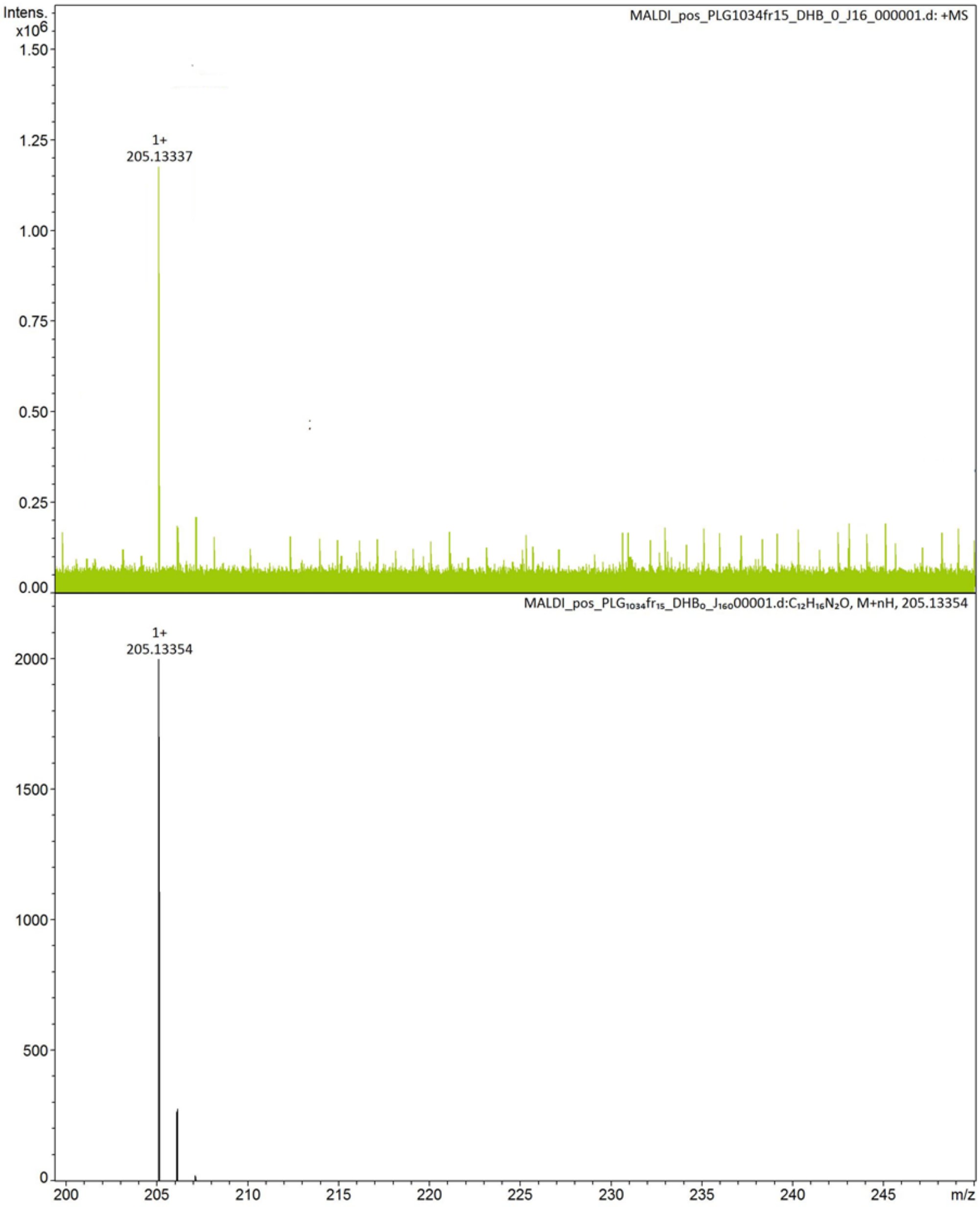

Figure S48. MALDI Mass Spectrum of the decarboxylate derivative.

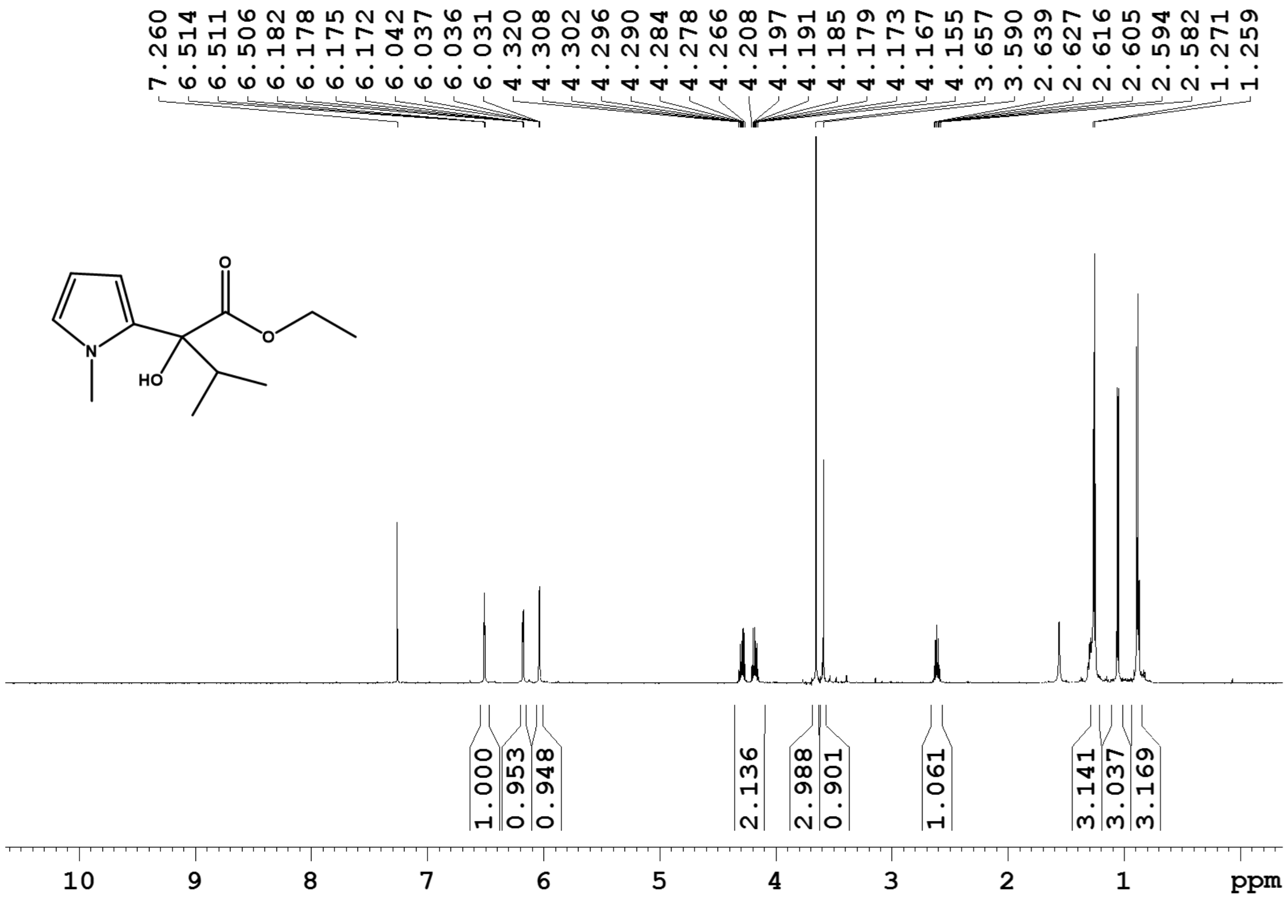

Figure S49. ^{1}H NMR Spectrum (600 MHz, $CDCl_3$, 298 K) of the derivative **6ac.**

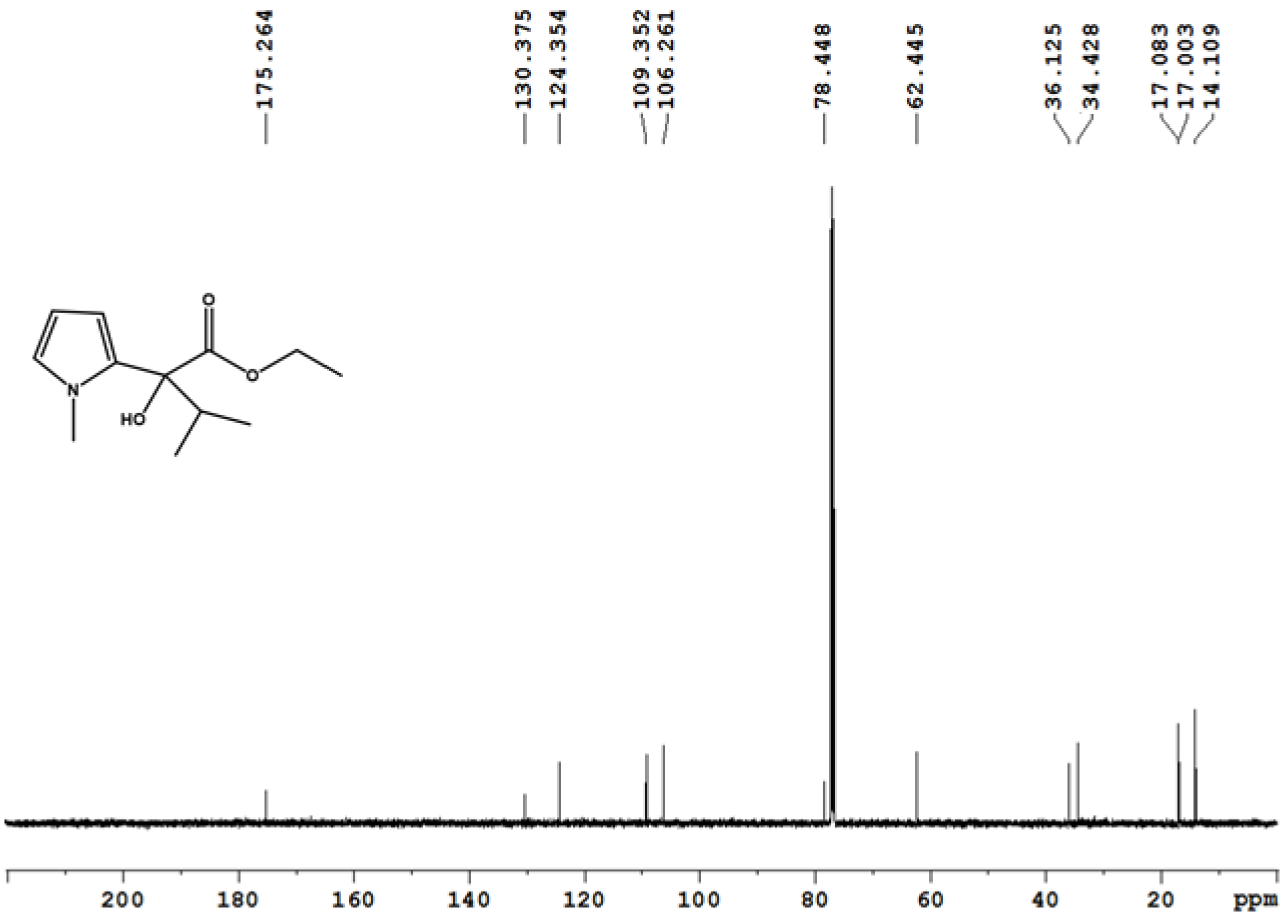

Figure S50. ^{13}C NMR Spectrum (150 MHz, $CDCl_3$, 298 K) of the derivative **6ac.**

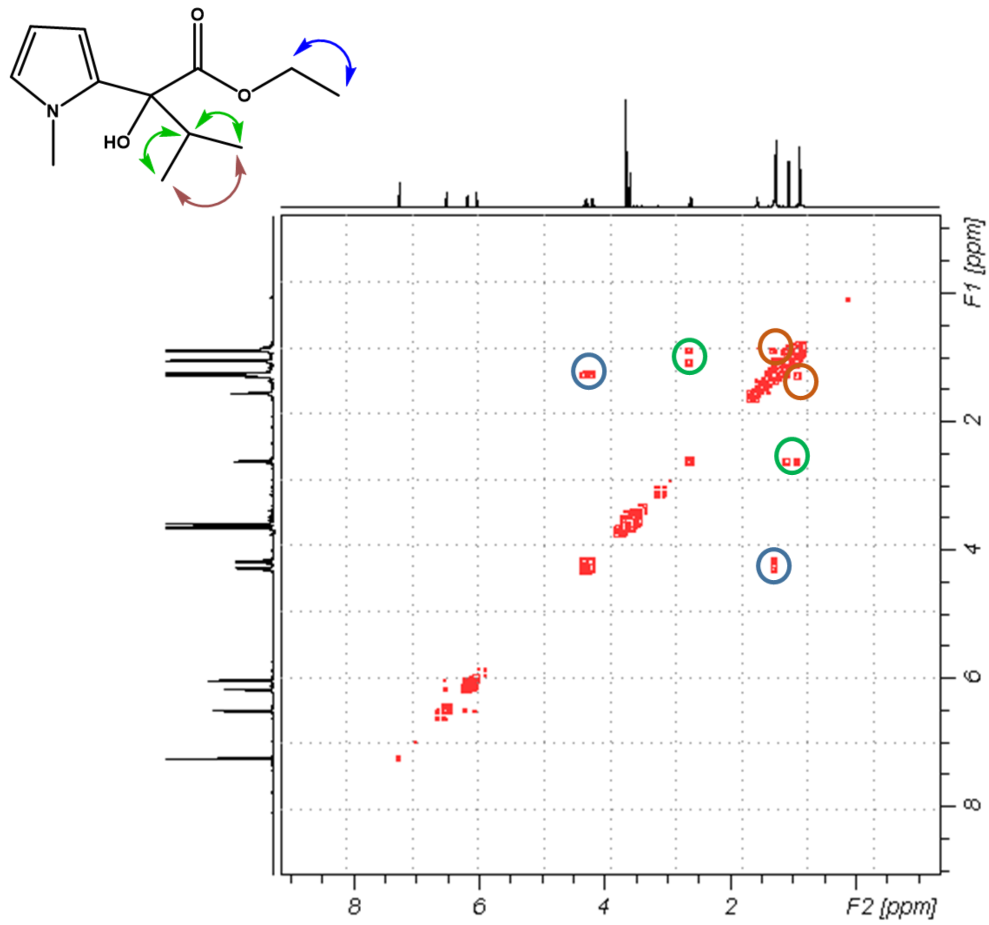

Figure S51. COSY NMR Spectrum (600 MHz, $CDCl_3$, 298 K) of the derivative **6ac**.

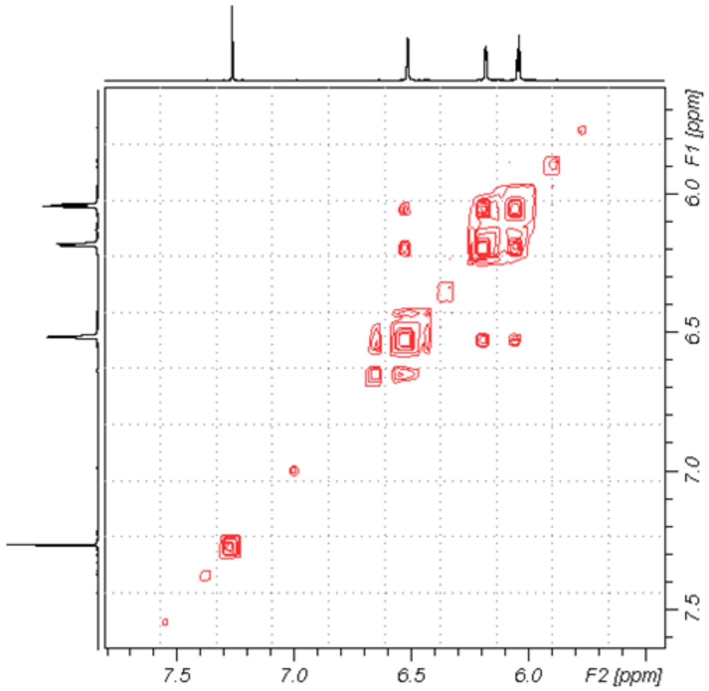

Figure S52. Expansion of COSY NMR Spectrum (600 MHz, $CDCl_3$, 298 K) of the derivative **6ac**.

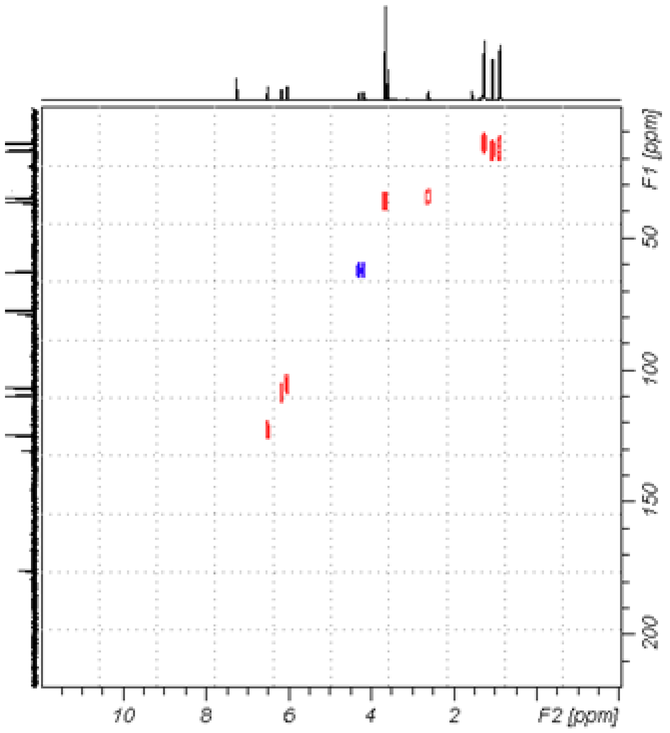

Figure S53. HSQC NMR Spectrum (600 MHz, $CDCl_3$, 298 K) of the derivative **6ac.**

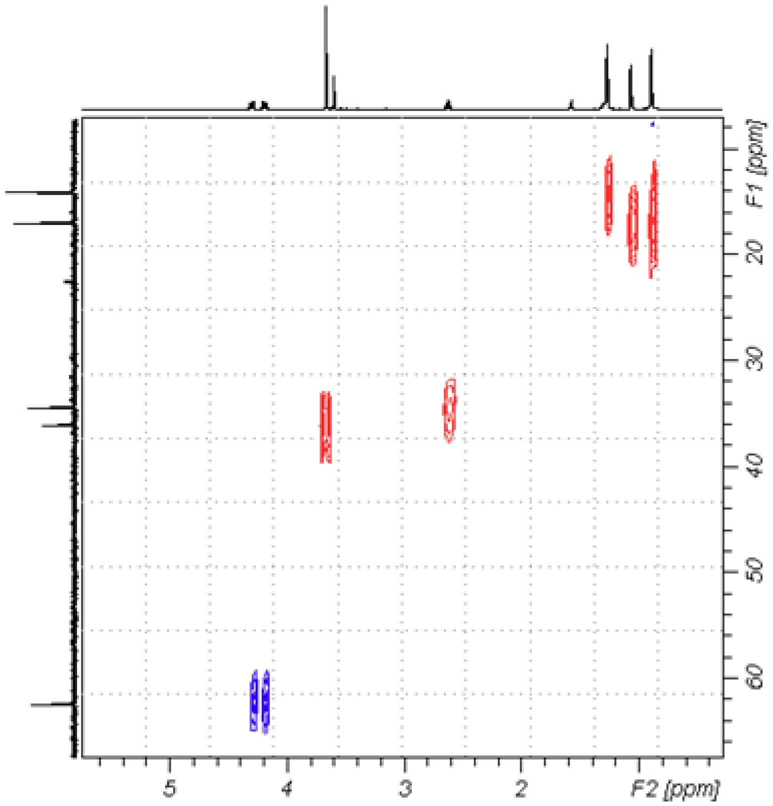

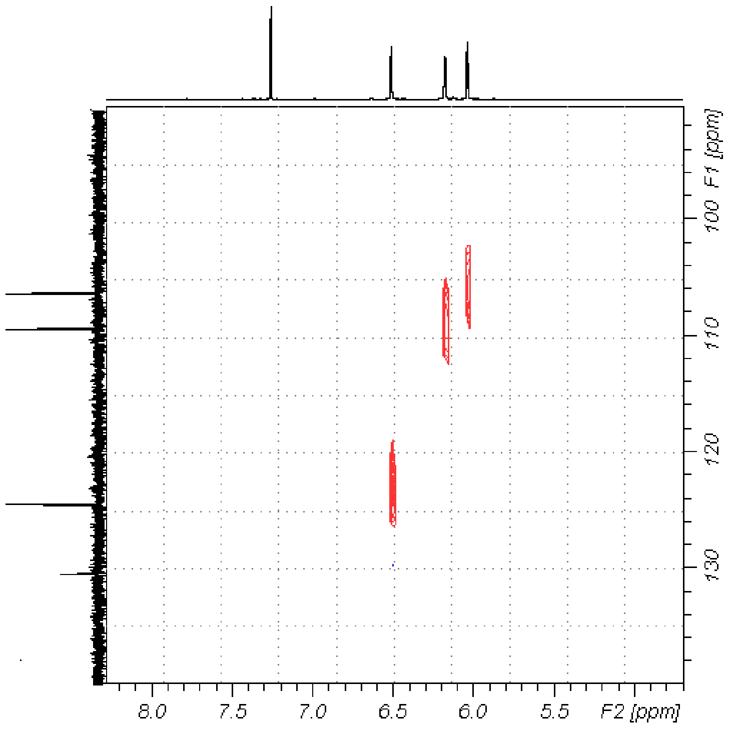

Figure S54. Expansions of HSQC NMR Spectrum (600 MHz, $CDCl_3$, 298 K) of the derivative **6ac**.

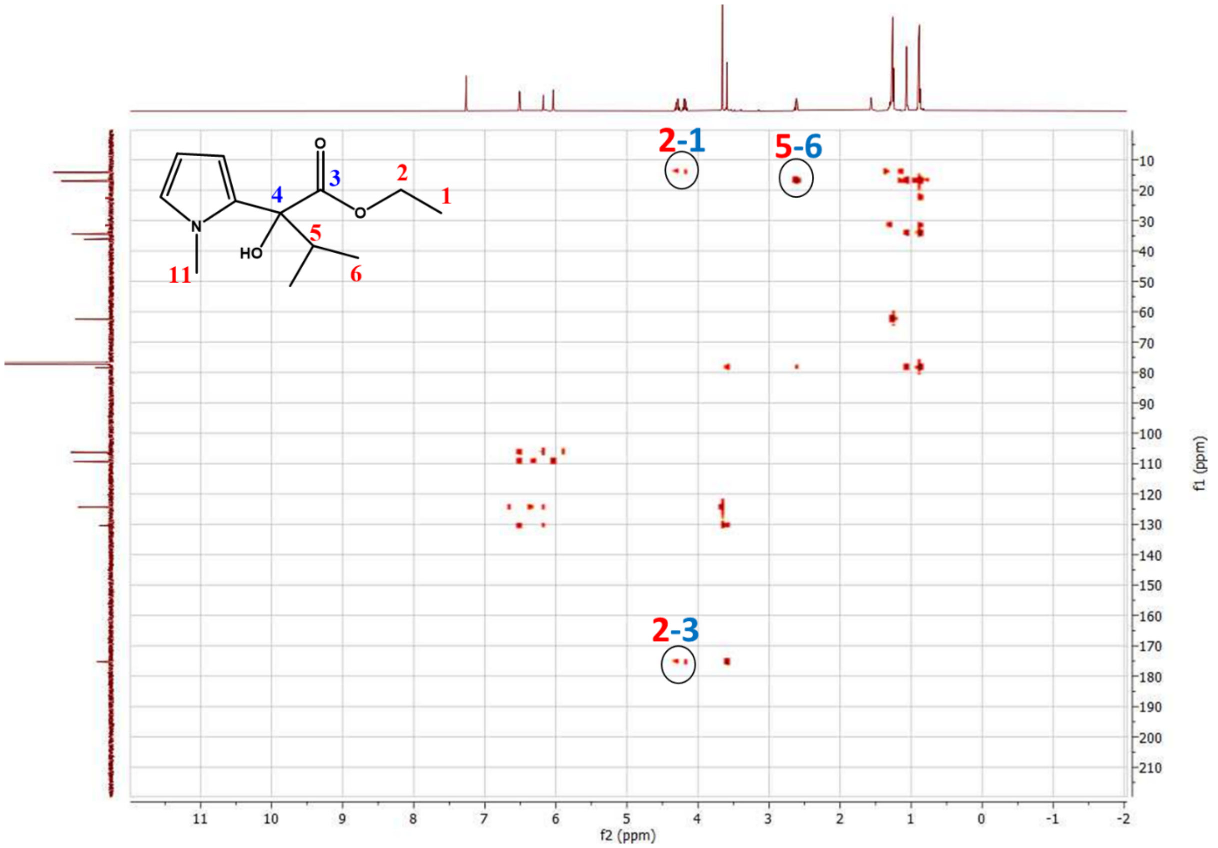

Figure S55. HMBC NMR Spectrum (600 MHz, $CDCl_3$, 298 K) of the derivative **6ac**.

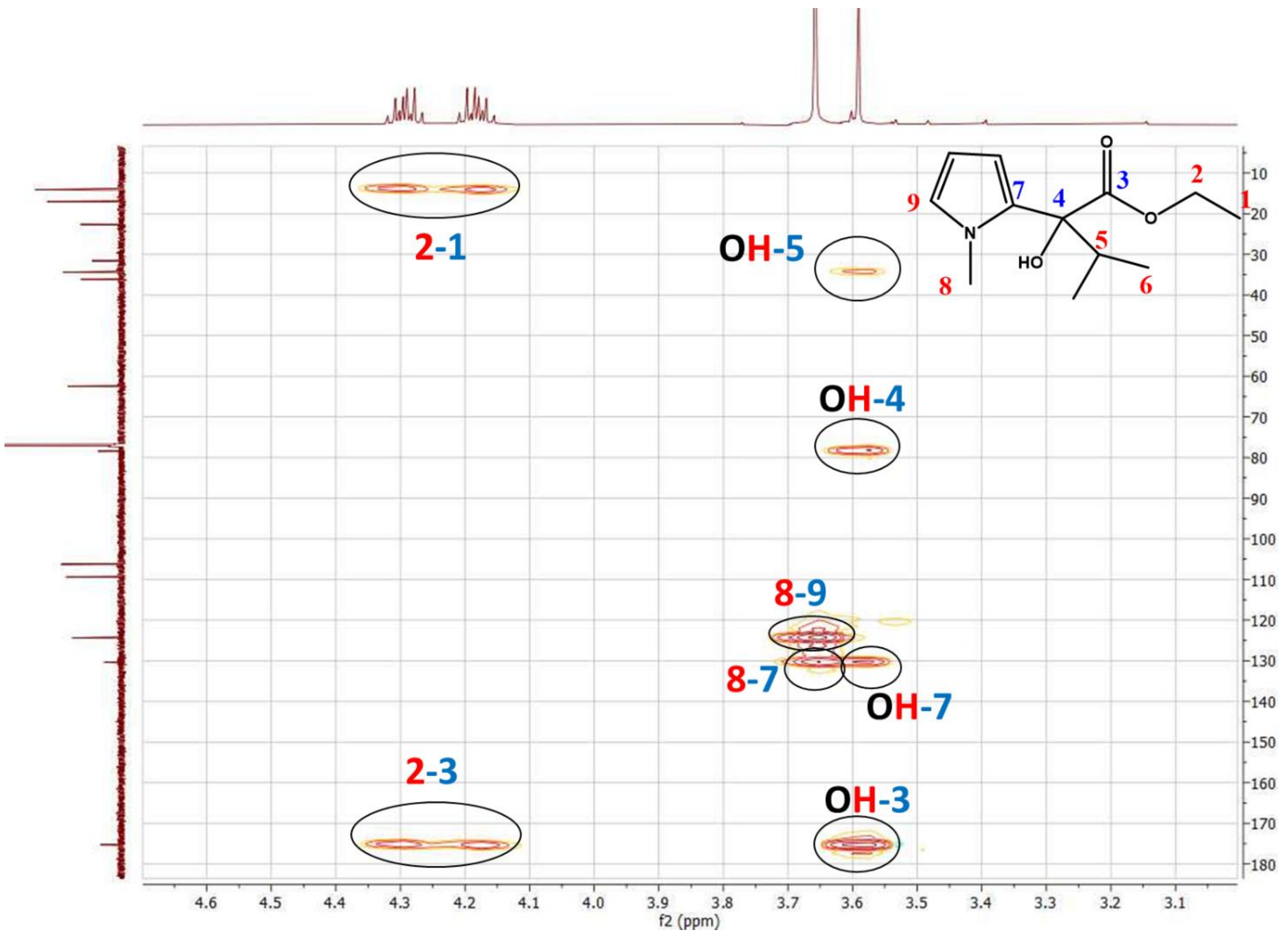

Figure S56. Expansion of the HMBC NMR Spectrum (600 MHz, $CDCl_3$, 298 K) of the derivative **6ac**.

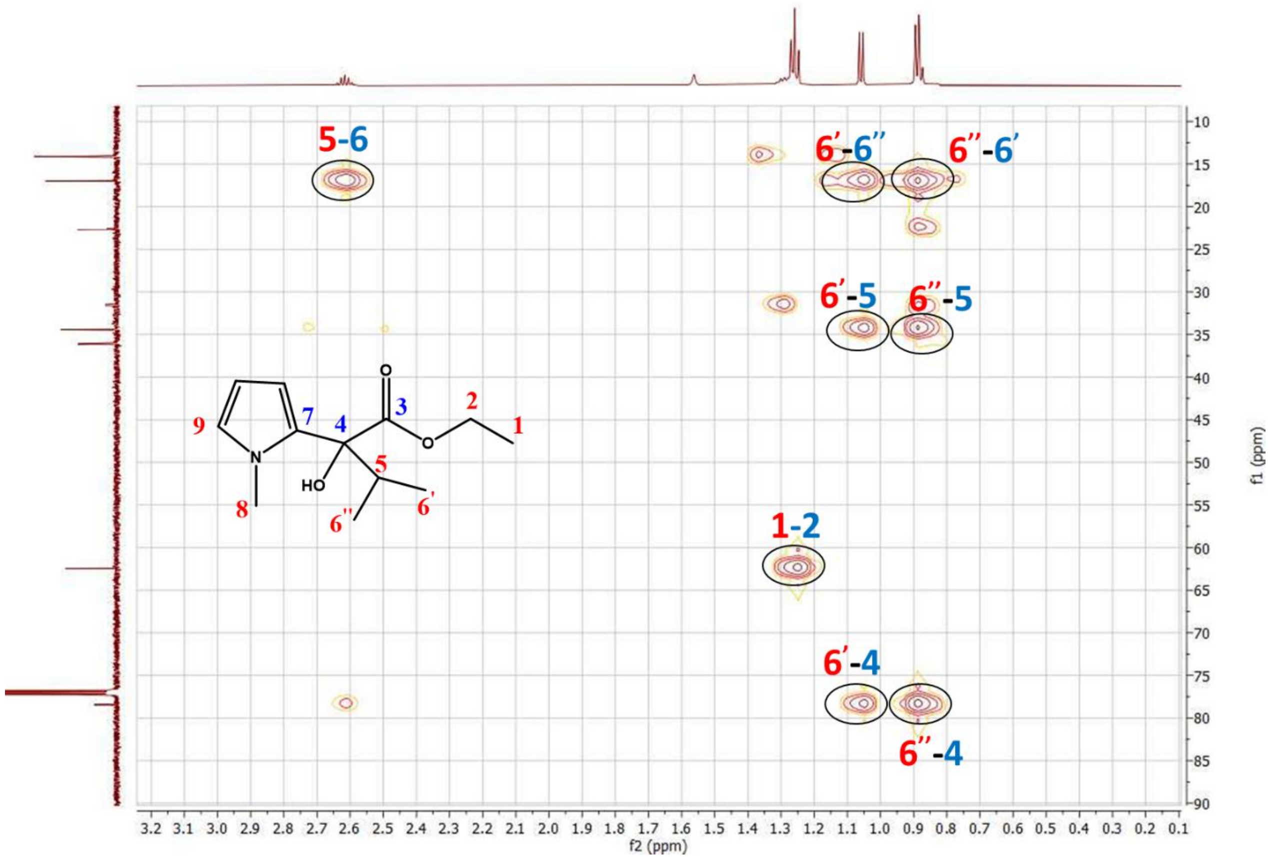

Figure S57. Expansion of the HMBC NMR Spectrum (600 MHz, $CDCl_3$, 298 K) of the derivative **6ac.**

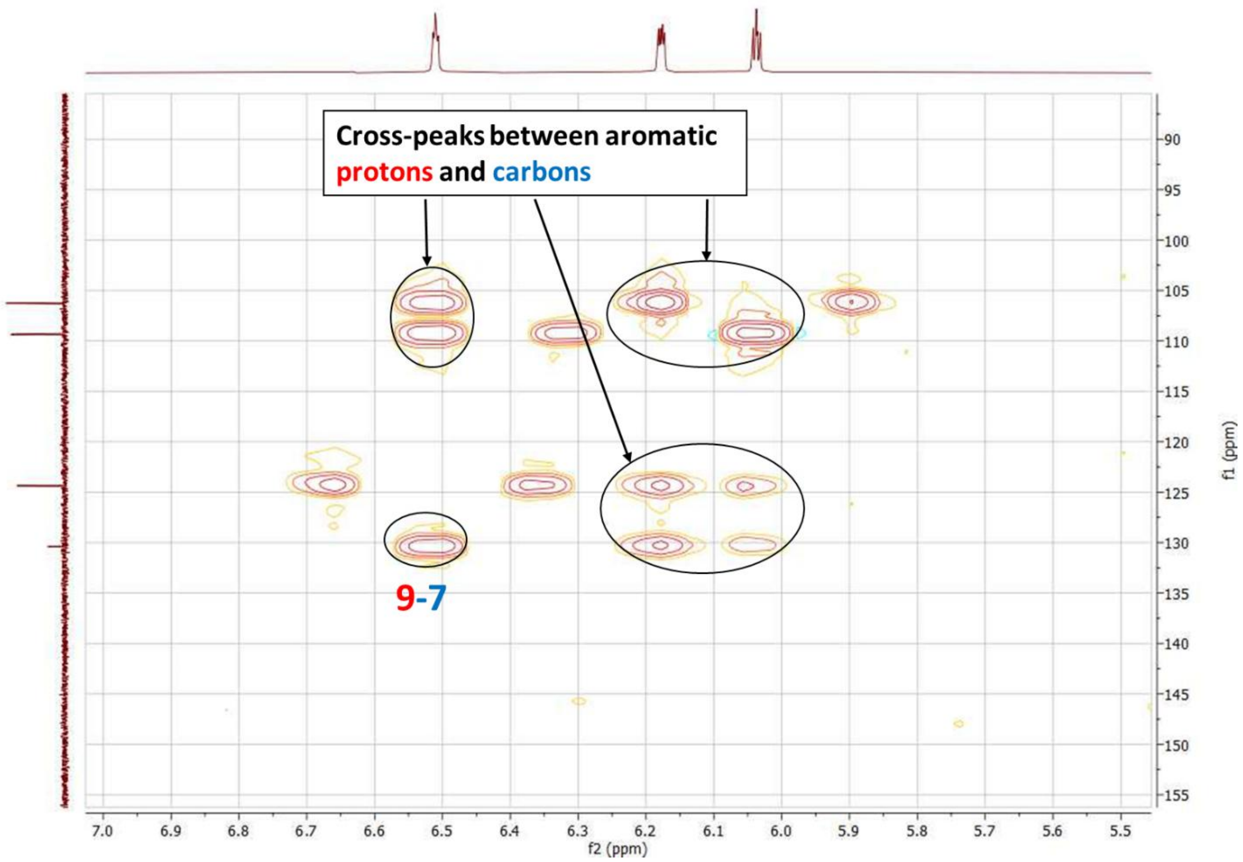

Figure S58. Expansion of the HMBC NMR Spectrum (600 MHz, $CDCl_3$, 298 K) of the derivative **6ac.**

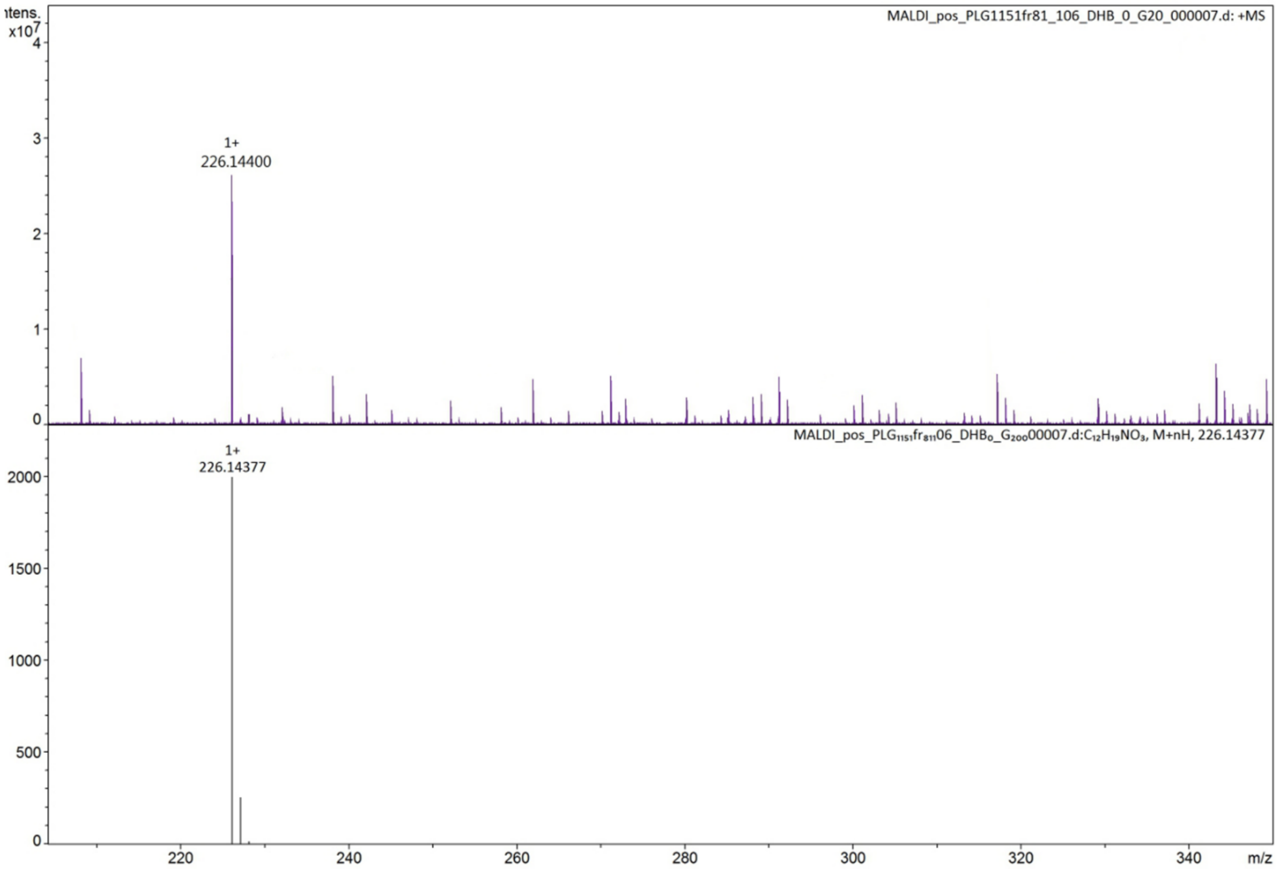

Figure S59: MALDI MS spectrum of the derivative **6ac**.

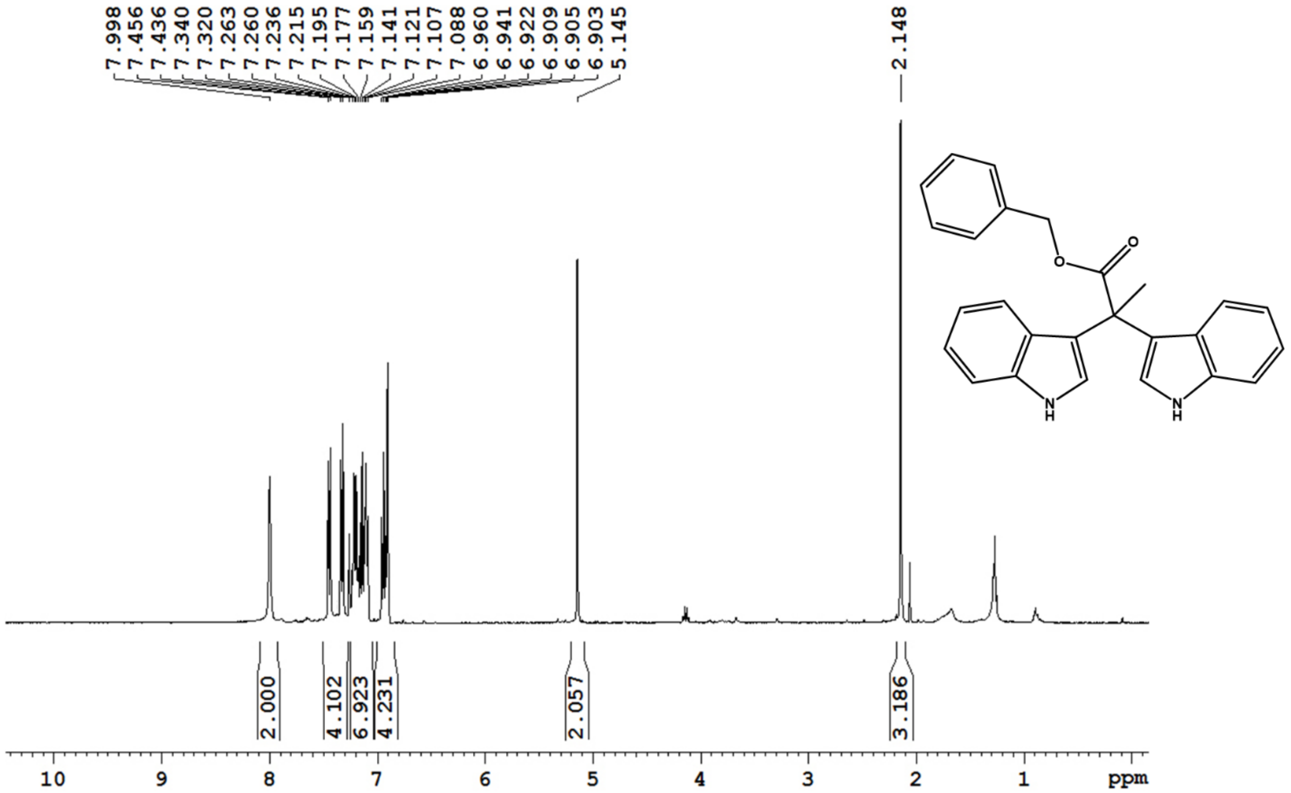

Figure S60. ^{1}H NMR (600 MHz, $CDCl_3$, 298 K) of the derivative **9ad.**

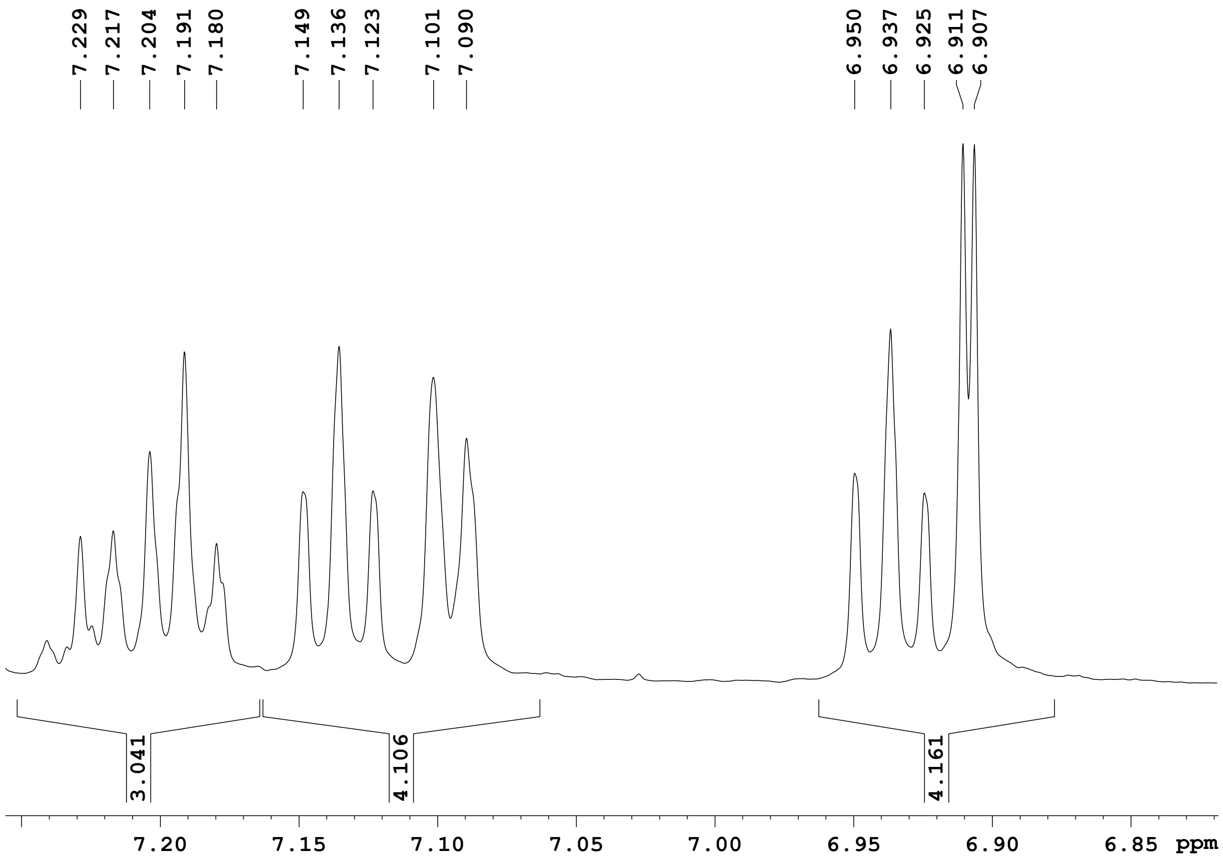

Figure S61. Relevant region comprised between 7.23 ppm nd 6.91 ppm. ^{1}H NMR (600 MHz, $CDCl_3$, 298 K) of the derivative **9ad.**

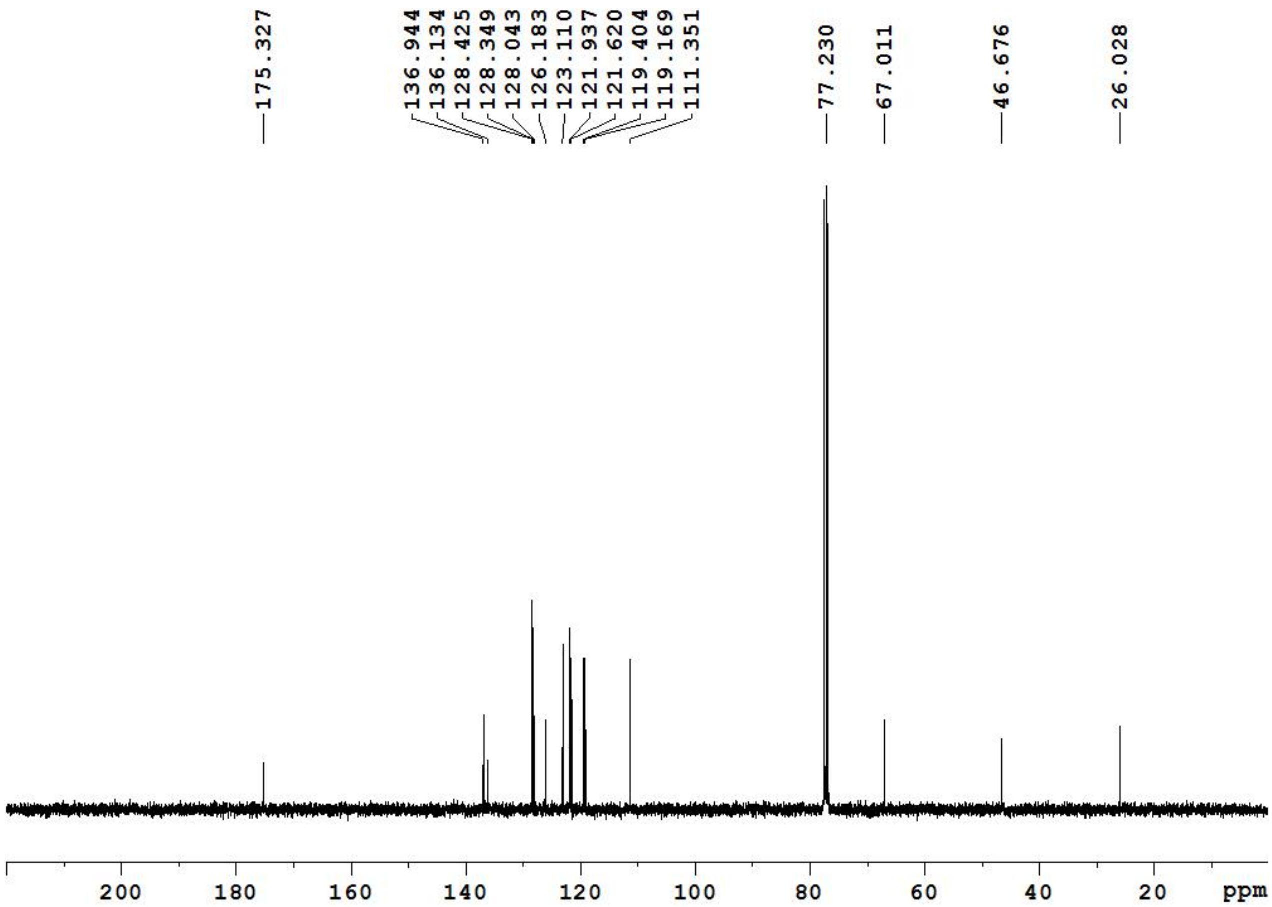

Figure S62. ^{13}C NMR (150 MHz, $CDCl_3$, 298 K) of the derivative **9ad**

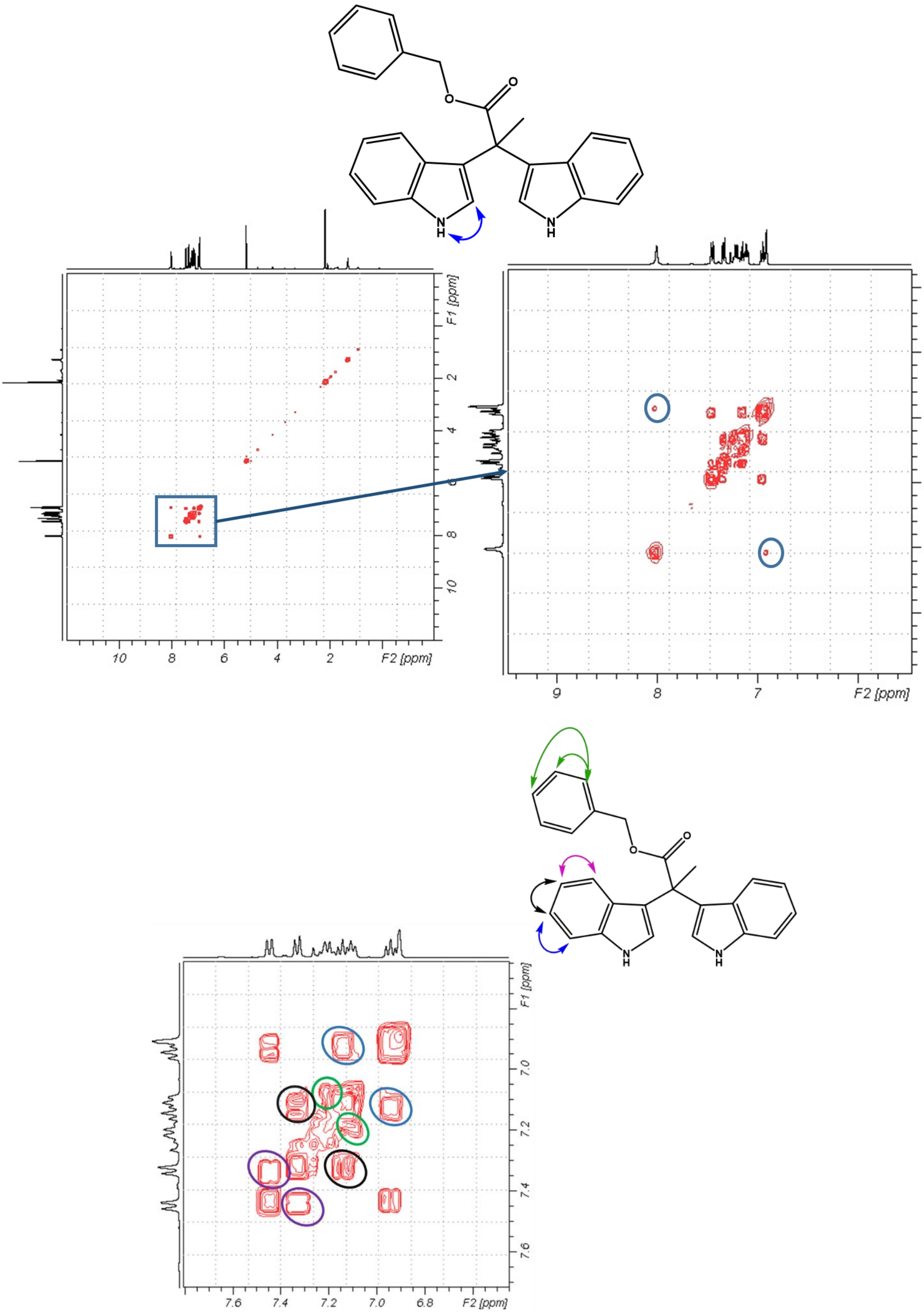

Figure S67. COSY NMR spectrum (400 MHz, $CDCl_3$, 298 K) of the derivative **9ad** with its relevant regions.

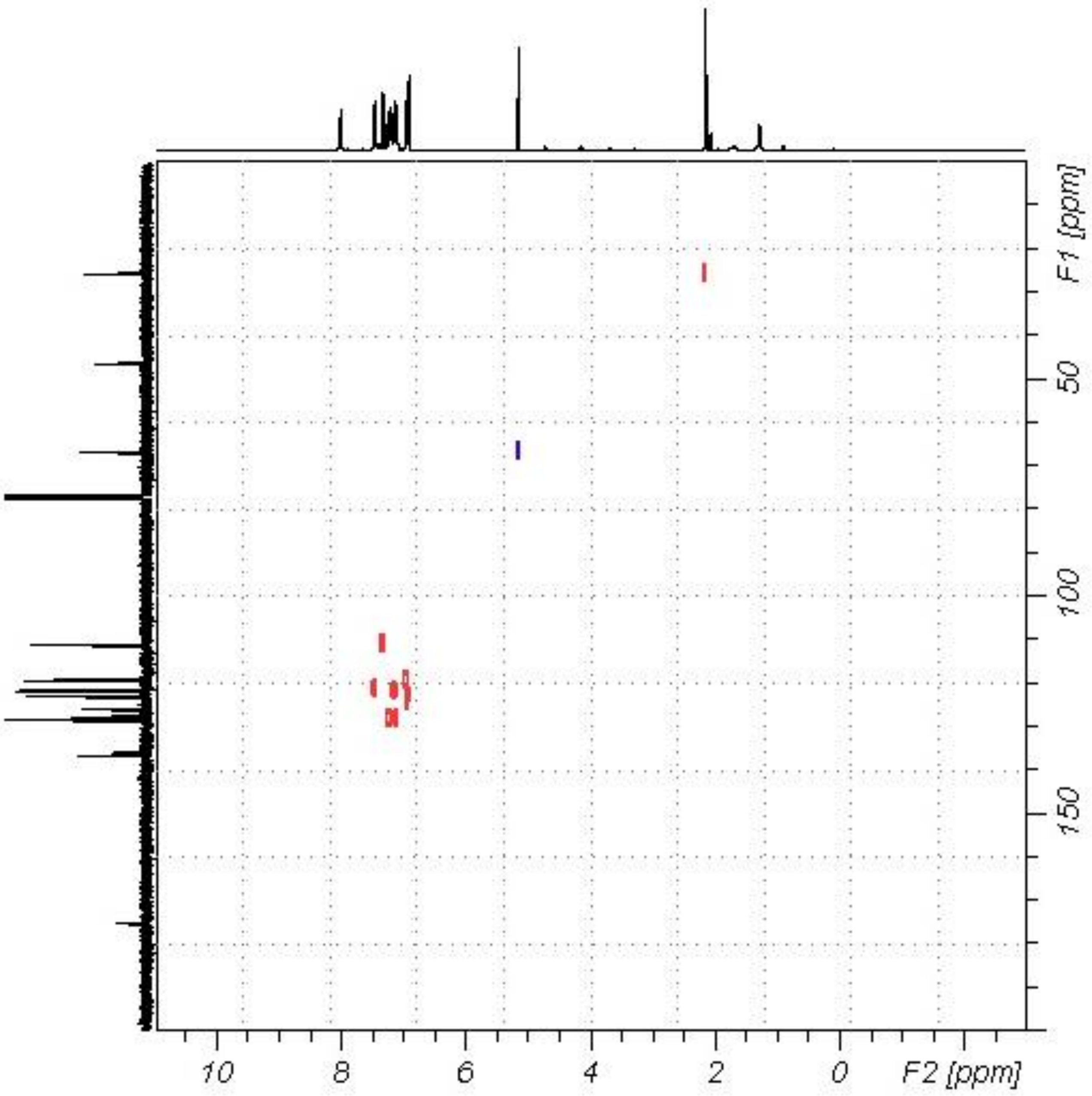

Figure S68. HSQC NMR spectrum (400 MHz, $CDCl_3$, 298 K) of the derivative **9ad.**

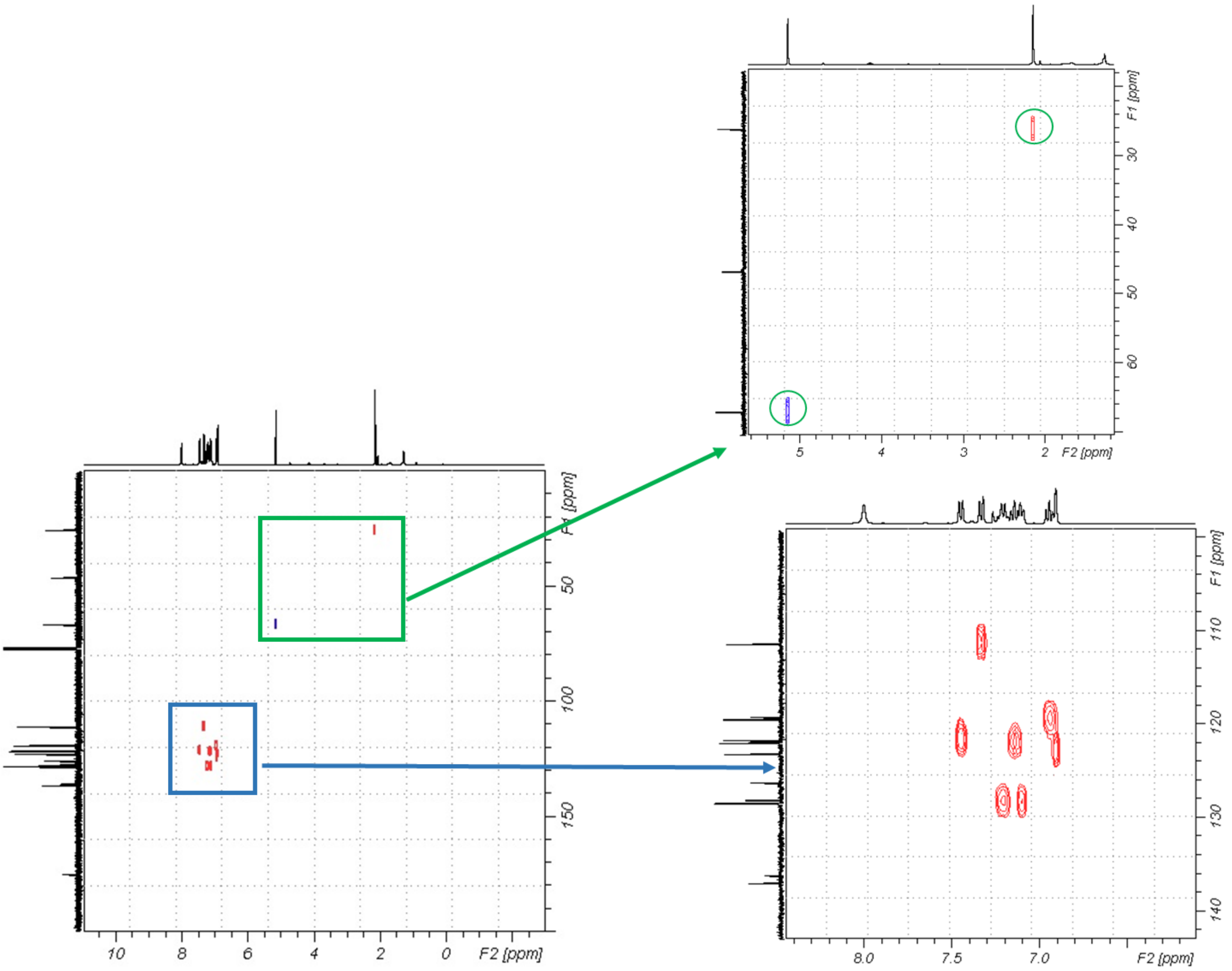

Figure S69. HSQC NMR spectrum (400 MHz, $CDCl_3$, 298 K) of the derivative **9ad** with expansions of the relevant regions. Blue spots are indicative of CH_2 protons, red sposts of CH and CH_3 protons.

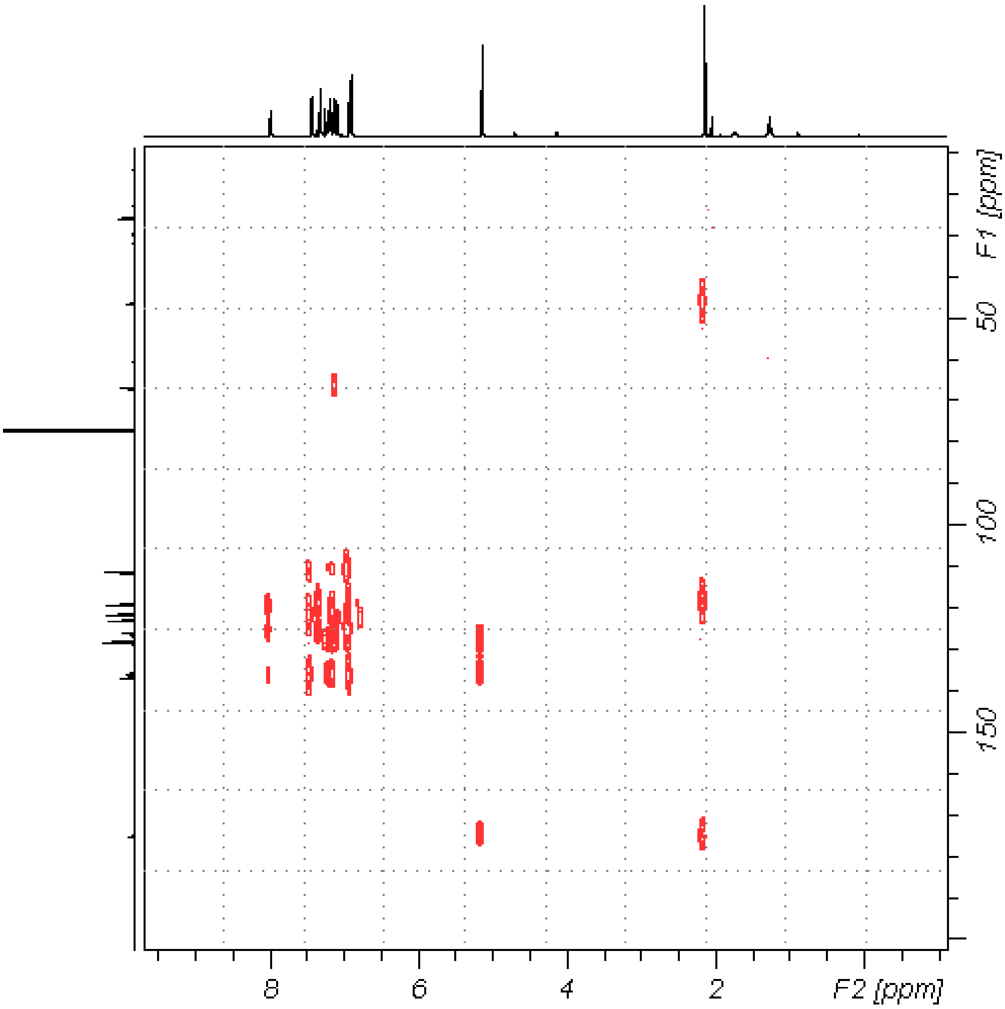

Figure S70. HMBC NMR spectrum (600 MHz, $CDCl_3$, 298 K) of the derivative **9ad.**

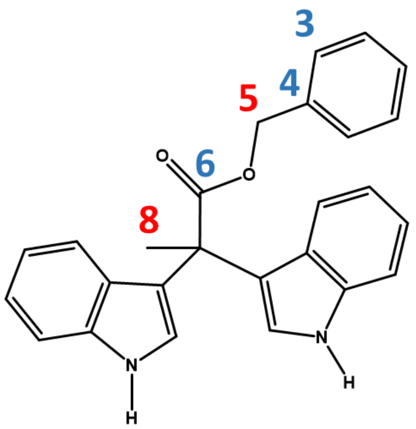
3
4
5
6
8
O
N
H

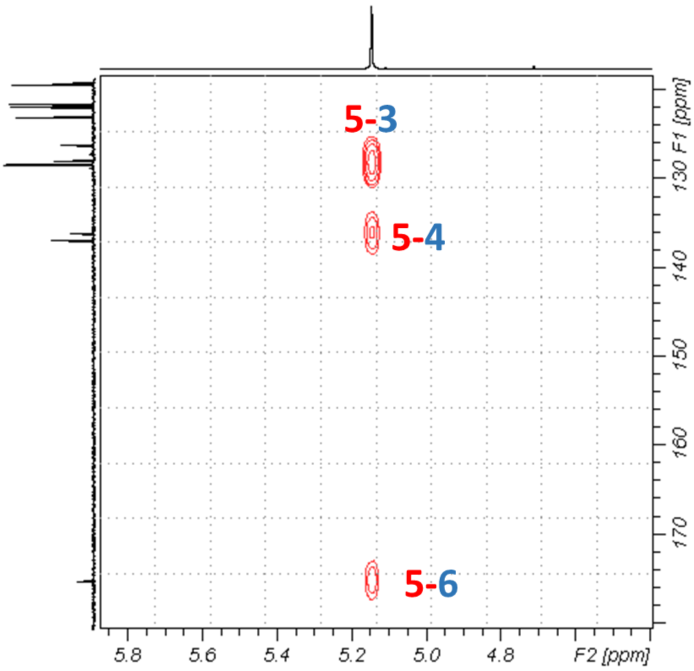
5-3
5-4
5-6
F1 [ppm]
130
140
150
160
170
5.8
5.6
5.4
5.2
5.0
4.8
F2 [ppm]

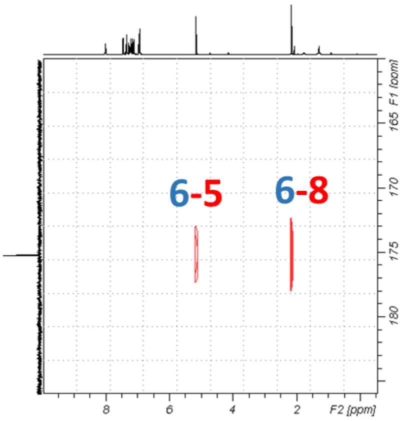
6-5
6-8
F1 (ppm)
165
170
175
180
8
6
4
2
F2 [ppm]

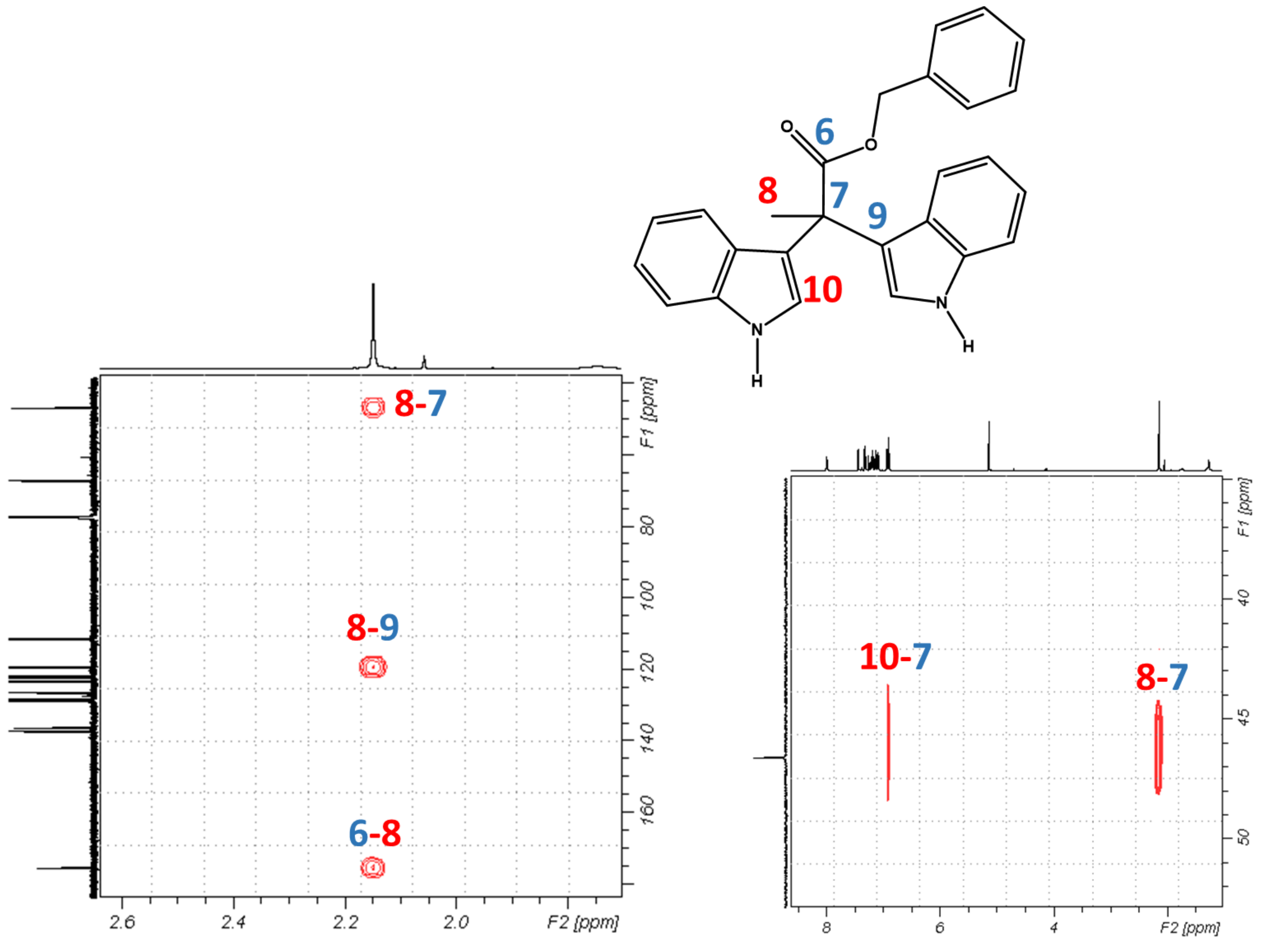
6
7
8
9
10
N
H
O
8-7
8-9
6-8
10-7
F1 [ppm]
F2 [ppm]

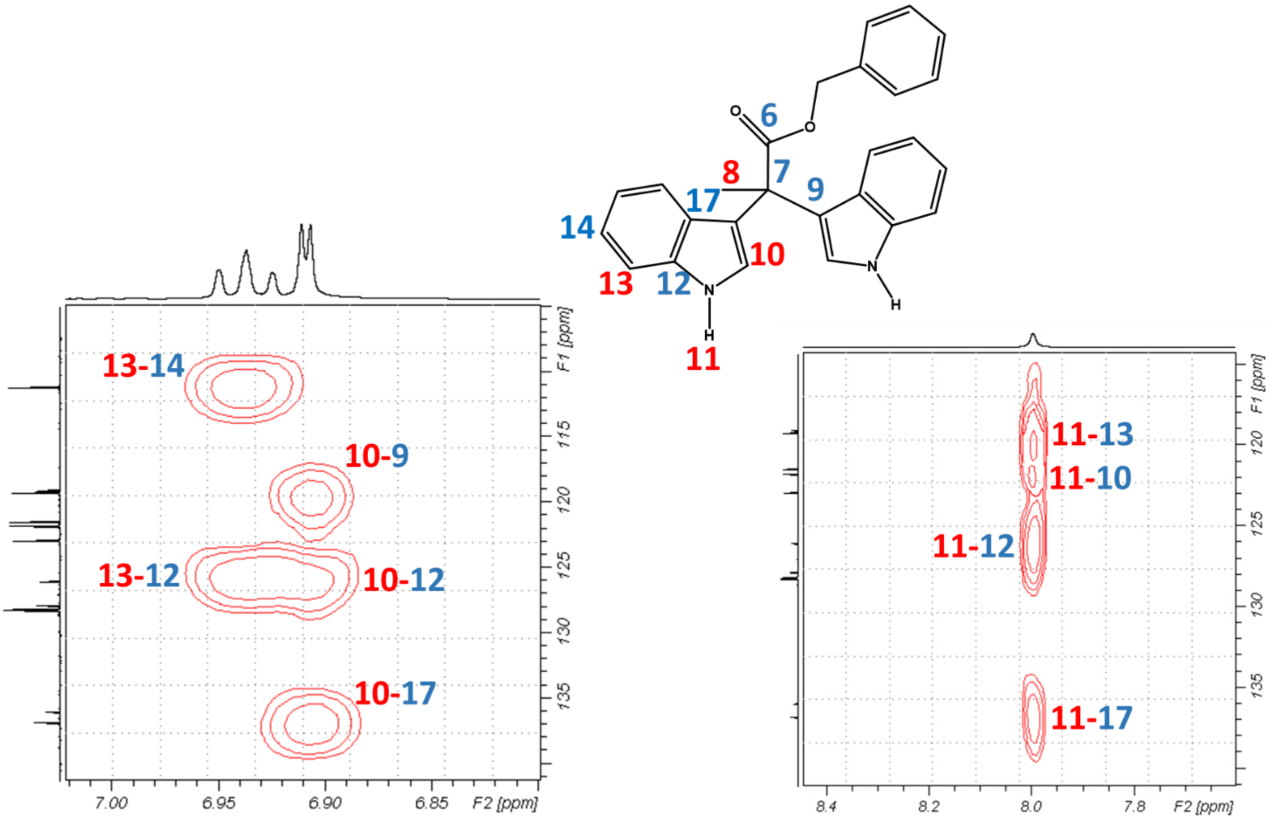
6
7
8
9
10
11
12
13
14
17
N
H
O
13-14
10-9
13-12
10-12
10-17
11-13
11-10
11-12
11-17
F1 [ppm]
F2 [ppm]

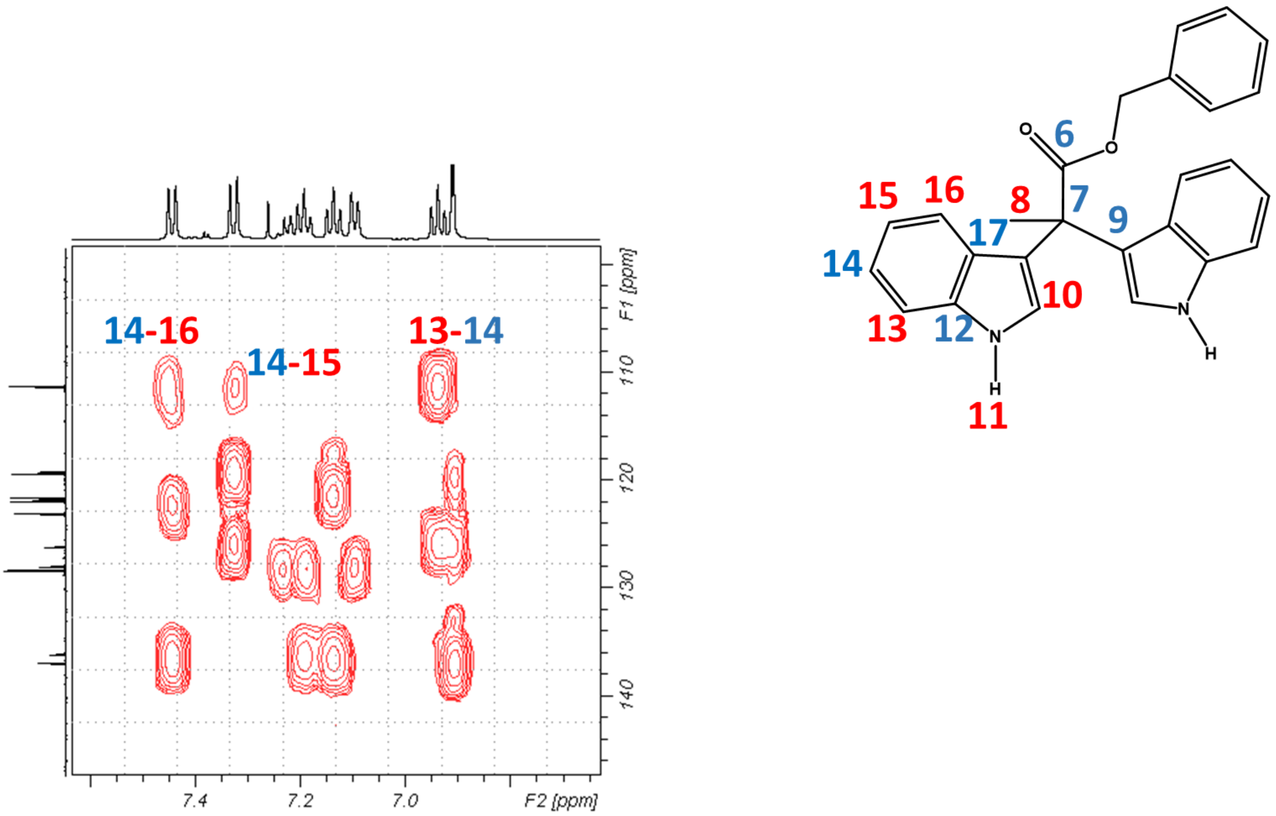

Figure S71: from above to down, expansions of relevant regions (with annotations) of HMBC NMR spectrum of derivative **9ad** (600 MHz, 298K, $CDCl_3$).

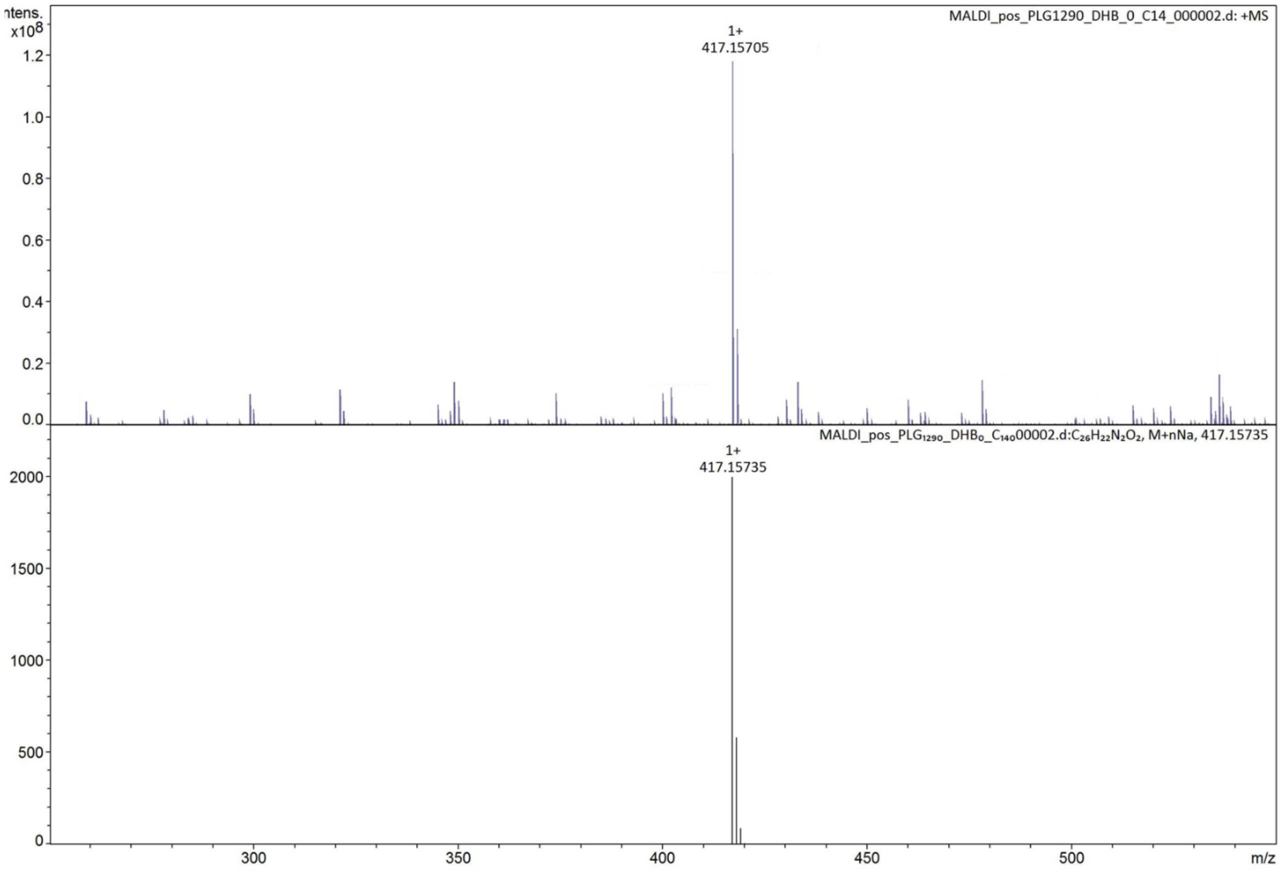

Figure S72: MALDI MS spectrum of the derivative **9ad.**

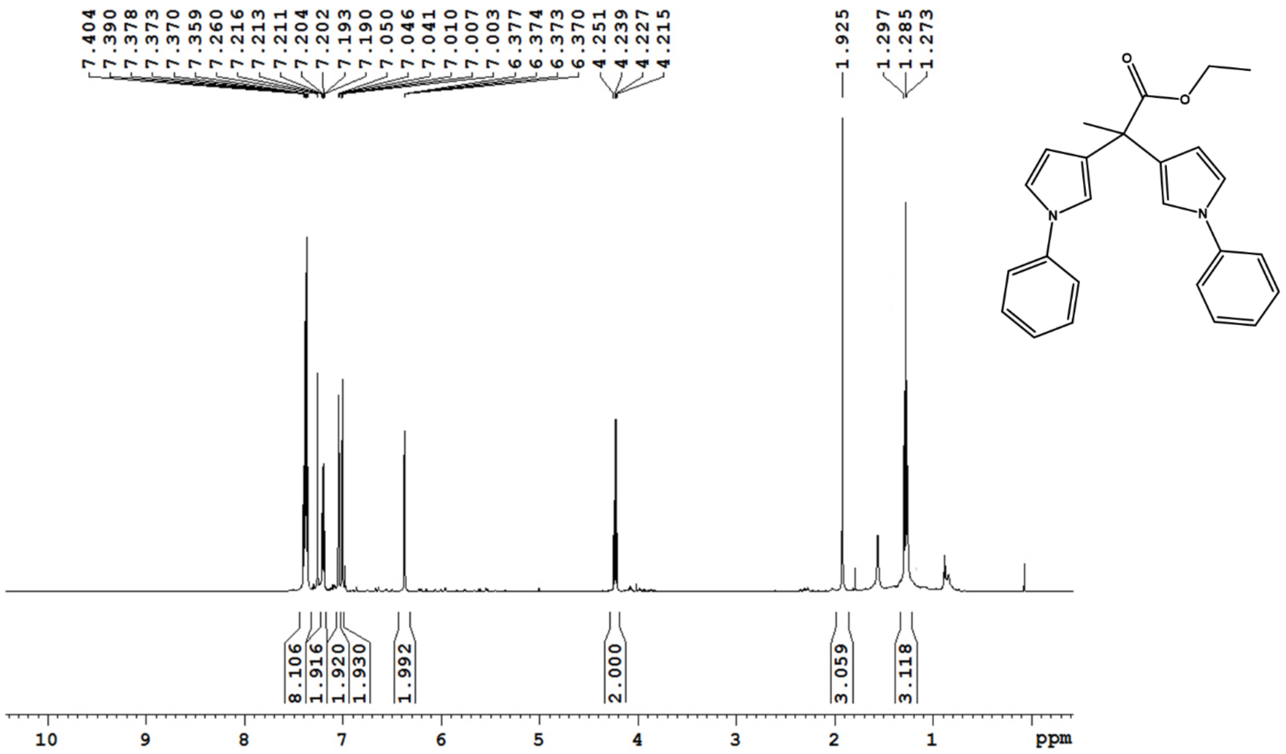

Figure S73. ^{1}H NMR spectrum (600 MHz, $CDCl_3$, 298 K) of the derivative **7ca**.

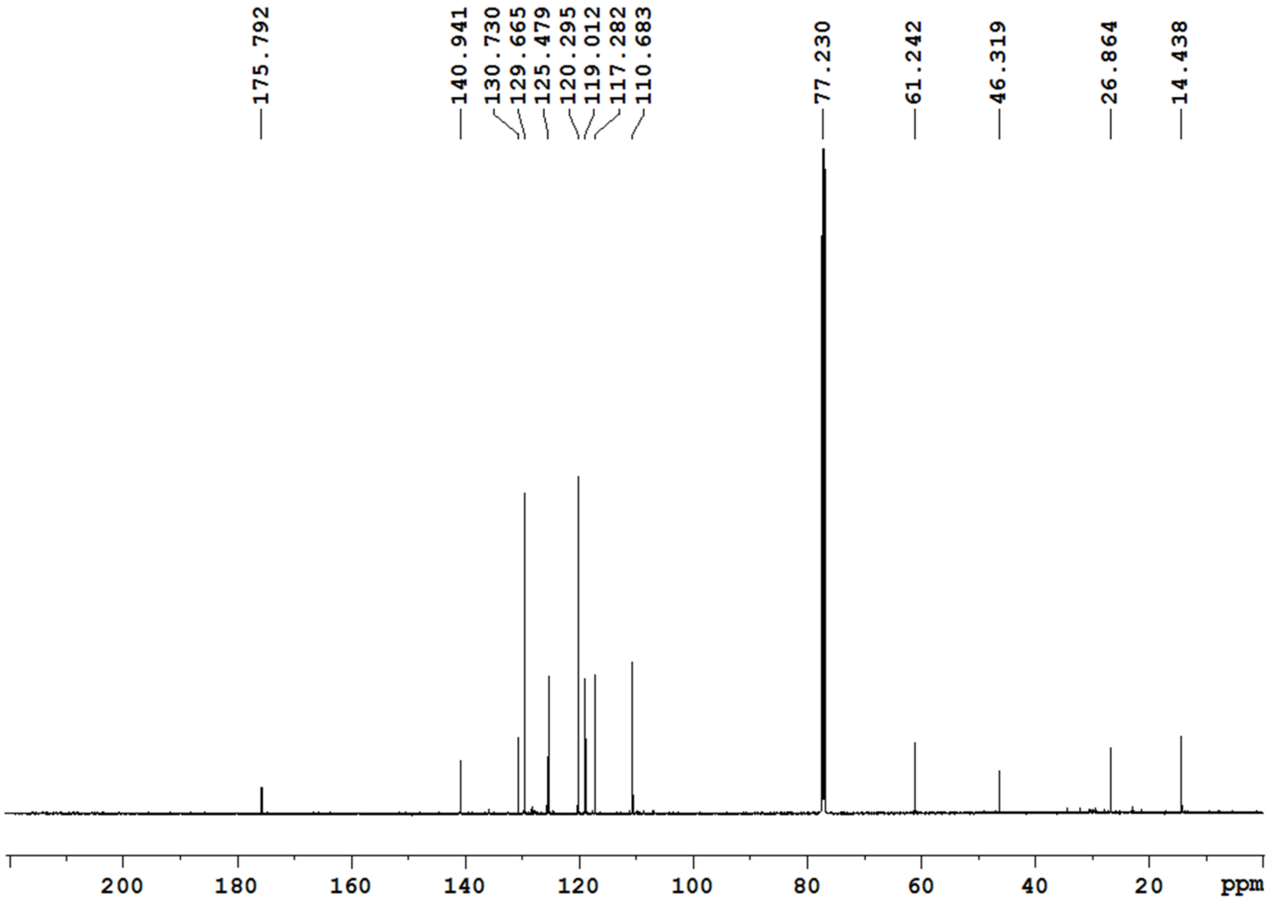

Figure S74. ^{13}C NMR spectrum (150 MHz, $CDCl_3$, 298 K) of the derivative **7ca.**

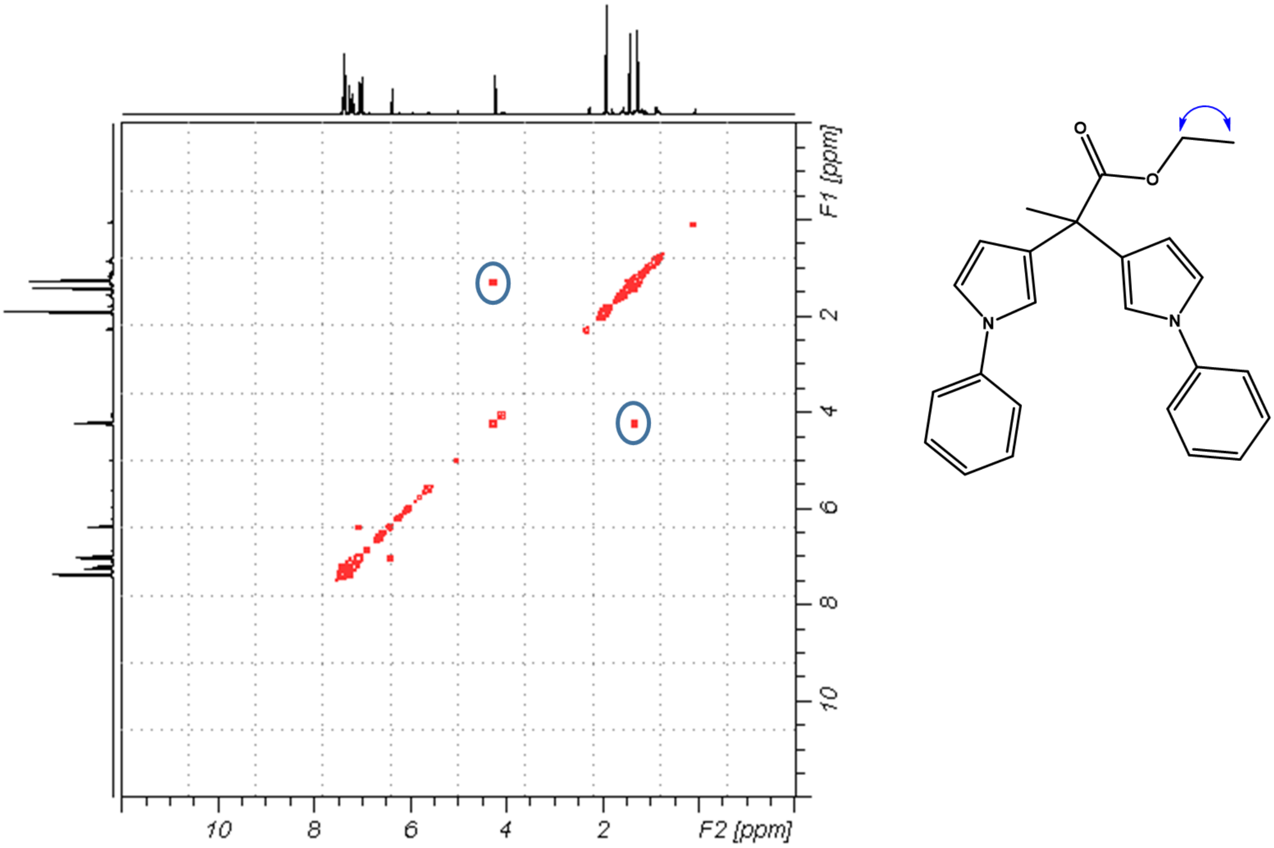

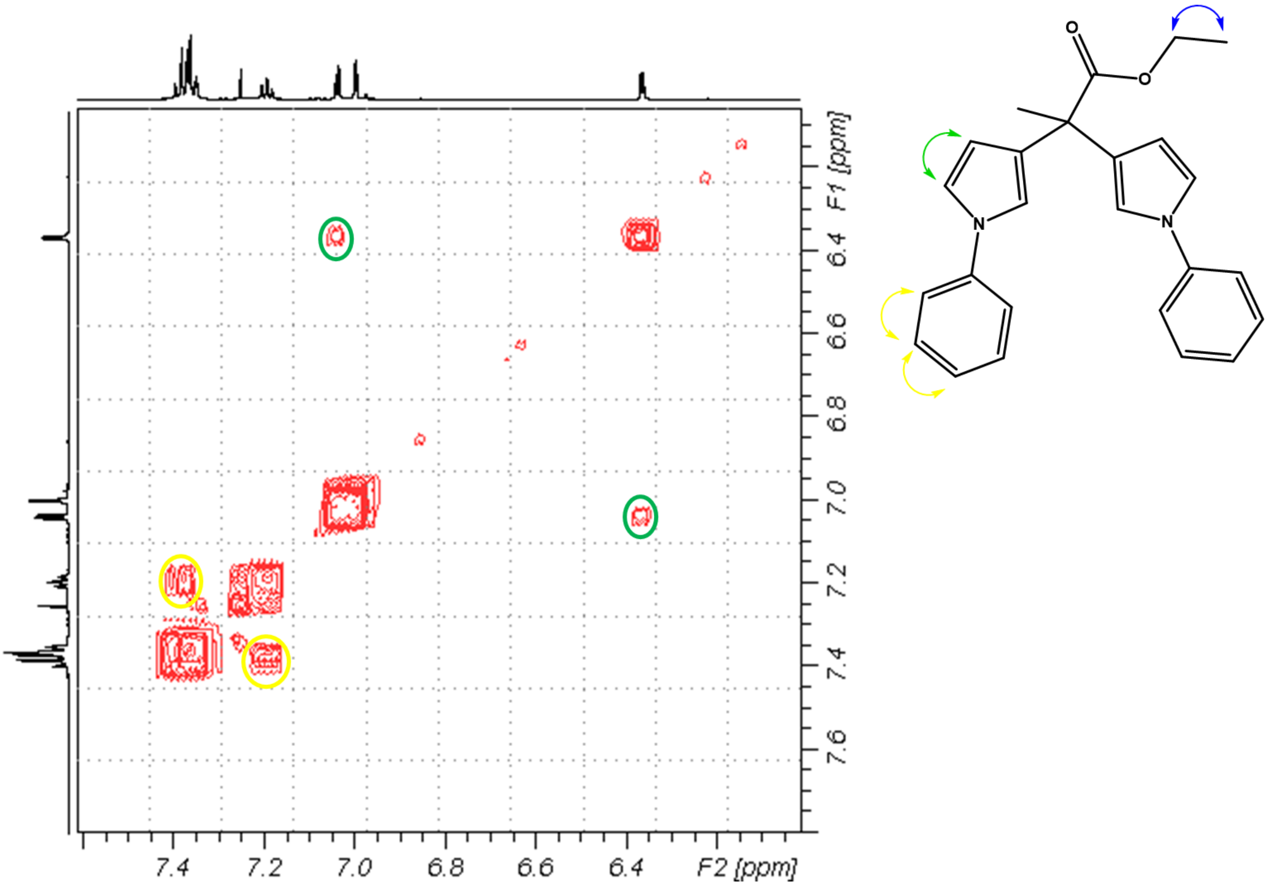

Figure S75. COSY NMR spectrum (600 MHz, $CDCl_3$, 298 K) of the derivative **7ca** with its relevant regions.

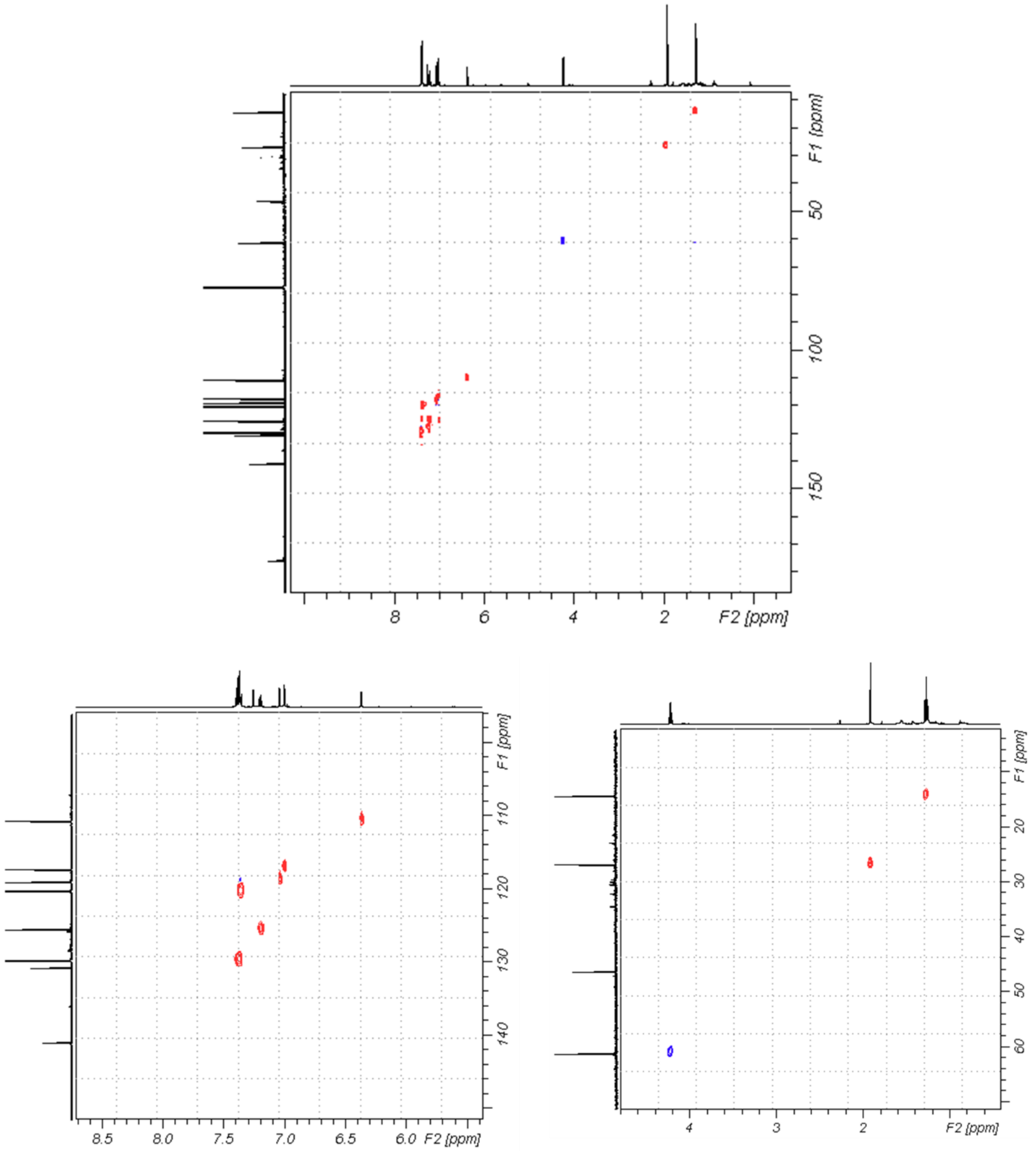

Figure S76. HSQC NMR spectrum (600 MHz, $CDCl_3$, 298 K) of the derivative **7ca** with expansions of the relevant regions. Blue spots are indicative of CH_2 protons, red sposts of CH and CH_3 protons.

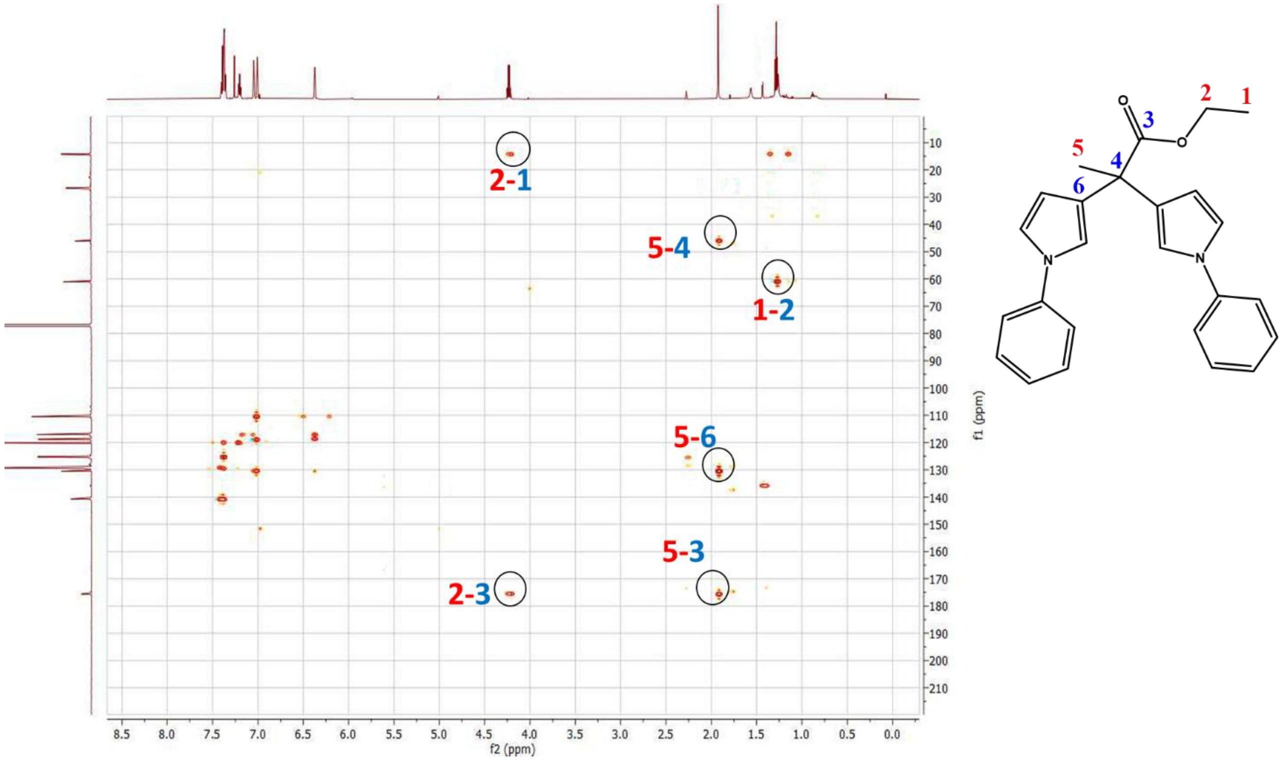

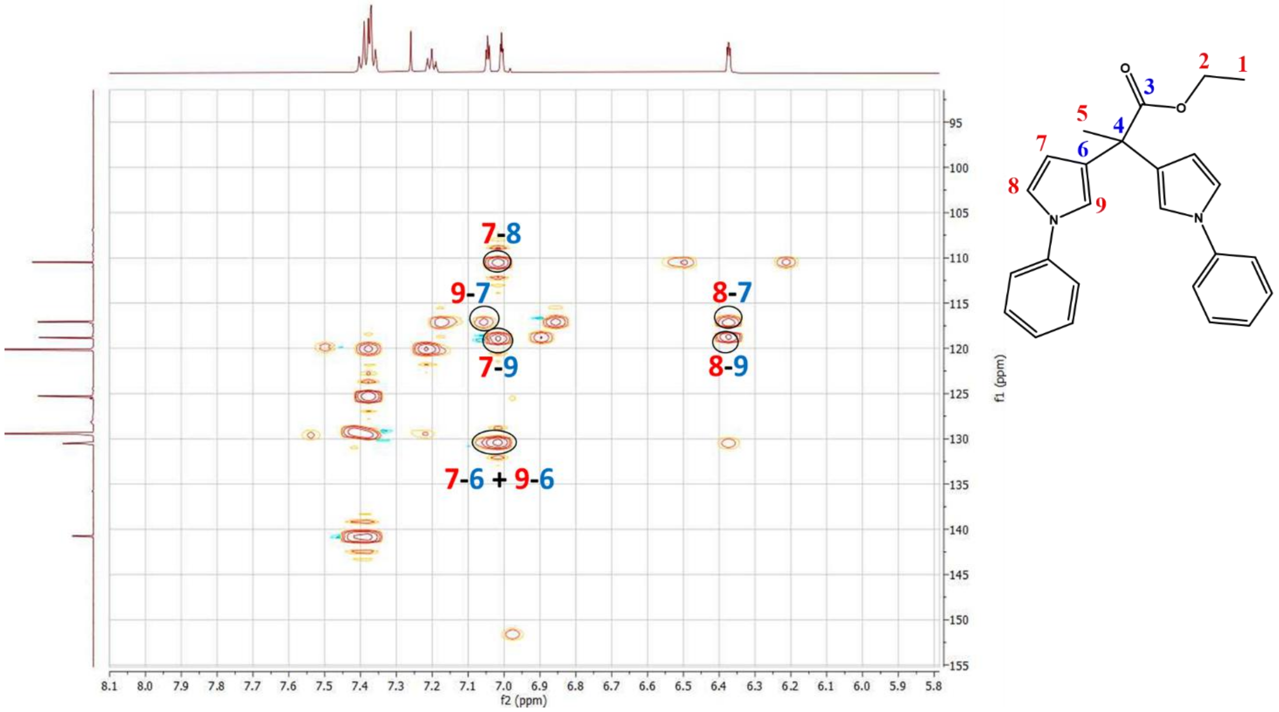

Figure S77. From upper to down: HMBC NMR spectra of derivative **7ca** (600 MHz, 298K, $CDCl_3$).

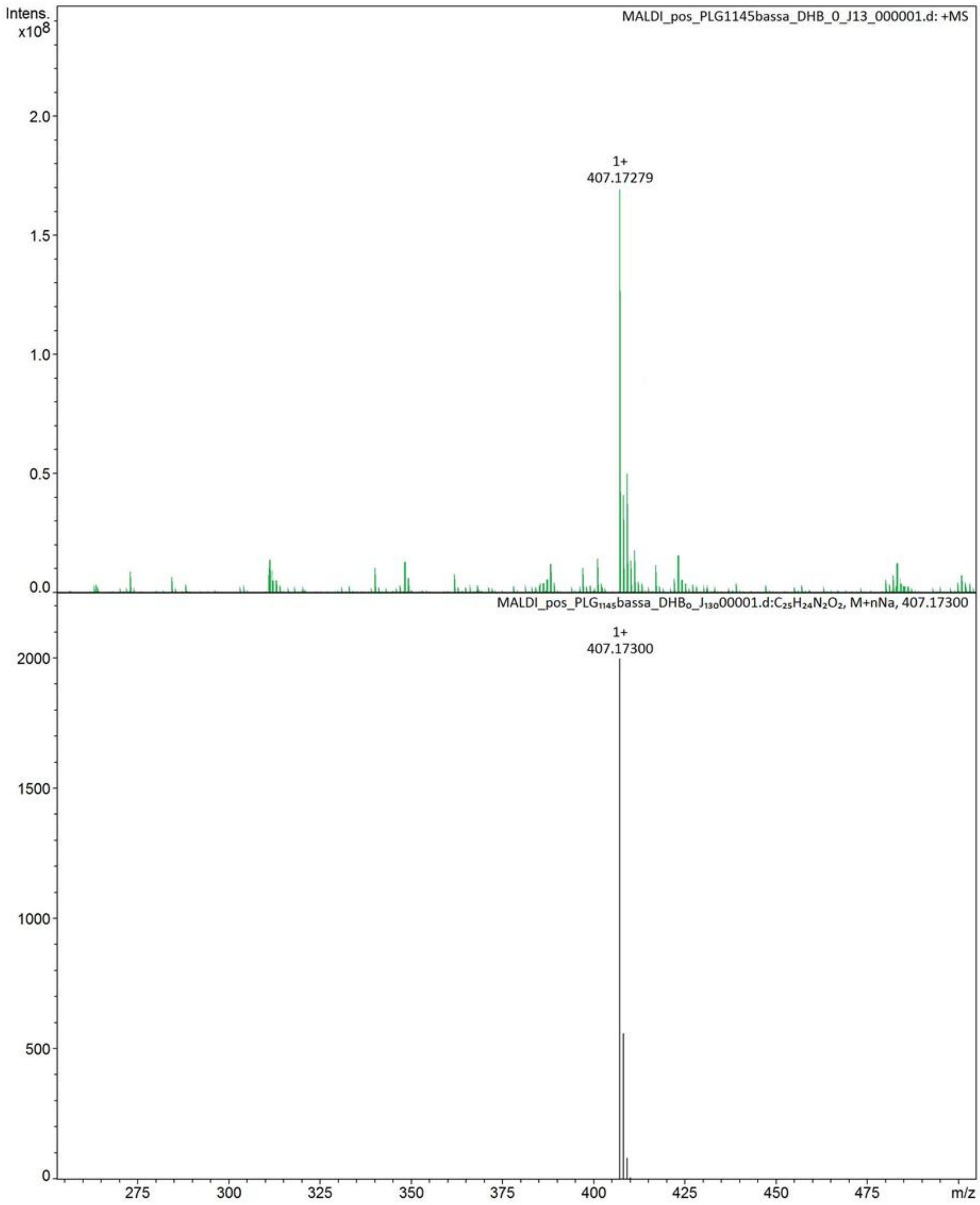

Figure S78: MALDI MS spectrum of the derivative **7ca.**

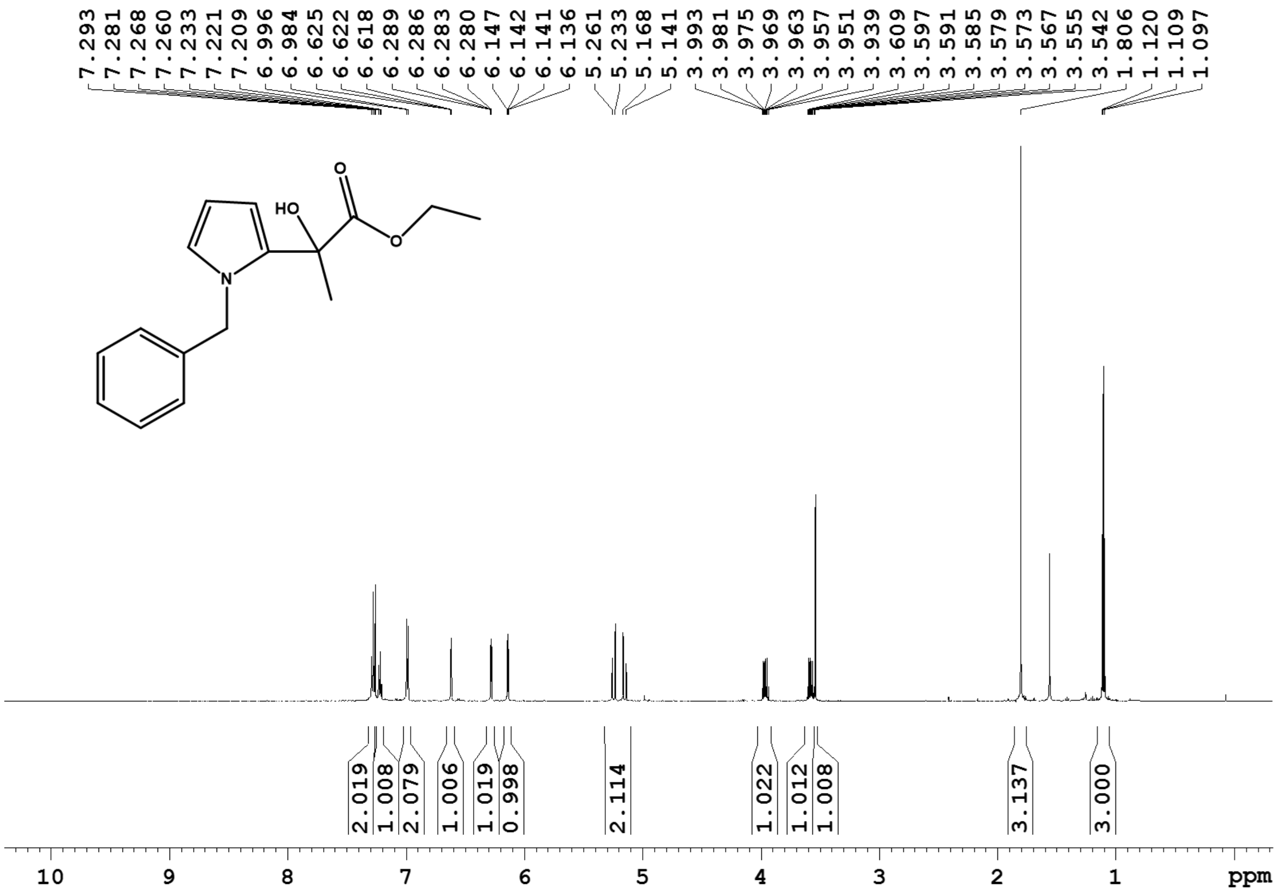

Figure S79. ^{1}H NMR spectrum (600 MHz, $CDCl_3$, 298 K) of the derivative **6da.**

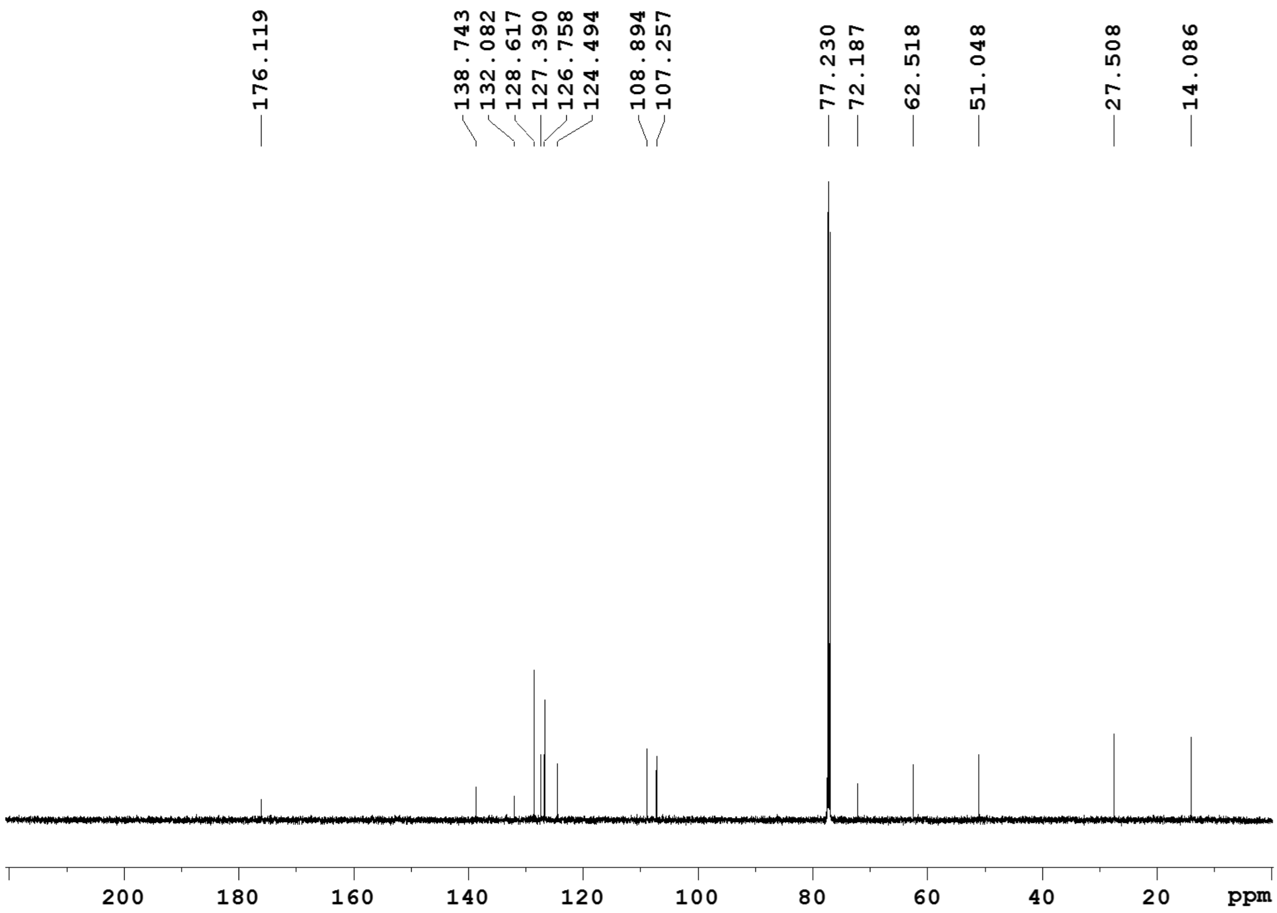

Figure S80. ^{13}C NMR spectrum (150 MHz, $CDCl_3$, 298 K) of the derivative **6da.**

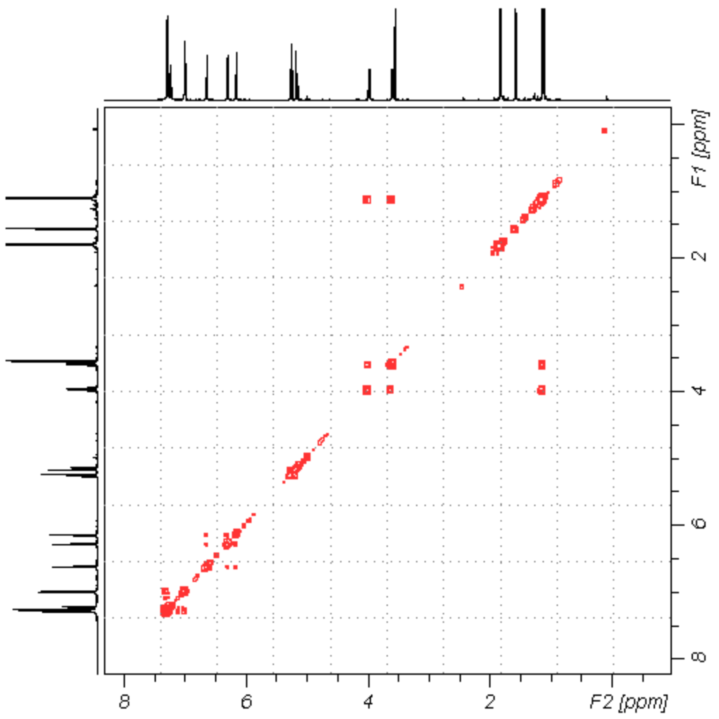

Figure S81. COSY NMR spectrum (600 MHz, $CDCl_3$, 298 K) of the derivative **6da.**

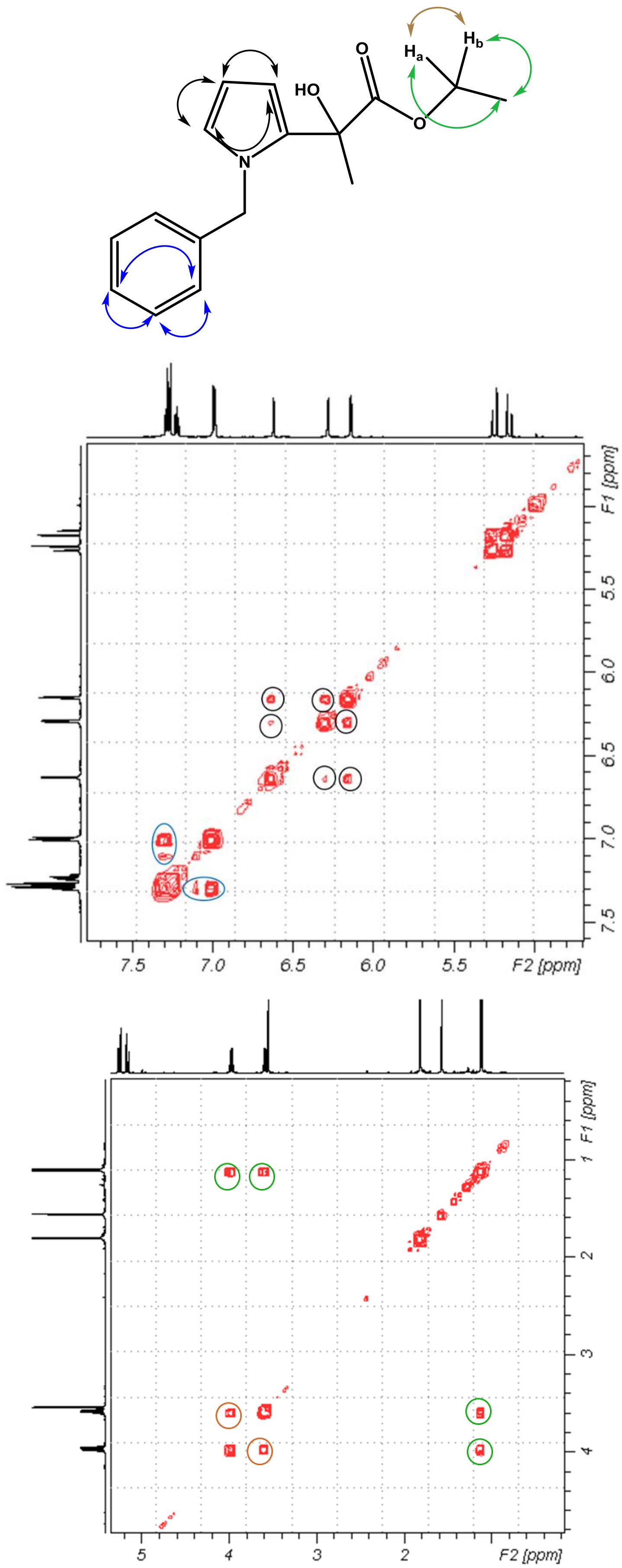

Figure S82. Selected regions of COSY NMR spectrum (600 MHz, $CDCl_3$, 298 K) of the derivative **6da**.

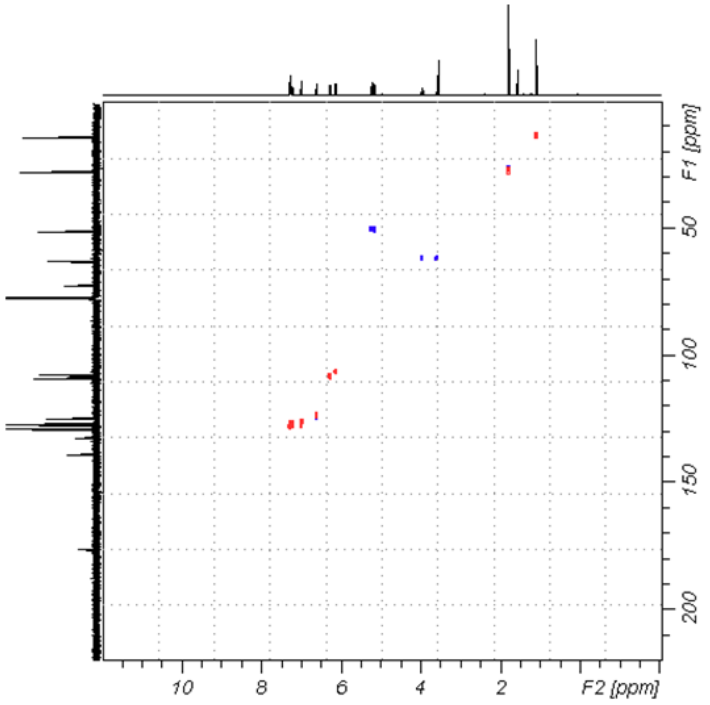

Figure S83. HSQC NMR spectrum (600 MHz, $CDCl_3$, 298 K) of the derivative **6da**.

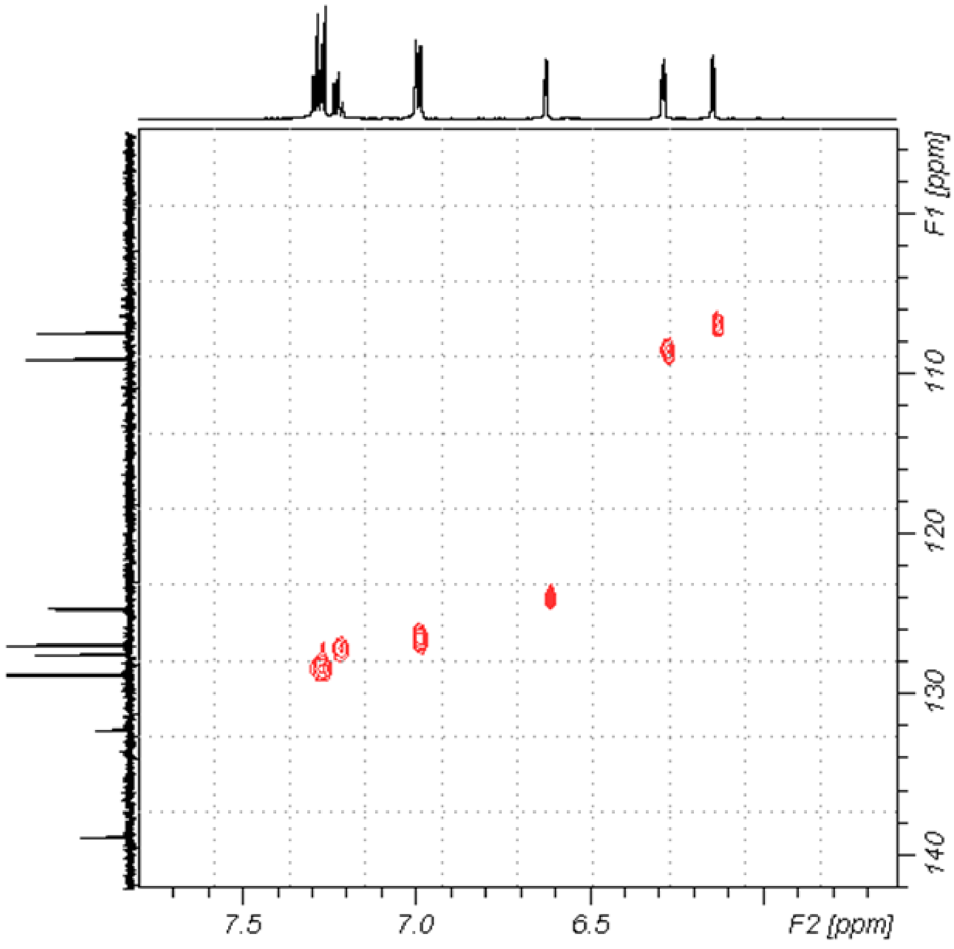

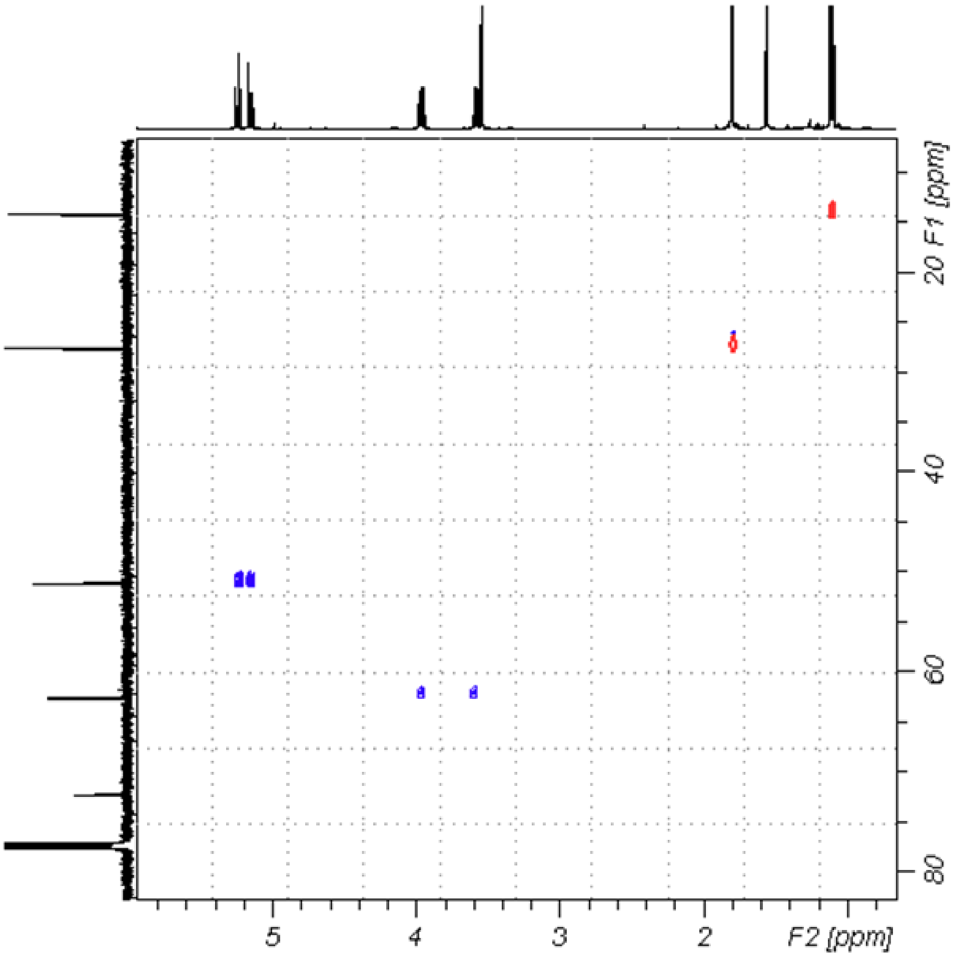

Figure S84. Upper: selected regions of HSQC NMR spectrum (600 MHz, $CDCl_3$, 298 K) of the derivative **6da.**

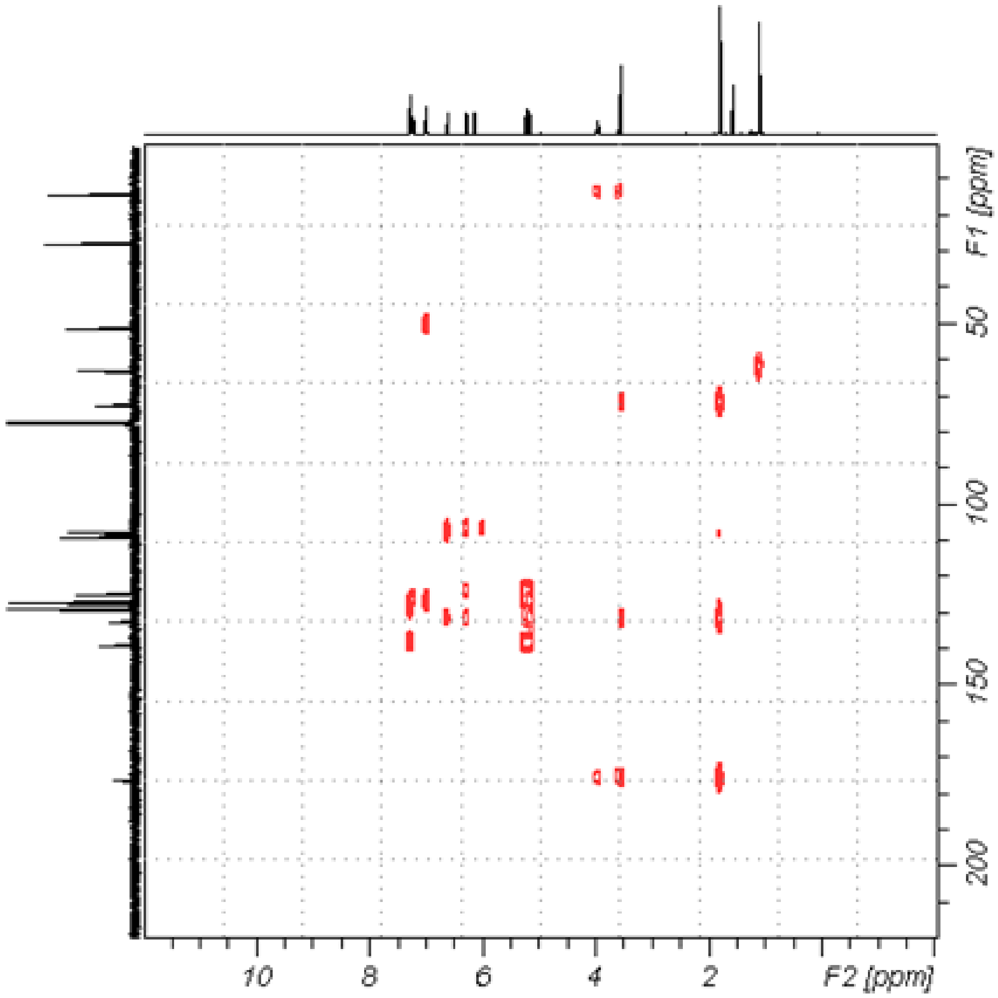

Figure S85. HMBC NMR spectrum (600 MHz, $CDCl_3$, 298 K) of the derivative **6da**.

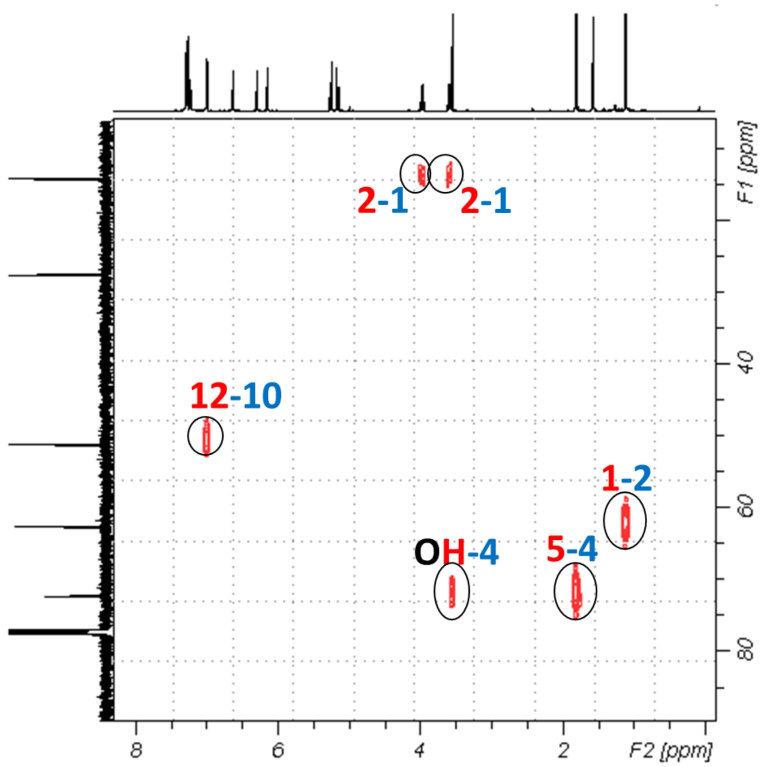
2-1
2-1
12-10
1-2
OH-4
5-4
F1 [ppm]
F2 [ppm]

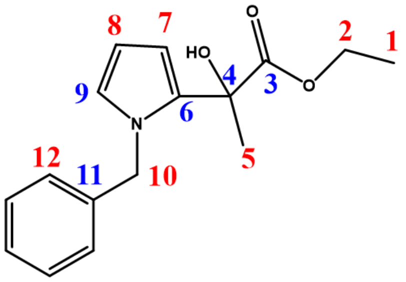
HO
1
2
3
4
5
6
7
8
9
10
11
12
N
O

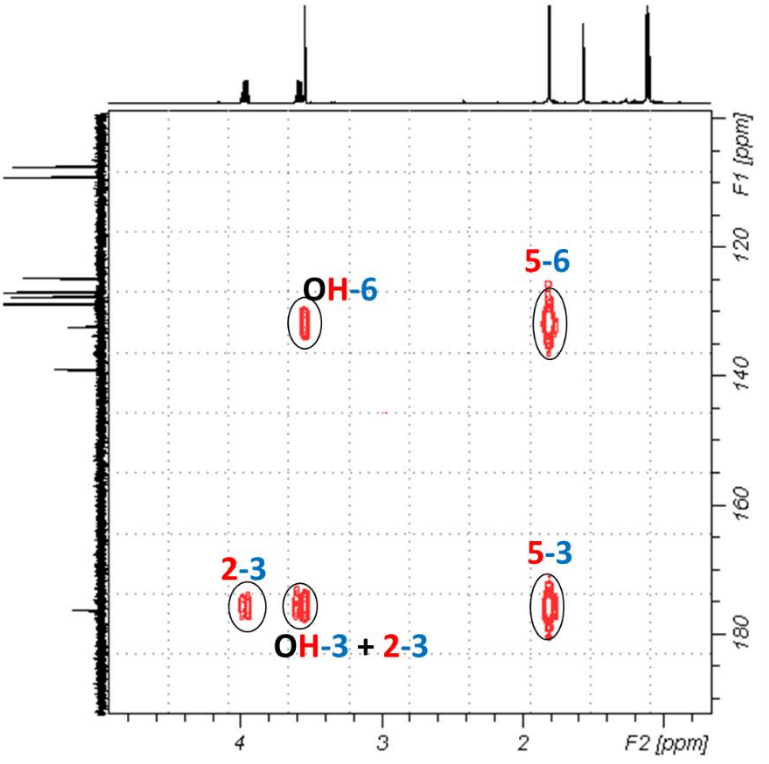
OH-6
5-6
2-3
OH-3 + 2-3
5-3
F1 [ppm]
F2 [ppm]

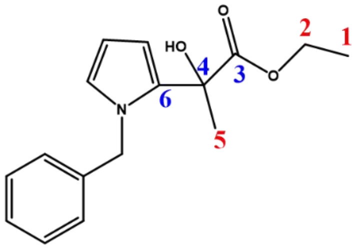
HO
1
2
3
4
5
6
N
O

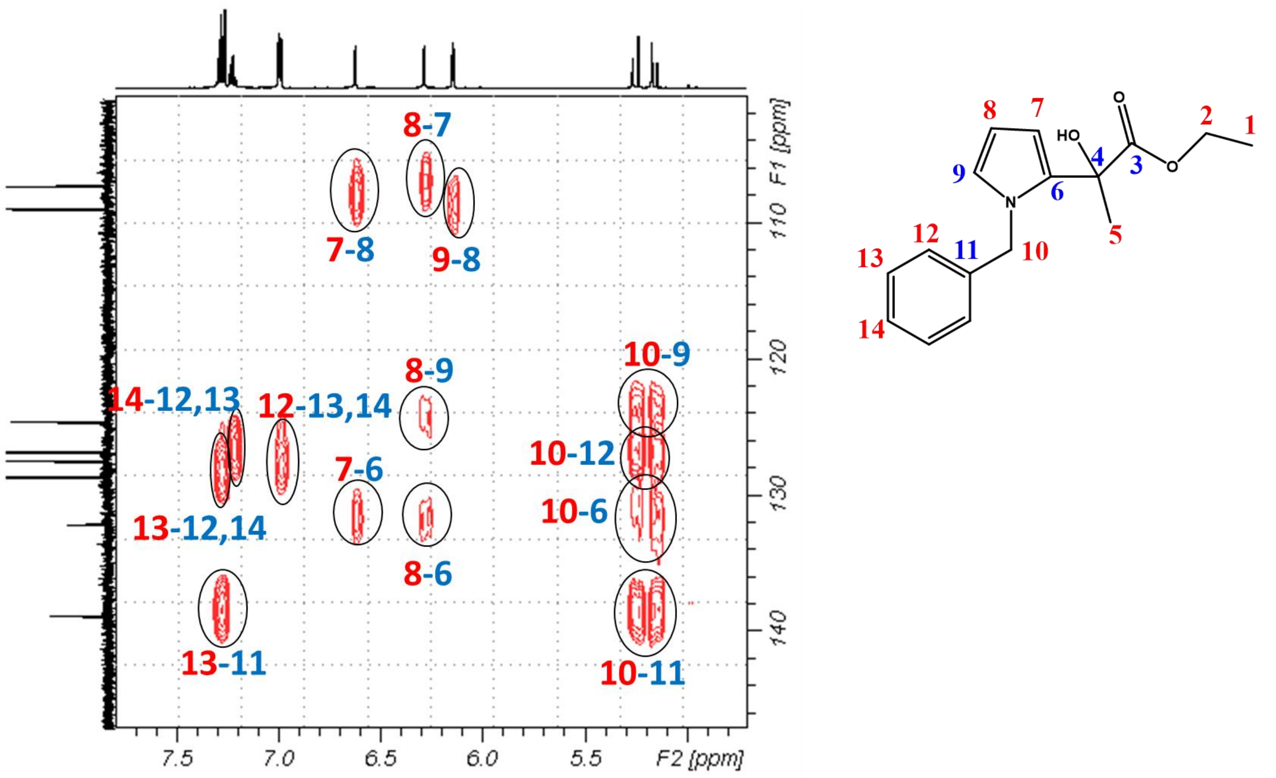

Figure S86. Selected regions of HMBC NMR spectrum (600 MHz, CDCl3, 298 K) of the derivative **6da**.

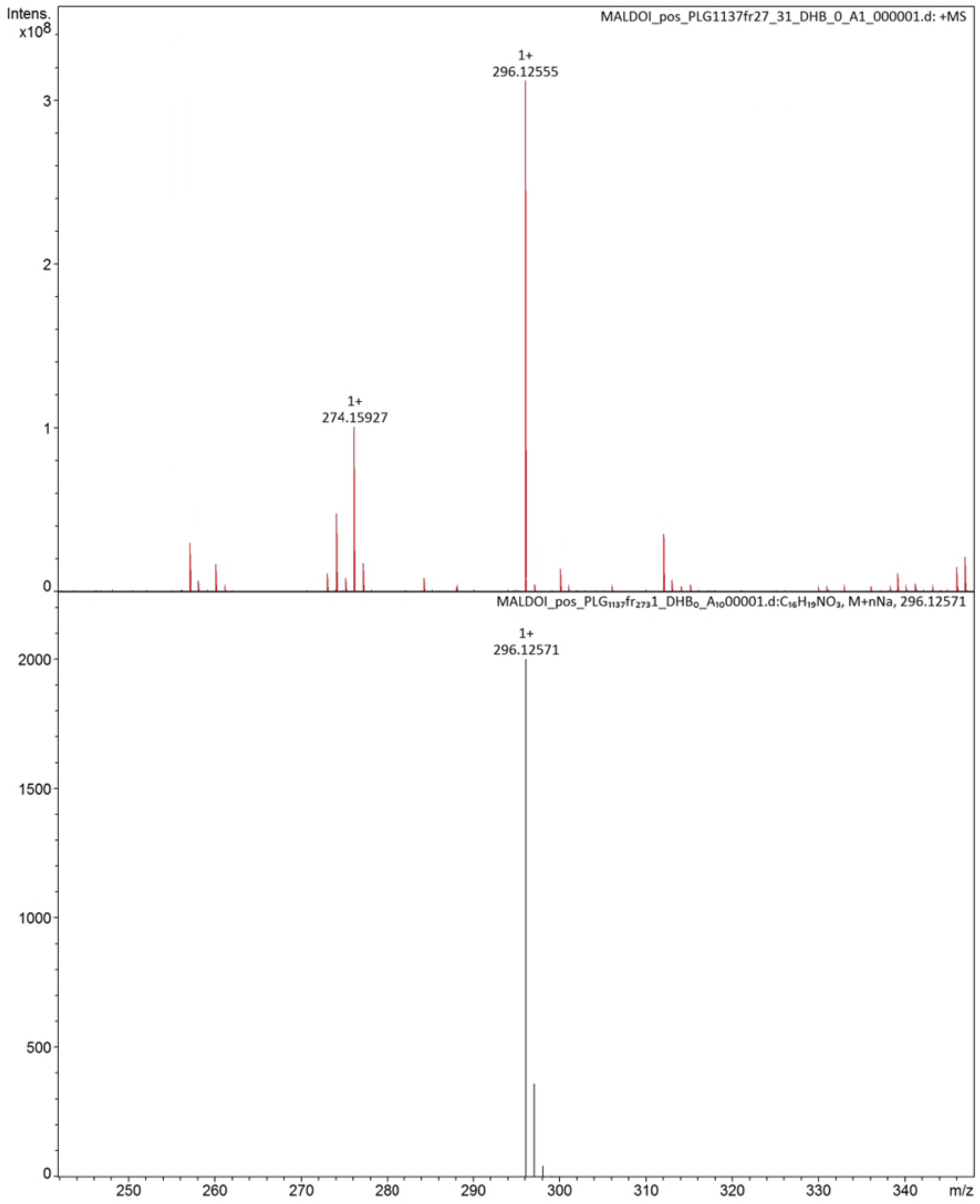

Figure S87. MALDI MS spectrum of the derivative **6da.**

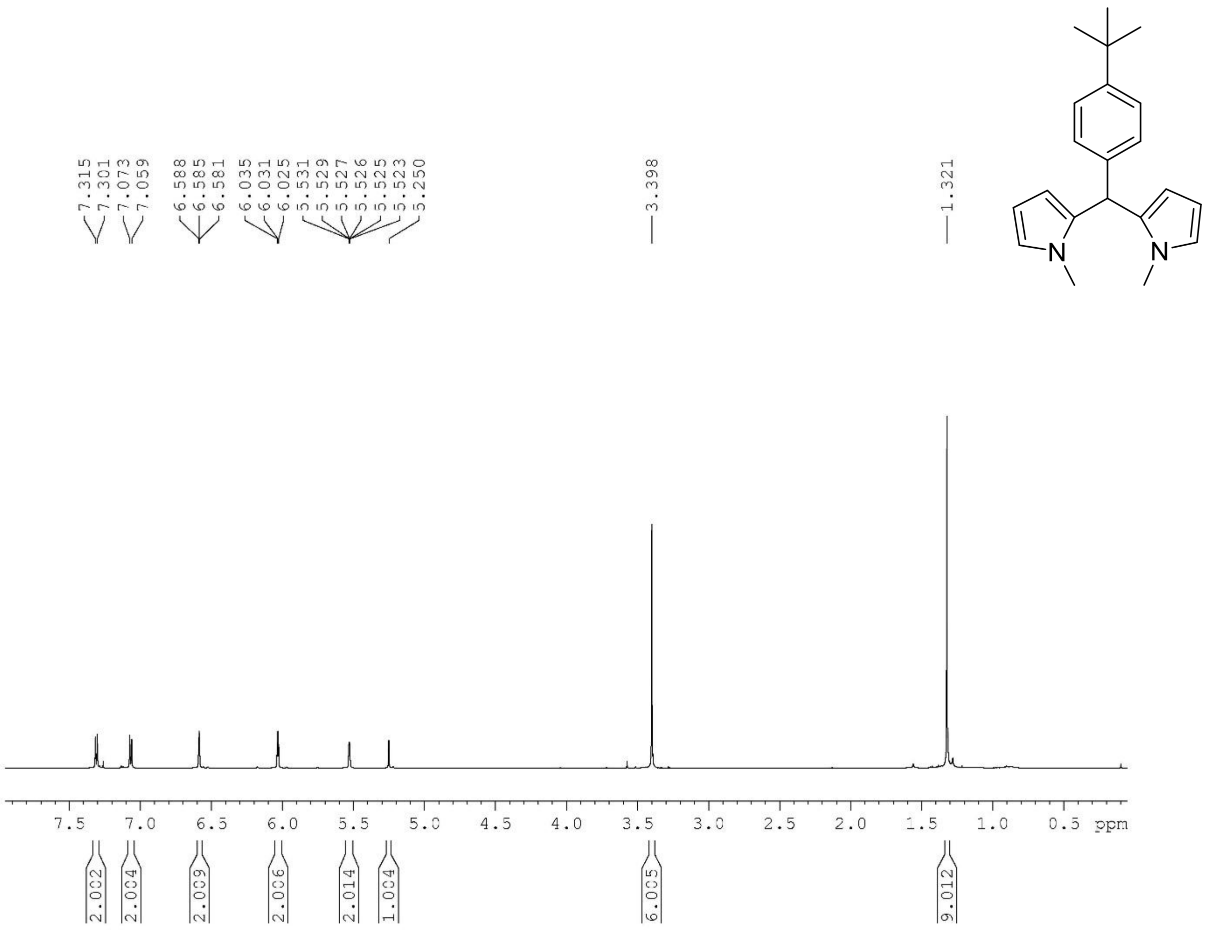

Figure S88. ^{1}H NMR (600 MHz, $CDCl_3$, 298 K) of the derivative

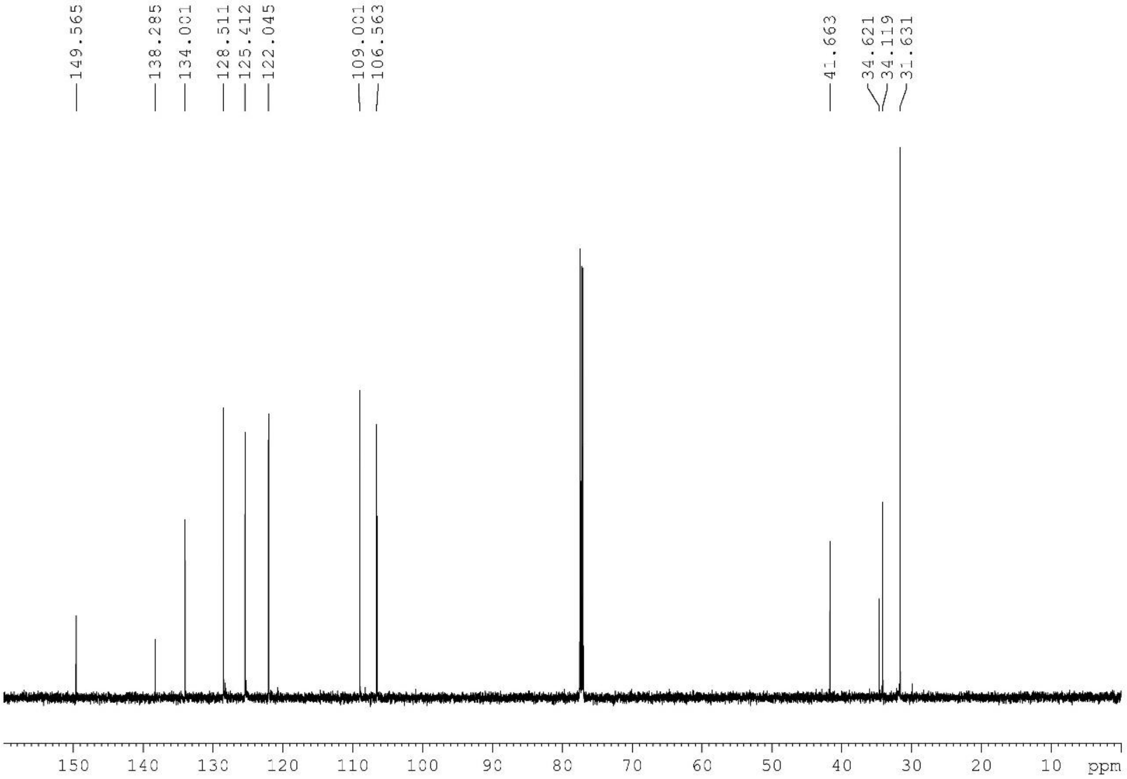

Figure S89. ^{13}C NMR (150 MHz, $CDCl_3$, 298 K) of the derivative **11ai**

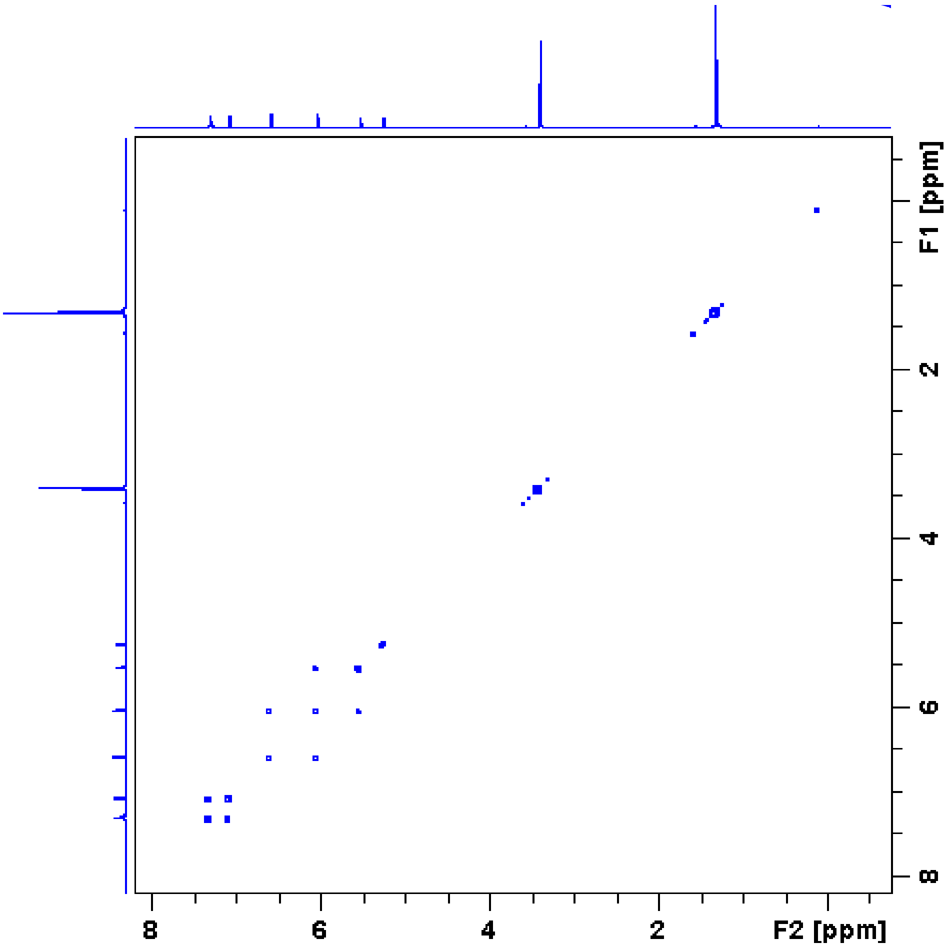

Figure S90. COSY NMR spectrum (600 MHz, $CDCl_3$, 298 K) of the derivative **11ai.**

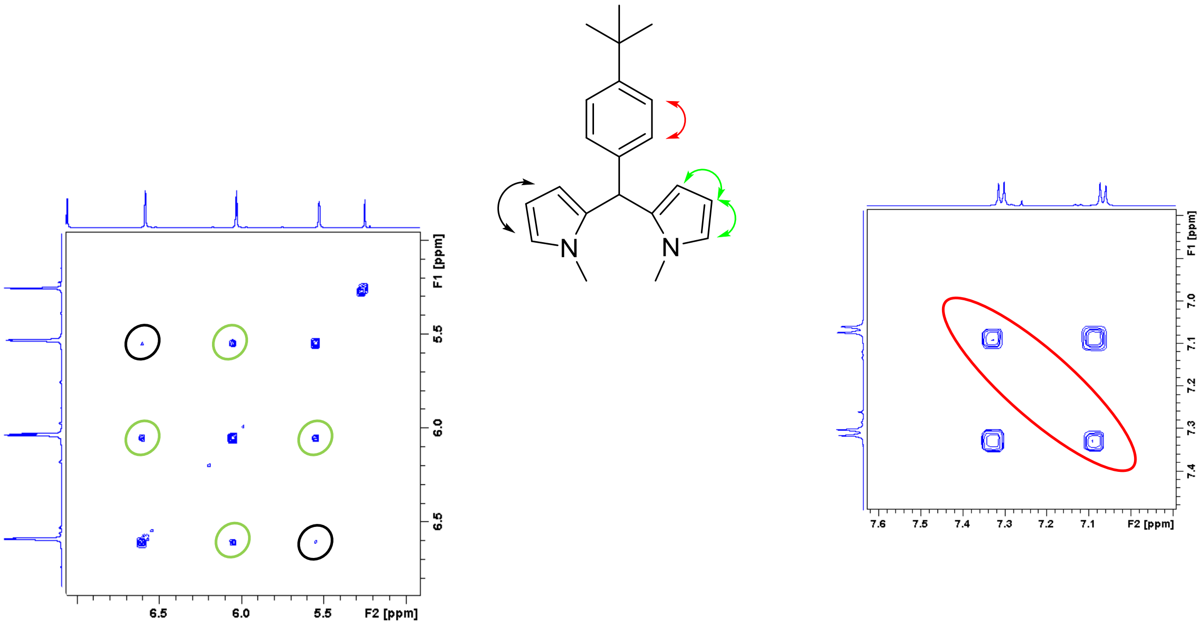

Fig of the derivative
11

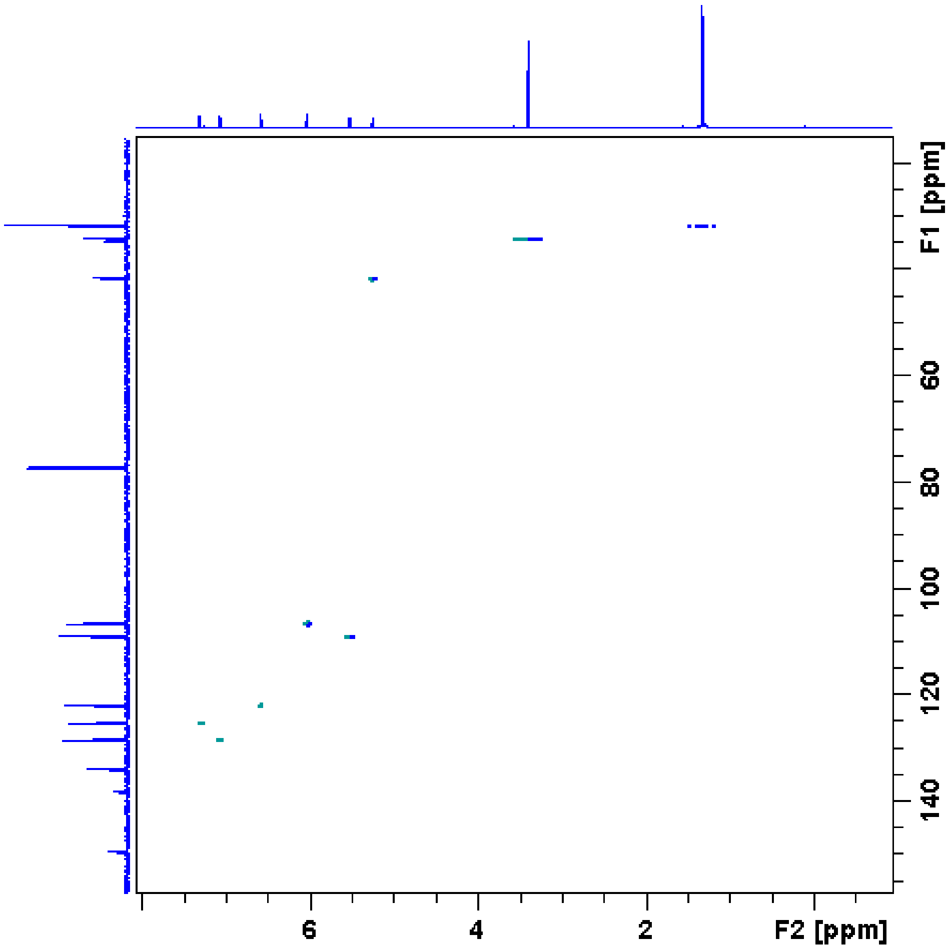

Figure S92. HSQC NMR spectrum (600 MHz, $CDCl_3$, 298 K) of the derivative **11ai.**

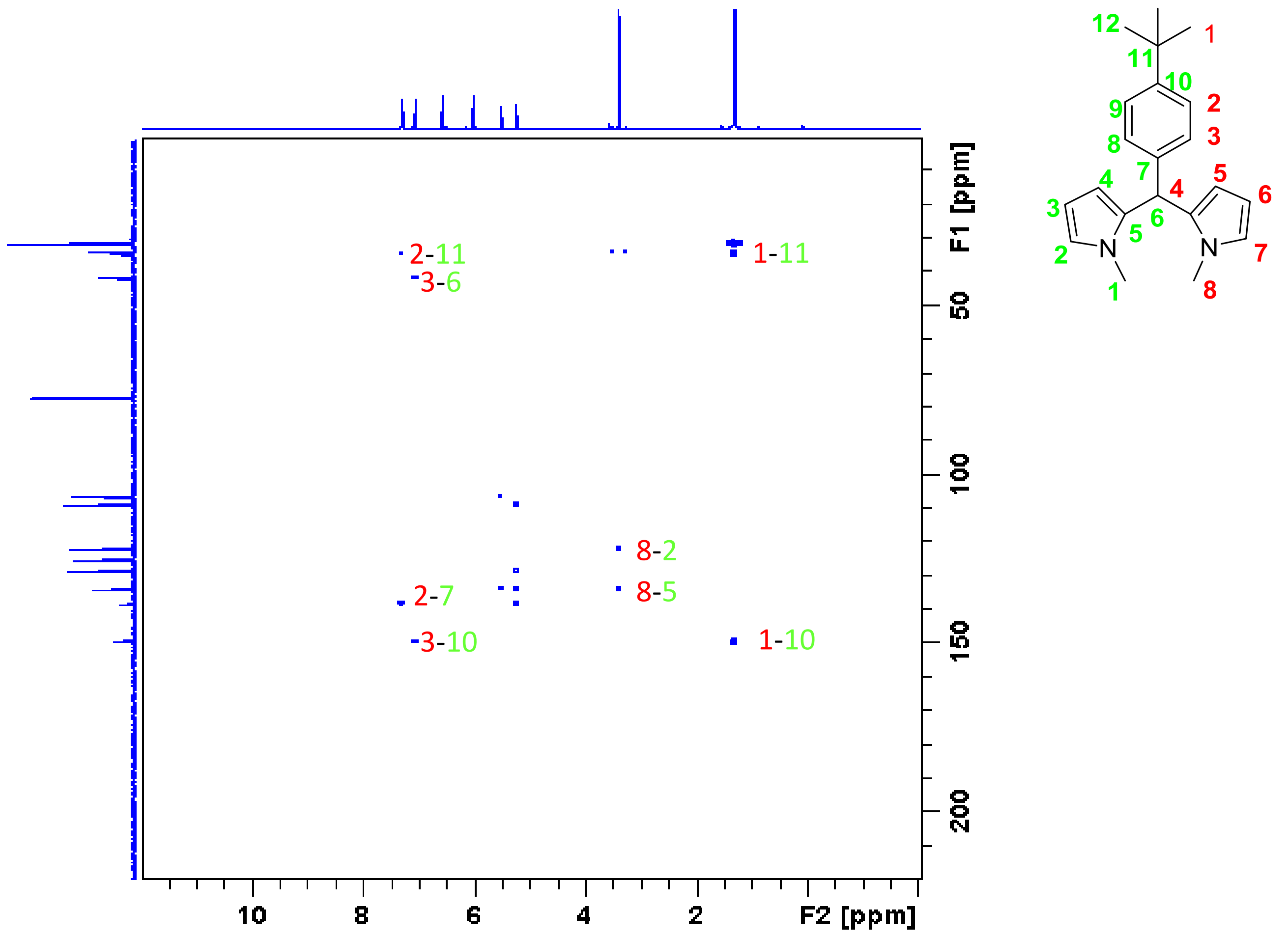

Figure S93. HMBC NMR spectrum (600 MHz, $CDCl_3$, 298 K) of the derivative **11ai.**

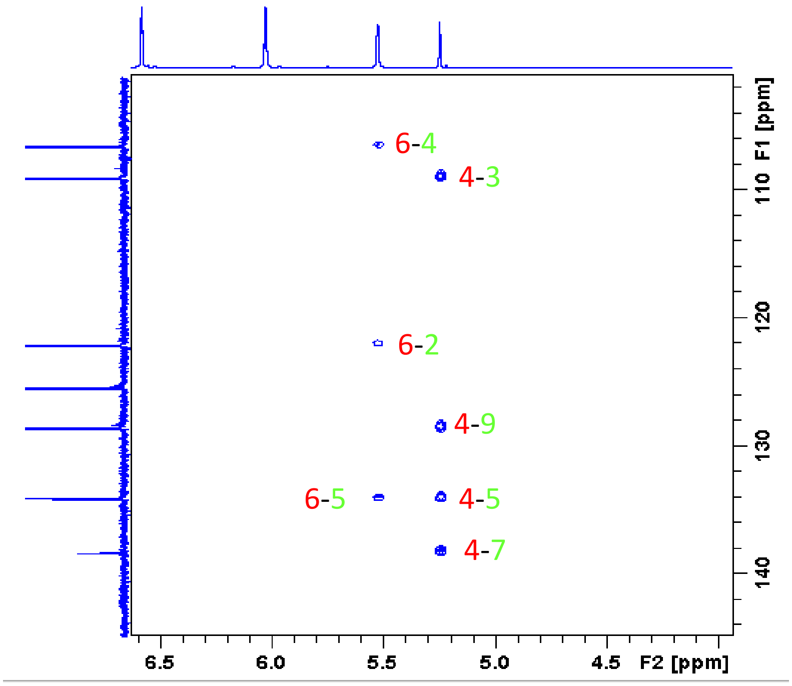

Figure S94. Relevant region of HMBC NMR spectrum (600 MHz, $CDCl_3$, 298 K) of the derivative **11ai.**

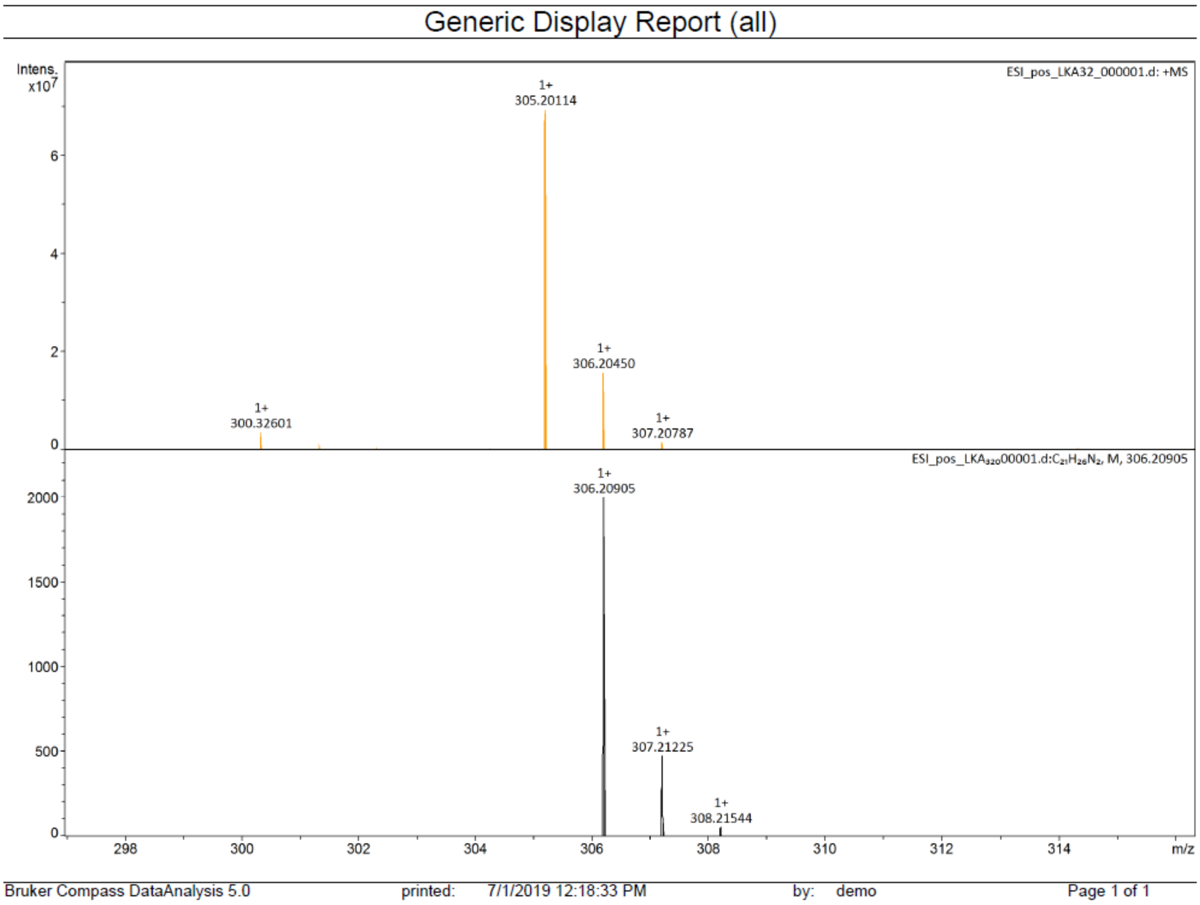

Figure S95. ESI MS spectrum of the derivative **11ai.**

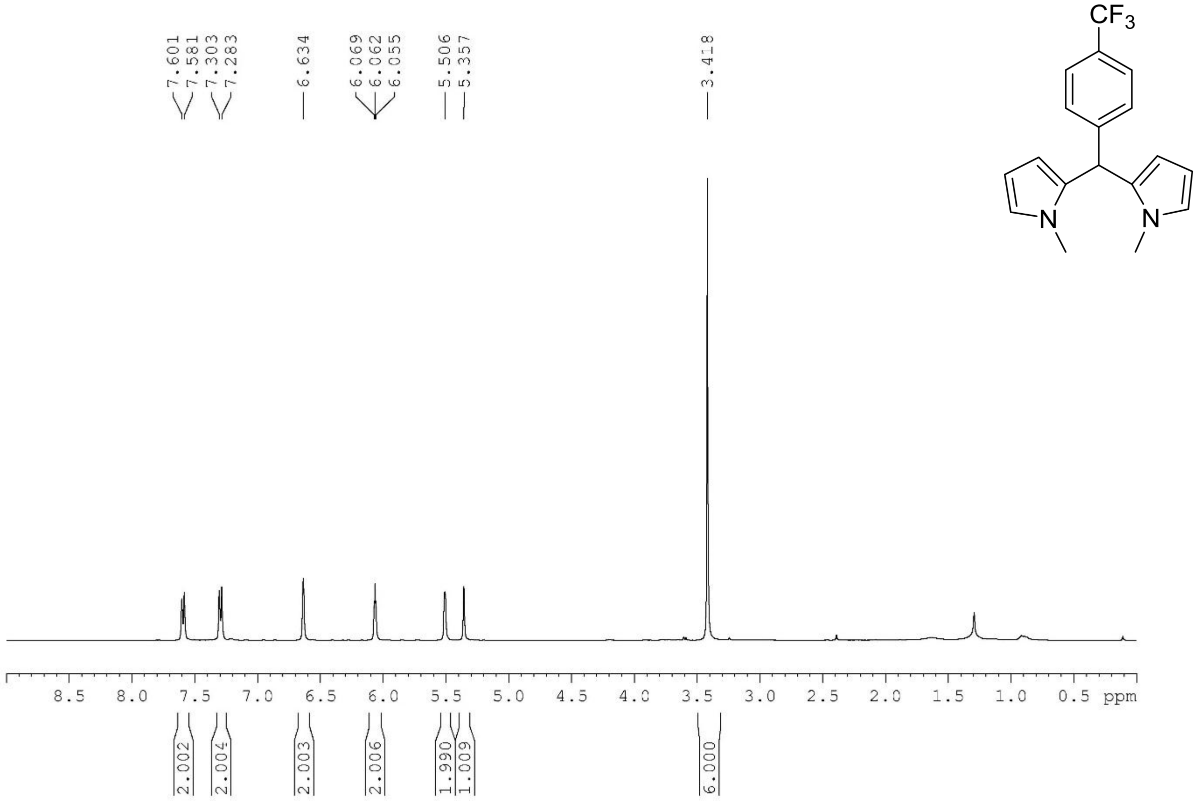

Figure S96. ^{1}H NMR (400 MHz, $CDCl_3$, 298 K) of the derivative **11ad**

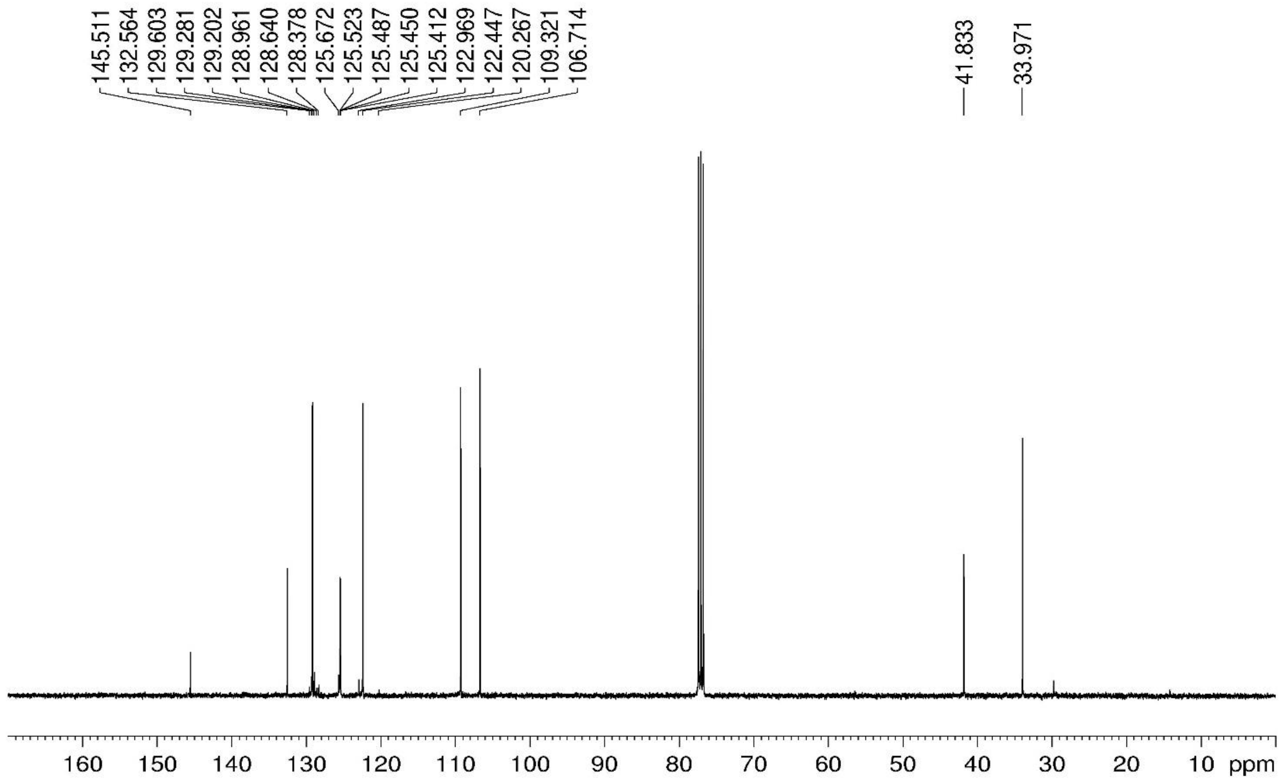

Figure S97. ^{13}C NMR (100 MHz, $CDCl_3$, 298 K) of the derivative **11ad**

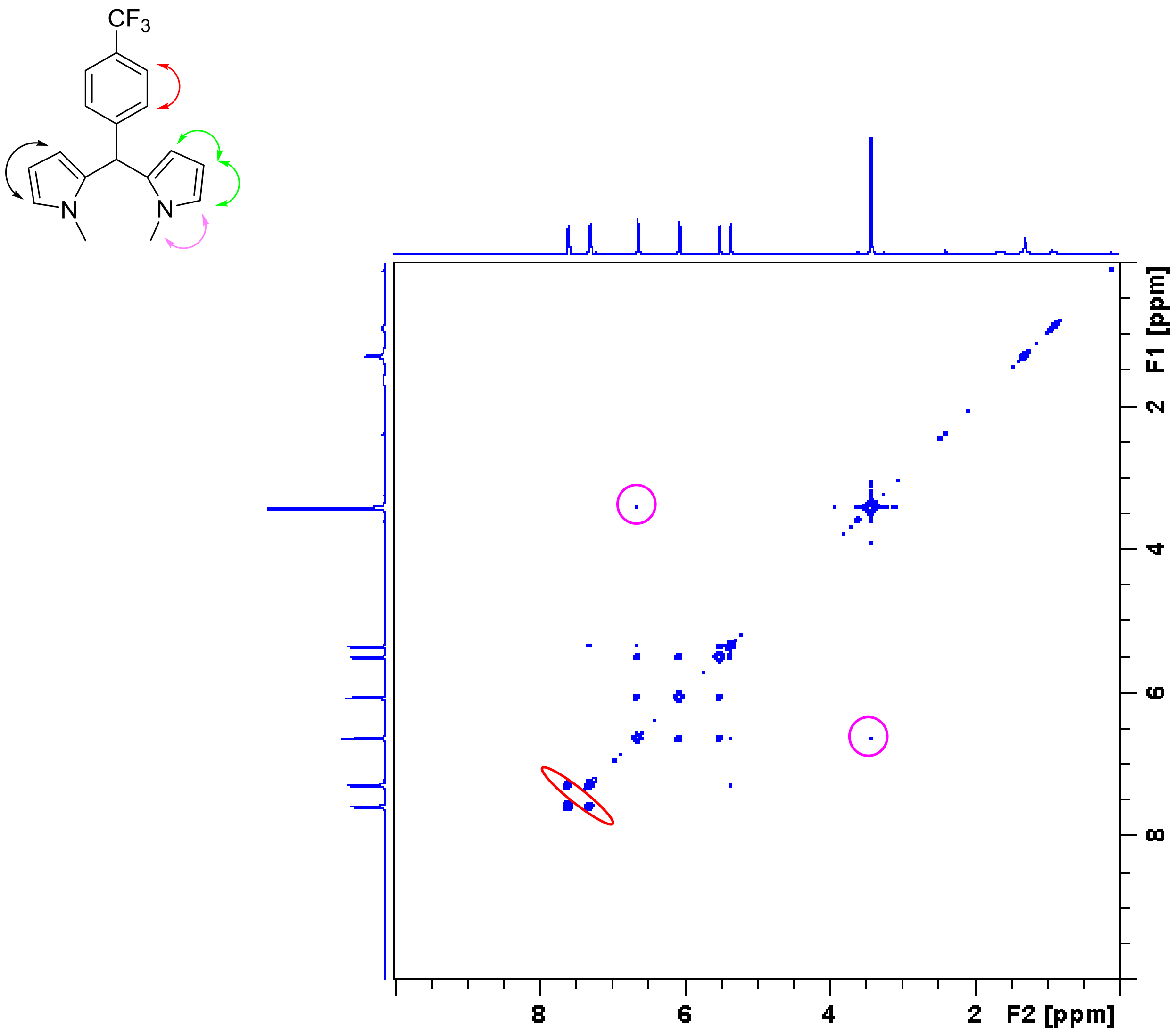

Figure S98. COSY NMR spectrum (400 MHz, $CDCl_3$, 298 K) of the derivative **11ad.**

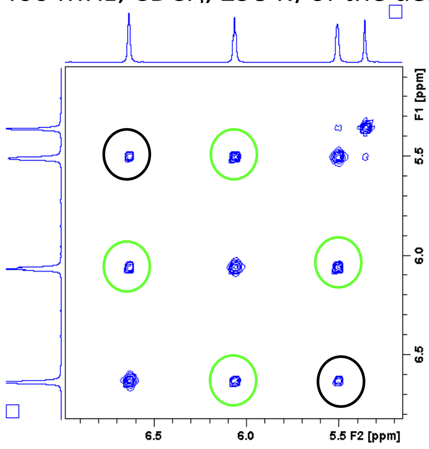

Figure S99. Expansion of COSY NMR spectrum (400 MHz, $CDCl_3$, 298 K) of the derivative **11ad.**

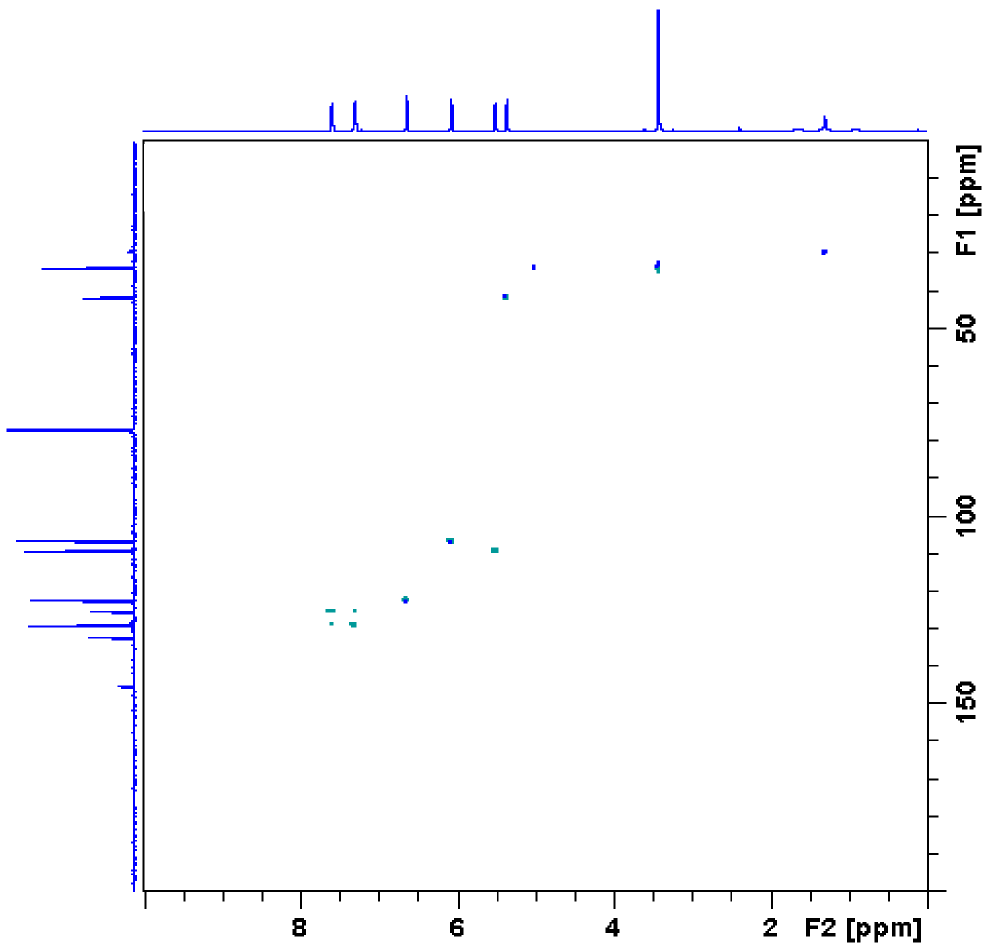

Figure S100. HSQC NMR spectrum 400 MHz, $CDCl_3$, 298 K) of the derivative **11ad.**

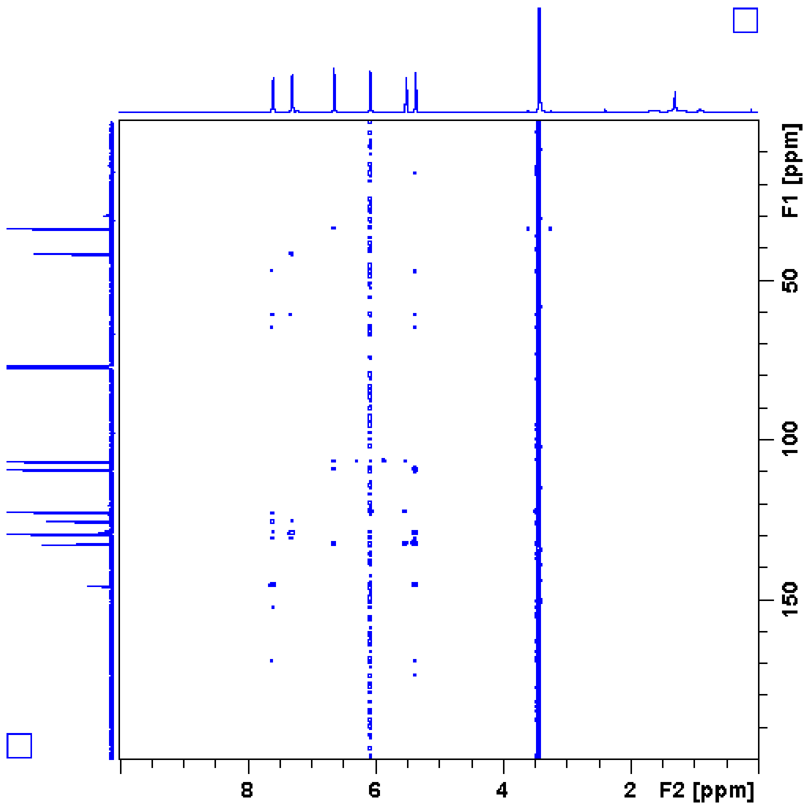

Figure S101. HMBC NMR spectrum (400 MHz, $CDCl_3$, 298 K) of the derivative **11ad.**

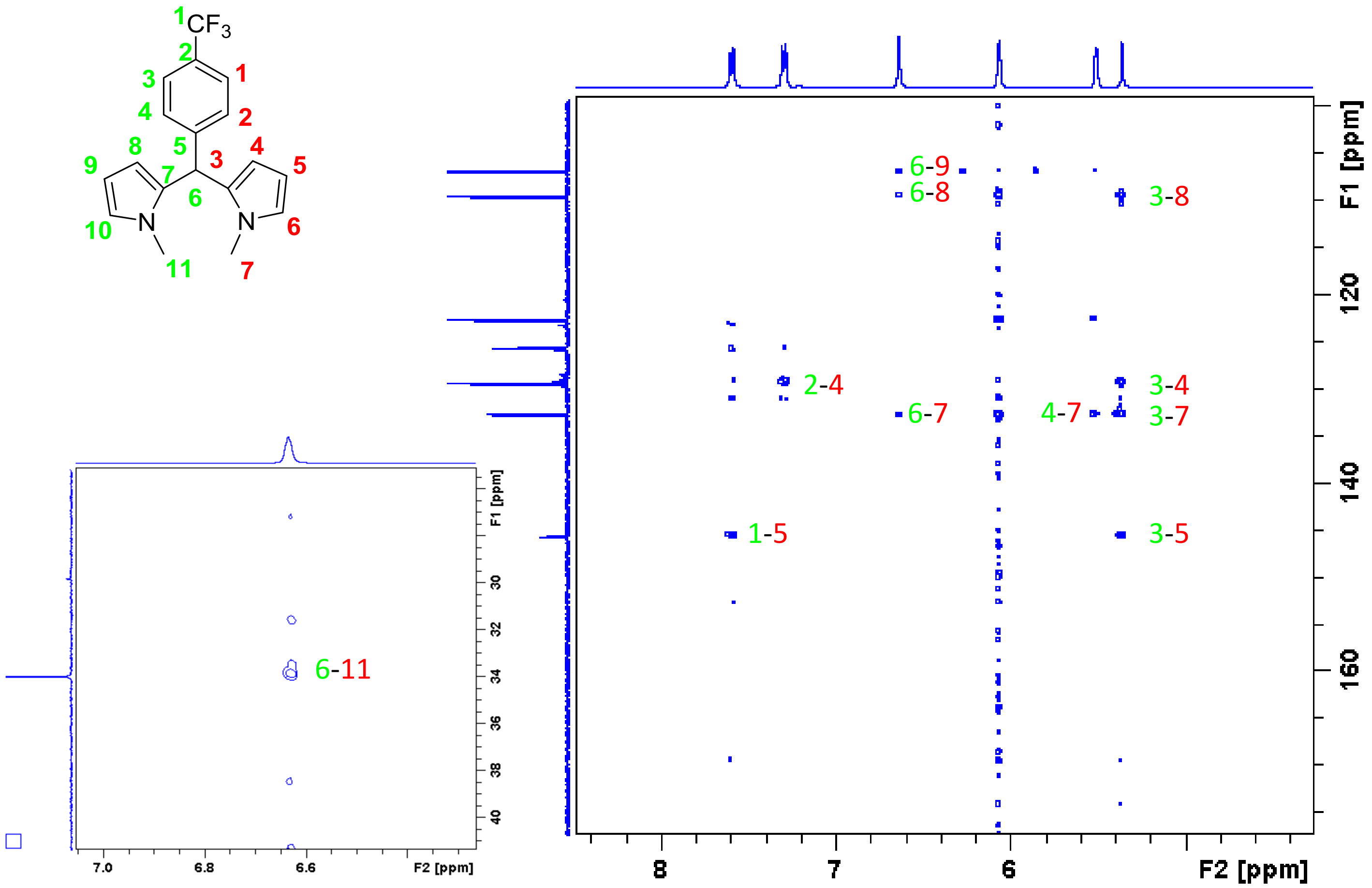

Figure S102. Expansions of HMBC NMR spectrum (400 MHz, $CDCl_3$, 298 K) of the derivative **11ad.**

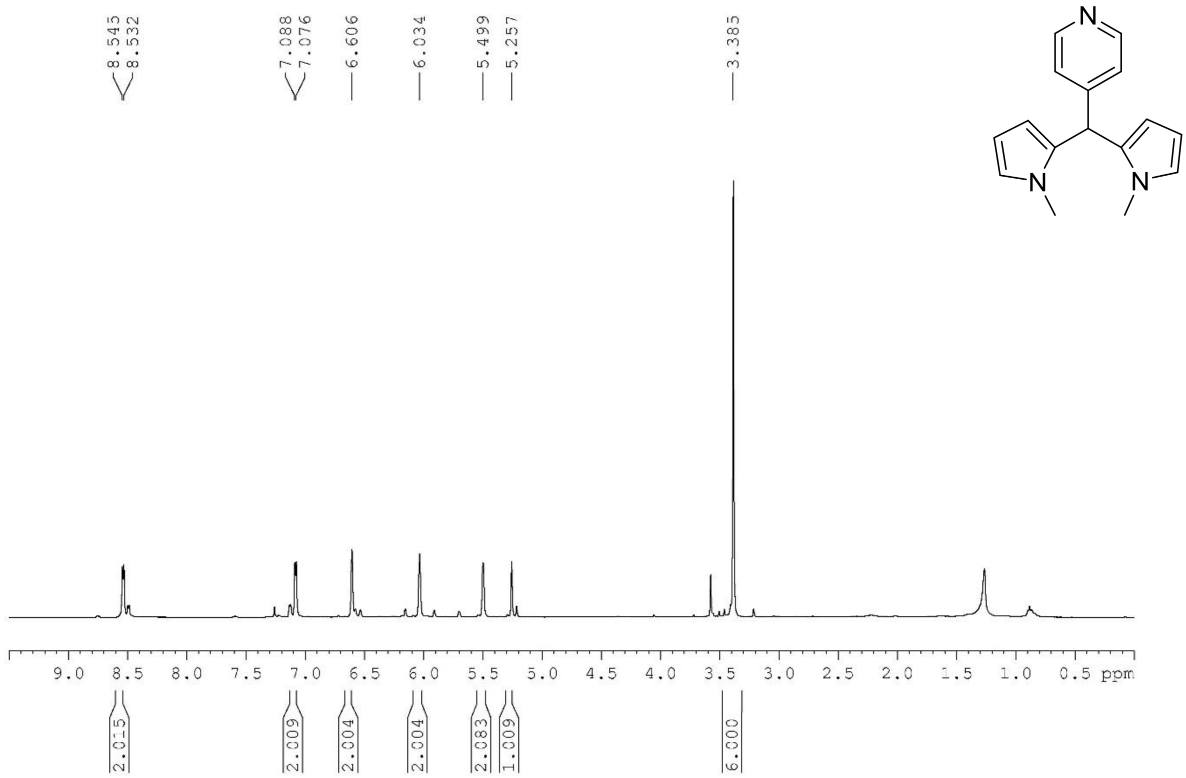

Figure S103. ^{1}H NMR (400 MHz, $CDCl_3$, 298 K) of the derivative **11aj.**

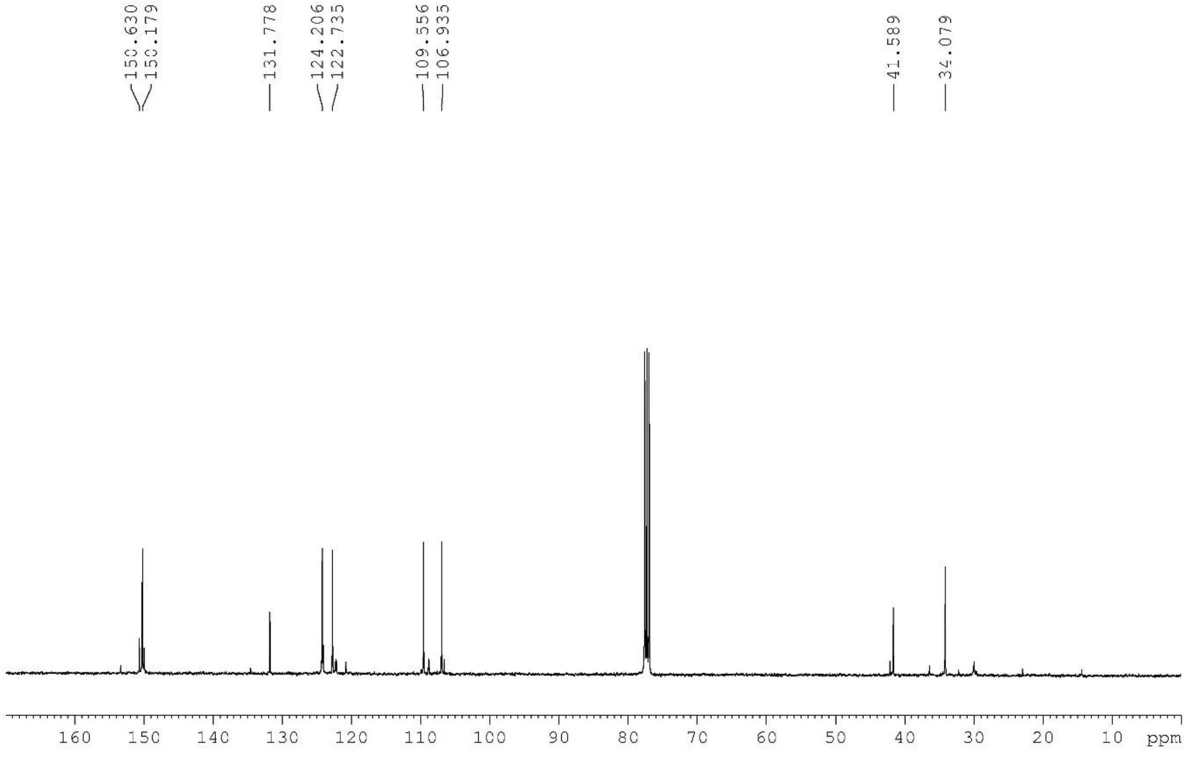

Figure S104. ^{13}C NMR (100 MHz, $CDCl_3$, 298 K) of the derivative **11aj.**

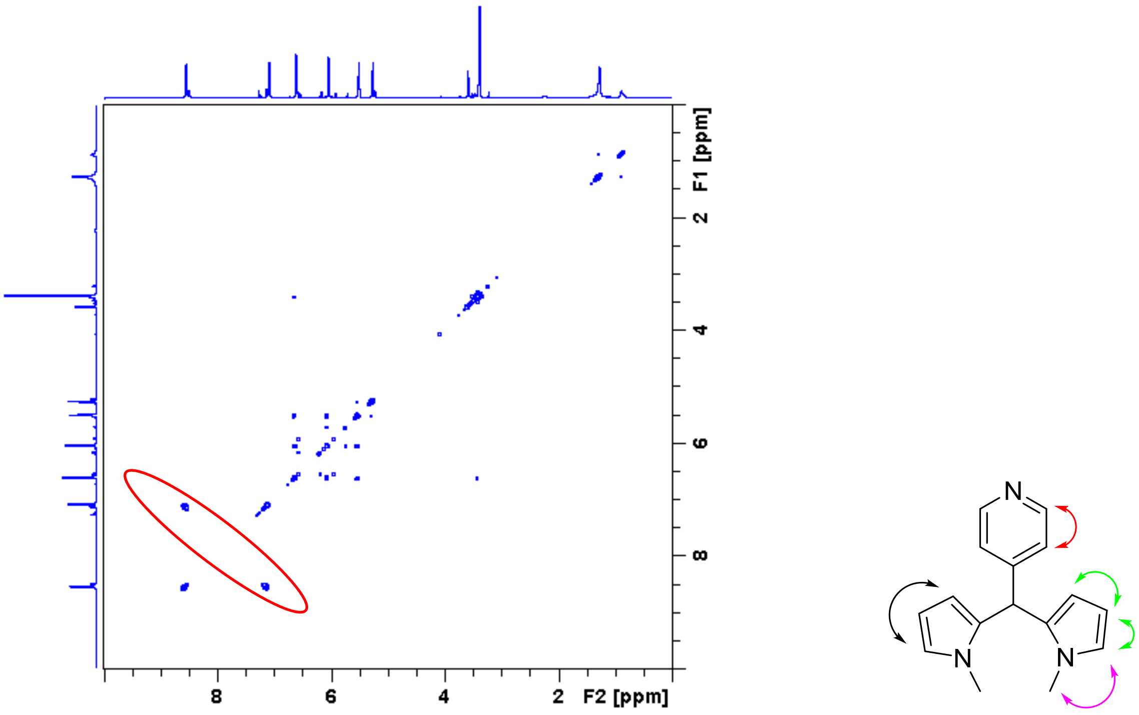

Figure S105. COSY NMR spectrum (400 MHz, $CDCl_3$, 298 K) of the derivative **11aj.**

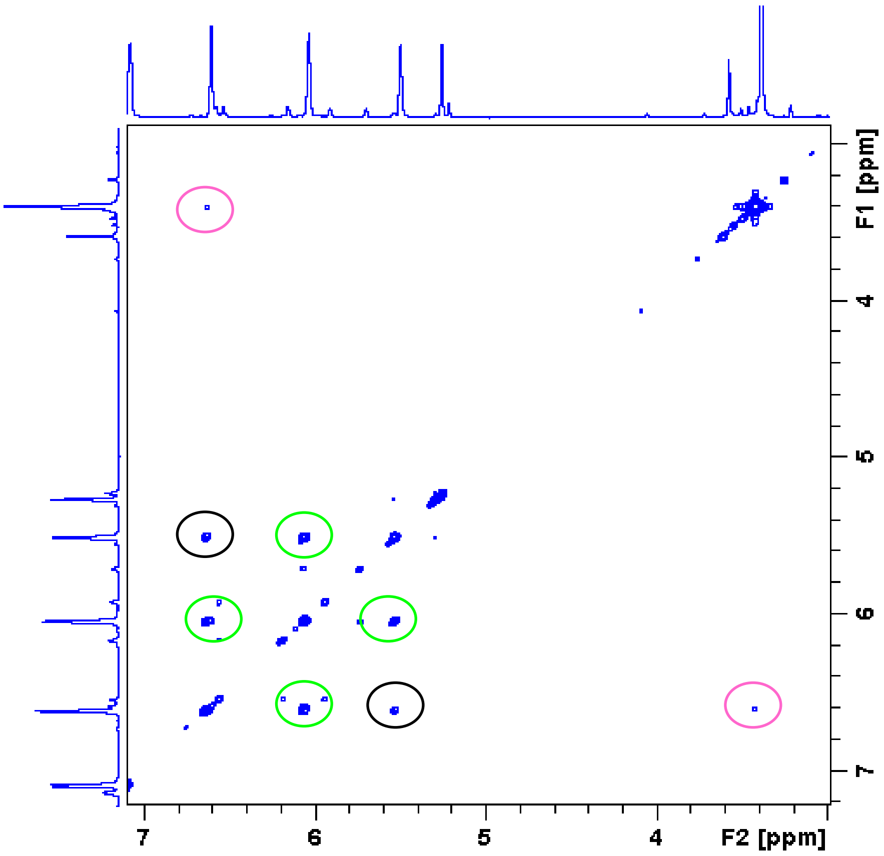

Figure S106. Relevant region of COSY NMR spectrum (400 MHz, $CDCl_3$, 298 K) of the derivative **11aj.**

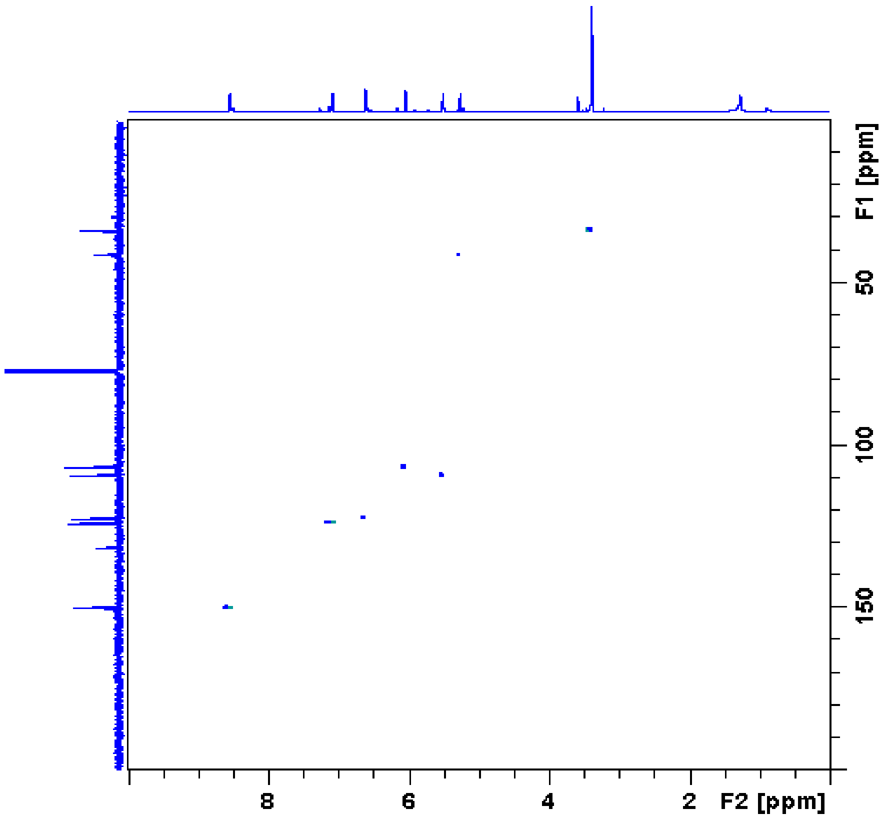

Figure S107. HSQC NMR spectrum (400 MHz, $CDCl_3$, 298 K) of the derivative **11aj.**

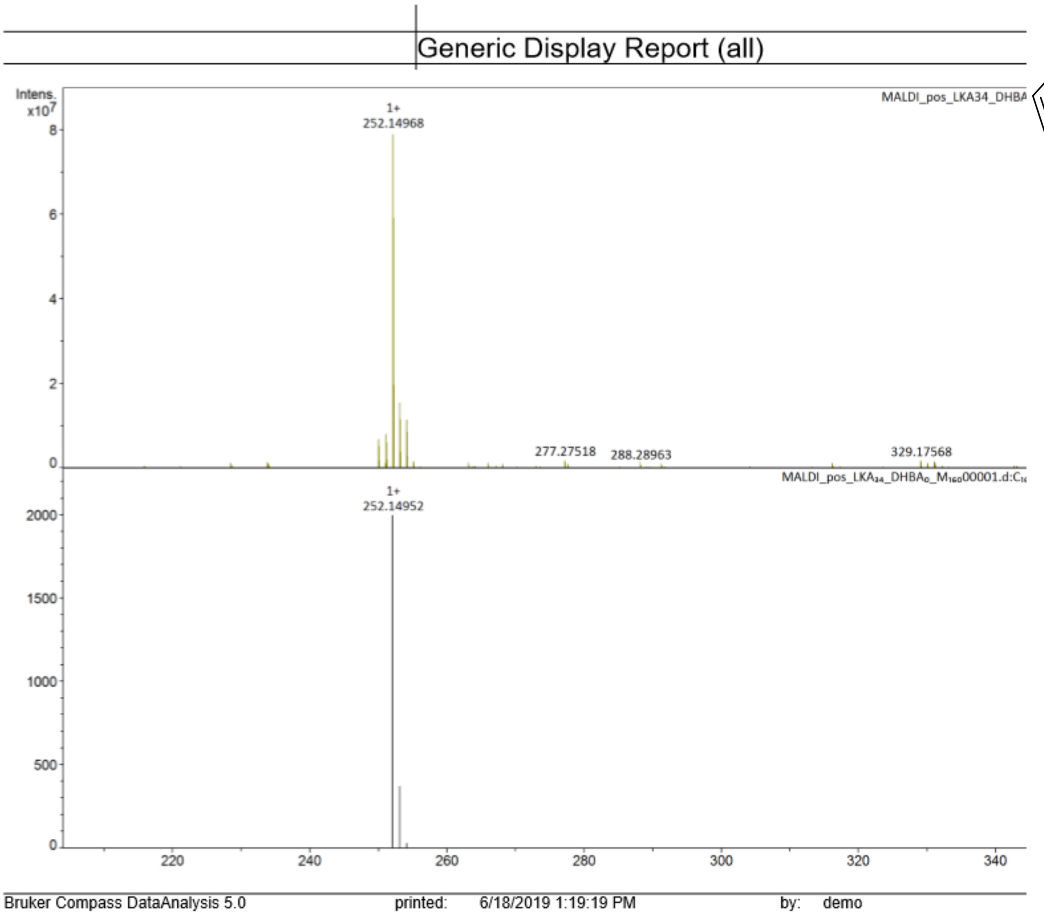

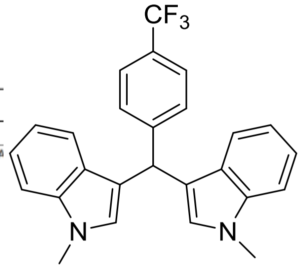

Figure S108. MALDI MS spectrum of the derivative **11aj.**

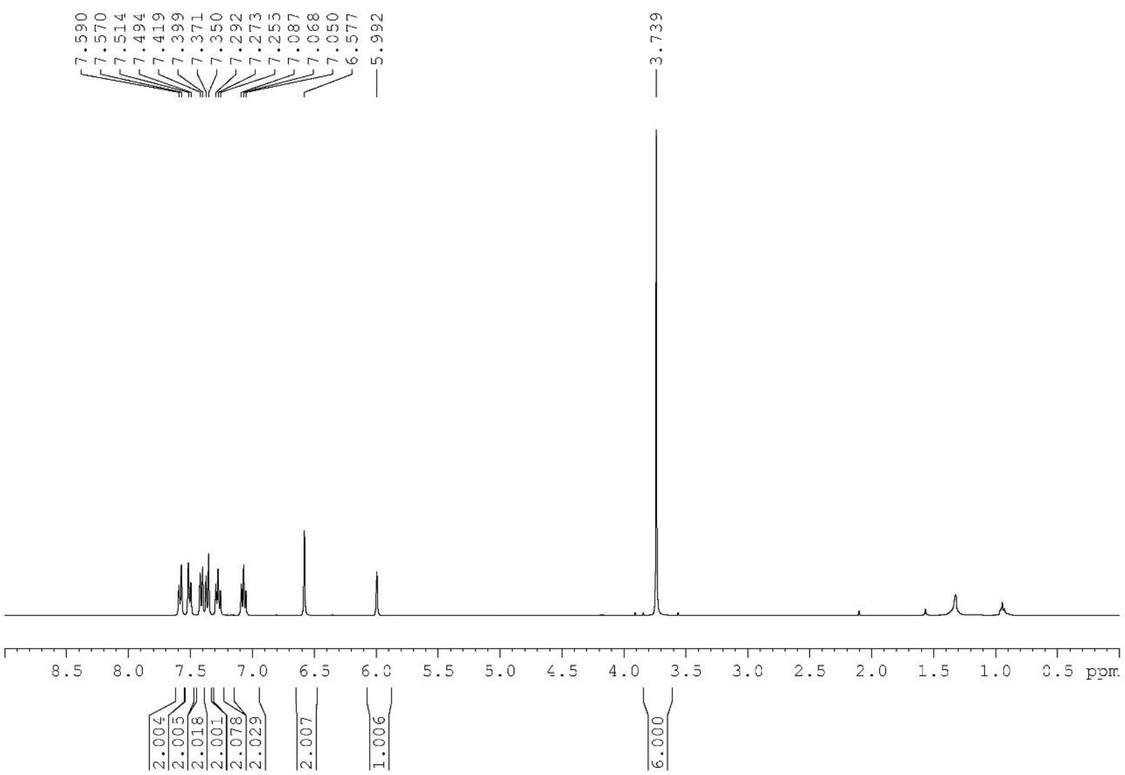

Figure

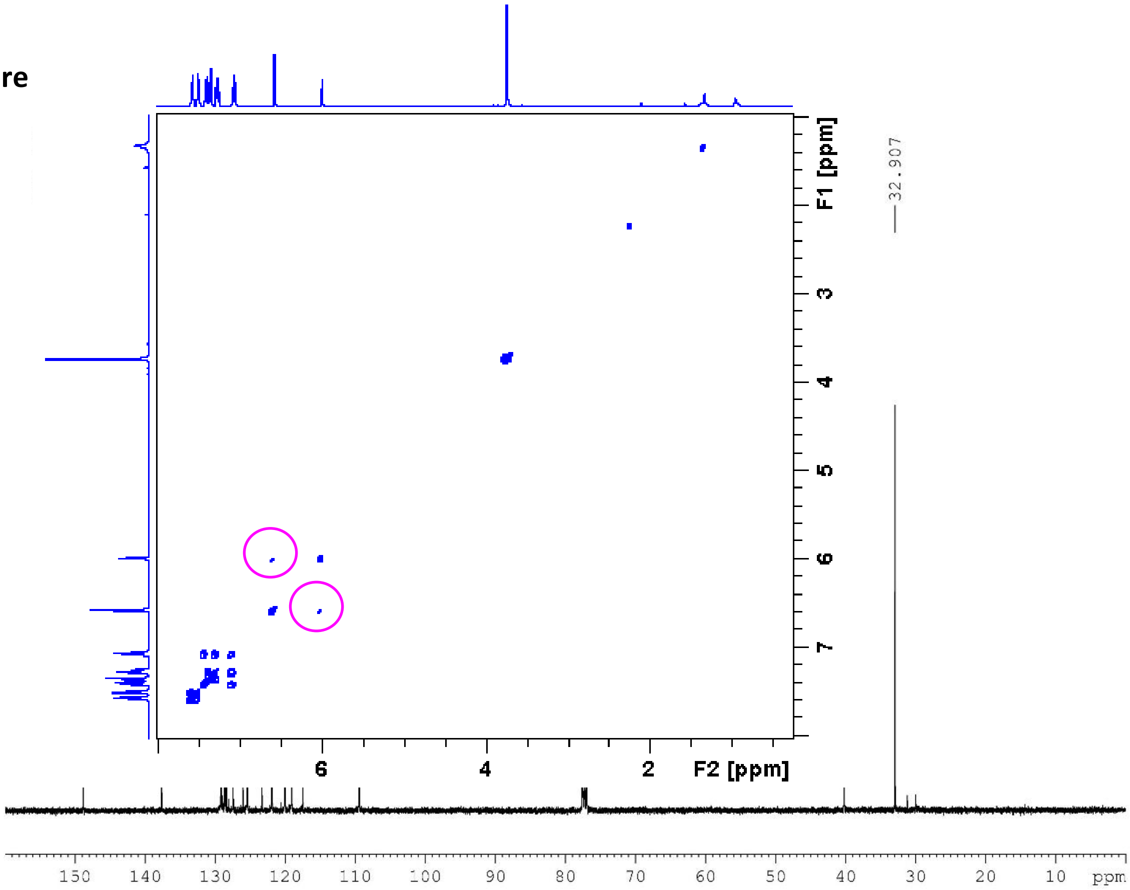

Figure S110. ^{13}C NMR (100 MHz, $CDCl_3$, 298 K) of the derivative **13ed.**

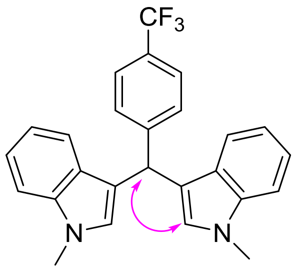

Figure S111. COSY NMR spectrum (400 MHz, $CDCl_3$, 298 K) of the derivative **13ed.**

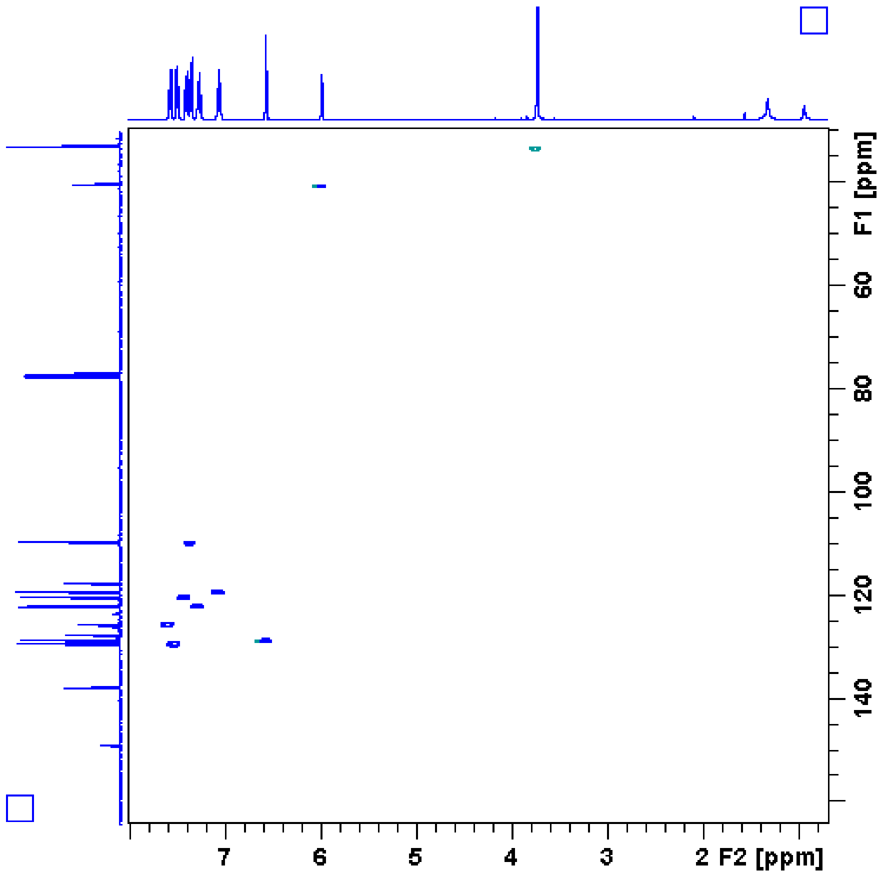

Figure S112. HSQC NMR spectrum (400 MHz, $CDCl_3$, 298 K) of the derivative **13ed.**

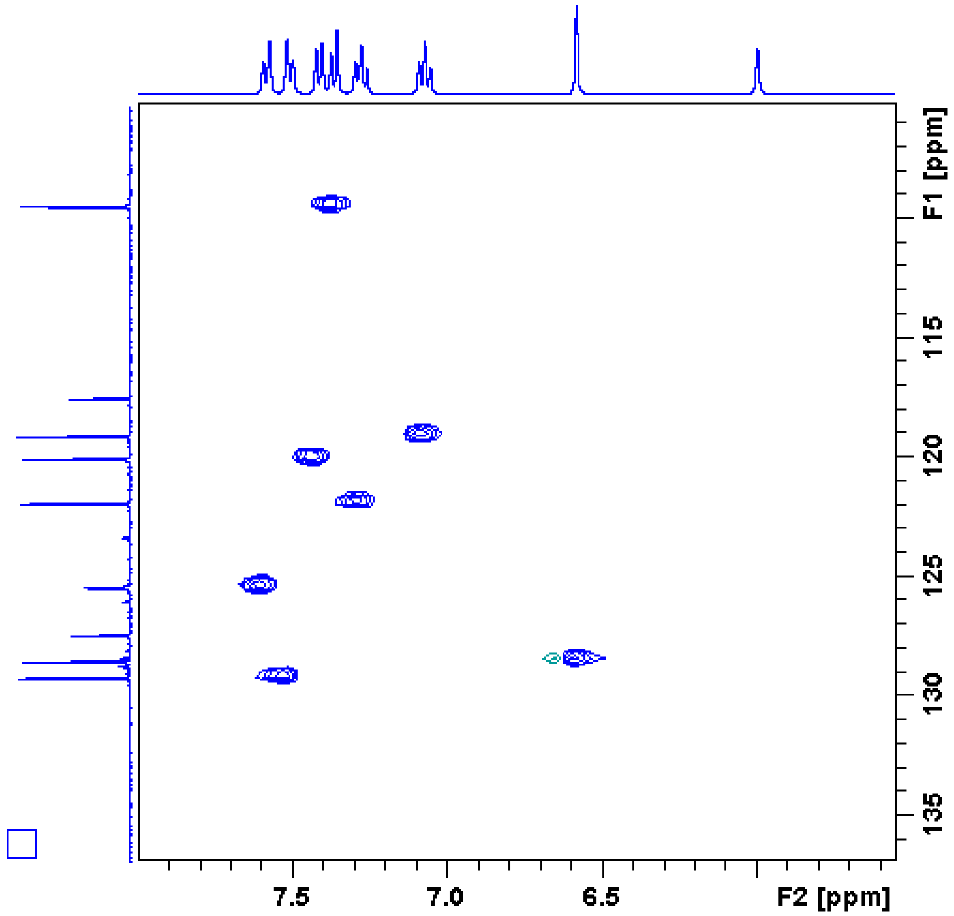

Figure S113. Expansion of HSQC NMR spectrum (400 MHz, $CDCl_3$, 298 K) of the derivative **13ed.**

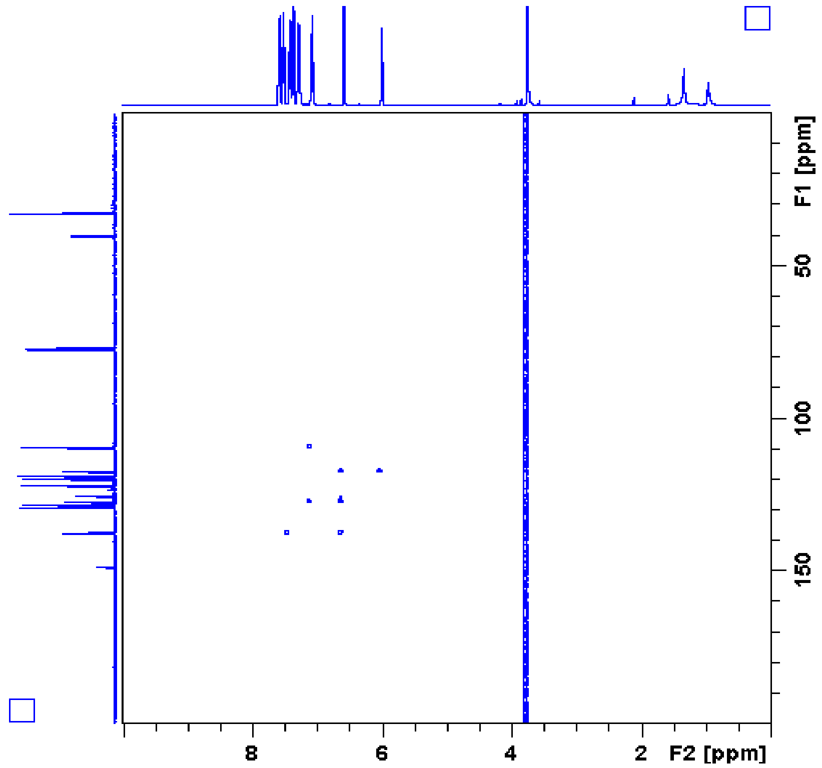

Figure S114. HMBC NMR spectrum (400 MHz, $CDCl_3$, 298 K) of the derivative **13ed.**

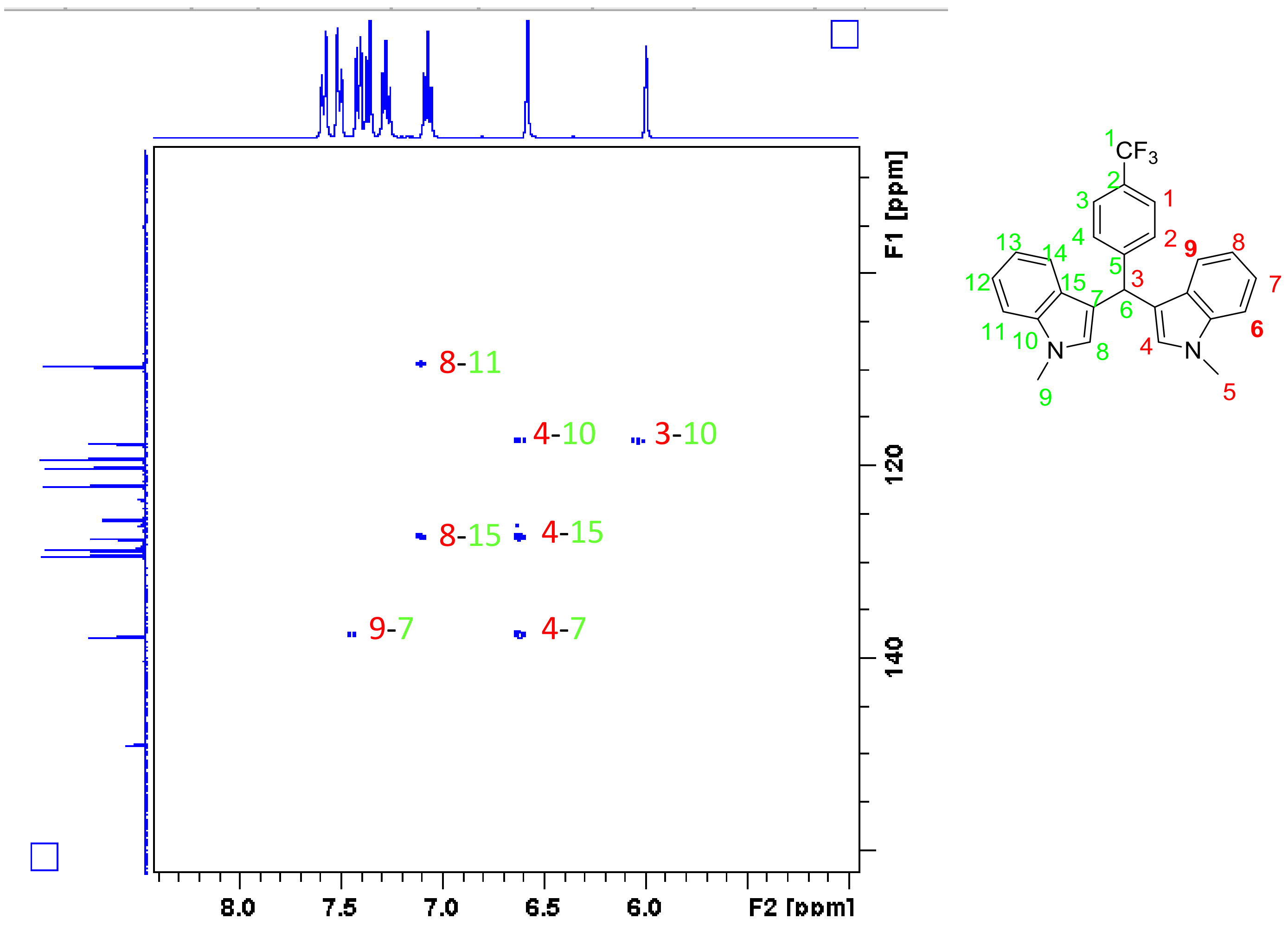

Figure S115. Expansion of HMBC NMR spectrum (400 MHz, $CDCl_3$, 298 K) of the derivative **13ed.**

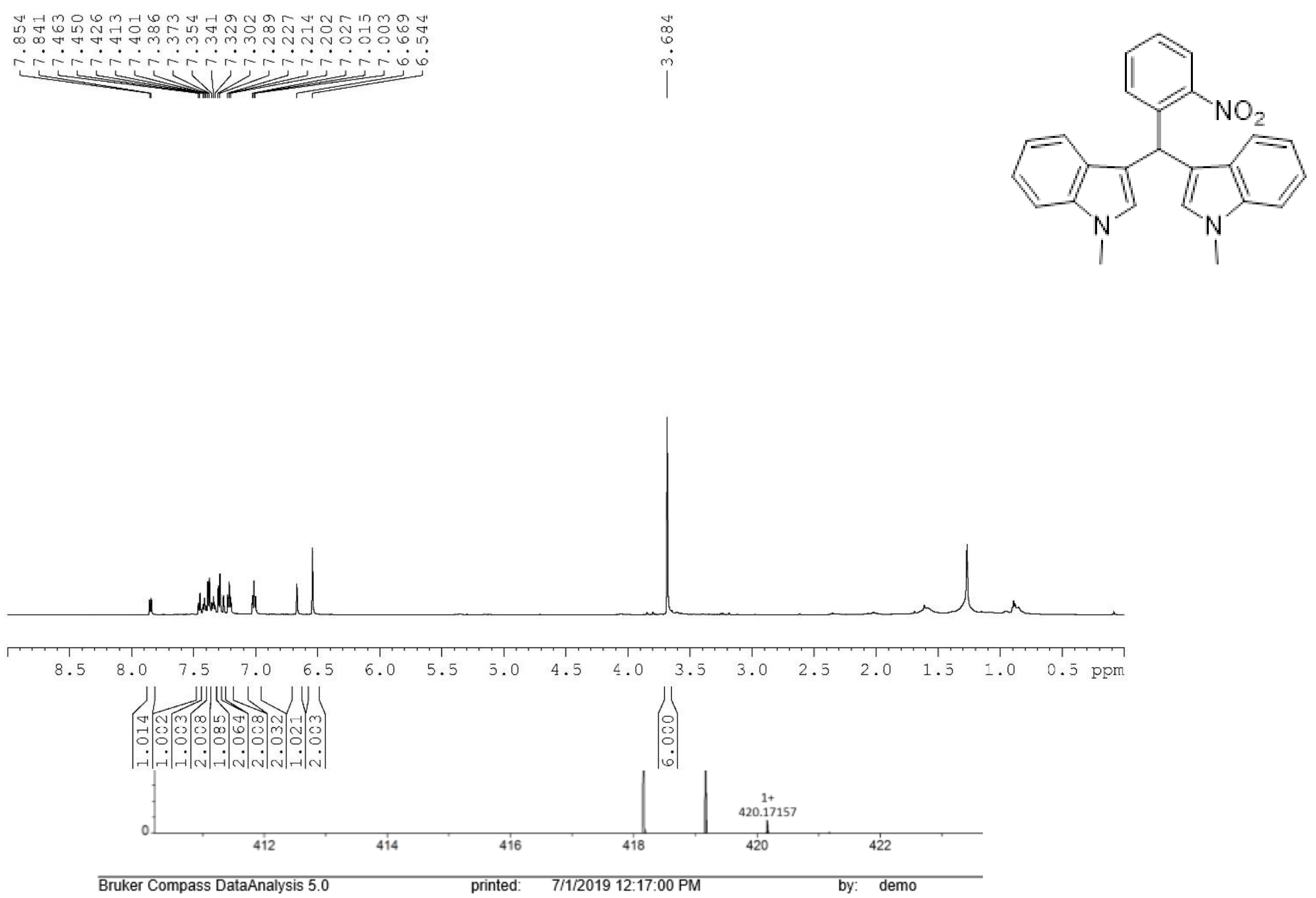

Figure S116. ESI MS spectrum of the derivative **13ed**

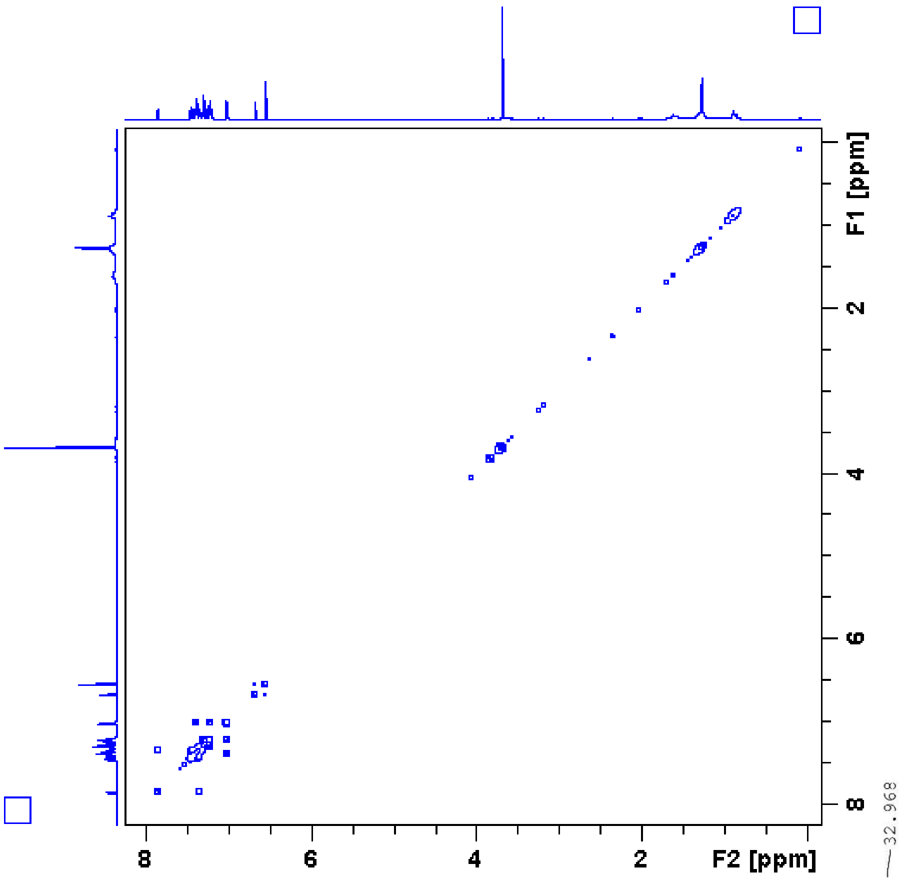

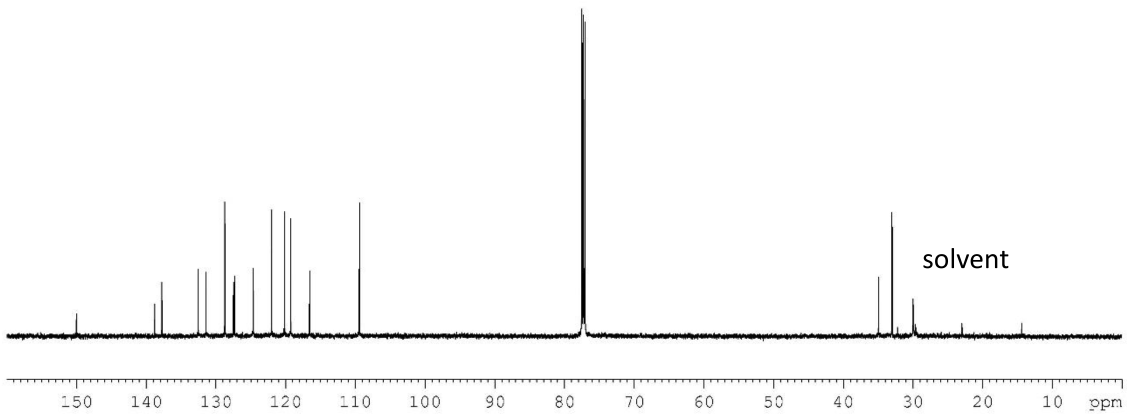

Figure S118. ^{1}H NMR (150 MHz, $CDCl_3$, 298 K) of the derivative **13eb.**

Figure S119. COSY NMR spectrum (600 MHz, $CDCl_3$, 298 K) of the derivative **13eb.**

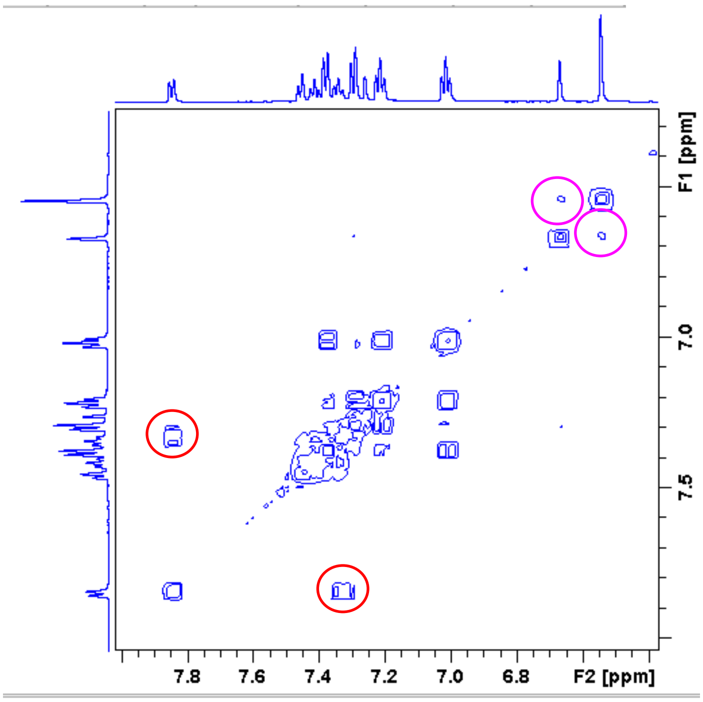

Figure S120. Expansion of COSY NMR spectrum (600 MHz, $CDCl_3$, 298 K) of the derivative **13eb.**

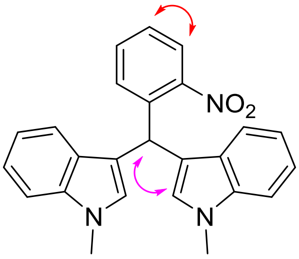

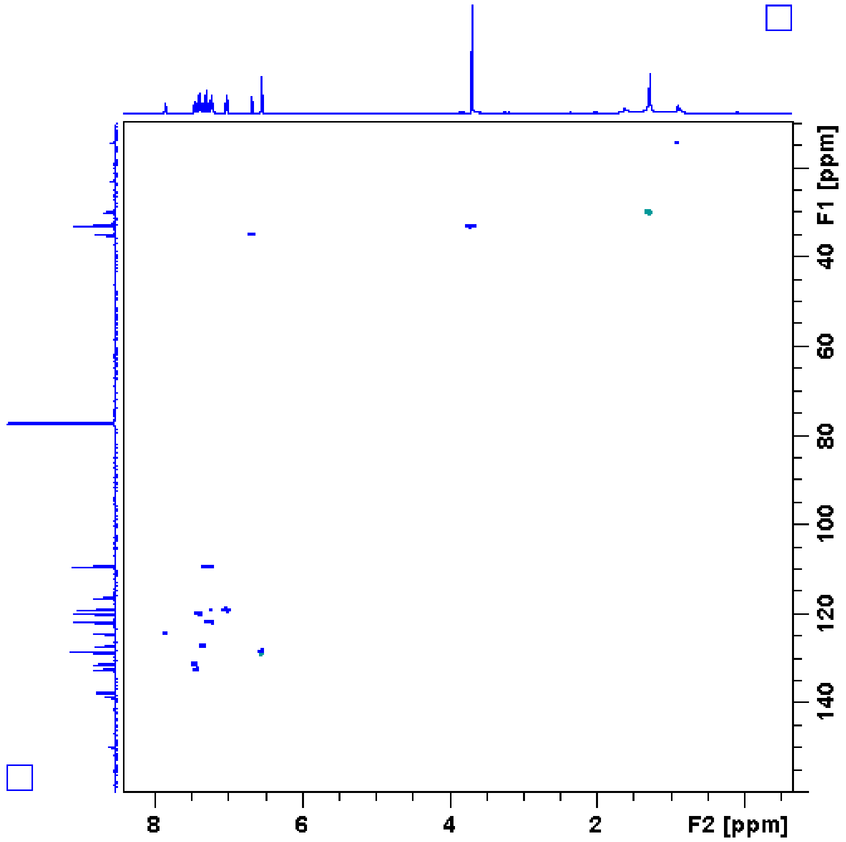

Figure S121. HSQC NMR spectrum (600 MHz, $CDCl_3$, 298 K) of the derivative **13eb.**

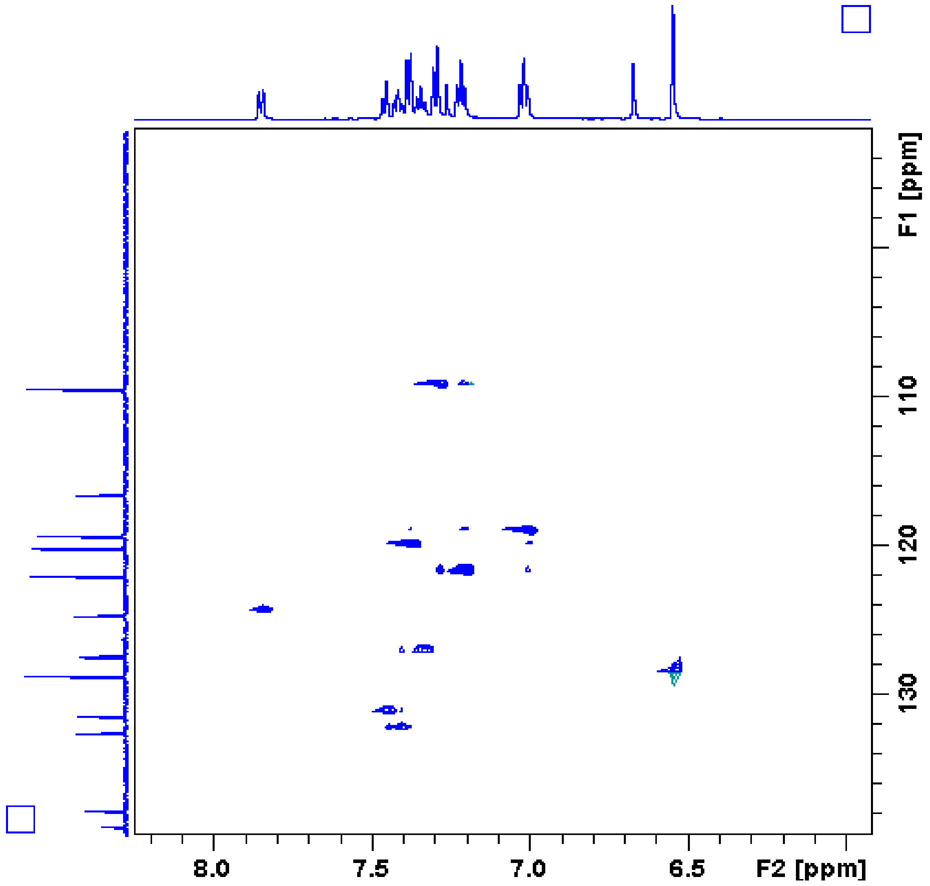

Figure S122. Expansion of HSQC NMR spectrum (600 MHz, $CDCl_3$, 298 K) of the derivative **13eb.**

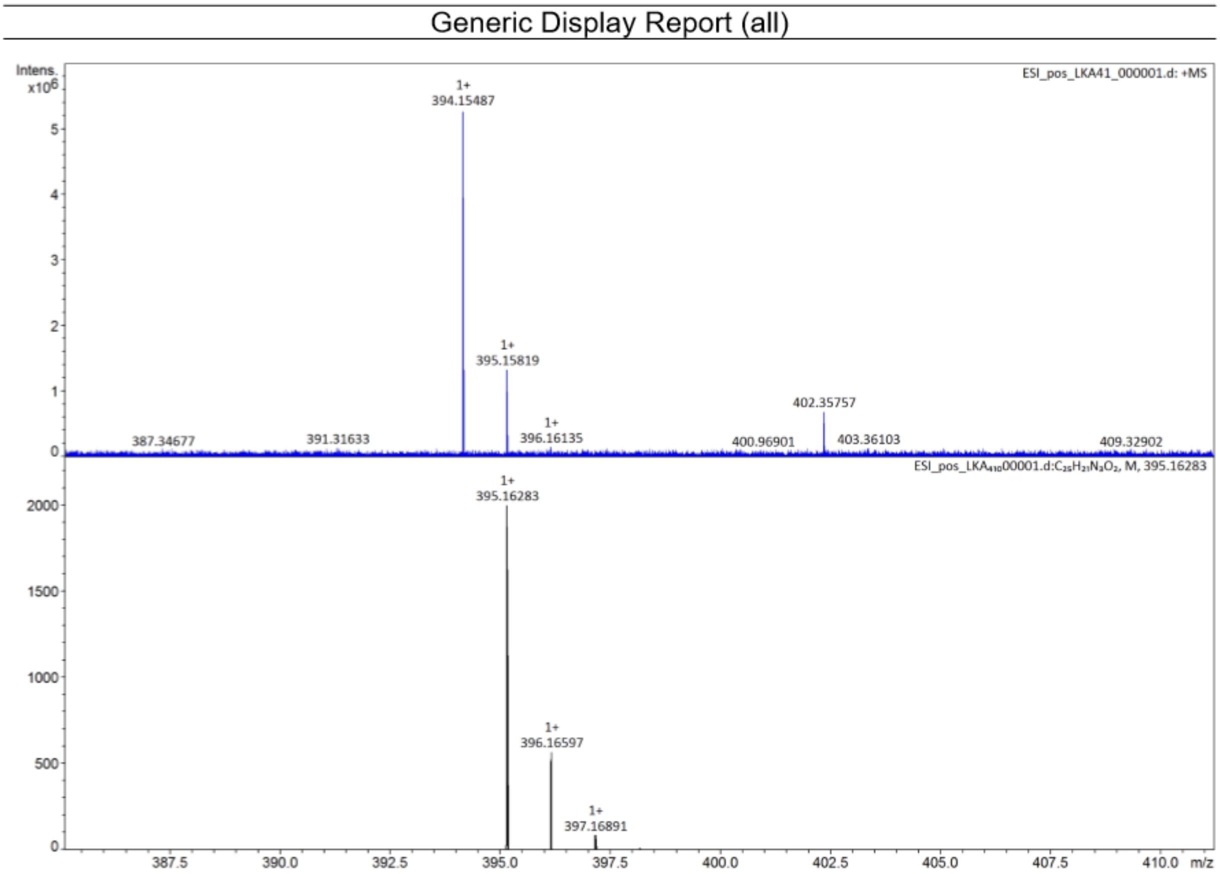

Figure S1234. ESI MS spectrum of the derivative **13eb.**

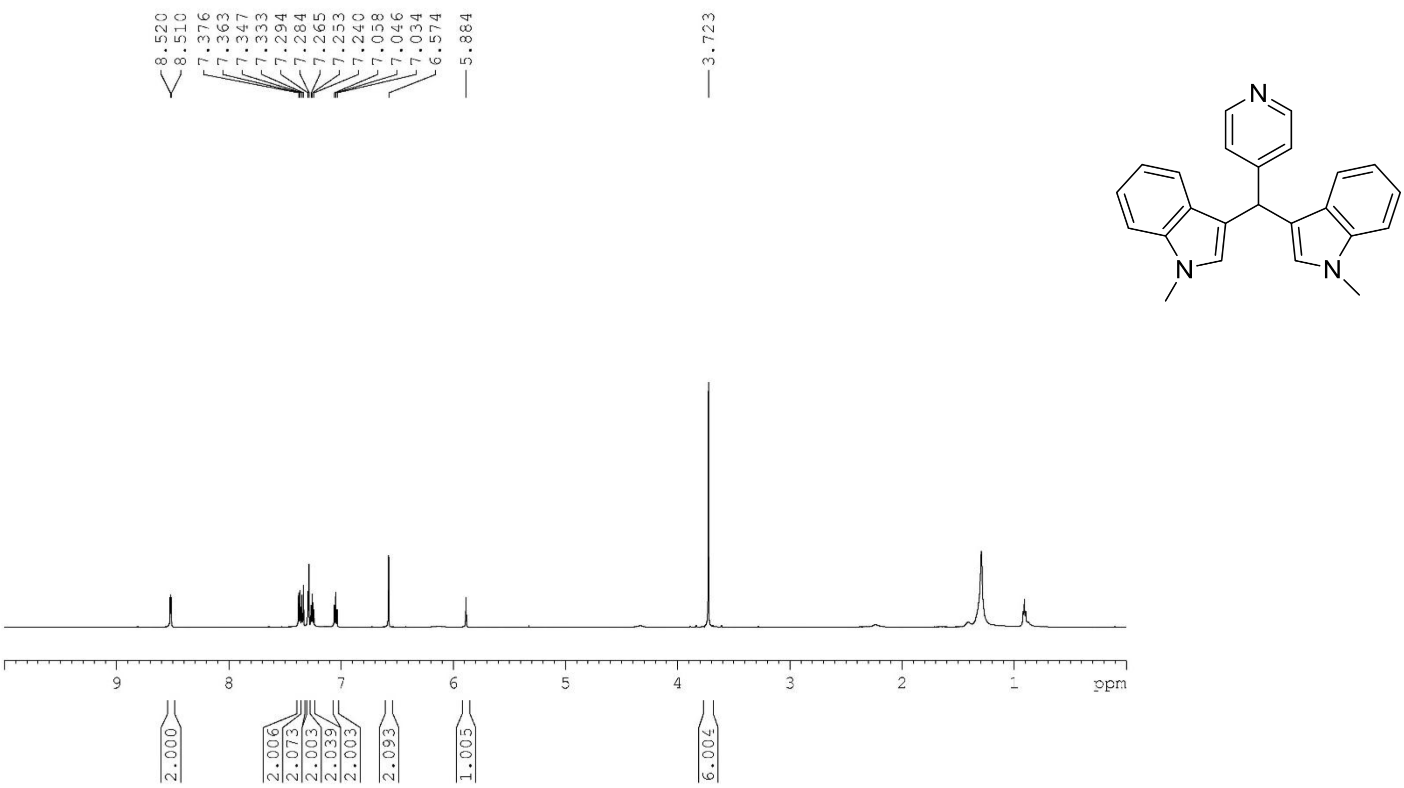

Figure S124. ^{1}H NMR (600 MHz, $CDCl_3$, 298 K) of the derivative **13ej**

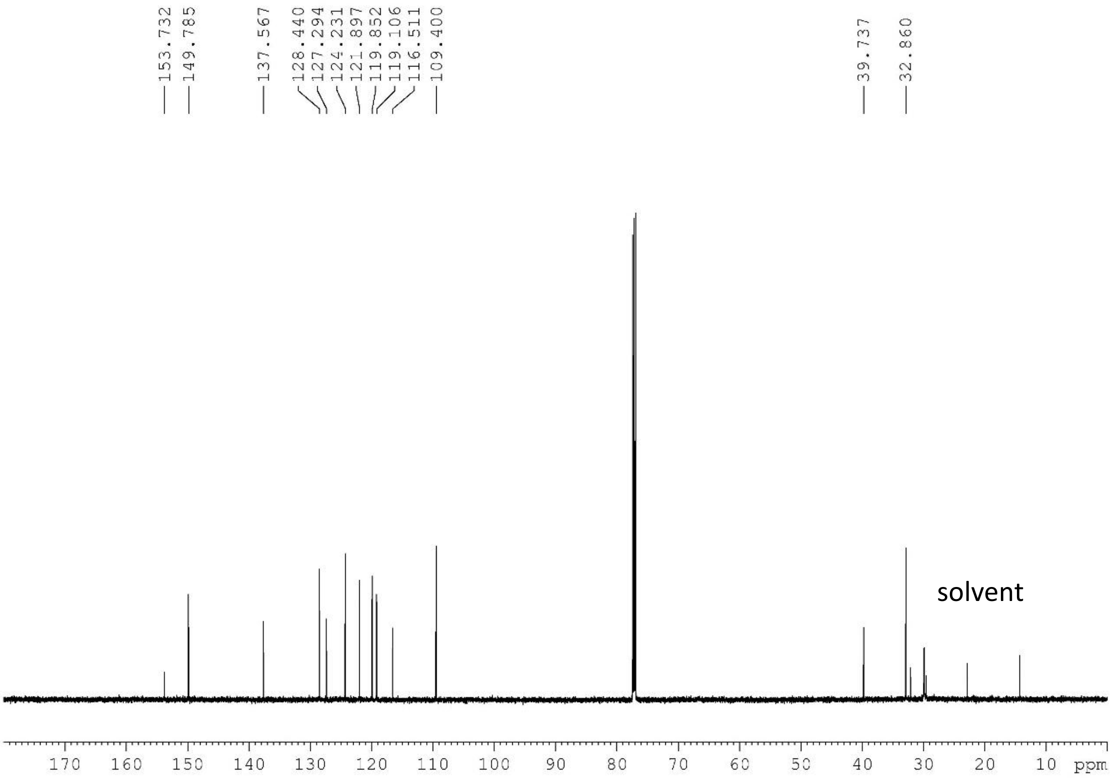

Figure S125. ^{13}C NMR (150 MHz, $CDCl_3$, 298 K) of the derivative **13ej.**

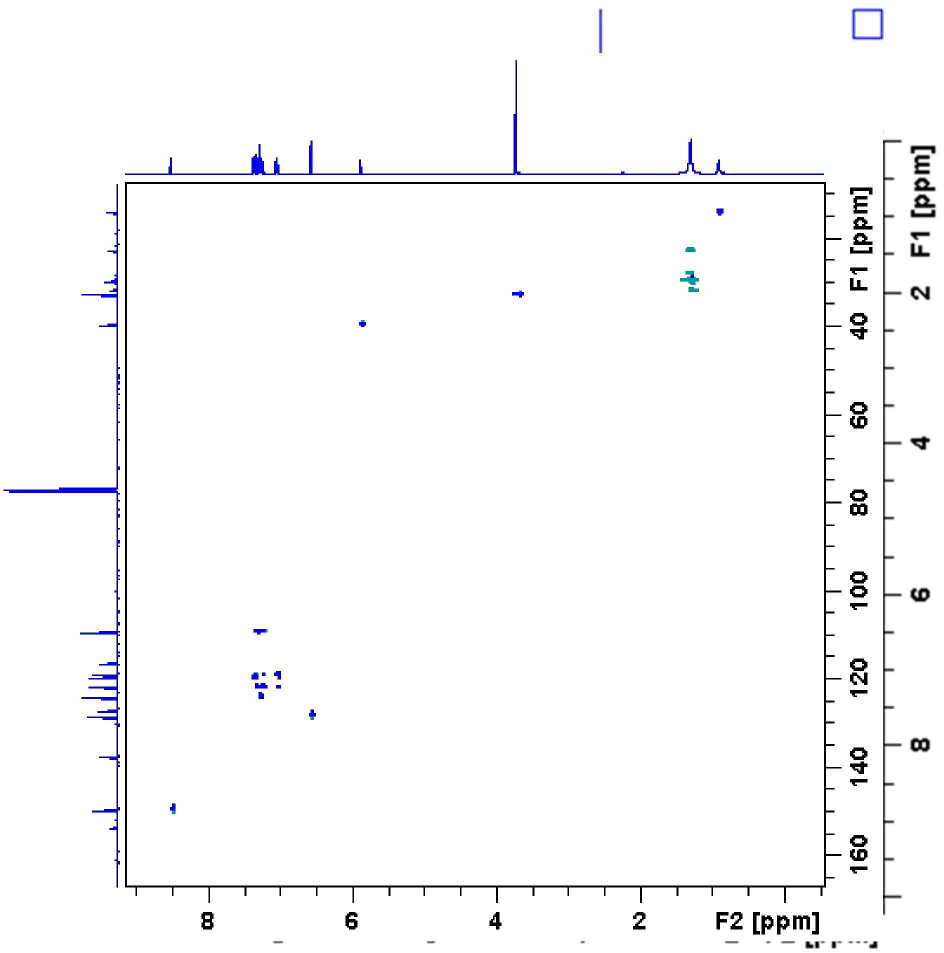

Figure S126. COSY NMR spectrum (400 MHz, $CDCl_3$, 298 K) of the derivative **13ej.**

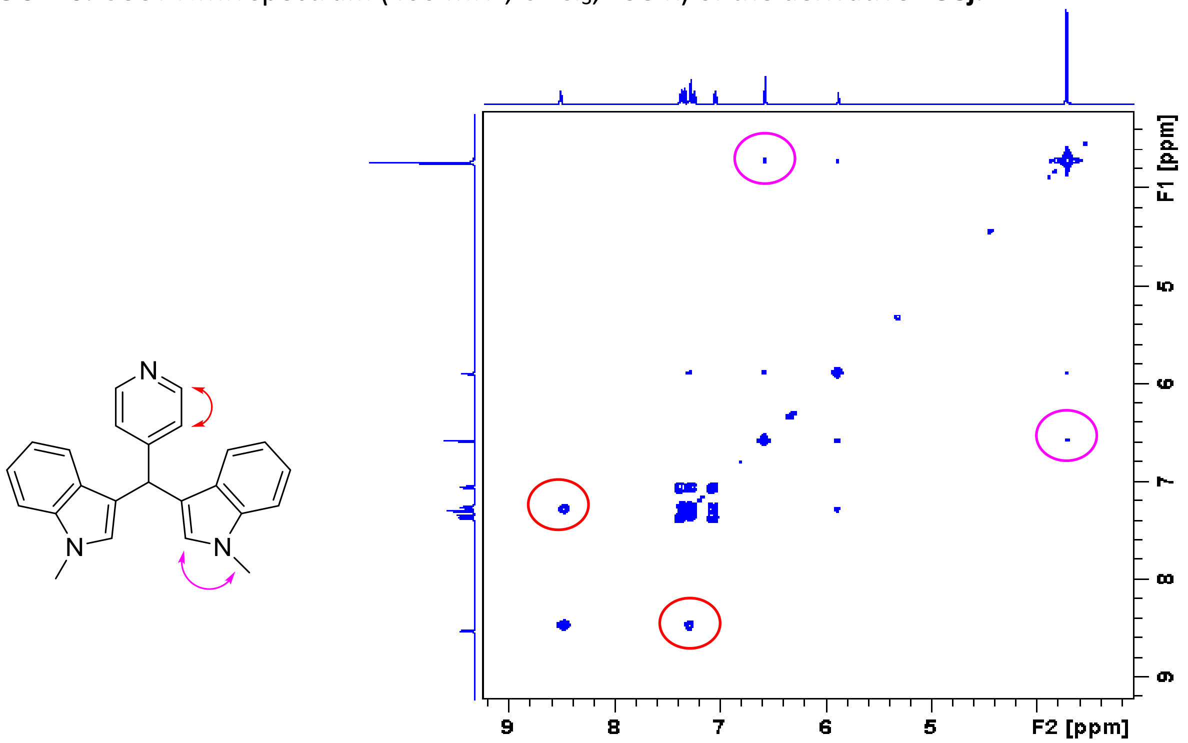

Figure S127. Relevant region of COSY NMR spectrum (400 MHz, $CDCl_3$, 298 K) of the derivative **13ej.**

Figure S128. HSQC NMR spectrum (600 MHz, $CDCl_3$, 298 K) of the derivative**13ej.**

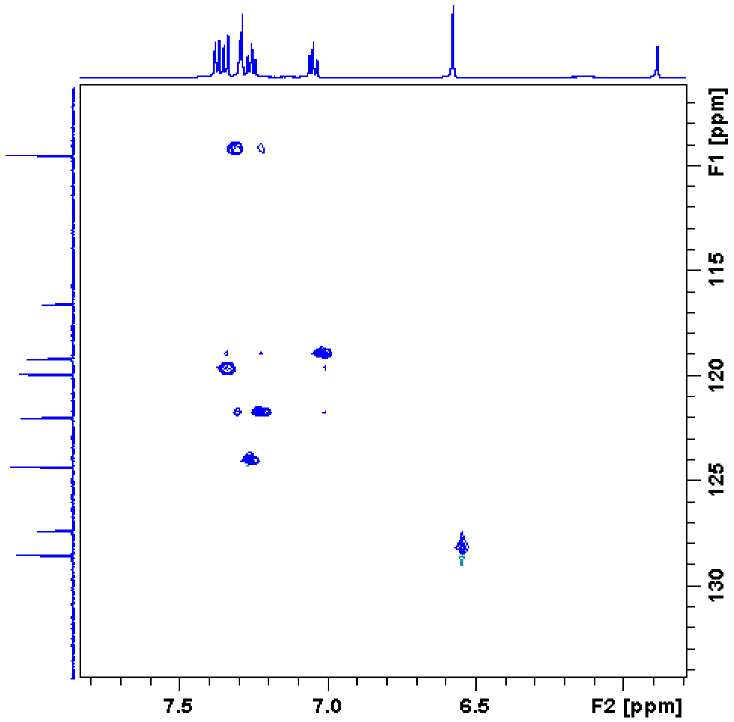

Figure S129. of the derivative **13ej.**

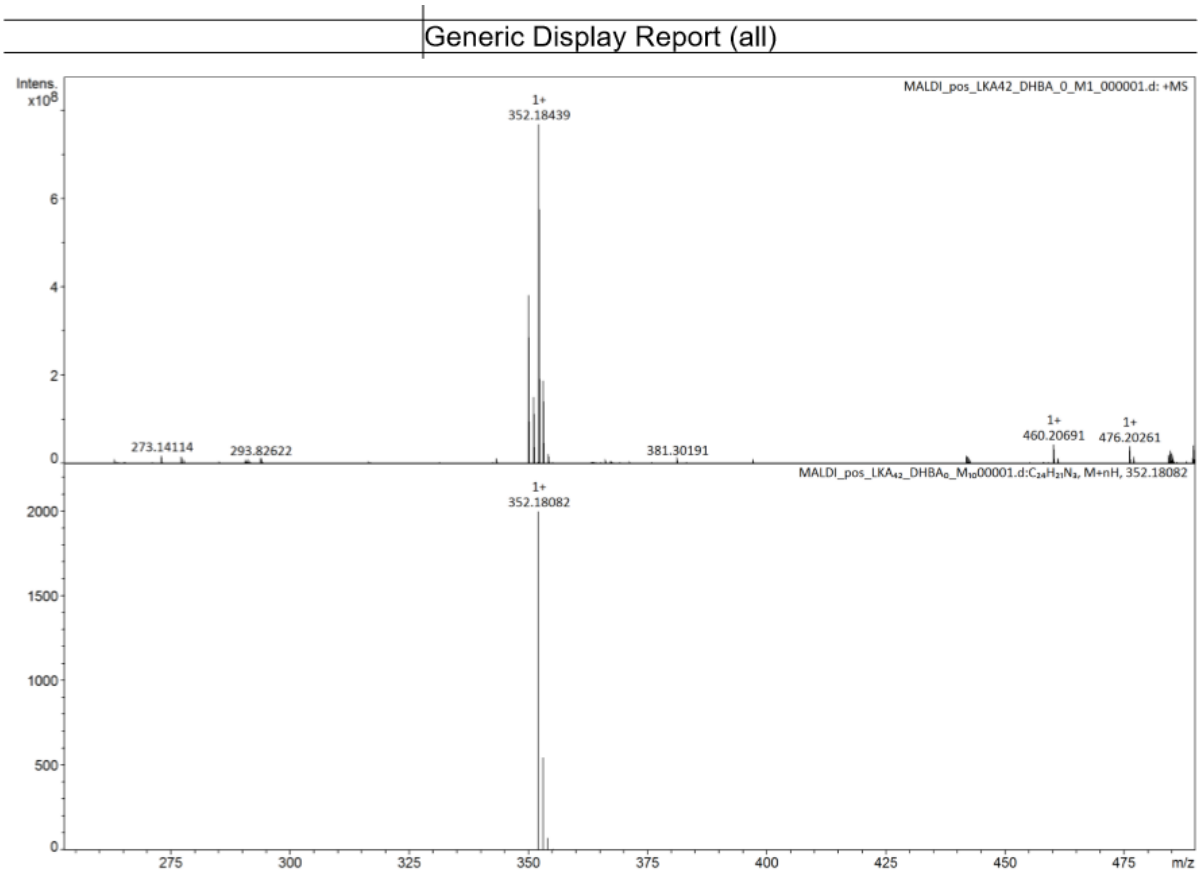

Figure S130. MALDI MS spectrum of the derivative **13ej.**

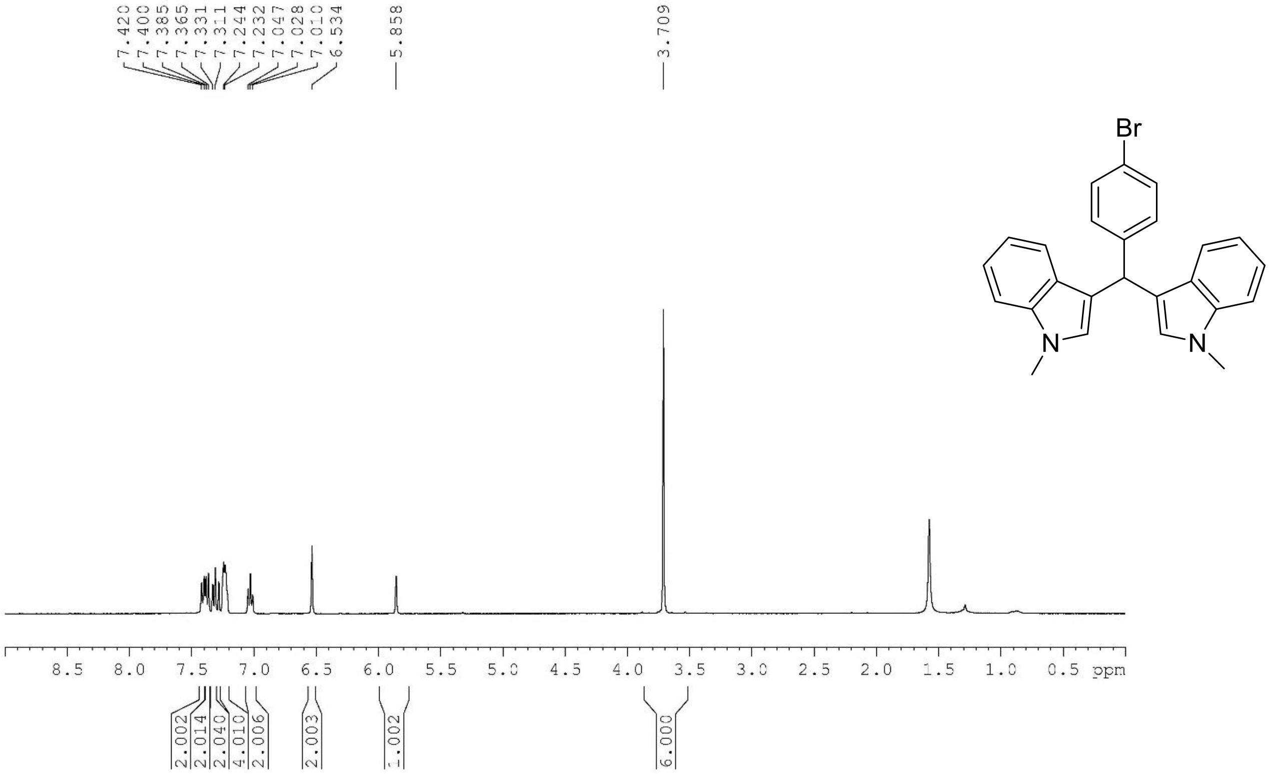

Figure S131. ^{1}H NMR (300 MHz, $CDCl_3$, 298 K) of the derivative **13ee.**

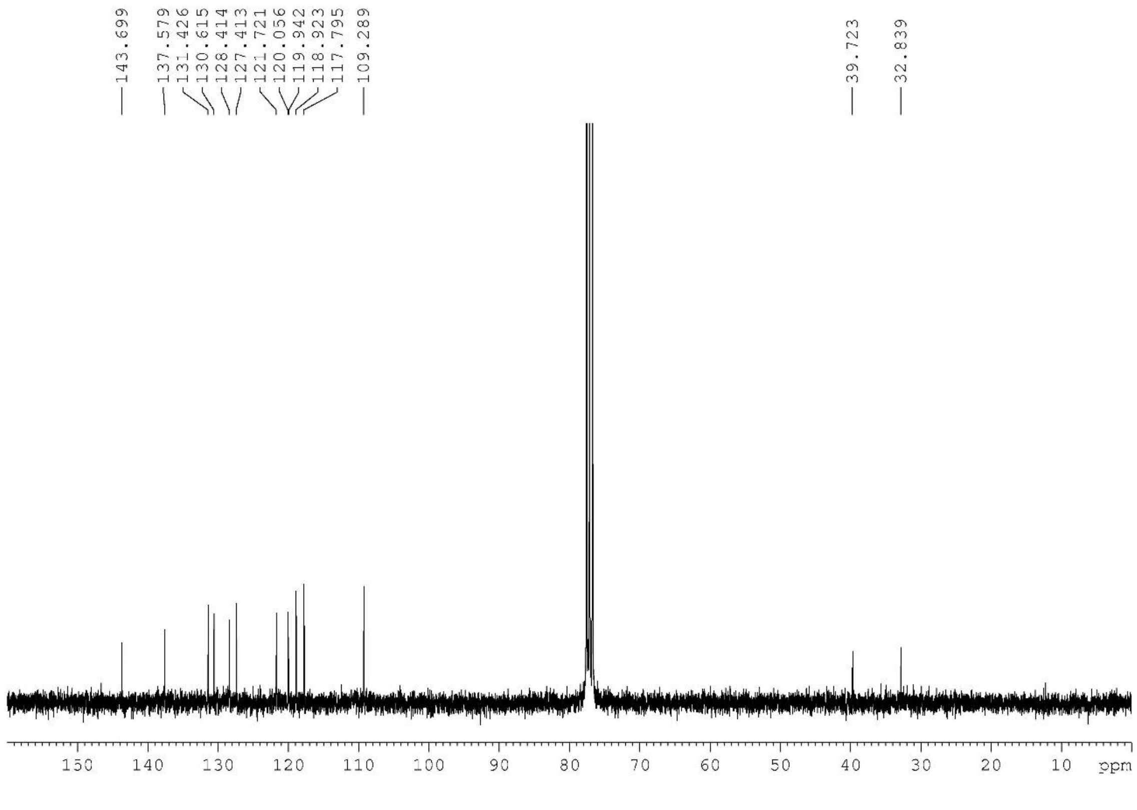

Figure 132. ^{13}C NMR (75 MHz, $CDCl_3$, 298 K) of the derivative **13ee.**

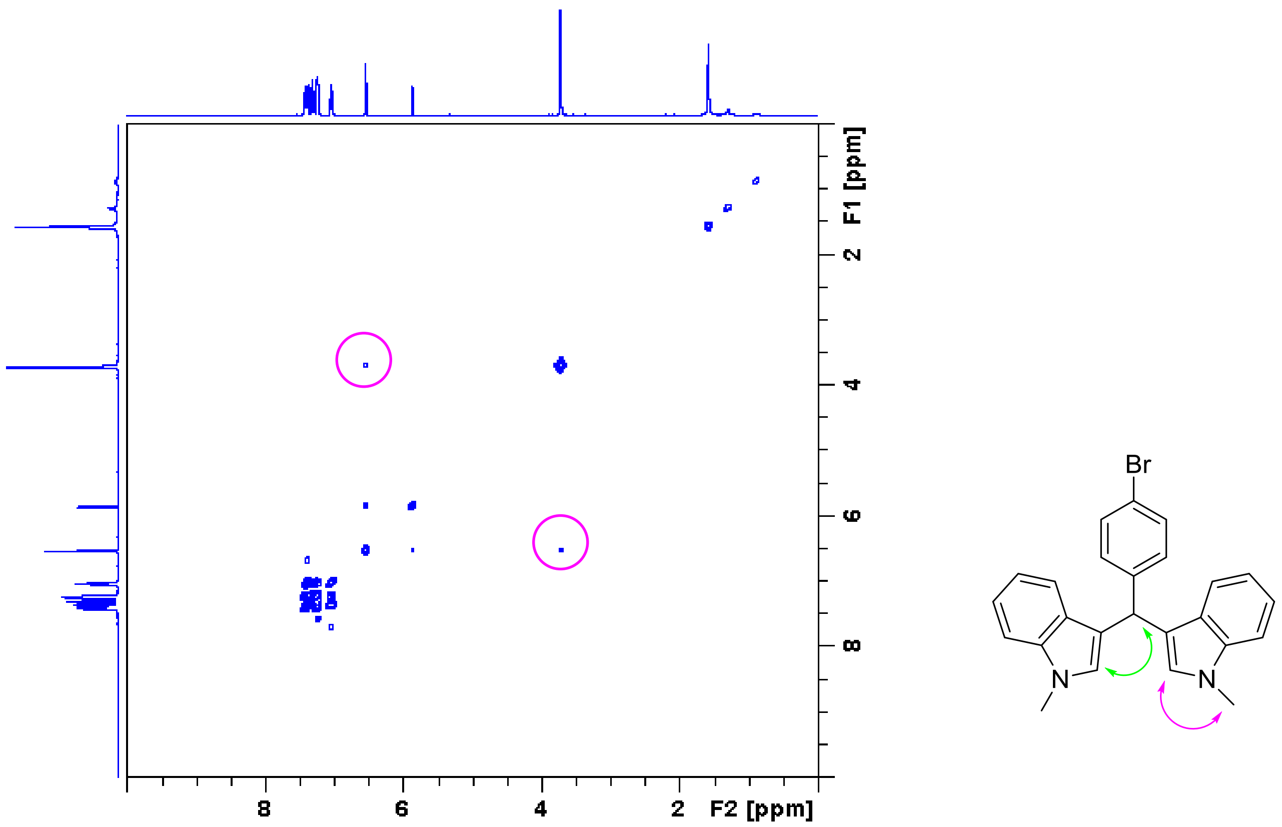

Figure S133. COSY NMR spectrum (300 MHz, $CDCl_3$, 298 K) of the derivative **13ee.**

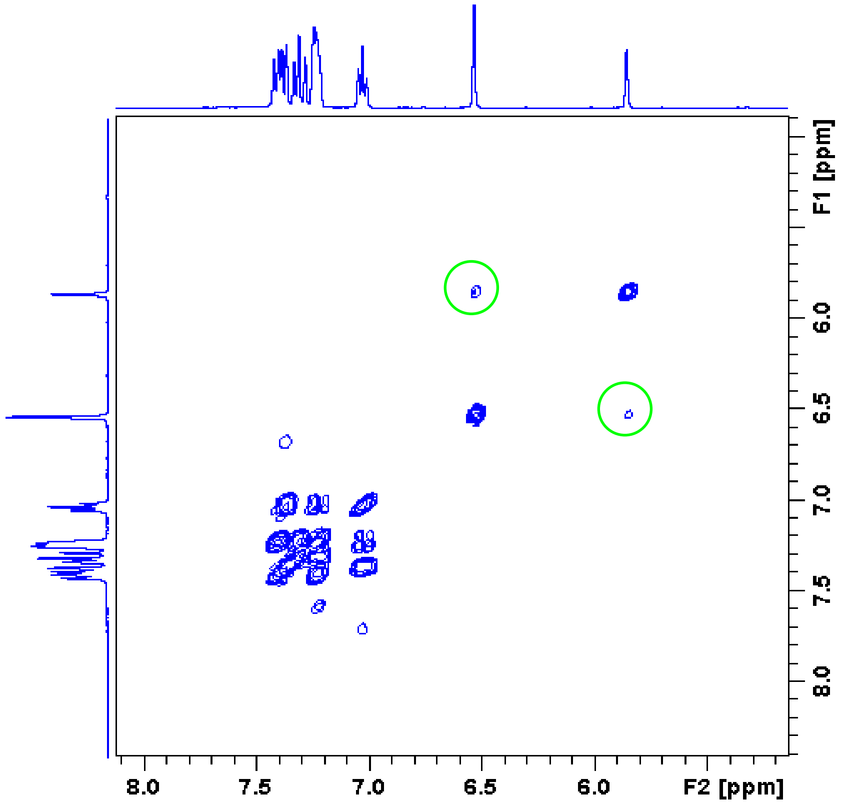

Figure S134. Relevant region of COSY NMR spectrum (300 MHz, $CDCl_3$, 298 K) of the derivative **13ee.**

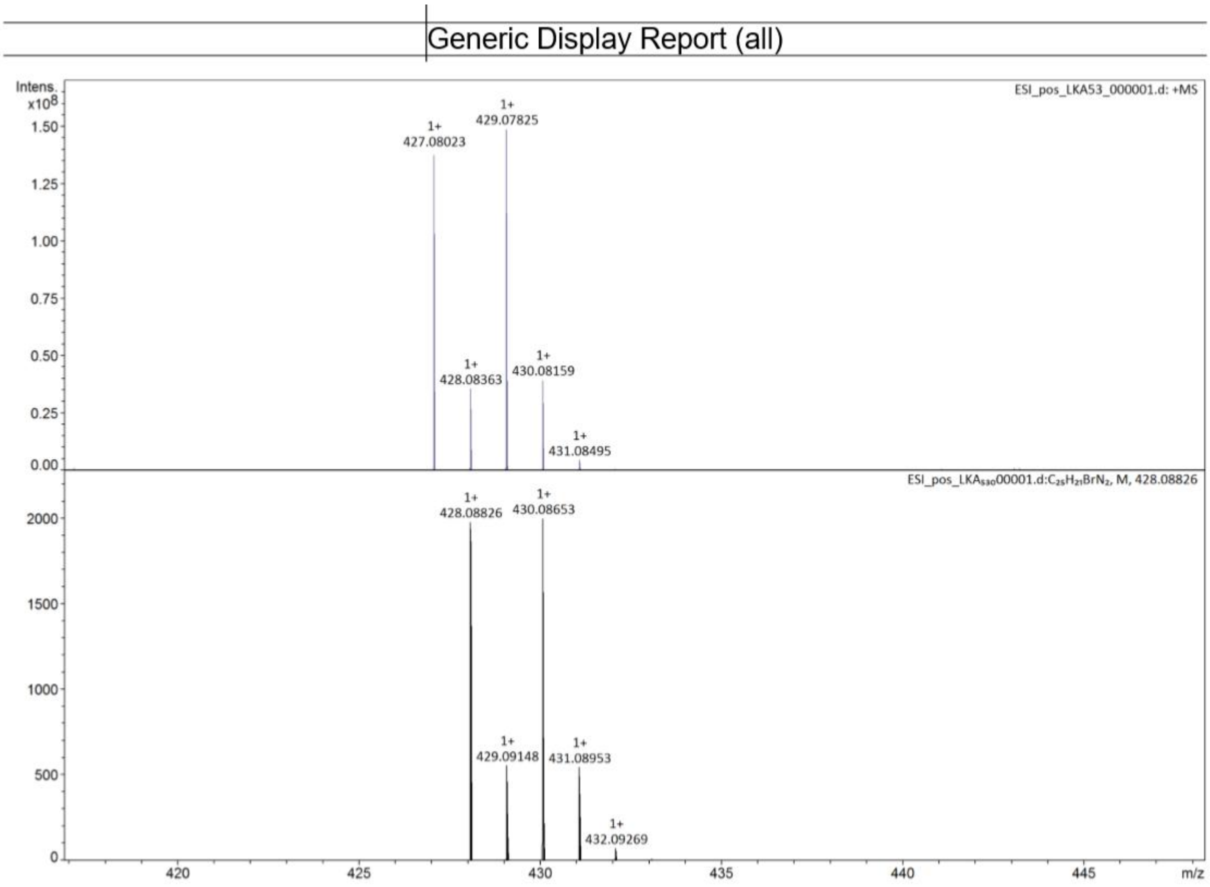

Figure S135. ESI MS spectrum of the derivative **13ee.**
